# Proceedings of the 24^th^ Paediatric Rheumatology European Society Congress: Part two: Athens, Greece. 14-17 September 2017

**DOI:** 10.1186/s12969-017-0186-9

**Published:** 2017-09-01

## Vasculitides

### P164 Evaluation of children with Behçet’s disease from regionally two different centers of a highly prevalent country

#### Nuray Aktay Ayaz^1^, Betül Sözeri^2^, Mustafa Çakan^1^, Zübeyde Gündüz^3^, Ayşenur Paç Kısaarslan^3^, Ruhan Düşünsel^3^, Hakan Poyrazoğlu^3^

##### ^1^Pediatric Rheumatology, Kanuni Sultan Süleyman Research And Training Hospital, İstanbul, Turke; ^2^Pediatric Rheumatology, Ümraniye Research And Training Hospital, İstanbul, Turkey; ^3^Pediatric Rheumatology, Erciyes University, Kayseri, Turkey

###### **Correspondence:** Nuray Aktay Ayaz

**Introduction:** Behçet’s disease is a complex multisystemic disease with variable vasculitis. The disease is most prevalent in the Eastern Mediterranean, the Middle East, and East Asia, along the ancient Silk Road. The typical triad is oral ulcers, genital ulcers and ocular inflammation. But other clinical manifestations due to vasculitis and thrombosis, involvement of the gastrointestinal system, nervous system and musculoskeletal system can be seen variably. Although it is a disease of young adulthood, one-forth of cases are diagnosed during childhood. There is a long interval before onset of enough manifestations to satisfy adult Behçet’s disease criteria, so a new subset of consensus classification criteria was put forward by an international Behçet’s expert group.

**Objectives:** To evaluate the demographic data, clinical features and treatment modalities of children with Behçet’s disease and to display the relationship of HLA B51 genotype with mild and severe system involvements of pediatric Behçet’s disease patients.

**Methods:** Two pediatric rheumatology centers; one from West and one from East of Turkey collected the data of patients with pediatric Behçet’s disease retrospectively between January 2010 and May 2016. The children were enrolled to the study if they fulfilled the Internationational classifications criteria for Behçet’s disease. Children diagnosed ≤16 years of age were included to the study. The demographic, clinical, laboratory and medication reports were collected from data sheets.

**Results:** Fifty-four patients were included; 23 male (42,5%), 31 female (57.5%). Family history of Behçet’s disease in a first-degree relative was present in 17 (31.5%) of cases. Of them, 11 (31,4%) were HLA B51 positive. The mean age of the patients was 15,1 (8-21) years. The mean age of onset was 11,1 (5-15,6) years. The mean age at diagnosis was 12,9 (6-16) years. All the patients had oral ulceration. Genital ulcers were present at 34 (62,9%) patients. Skin findings were present in 35 (64,8%) patients. Ten patients (18,5%) had uveitis during the course of disease. Seven patients (13%) had thrombosis. Gastrointestinal involvement was reported in 15 patients (27,8%) and arthralgia/arthritis was present at 29 patients (53,7%). Five patients (9,3%) had neurological signs and symptoms. ANA positivity was seen at 9 (17%) patients. HLA B51 positivity was detected at 35 (64,8%) children of whom 15 (42,8%) had a mild course of disease while 20 (57,2%) had a severe course with major organ involvement. All the patients were under colchicine treatment, 16 of them had aditional DMARD (azathiopurine or methotrexate) and 2 of them had biologic treatment. Steroids were used by 15 patients. The age that the first symptom ensues was significantly lower in males (9,9 vs 12,8). No relationship with the family history and age of onset of the disease, severity of the disease was shown. The age of onset, the clinical features, treatment modalities reported from two centers from different regions of country were very similar to each other.

**Conclusion:** This is a retrospective evaluation of a large series of children with Behçet’s disease from a country with high prevalance. HLA-B51 positivity does not seem to help to predict the course of the disease.

**Disclosure of Interest**

None Declared.

### P165 Evaluation of cognitive function electrophysiologically in children with Behçet disease

#### Nuray Aktay Ayaz^1^, Gökçer Eskikurt^2^, Gonca Keskindemirci^1^, Mustafa Çakan^1^, Numan Ermutlu^3^, Ümmühan İşoğlu Alkaç^4^

##### ^1^Pediatric Rheumatology, Kanuni Sultan Süleyman Research And Training Hospital, İstanbul, Turkey; ^2^Neuroscience, Istanbul University; ^3^Physiology, Istanbul Bilim University, İstanbul, Turkey; ^4^Physiology, Istanbul University, İstanbul, Turkey

###### **Correspondence:** Nuray Aktay Ayaz

**Introduction:** Behçet disease (BD) is a systemic inflammatory condition characterized by recurrent oral aphthae and several systemic manifestations including genital aphthae, skin lesions, ocular, gastrointestinal, neurologic and vascular involvement, and arthritis. Generally, recurrent oral aphthous ulcers are the first symptoms and may begin during early childhood and in 4-26% of the cases full clinical picture is completed before the age of 16. Central nervous system involvement is uncommon but it is one of the most severe manifestation of pediatric BD. Evoked response potentials (ERPs) are commonly used as physiological measures of cognitive function as they are easily measured and noninvasive.

**Objectives:** This study was conducted to assess the cognitive function of children with the diagnosis of BD without neurological involvement.

**Methods:** Children included to the study were diagnosed as BD according to ISG criteria. Nine children with BD and 9 controls were enrolled in this study. All participants were good at school performance and examined by pediatric psychiatrist. Patients with any neurological symptoms were excluded from the study. The EEG were recorded from 19 scalp electrode sites. During the EEG recording, all participants were instructed to discriminate auditorially and visually the rare stimuli (target stimuli) from the frequent stimuli (standard stimuli) and to press the button of the mouse immediately following the target stimulus to perform ERPs and P300.

**Results:** Neurologic involvement in BD has a wide spectrum of symptoms consisting of acute type and chronic progressive type. Subclinical neurological involvement without neurological symptoms may also be possible. We aimed to look for the presence or absence of subclinical impairment of cognitive functions in pediatric BD patients. We did not find any difference in visual processing between patients and controls. But in auditory processing, latency of P300 in Behçet patients was longer than controls. We think that this finding may be due to subtle inflammation or vasculitis in different cerebral regions of pediatric BD patients (Table 1).

**Conclusion:** As a conclusion further evidence is needed before stating that pediatric BD patients without neurological involvement might have cognitive impairment in early periods of their disease.

**Disclosure of Interest**

None Declared.

**Table 1 (Abstract P165).** P300 latencies and amplitudes obtained from patients and controls after visual and auditory stimulations

Visual P300	Behçet	Control	p	Auditory P300	Behçet	Control	p
Amplitude (mV)	13.134	12.268	0.218	Amplitude (mV)	9.467	9.681	0.695
Latency (ms)	394.854	406.152	0.03	Latency (ms)	435.868	412.211	0.0001

### P166 Measurement of the carotid intima media thickness (CIMT) of carotid arteries in pediatric Behçet’s disease

#### Zehra S. Arici^1^, Berna Oğuz^2^, Ezgi D. Batu^1^, Hafize E. Sönmez^1^, Selcan Demir^1^, Ali Düzova^3^, Yelda Bilginer^1^, Seza Özen^1^

##### ^1^Pediatric Rheumatology, Hacettepe University, Ankara, Turkey; ^2^Pediatric Radyology, Hacettepe University, Ankara, Turkey; ^3^Pediatric Nephrology, Hacettepe University, Ankara, Turkey

###### **Correspondence:** Zehra S. Arici

**Introduction:** Cardiovascular disease is significantly higher in patients with chronic inflammatory diseases, such as rheumatoid arthritis (RA) or systemic lupus erythematosus (SLE). Behçet’s disease (BD) is also associated with acute and chronic inflammatory processes, which may cause increases in arterial stiffness and vascular damage. However, until now, little research has been reported on cardiovascular disease in association with BD.

**Objectives:** The assessments of coronary artery disease and generalized atherosclerosis, as well as the detection of carotid artery intima-media thickness (cIMT) by using high-resolution B-mode ultrasound, are good indicators of the increased risk of subclinical atherosclerosis.

**Methods:** This study included 21 patients with BD. In all patients, the cIMT were examined.cIMT standard deviation scores (cIMT-SDS) for age and height were calculated for all patients.

**Results:** The clinical characteristics of the 21 patients: male/female 13/8; median age years 17 (10-20.6); mean disease duration 57.7 (12-168) months. None of our patients had traditional cardiovascular risk factors. Median of our patients’ activity score is 0 (0-3). The median cIMT-SDS was found as 0.009 (-0.01-0.06).

**Conclusion:** We think suggest that the normal cIMT values in our patients may be related to the absence of other cardiovascular risk factors, the short duration of illness according to adult patients the regular follow-up and disease control with appropriate immunosuppressive treatment, and limited number of patients. Studies with larger number of pediatric patients are required.

**Disclosure of Interest**

None Declared.

### P167 Difficulties in diagnostics systemic children’s vasculitides

#### Olena A. Oshlyanska, Agar G. Artsymovych

##### Department of connective tissue disorders in children, State Institute of Pediatrics, Obstetrics and Gynecology, Academy of Medical Sciences of Ukraine, Kyiv, Ukraine

###### **Correspondence:** Agar G. Artsymovych

**Introduction:** Despite the specification of the criteria for the diagnosis of children’s systemic vasculitides (SV) (2006), their diagnostic is still difficult.

**Objectives:** To identify the main difficulties in the diagnosis of vasculitis.

**Methods:** A retrospective analysis of the course of SV in 8 patients according to the clinical register of connective tissue disease in children (2 PAN, 1 Takayasu syndrome, 1 Wegener's granulomatosis, 1 Churg-Strauss, the rest remained unspecified).

**Results:** The clinical picture in children with systemic vasculitis was various: hyperthermia - 6 cases, weight loss 6, dorsalgia 2, skin manifestations 7 (3 hemorrhagic rash, 2 spotted hemorrhagic, 2 dry gangrene, 1 hyperemia along the vessels, 1 liverdo, 1 child with syndrome Takayasu without skin changes), 3 migrating edema, 3 nodules, 5 myalgias, 4 hyperesthesia, 6 transient arthritis, 1 pericardial effusion, 4 heart rhythm disorders, 7 hepatolienal syndrome, 1 mucosal aphthae, 4 pulmonary lesions, 1 nosebleeds, 2 menorrhagia, 4 abdominalgia, 2 Gastrointestinal bleeding, 2 arterial hypertension, 1 asymmetry of blood pressure, pulse attenuation, vascular noises, 1 stroke, 5 urinary syndrome (3 proteinuria, 1 macro, 4 hematuria, 1 macro of them), 3 patients had polyneuropathy, 2 secondary cataracts, 2 destruction of the nasal septum, 1 – of sinus of the nose. The time of unfolding of the symptom complex before the diagnosis of SV was 7.1 ± 3.5 months (2-12).

Laboratory changes: leukocytosis 7 cases (20,8 ± 4,3G/l), anemia - 6 (in 1 case Hv 46 g/l), ESR increased in all cases 44.6 ± 13.2 mm/h, CRP 21, 2 ± 6.1 mg/l, hypereosinophilia was noted in 1 patient, hypertransaminase in 2, increased creatinine level in 2, hypercoagulation in 4, hemoculture, procalcitonin, complementation - all was normal, APLAB in 1 case revealed lupus anticoagulant, ANA, aDNA, aNRP in all patients were negative, antibodies to MP3 were positive in 2 cases (in 5 negative, in 1 there were no data), in the same patients there was RF +, markers of viral hepatitis were not detected s in any patient.

To the diagnostic criteria of Takayasu’s syndrome (4 criteria) corresponded 1 patient, the diagnosis was confirmed by aortography, 1 - the Churg-Strauss syndrome (4 criteria), 3 criteria had 2 patients (diagnosed with PAN). Of the patients, long-term observed as undifferentiated systemic vasculitis, 1 had 3 criteria of congruity with the diagnosis of MPA, in the 2nd 3 GPA or 5 MPA, 3 of 2 GPA or 5 MPA, in the 4th - 2 criteria of PAN. Morphologically confirmed 2 cases. Certain changes did not correspond to laboratory data: destruction of the nasal septum was detected in cases of seronegative for C-ANCA, and in 1 case the clinical symptom complex complied with the PAN criteria. In seropositive patients with C-ANCA, the common clinical features were arthritis, nephritis, menorrhagia and destructive pulmonary disorders.

All patients received GC, 6 cyclophosphamide, 1 mycophenolate mofetil, 1 azathioprine, 6 antimalarial, 2 plasmapheresis. In 4 cases, there is remission, 3 recurrences of vasculitis, 1 death.

**Conclusion:** The main problems in timely diagnosis of SV are insufficient awareness of general practitioners about nosologies data, the multidimensional unfolding of the clinical symptom complex, the frequent absence of pathognomonic autoantibodies, and the difficulty in conducting morphological studies.

**Disclosure of Interest**

None Declared.

### P168 Kawasaki disease and giant aneurysm in mexican children: evolution and clinical characteristics: a 5-year experience

#### Maria T. Brana^1^, Talia Diaz^1^, Yuridiana Ramirez^1^, Sofia Osorio^1^, Luis Aparicio^2^, Andres Rodriguez^2^, Enrique Faugier^2^, Rocio Maldonado^2^

##### ^1^Pediatric Rheumatology, Hospital Infantil de Mexico Federico Gomez, Mexico, Mexico; ^2^Hospital Infantil de Mexico Federico Gomez, Mexico, Mexico

###### **Correspondence:** Maria T. Brana

**Introduction:** Kawasaki disease (KD) is an acute, self-limited, systemic vasculitis, predominantly involving medium-sized arteries. It mainly affects children younger than five years and it is the leading cause of acquired heart disease in children in developed countries. Of unknown pathogenesis, KD severe complication is the occurrence of coronary artery lesions. Without early treatment, there is a 15 to 25% incidence of coronary artery lesions. Management with intravenous immunoglobin (IVIG), combined with aspirin, effectively decrease the incidence of this lesions to a 4%. The long-term prognosis is determined by the initial and current level of coronary artery involvement. Methods to predict which children are at higher risk for coronary aneurysms have been sought to determine prognosis and select patients for more rigorous treatment and follow-up.

**Objectives:** To describe the clinical presentation and evolution in addition to laboratory findings in Mexican pediatric population who developed giant aneurysms diagnosed with KD during the past 5 years. By identifying mayor risk factors in our population, an effective score could be used to select children for evaluation of additional therapies to prevent coronary artery aneurysms that occur despite treatment with IVIG.

**Methods:** Retrospective cohort study of the Children’s Hospital of Mexico Federico Gomez, last 5 years. We reviewed the data form the clinical archives of the patients who developed giant aneurysms after the diagnosis of KD from 2011 to 2016. A total of 84 patients with KD, 7% developed giant aneurysms. The variables analyzed, apart from the typical clinical and laboratory findings of KD, include size and Z score of the aneurysms, involution through follow up, cardiac morbidity and mortality, and treatment strategy.

**Results:** The mean age of patients at diagnostic was 17 months, and 84% were males. Only 33% of the patients developed complete KD, while 66% were diagnosed as incomplete. All patients presented with a positive Harada score. IVIG was administer in 83% of the patients, and a second dose was needed in 33%. Infliximab was used in 33% of the patients. One patient died due to cardiogenic shock. Results from echocardiography in the follow-up show that 33% of the patients have evolved to even larger aneurysms and 50% present no changes. Of the patients with a longer follow-up, 4 years after diagnostic, 33% have developed arrhythmias and 16% myocardial infraction. All are at high risk of sudden death.

**Conclusion:** The late diagnosis is the characteristic present in all patients which developed giant aneurysms, making imperative to identify clinical and laboratory findings that will help identify KD in Mexican pediatric population to avoid cardiac complications.

**Disclosure of Interest**

None Declared.

### P169 Scrotal involvement in children and adolescents Henoch Schonlein purpura

#### Izabel M. Buscatti, Henrique M. Abrão , Katia Kozu , Vitor V. Marques , Roberta Cunha , Adriana M. Sallum, Clovis A. Silva 

##### Pediatric rheumatology, University of São Paulo, São Paulo, Brazil

###### **Correspondence:** Izabel M. Buscatti

**Introduction:** Scrotal involvement in children and adolescents with Henoch-Schönlein purpura (HSP) is generally acute, resulting in swelling, pain and/or tenderness. Data of scrotal involvement in HSP patients are limited due to the small representation of this complication in previous case series, precluding an accurate analysis of associated factors and outcomes in patients with and without this complication, and particularly using the validated HSP criteria.

**Objectives:** To assess scrotal involvement and outcomes in children and adolescents with HSP.

**Methods:** Two hundred ninety six patients with HSP were retrospectively evaluated. All patients fulfilled validated European League Against Rheumatism/Paediatric Rheumatology International Trials Organisation/Paediatric Rheumatology European Society (EULAR/PRINTO/PRES) criteria for HSP patients. Of them, 150/296 (51%) were males and were assessed by demographic data, clinical manifestations, laboratory exams and treatments. Scrotal involvement was defined by the presence of scrotal edema and pain/tenderness in physical examination and/or testicular Doppler ultrasound abnormalities. Patients with only pain, petechiae or purpuric rash on scrotum were excluded. They were also divided in two groups at study entry: with and without acute scrotal involvement.

**Results:** Acute scrotal involvement was observed in 28/150 (18%) HSP patients. Six patients (21%) had bilateral involvement. None of patients had acute scrotum as the first sign/symptom of HSP. This complication was evidenced at HSP diagnosis in 27/28 (96%) and only one of them was diagnosed at 1 year of disease duration. Recurrent scrotal involvement was identified in 2/28 (7%) HSP patients. Thirteen patients underwent testicular Doppler ultrasound and revealed acute epididymitis-orchitis in all. A short course of prednisolone/prednisone (0.5-2.0 mg/kgday) was administered in 26/28 (93%), with resolution of scrotal signs/symptoms. Further analysis of HSP patients with acute scrotal involvement (n = 27) compared to those without this complication (n = 122) at diagnosis revealed that the median of current age [4.9 (1.7-12.6) vs. 6.1 (0.9-14.8) years, p = 0.201] and purpura duration [18.5 (5-60) vs. 14 (1120) days, p = 0.101] were similar in both groups. The frequency of elevated serum IgA was significantly lower in the former group (18% vs. 57%, p = 0.017), whereas corticosteroid (93% vs. 49%, p < 0.0001) and ranitidine use (63% vs. 30%, p = 0.003) were significantly higher in the former group. The frequencies of persistent purpura, arthritis, abdominal pain and nephritis were also similar in patients with and without acute scrotal involvement (p > 0.05).

**Conclusion:** Acute scrotal involvement was observed in almost one fifth of males with HSP. This complication was commonly evidenced at HSP diagnosis, and the recurrence was rarely seen. Scrotal signs/symptoms improved after a prompt use of corticosteroid.

**Disclosure of Interest**

None Declared.

### P170 Renal involment in henoch-schonlein purpura; possible correlation of positive antiphospholipid antibodies

#### Mehrnoush Hassas Yeganeh

##### Paediatric Rheumatology, Childran Hospital, Tehran, Iran, Islamic Republic of

**Introduction:** Henoch-Schönlein purpura (HSP) is the most common vasculitis affecting children. Renal impairment is one of severe complications among children with renal involvement that underline its early diagnosis and treatment. Antiphospholipid syndrome (APS) is an autoimmune multisystem disorder that has been shown to be associated with HPS. The association between antiphospholipid antibodies (aPL) and HSP renal involvement has not been investigated previously.

**Objectives:** A large body of evidence, investigated the association between aPL and renal involvement implied a wide spectrum of pathological manifestations such as renal artery stenosis, renal infarction, renal vein thrombosis and thrombotic microangiopathy. Interrelation between HPS and aPL has been reported as a complicated HSP or has been shown to be associated with central nervous system involvement in HPS cases.

Although complication of HSP with aPL has been documented previously, the impact of aPL in the HPS renal manifestations and prognosis has been remained elusive. Here, we followed 48 children with documented HPS up to 6 months and evaluated serum aPL and renal involvement in these cases. Also, the correlation of renal involvement with positive aPL investigated with the pattern of urinalysis in the mentioned patients.

**Methods:** Here, we studied 48 patients with HSP and investigated the association of renal involvement with aPL. Lupus anticoagulant, anticardiolipin and anti-b2 glycoprotein I antibodies were detected, along with six months follow-up to ascertain renal involvement.

**Results:** 14 (29.16%) of 48 patients demonstrated renal involvement that all renal complications appeared within 6 months during the course of HPS. Total positive aPL without renal involvement were 17.64% (6/34). The corresponding with renal involvement were 64.28% (9/14) that marks significant higher ratio.

**Conclusion:** Our data suggested that there seemed to be a cause-effect association between renal disease in HSP and positive aPL, which underscore its importance in early diagnosis of probable renal impairment.

**Disclosure of Interest**

None Declared.

### P171 “Eczema” with eosinophilia and raised ige: is it eosinophilic granulomatosis and polyangiitis (EGPA)?

#### Assunta Chi Hang Ho, Chung Mo Chow 

##### Paediatrics, Prince of Wales Hospital, Chinese University of Hong Kong, Hong Kong, Hong Kong

###### **Correspondence:** Assunta Chi Hang Ho

**Introduction:** EGPA is rare in children^1^. Patients usually have protracted asthma prodromal phase before onset of vasculitic manifestation. However in children without typical pulmonary symptoms, recognizing the condition may be challenging.

**Objectives:** Here we describe a case, which was previously misdiagnosed as eczema for 2 years.

**Results:** A 6 year-old Chinese girl with history of mild allergic rhinitis developed on and off erythematous itchy skin rash since 2014. The rashes were both nodular and patches and occurred symmetrically at dorsum of feet, knees, buttocks & elbows. It came as a crop and would regress in about 2 weeks, leaving hyperpigmentation (fig 1). There was raised IgE (1983kIU/l, <52). She was diagnosed as eczema and was given topical steroid cream.

From 2015 the skin eruption became more intense and frequent. She also developed recurrent arthritis of elbows, knees and ankles. She was only referred to rheumatology by 10/2016. Joint tapping showed inflammation. Urine dipstick indicated the presence of RBC but no proteinuria. Renal function was normal. Skin biopsy revealed presence of nuclear dust and fibrin deposition in subepidermal, perivascular and interstitial tissues, compatible with Leucocytoclasia.

From 11/2016 she developed moderate eosinophilia up to 2.9 x 10^9^/L (26% of circulating wcc). There was also recurrent soft tissue swelling over upper limbs, which appeared as inflammation in muscles and adjacent subcutaneous tissues in MRI. Biopsy showed eosinophil rich inflammation. There was no other cause to explain the eosinophilia.

Autoantibodies profile showed strongly positive ANA, anti MPO as well as anti scl-70 antibodies. Lung function showed no impairment.

Taking together her clinical features of mild allergic rhinitis, leucocytoclasia, high IgE, eosinophilia and extravascular infiltration of eosinophil and positive anti MPO, she was diagnosed EGPA. She was put on systemic prednisolone and azathioprine with good response.

**Conclusion:** In our case the rash and raised IgE led to the initial assumption of eczema. The diagnosis was only suspected when she developed systemic vasculitic features and eosinophilia. EGPA is extremely rare in children and there is no validated diagnostic criteria. While major classification criteria for EGPA require presence of asthma^2^, it may not be applicable in children due to the young age.

References

1) Zwerina J et al. Churg-Strauss Syndrome in childhood: a systemic literature review and clinical comparison with adult patients. Semin Arthritis Rheum. 2008;39:108-115.

2) Greco A et al. Churg-Strauss syndrome. Autoimmunity reviews. 2015;14:341-348.

**Disclosure of Interest**

None Declared.

### P172 Childhood Takayasu arteritis with drug resistant tuberculous lymphadenitis

#### Mahesh Janarthanan^1^, Padmasani Venkatramanan^2^, Vidya Krishna^2^, TS Arunprasath^2^, B Rajesh^2^

##### ^1^Division of Pediatric Rheumatology, Sri Ramachandra University, Chennai, India; ^2^Department of Pediatrics, Sri Ramachandra University, Chennai, India

###### **Correspondence:** Mahesh Janarthanan

**Introduction:** Takayasu arteritis (TA) is an idiopathic large vessel vasculitis. The clinical features can be varying and patients may present in the acute or chronic phase with nonspecific clinical features. Tuberculosis is widely prevalent in tropical countries and has been implicated in the pathogenesis of Takayasu arteritis.

**Objectives:** There are many reports regarding coexisting Takayasu arteritis and active tuberculosis but none regarding Takayasu arteritis and drug resistant tuberculosis in children.

**Methods:** We present the case of a child who presented with nonspecific features but on investigation proved to have Takayasu arteritis associated with drug resistant TB lymphadenitis.

**Results:** A 17 year old girl presented with swelling left supraclavicular region of neck, low grade fever, loss of appetite and loss of weight for 4 months. A week prior to admission to the hospital she had developed swelling of both the legs. The patient’s father had been treated for pulmonary tuberculosis 2 years ago.

On examination she had mild pallor, was undernourished with mild bilateral pitting pedal edema. She had multiple cervical and supraclavicular node 3x2cms which were firm, non-tender and matted.She had asymmetric pulses and discrepancy in 4 limb blood pressure.

Laboratory investigations revealed anemia and elevated ESR (71 mm/hour). Peripheral smear and iron studies were suggestive of iron deficiency. Mantoux test was positive (10mm). Echocardiogram showed left subclavian stenosis, dilated ascending aorta and normal abdominal aorta. Aortogram revealed aortic arch vessel involvement with thoracic aortic narrowing at subclavian artery and second narrowing at the level of diaphragm.

Histopathology report of left cervical lymphnode biopsy showed necrotizing granulomatous lymphadenitis consistent with tuberculosis. Gene Expert detected mycobacterium tuberculosis resistant to rifampicin. The diagnosis of Takayasu with drug resistant tuberculous lymphadenitis was established.

On the advice of infectious diseases team she was commenced on Levofloxacin, Ethionamide, Pyrazinamide, Ethambutol and Inj Kanamycin for 8 months. This was followed by Levofloxacin Ethionamide and Ethambutol for further 12 months. For Takayasu arteritis she received steroids initially and then 6 cycles of monthly cyclophosphamide subsequently. This was followed by weekly oral methotrexate. The patient is well 4 years since presentation and is currently on methotrexate, aspirin and antihypertensives.

**Conclusion:** Co existing drug resistant Tuberculosis is possible in Takayasu arteritis particularly in tropical countries where TB is prevalent. Treatment with combination of anti-tuberculous and disease modifying agents with careful monitoring of therapy is the key to the management of such patients.

**Disclosure of Interest**

None Declared.

## Treatment of autoinflammatory diseases

### P173 Evaluation of efficacy and safety of opocalcium colchicine, anakinra and canakinumab in colchicine-resistant childhood familial Mediterranean fever

#### Kenan Barut, Amra Adrovic, Sezgin Sahin, Asli Kaplan, Ozgur Kasapcopur

##### Pediatric Rheumatology, Istanbul University, Cerrahpasa Medical School, Istanbul, Turkey

###### **Correspondence:** Kenan Barut

**Introduction:** Familial Mediterranean fever (FMF) is the most common autoinflammatory condition characterized by recurrent attacks of fever and serositis. Colchicine is the main therapeutic option for the prevention of FMF attacks. Poor compliance to colchicine can lead to life threating complications, such as amyloidosis. In addition to the recurrent disease attacks despite regular usage of colchicine in appropriate and efficacious dosage, continuous subclinical inflammation between attacks is also a treatment challenge. The colchicine-resistant FMF (crFMF) is defined as 6 or more serositis attacks in the last year or 4-6 attacks during the last 3 months despite the regular usage of colchicine in the highest tolerable dose (maximum 2 mg/kg for children). IL-1 receptor antagonists (anakinra, canakinumab) have been shown to be efficient in crFMF.

**Objectives:** Initially we aimed to determine the crFMF patients follow up at our department. Furthermore, we tried to define the efficacy of opocalcium colchicine (OC), anakinra and Canakinumab by comparing the FMF50 scores, the complete and partial clinical responses among patients with different treatment modalities.

**Methods:** We assessed FMF patients followed up at our department. The data of patients are taken from the patients’ database. Patients who were under OC, anakinra and canakinumab are considered to be resistant to standard colchicine treatment. The FMF50 score is used to define the response to treatment. The FMF50 is defined as at least 50% improvement in five of six criteria, without worsening in any of them. Complete clinic and laboratory response is characterized by an absence of clinical features and normal laboratory findings. The partial clinical and laboratory response include 30% improvement in clinical and laboratory features. FMF50 score of crFMF patients under colchicine or other treatment modalities for at least 6 months has been determined retrospectively by using data from the patients’ records. Complete or partial clinical remission has been evaluated for all crFMF patients.

**Results:** A total of 839 FMF patients has been assessed and 49/839 (5,8%) of them has been considered resistant. FMF50 response has been obtained in 4/49 (8.2%) patients under standard colchicine treatment; in 14/30 (46.7%) patients treated with OC, in 5/6 (83.3%) with anakinra and in 12/13 (92.3%) patients with canakinumab. The FMF50 response significantly differed according to treatment modality (p < 0.005). Clinical remission has been achieved in 10/30 (33.3%), 5/6 (83.3%) and in 11/13 (84.6%) patients treated with OC, anakinra and canakinumab, respectively. Patients treated with OC significantly differed from those treated with anti IL-1 according to complete clinical remission (p < 0.002). Laboratory remission has been obtained in 7/30 (23.3%), 4/6 (66.7%) and 11/13 (84.6%) patients treated with OC, anakinra and canakinumab, respectively. The laboratory remission was significantly different between patients treated with OC and with anti IL-1 (p < 0.0001).The most common adverse effect was diarrhea in 17/49 (34.6%), transaminases elevation in 3/49 (6%) patients and leukopenia only in 1 patient. Diarrhea was seen in 3/30 (10%) patients under OC treatment. Local allergic reactions were seen in 3/6 (50%) anakinra patients. One patient developed pneumonia while on canakinumab treatment; upper respiratory tract infection has been registered in 3/13 (23%) patients and acute gastroenteritis in one patient. All of them responded to antibiotics treatment. None of the patients developed severe adverse effect.

**Conclusion:** The FMF50 response has not been achieved in majority of patients under OC treatment, although their drug compliance was better comparing to standard colchicum-dispert. Complete clinical remission has been obtained in minority of patients treated with OC. In contrary, FMF50 response, complete clinical and laboratory response have been detected in most of patients treated with anakinra and canakinumab. Both of anti IL-1 agents were safe and effective in crFMF patients.

**References**

1. Ozdogan H, Ugurlu S. Canakinumab for the treatment of familial Mediterranean fever. Expert review of clinical immunology. 2017;13(5):393-404.

2. Ozen S, Demirkaya E, et al. FMF50: a score for assessing outcome in familial Mediterranean fever. Annals of the rheumatic diseases. 2014;73(5):897-901.

**Disclosure of Interest**

None Declared.

### P174 Proton pomp inhibitors treament improve the disease in a novel knock-in caps model developing systemic amyloidosis

#### Arinna Bertoni^1^, Sonia Carta^2^, Enrica Balza^2^, Federica Penco^1^, Silvia Borghini^3^, Michele Fiore ^3^, Chiara Baldovini ^4^, Paolo Nozza^4^, Emanuela Ognio^5^, Francesca Schena^1^, Patrizia Castellani^2^, Marco DiDuca^3^, Claudia Pastorino^1^, Maria Libera Trotta^6^, Isabella Ceccherini ^3^, Alberto Martini^1^, Anna Rubartelli^2^, Marco Gattorno^1^, Sabrina Chiesa^1^

##### ^1^UOC Pediatria II, Laboratorio di Immunologia delle Malattie Reumatiche, Istituto Giannina Gaslini, Genova, Italy; ^2^Unità di Biologia Cellulare, IRCCS San Martino-Ist, Genova, Italy; ^3^Genetica Medica, Istituto Giannina Gaslini, Genova, Italy; ^4^UOC Anatomia Patologica, Istituto Giannina Gaslini, Genova, Italy; ^5^UOS Animal Facility, IRCCS San Martino-Ist; ^6^Università degli Studi di Genova, Genova, Italy

###### **Correspondence:** Arinna Bertoni

**Introduction:** CAPS are a group of rare autoinflammatory diseases associated to NLRP3 mutations, leading to inflammasome hyperactivity and IL-1β hypersecretion. CINCA syndrome is the most severe CAPS disease characterized by central nervous system disabilities with a long-term risk of secondary amyloidosis. Amyloid deposits in AA amyloidosis are composed mainly of the serum amyloid A (SAA) protein.

Recently, we have developed a novel NLRP3 Knock In (KI) mouse model for CAPS that replicate many of the clinical and pathologic features seen in humans.

NLRP3 is a main target of proton pomp inhibitors (PPIs), commonly used for the treatment of excessive acid secretion. Omeprazole (OME), a well-known PPIs, has a potential anti-inflammatory effect making him a promising drug against sepsis and other severe inflammatory conditions.

**Objectives:** To identify alternative and repurposing drugs for the treatment of CAPS diseases.

**Methods:** Cytokines secretion from bone marrow derived dendritic cells (BMDCs) and peritoneal macrophages (PMs) was evaluated by ELISA.

We have conducted a preclinical animal study to evaluate the response of KI to OME.

Hystological analysis of all organs was evaluated by hematoxylin and eosin staining.

Amyloid deposition was quantified through Congo Red staining.

**Results:** We engineered N475K mutation in the murine NLRP3 gene to obtain heterozygous KI that recapitulates CAPS syndromes. Mice developed systemic inflammation with consequent delayed growth, splenomegaly and hepatomegaly when compared to wild type (WT) controls. Hystological analysis revealed the presence of an intense acute and chronic inflammatory cell infiltrates and amyloid deposits in spleen, liver, kidneys and lungs.

As in CAPS patients, KI mice showed an increase in IL-1β, IL-1α and IL-18 secretion ad a decrease level of IL1RA production, the physiological inhibitor of preformed IL-1b.

OME treatment increased the lifetime of KI mice, reduced the *in vitro* secretion of IL-1β by BMDCs and PMs isolated from KI and restored the production of IL1RA. Remarkably, PPIs improved inflammatory condition of the organs *in vivo* and since the first month were able to limit the amyloid deposition in the organs of KI mice.

Serum analyzes are ongoing to quantify Serum Amyloid A (SAA) protein levels.

**Conclusion:** PPIs are promising new drugs, economical, non toxic, available in all countries. Down-modulation of IL-1b secretion by PPIs suggest the potential use of these drugs also for the treatment of autoinflammatory disorders. Although further studies are needed, these drugs seem encouraging also to improve a severe CAPS complication, amyloidosis.

**Disclosure of Interest**

None Declared.

### P175 Canakinumab dosing in patients with still’s disease: exposure-response analysis of pooled SJIA data by age groups

#### Eugen Feist^1^, Pierre Quartier^2^, Bruno Fautrel^3^, Rayfel Schneider^4^, Paolo Sfriso^5^, Petros Efthimiou^6^, Luca Cantarini^7^, Karine Lheritier^8^, Karolynn Leon^9^, Chetan S. Karyekar^9^, Antonio Speziale^8^

##### ^1^Charite-Universitätsmedizin, Berlin, Germany; ^2^Necker-Enfants Malades Hospital, Paris, France; ^3^Pitie Salpetriere Hospital, Paris, France; ^4^University of Toronto and The Hospital for Sick Children, Toronto, Canada; ^5^University of Padova, Padova, Italy; ^6^Weill Cornell Medical College, New York, NY, USA; ^7^University of Siena, Siena, Italy; ^8^Novartis Pharma AG, Basel, Switzerland; ^9^Novartis Pharmaceuticals Corporation, East Hanover, NJ, USA

###### **Correspondence:** Eugen Feist

**Introduction:** Still’s disease presents as a disease continuum in paediatric and adult patients (pts), denoted as systemic juvenile idiopathic arthritis (SJIA) and adult-onset Still’s disease (AOSD), respectively.^1-3^ Both SJIA and AOSD have cytokine-driven pathophysiology wherein interleukin-1 (IL-1) plays a key role.^1^ Canakinumab, a selective human anti-IL1 β monoclonal antibody, is shown to be efficacious in treating active SJIA pts.^4^

**Objectives:** To evaluate the adequacy of canakinumab dosing in pts in the AOSD age range through an exposure-response (E-R) analysis of pooled canakinumab data from SJIA pts across three age groups (grps) of children, young adolescents, and older adolescents and young adults (the latter representing AOSD population).

**Methods:** In this pooled subgroup analysis, pharmacokinetics (PK) and efficacy data of canakinumab treated pts were included from 4 SJIA studies (NCT00426218, NCT00886769, NCT00889863, NCT00891046). Study designs for trials have been published.^4-5^ Canakinumab was administered at 4 mg/kg every 4 weeks (wks) in 3 studies and 0.5-9.0 mg/kg in 1 study. Population PK and E-R modelling was performed with dose, age and weight as covariates. PK concentrations in different age grps were predicted using the PK-binding model. Steady-state exposure metrics including trough concentration (C_MINss_), peak concentration (C_MAXss_) and area under the plasma concentration-time curve (AUC_ss_) were derived. Of 4 SJIA studies, one study [NCT00426218] where 4 mg/kg every 4 wks was not given, was excluded for efficacy outcomes. Efficacy endpoint was adapted American College of Rheumatology (aACR) paediatric responses over 12 wks of treatment in the 3 age grps. Safety and tolerability were also assessed by age grps.

**Results:** PK analysis was done in 233 children (0-12 years [yrs] of age), 60 young adolescents (12-16 yrs) and 31 older adolescents and young adults (16-20 yrs; age at the time of enrolment in the first study). Steady-state canakinumab exposure showed positive trends in median exposure with increasing pts age and were comparable across age grps (Table 2). A total of 216 children (2 to <12 yrs of age), 56 young adolescents (12 to <16 yrs) and 29 older adolescents and young adults (≥16 yrs) were analysed for efficacy outcomes. aACR response was comparable across the 3 age grps (Table 2). The safety profile of canakinumab was similar across different age grps with infections and infestations being the most common adverse event.

N, total number of patients in each age group; n, number of patients assessed for ACR variables; Q1, 1^st^ quartile (25^th^ percentile); Q3, 3^rd^ quartile (75^th^ percentile)

**Conclusion:** A comparable canakinumab exposure-response relationship was observed across all 3 age groups. Canakinumab showed a sustained therapeutic effect and no meaningful differences in safety profiles were noted across all age grps. These analyses suggest that a canakinumab body-weight adjusted regimen at the dose of 4 mg/kg every 4 weeks offers similar safety and efficacy in AOSD pts to that observed in SJIA pts.

1. Jamilloux et al. *Immunol Res* 2015;61:53-62. 2. Nirmala et al. *Pediatric Rheumatol* 2015;13:50. 3. Jamilloux, et al. *Ther Clin Risk Manag* 2015;11:33-43. 4. Ruperto et al. *N Engl J Med*. 2012;367(25):2396-406. 5. Ruperto et al. *Arthritis Rheum* 2012;64(2):557-67.

**Trial registration identifying number:** NCT00426218, NCT00886769, NCT00889863, NCT00891046

**Disclosure of Interest**

E. Feist Grant/Research Support from: Novartis, Consultant for: Novartis, P. Quartier Grant/Research Support from: Abbvie, Novartis, Pfizer, Chugai-Roche, Consultant for: Abbvie, Novartis, Pfizer, Sobi, Roche, Novimmune and Sanofi, Speaker Bureau of: Abbvie, Novartis, Sobi, Roche, B. Fautrel Grant/Research Support from: Abbvie, MSD, Pfizer, Consultant for: Abbvie, Biogen, BMS, Celgene, Hospira, Janssen, Lilly, MSD, Nordic Pharma, Pfizer, Roche, Sobi, UCB, R. Schneider Consultant for: Novartis, Novimmune and Sobi, P. Sfriso Consultant for: Novartis, P. Efthimiou Consultant for: Novartis, L. Cantarini Consultant for: Sobi, Novartis, and AbbVie, K. Lheritier Employee of: Novartis, K. Leon Employee of: Novartis, C. S. Karyekar Employee of: Novartis, A. Speziale Employee of: Novartis.

**Table 2 (Abstract P175).** Simulated steady-state exposure metrics of canakinumab and aACR paediatric response for SJIA patients by age group

Steady-state exposure metrics, Median (Q1–Q3)	Responses (%)	Children	Young adolescents	Older adolescents and young adults
C_MINss_	-	13.59 (8.91–19.7)	16.17 (11.21–22.15)	20.11 (14.5–25.92)
C_MAXss_	-	37.35 (29.07–45.93)	38.06 (28.68–48.44)	41.63 (28.85–51.83)
AUC_ss_	-	685.84 (511.88–910.71)	726.44 (572.15–976.65)	916.29 (610.3–1080.89)
**aACR paediatric response, Day 15**, n/N (%)	**≥30**	158/216 (73.1)	47/56 (83.9)	25/29 (86.2)
	**≥70**	109/216 (50.5)	33/56 (58.9)	19/29 (65.5)
	**≥100**	46/216 (21.3)	15/56 (26.8)	4/29 (13.8)
**aACR paediatric response, Day 85**, n/N (%)	**≥30**	90/133 (67.7)	20/27 (74.1)	15/18 (83.3)
	**≥70**	77/133 (57.9)	18/27 (66.7)	13/18 (72.2)
	**≥100**	42/133 (31.6)	8/27 (29.6)	4/18 (22.2)

### P176 Improvement of disease activity in patients with colchicine-resistant FMF, HIDS/MKD and traps assessed by autoinflammatory disease activity index (AIDAI): results from the cluster trial

#### Isabelle Kone-Paut^1^, Michaël Hofer^2^, Susanne Benseler^3^, Jasmin Kuemmerle-Deschner^4^, Annette Jansson^5^, Itzhak Rosner^6^, Alberto Tommasini^7^, Sara Murias^8^, Omer Karadag^9^, Jeremy Levy^10^, Chetan S. Karyekar^11^, Fabrizio De Benedetti^12^

##### ^1^APHP, CHU de Bicêtre, Univ Paris Sud, Le Kremlin Bicêtre, France; ^2^University of Lausanne, Lausanne, Switzerland; ^3^Alberta Children’s Hospital, Calgary, Canada; ^4^University Hospital Tuebingen, Tuebingen, Germany; ^5^Ludwig Maximilian University, Munich, Germany; ^6^Bnai-Zion Medical Center, Rheumatology, Haifa, Israel; ^7^Institute for Maternal and Child Health IRCCS Burlo Garofolo, Trieste, Italy; ^8^Hospital La Paz, Madrid, Spain; ^9^Hacettepe University Faculty of Medicine, Ankara, Turkey; ^10^BIOP, Reinach, Switzerland; ^11^Novartis Pharmaceuticals Corporation, East Hanover, NJ, USA; ^12^IRCCS Ospedale Bambino Gesú, Rome, Italy

###### **Correspondence:** Isabelle Kone-Paut

**Introduction:** AIDAI is being increasingly recognised as a novel, validated tool for the assessment of disease activity across a wide spectrum of autoinflammatory diseases including periodic fever syndromes (PFS) [familial Mediterranean fever (FMF), hyper-IgD syndrome/mevalonate kinase deficiency (HIDS/MKD) and TNF receptor-associated periodic syndrome (TRAPS)].^1^ Canakinumab (CAN), a fully human anti-interleukin-1β monoclonal antibody, has demonstrated efficacy in resolving active flares and preventing new flares in PFS patients (pts) through CLUSTER trial.^2^

**Objectives:** To calculate AIDAI scores over 16 weeks of CAN treatment in patients from CLUSTER study and assess correlation between AIDAI and disease/response characteristics.

**Methods:** CLUSTER study consisted of 3 cohorts (crFMF, HIDS/MKD and TRAPS), the design and results of which have been previously presented.^2^ AIDAI was calculated as the sum of 12 items (Yes = 1or No = 0)^1^ for 30 consecutive days. AIDAI score was calculated if the first score was recorded ≥29 days before baseline. Missing AIDAI assessments between first and last assessments were imputed with No. Missing items beyond last evaluable measurement were imputed by LOCF. Proportion of pts with inactive disease (ID; AIDAI score <9) was calculated at Wk 16. Correlation analysis of AIDAI with C-reactive protein (CRP), serum amyloid A (SAA), physician global assessment (PGA) and Sheehan disability score (SDS), and child health questionnaire–psychological/physical (CHQ–PsCS/PCS) and short form 12–physical/mental (SF12–PCS/MCS) component summaries were performed at baseline and Wk 16, with significance set at p < 0.05.

**Results:** Overall, 181 (crFMF, N = 63; HIDS/MKD, N = 72; TRAPS, N = 46) patients were randomised (1:1) to CAN 150 mg or placebo every 4 wks. Median AIDAI scores in all three cohorts decreased from baseline to Wk 16 (crFMF, 23 to 6; HIDS/MKD, 41 to13; TRAPS, 89 to 14). The proportion of pts with ID at Wk 16 was 52% in crFMF, 40% in HIDS/MKD and 46% in TRAPS cohorts. AIDAI at Wk 16 correlated significantly with: SDS in all 3 cohorts; PGA in HIDS/MKD and TRAPS; SF12–MCS in crFMF and HIDS/MKD (Table 3). CRP and SAA did not correlate with AIDAI.

**Conclusion:** Decrease in AIDAI scores over 16 weeks in crFMF, HIDS/MKD and TRAPS patients treated with canakinumab corroborates rapid and sustained disease control with canakinumab in the CLUSTER study. At Week 16, approximately half of the crFMF and TRAPS patients, and 40% of the HIDS/MKD patients had inactive disease. AIDAI improvements at Week 16 correlated with patient and physician driven evaluations (PGA, SF12–MCS and SDS).

^1^Piram M, et al. *Ann Rheum Dis*. 2014;73:2168-73.

^2^De Benedetti F, et al. *Ann Rheum Dis*. 2016;75:615-6.

**Trial registration identifying number:** NCT02059291

**Disclosure of Interest**

I. Kone-Paut Grant/Research Support from: Novartis, SOBI and Roche, Consultant for: Novartis, SOBI, Pfizer, AbbVie and Roche, M. Hofer Consultant for: Novartis and AbbVie, S. Benseler Consultant for: Novartis, SOBI and AbbVie, J. Kuemmerle-Deschner Grant/Research Support from: Novartis, Consultant for: Novartis, SOBI and Baxalta, A. Jansson: None Declared, I. Rosner: None Declared, A. Tommasini: None Declared, S. Murias: None Declared, O. Karadag: None Declared, J. Levy Consultant for: Novartis, C. S. Karyekar Employee of: Novartis, F. De Benedetti Grant/Research Support from: Pfizer, AbbVie, Roche, Novartis, Novimmune and BMS.

**Table 3 (Abstract P176).** Correlation between AIDAI and disease activity/response variables at Week 16

	Correlation coefficient (95% CI; *p < 0.0001; ^†^p < 0.001; ^§^p < 0.01; ^‡^p < 0.05)		
	crFMF N = 63	HIDS/MKD N = 72	TRAPS N = 46
CRP	-0.12 (-0.36; 0.14)	0.23 (-0.01; 0.45)	0.12 (-0.19; 0.42)
SAA	-0.01 (-0.27; 0.25)	-0.05 (-0.30; 0.21)	0.06 (-0.26; 0.37)
PGA	0.23 (-0.02; 0.46)	**0.35** ^**§**^ **(0.12; 0.55)**	0.73* (-0.54; 0.85)
CHQ–PsCS	-0.18 (-0.56; 0.26)	-0.25 (-0.55; 0.11)	-0.33 (-0.72; 0.22)
CHQ–PCS	-0.33 (-0.66; 0.11)	**-0.46** ^**§**^ **(-0.70; -0.14)**	-0.48 (-0.80; 0.04)
SF12–PCS	-0.26 (-0.57; 0.11)	-0.23 (-0.68; 0.35)	**-0.52** ^**‡**^ **(-0.81; -0.03)**
SF12–MCS	**-0.45** ^**‡**^ **(-0.70; -0.10)**	**-0.55** ^**‡**^ **(-0.84; -0.03)**	0.09 (-0.43; 0.56)
SDS	**0.47** ^**†**^ **(0.22; 0.67)**	**0.37** ^**§**^ **(0.10; 0.59)**	**0.41** ^**‡**^ **(0.06; 0.67)**

### P177 Long-term effectiveness of canakinumab in children with familial mediterranean fever

#### Nesrin Gülez , Balahan Makay, Betül Sözeri

##### Behçet Uz Children/s Hospital, Izmir, Turkey

###### **Correspondence:** Balahan Makay

**Introduction:** Although colchicine therapy has dramatically improved the prognosis of familial Mediterranean fever (FMF) by decreasing the frequency of attacks and preventing amyloidosis, 5– 15% of patients are reported to be unresponsive to colchicine. *Canakinumab* is an anti-IL-1β monoclonal antibody. Food and Drug Administration (FDA) recently aproved this drug for familial Mediterranean fever. Little is known about the long-term efficacy and safety of canakinumab in FMF.

**Objectives:** To present the single center experience of colchicine-resistant pediatric FMF patients who were treated with canakinumab by off-label use since 2012.

**Methods:** The hospital files of children with FMF who were started canakinumab were retrospectively evaluated. Fifteen patients (8 girls and 7 boys) were included in the study. Initial dose of canakinumab was 2 mg/kg/8 week for patients below 40 kg and 150 mg/8 week for patients above 40 kg.Dose and frequency were adjusted according to patients’response to treatment.All patients continued colchicine treatment during canakinumab use. Clinical and laboratory data of each visit were recorded. Drug-related adverse events were recorded. Complete remission was described as no attacks and normal acute phase reactants; partial remission was defined as decrease in severity and rates of attacks and/or elevated acute phase reactants with anti-IL-1 treatment.

**Results:** The mean age of the patients was 14.5 ± 3.8 years, and the mean follow-up time from diagnosis was 8.5 ± 3.7 years. The duration of canakinumab use was 22.5 ± 15 months (min:9 max:55 months). Twelve patients were M694V homozygotes. One patient had nephrotic range proteinuria and renal amyloidosis proved by biopsy. Eleven patients achieved complete remission after the first dose at 2 months and 12 patients at 6 months. Canakinumab interval was shortened in 2 patients from 150 mg/8 weeks to 150 mg/4 weeks. Except one, all patients achieved complete remission by 12 months. This patient had bilateral sacroiliitis and HLA B27 positivity and also been treated with methotrexate, sulphasalazine, and adalimumab in the past. Nephrotic range proteinuria of the other patient with amyloidosis disappeared and re-biopsy was normal at one year of canakinumab. Two patients had mild urinary tract infections. One patient had bronchopneumonia requiring hospitalization. Two patients had teeth abscess. There were no serious adverse events such as opportunistic infections, malignancies, or deaths. There were no injection site reactions. Besides, no significant laboratory abnormalities occurred in complete blood count parameters, liver and kidney function tests.

**Conclusion:** To the best of our knowledge, this is the longest outcome study about canakinumab use in pediatric FMF patients**.** The results of this study suggested that canakinumab treatment is safe and effective in FMF patients in the long-term.

**Disclosure of Interest**: None Declared.

### P178 Evaluation of a dosing regimen for tocilizumab in patients younger than two years of age with systemic juvenile idiopathic arthritis

**Abstract withdrawn**

### P179 Joint involvement predicts severe pediatric crohn’s disease evolution

#### Naim Ouldali^1, 2^, Jean-Pierre Hugot^3, 4^, Jérome Viala^3^, Mohamed Damir^2^, Christine Martinez-Vinson^3^, Ulrich Meinzer^1,4,5^

##### ^1^Pédiatrie générale, maladies infectieuses et médecine interne, Hôpital Robert Debré, Paris, France; ^2^Unité d’épidémiologie Clinique, Hôpital Robert Debré, Paris, France; ^3^Service de gastronetérologie pédiatrique, Hôpital Robert Debré, Paris, France; ^4^INSERM UMR1149, Universite Paris Diderot, Paris, France; ^5^Unité Biologie et génétique de la paroi bactérienne, Institut Pasteur, Paris, France

###### **Correspondence:** Ulrich Meinzer

**Introduction:** Crohn disease (CD) is a chronic relapsing inflammatory bowel disease with a significant impairment of quality of life. Disease onset occurs at pediatric age in up to 20% of the cases. The use of intensified treatment approaches for patients with severe disease has been proposed previously. These approaches rely on early identification of severe disease. Therefore it is essential to establish parameters that allow prediction of severe disease course. Some parameters, including disease location, age at diagnosis and disease behavior have been proposed but no data are available about joint involvement, to predict severe digestive disease.

**Objectives:** To sudy the link between joint involvement and severity of intestinal Crohn’s disease.

**Methods:** We studied the link between joint involvement (JI) and therapeutic failure in a retrospective and prospective cohort of 272 pediatric patients with confirmed CD according to international criteria, followed in a French tertiary center between 2005 and 2016.

**Results:** JI was observed in 65/272 (23.9%) patients and occurred in 75% of cases during the first year following CD diagnosis. We found a highly significant association between JI and therapeutic failure due to digestive disease (OR = 6.2, IC [2.7 to 14.5], *p* < 0.0001) and introduction of biotherapy (OR = 3.2, IC [1.8 to 6.0], *p* = 0.0001) two and four years after diagnosis.

**Conclusion:** This study suggests that in pediatric patients, occurrence of joint involvement may be used as a marker to identify patients with severe CD. From a clinical point of view it indicates that patients with arthropathies should be managed with particular attention, which may justify closer follow-up visits. As joint involvement occurred during the first year after diagnosis in the large majority of patients it may allow for selection of patients that may benefit from early aggressive treatment algorithms.

**Disclosure of Interest**

None Declared.

### P180 A diagnostic score for early detection of macrophage activation syndrome in systemic juvenile idiopathic arthritis based on an improper linear model method

#### Francesca Minoia^1^, Francesca Bovis^2^, Sergio Davì^1^, AnnaCarin Horne^3^, Michel Fischbach^4^, Michael Frosch ^5^, Adam Huber ^6^, Marija Jelusic-Drazic^7^, Sujata Sawhney^8^, Deborah McCurdy ^9^, Clovis A. Silva^10^, Donato Rigante^11^, Nicola Ruperto^1^, Alberto Martini^1^, Randy Q. Cron^12^, Angelo Ravelli^1,2^

##### ^1^Istituto G. Gaslini, Genoa, Italy; ^2^Università degli Studi di Genova, Genoa, Italy; ^3^Karolinska University Hospital, Stockholm, Sweden; ^4^Hopital Universitaire Hautepierre, Strasbourg, France; ^5^University Children’s Hospital, Munster, Germany; ^6^IWK Health Centre, Halifax, Canada; ^7^University Hospital Centre, Zagreb, Croatia; ^8^Sir Ganga Ram Hospital, New Delhi, India; ^9^Mattel Children’s Health Center UCLA, Los Angeles, CA, USA; ^10^Universidade de Sao Paulo, Sao Paulo, Brazil; ^11^Università Cattolica del Sacro Cuore, Rome, Italy; ^12^University of Alabama, Birmingham, AL, USA

###### **Correspondence:** Francesca Minoia

**Introduction:** Macrophage activation syndrome (MAS) is the most severe complication of systemic juvenile idiopatic arthritis (sJIA). Because MAS can follow a rapidly fatal course, its prompt recognition and immediate therapeutic intervention are critical. An international collaborative effort has recently led to the publication of the 2016 classification criteria for MAS in sJIA. However these criteria are intended to serve for classification purposes and not as diagnostic tool

**Objectives:** To develop and validate a diagnostic score for timely detection of MAS in patients with sJIA.

**Methods:** The clinical and laboratory features of 362 patients with sJIA-associated MAS and 404 patients with active sJIA without evidence of MAS were collected in a multinational collaborative project. 80% of the study population was used to develop the score and the remaining 20% as a validation sample. All features associated with the diagnosis of MAS in univariate analysis (p < 0.05) were further scrutinized in multivariable logistic regression. By means of an improper linear model method, the variables that entered the best fit model were assigned a score, based on their β-coefficient weights (score 1 for β-coefficients ≤ 1, score 2 for β-coefficients > 1, score 3 for β-coefficients ≥ 2). The best cut-off that discriminated MAS from active sJIA was calculated by means of receiver operating characteristic (ROC) curve analysis. The sensitivity (SE), specificity (SP), area under the curve (AUC) and kappa value of the score were calculated for both the developmental and validation samples

**Results:** The 12 variables that were most closely associated with a diagnosis of MAS in logistic regression analysis are presented in Table 4, together with their respective score. The final score ranged from 0 to 21. A cut-off value > 7 performed best in discriminating MAS from active sJIA (SE = 84%, SP = 84%, AUC = 0,91, kappa = 0,69). The good performance of the score was confirmed in the validation sample (SE = 92%, SP = 88%, AUC = 0,90, kappa = 0,80).

**Conclusion:** The new diagnostic score, provisionally named MS score, is a powerful and feasible tool for the early detection of MAS in patients with active sJIA. Notably, the use of an improper linear model method may allow the application of the score in different settings. The MS score deserve validation in a prospective cohort of patients with sJIA-associated MAS

**Disclosure of Interest**

None Declared.

**Table 4 (Abstract P180).** See text for description

Variables scored with 3 points	CNS involvement; Hemorrhagic manifestations
Variables scored with 2 points	Platelet count ≤ 340 x 10^9^/l ; Triglycerides ≥ 145 mg/dl; Ferritin ≥ 1550 ng/ml; Lactic dehydrogenase ≥ 640 U/l ; Absence of arthritis
Variables scored with 1 point	Albumin ≤ 3.3 g/dl; White blood cell count ≤ 14 x 10^9^/l; Aspartate transaminase ≥ 48 U/l; Hepatomegaly; Absence of skin rash

### P181 Predictors of incomplete response to tocilizumab therapy in children with systemic-onset juvenile idiopathic arthritis

#### Kenichi Nishimura, Seira Hattori, Asami Oohara, Tomo Nozawa, Ryoki Hara , Shuichi Ito

##### Department of Pediatrics, Yokohama City University, Yokohama, Japan

###### **Correspondence:** Kenichi Nishimura

**Introduction:** Glucocorticoids (GCs) are first-line therapy for patients with systemic-onset juvenile idiopathic arthritis (sJIA), but approximately half of patients develop steroid dependence or resistance. Tocilizumab (TCZ) is a promising option for such intractable patients, although some continue to require high-dose GCs to maintain remission or treat persistent arthritis even under TCZ therapy. The predictors of incomplete response to TCZ in patients with sJIA remain unknown.

**Objectives:** To investigate the predictors of incomplete response to TCZ and their clinical course in patients with sJIA.

**Methods:** This study was a single-center, retrospective observational study. Twenty-two patients with sJIA treated with TCZ were enrolled, and the observation period was more than 2 years. Good responder (GR) was defined as the achievement of remission with less than 0.2 mg/kg/day of prednisolone (PSL). Incomplete responder (IR) was defined as the inability to achieve remission and/or requirement for greater than or equal to 0.2 mg/kg/day PSL. Remission was defined as inactive disease according to the American College of Rheumatology. We compared the two responder groups for age at disease onset, sex, period from onset to TCZ treatment, indication of TCZ, various biomarkers at 2 years from initiation of TCZ, adjunctive immunosuppressant therapy at last visit, number of relapses under TCZ treatment, height, BMI, bone density, and Steinbrocker stage and class. Binary data were analyzed by Fisher’s exact test and quantitative data were analyzed by Mann-Whitney U test.

**Results:** Among 22 patients (male 12, female 10, 9–18 years old), 12 patients (55%) were classified as GR and 10 patients (45%) were IR. Two IR patients presented with persistent arthritis under TCZ. There were no significant differences between the groups in age at onset, sex, and period from onset to TCZ treatment. For the indication of TCZ treatment, 8 patients could not achieve remission at onset, 12 patients relapsed with systemic manifestations with/without arthritis upon tapering of PSL, and 2 patients suffered persistent arthritis. There was no significant difference in white blood cell count and serum CRP level between the groups after 2 years of observation. However, serum CRP remained at a measurable level in 3 IR patients.

Serum IL-6 in the IR group was significantly higher than in the GR group at 6 months, 1 year, and 2 years from initiation of TCZ (p = 0.008, p = 0.02, p = 0.003, respectively). ROC analysis revealed that the cut-off value of serum IL-6 at 6 months was 12.3 pg/mL (sensitivity 90%, specificity 75%, AUC 0.83) and 16.1 pg/mL (sensitivity 80%, specificity 75%, AUC 0.79) at 1 year. Serum IL-18 in the IR group was significantly higher than in the GR group at 1 and 2 years from initiation of TCZ (p = 0.001, p < 0.001, respectively). ROC analysis revealed that the cut-off value of IL-18 level at 1 year was 2328.4 pg/mL (sensitivity 70%, specificity 100%, AUC 0.91).

At the last visit, adjunctive immunosuppressant use, number of relapses, and daily steroid dose were significantly greater in the IR group than in the GR group (p = 0.02, p < 0.001, p < 0.001, respectively). Six IR patients had a Steinbrocker stage of II or greater (60%), but all GR patients remained in stage I (p = 0.003). Six of 13 patients with IL-18 > 2328.4 pg/mL and/or IL-6 > 16.1 pg/mL at 1 year after TCZ initiation presented with joint destruction at the last visit.

**Conclusion:** Serum levels of IL-6 and IL-18 at 1 year after initiation of TCZ could predict IR and joint destruction in patients with sJIA.

**Disclosure of Interest**

None Declared.

### P182 Efficacy, safety, pharmacokinetics and pharmacodynamics of canakinumab in patients with colchicine-resistant fmf: results from the phase 3 cluster trial

#### Seza Ozen^1^, Isabelle Kone-Paut^2^, Michael Hofer^3^, Jeroen van Der Hilst^4^, Ahmet Gül ^5^, Paul Brogan ^6^, Rafaelle Manna^7^, Sara Murias^8^, Omer Karadag ^9^, Sinisa Savic ^10^, Yankun Gong ^11^, Eleni Vritzali^12^, Guido Junge^12^, Fabrizio De Benedetti^13^, Eldad Ben-Chetrit^14^

##### ^1^Department of Pediatrics, Hacettepe University, Ankara, Turkey; ^2^Hôpital Kremlin Bicetre, University of Paris SUD, Paris, France; ^3^University of Lausanne, Lausanne, Switzerland; ^4^Department of General Internal Medicine, Radbound University Nijmegen Medical Centre, Nijmegen, Netherlands; ^5^Department of Internal Medicine, Istanbul University, Istanbul, Turkey; ^6^UCL Institute of Child Health, and Great Ormond Street Hospital NHS Foundation Trust, London, UK; ^7^Institute of Internal Medicine, Università Cattolica Sacro Cuore, Rome, Italy; ^8^Hospital La Paz, Madrid, Spain; ^9^Hacettepe University Faculty of Medicine, Ankara, Turkey; ^10^University of Leeds, Leeds, UK; ^11^Novartis Pharma, Shanghai, China; ^12^Novartis Pharma AG, Basel, Switzerland; ^13^IRCCS Ospedale Pediatrico Bambino Gesú, Rome, Italy; ^14^Rheumatology Unit, Hadassah–Hebrew University Medical Center, Jerusalem, Israel

###### **Correspondence:** Seza Ozen

**Introduction:** Canakinumab (CAN), a fully human, specific, anti-interleukin-1β monoclonal antibody, has been shown efficacious in the treatment of patients (pts) with colchicine-resistant familial Mediterranean fever (crFMF) in an open-label study.^1^

**Objectives:** The primary objective was to demonstrate that CAN 150 mg every 4 weeks (q4w) was superior to placebo (PBO) in resolving the flare by Day 15 with no new flares over 16 weeks (wks). Secondary objectives included: the proportion of pts who maintained optimal control of disease activity (absence of new flares) between Wk 16 and Wk 40 after dose reduction; evaluation of pharmacokinetics (PK), pharmacodynamics (PD) and safety of CAN in crFMF pts.

**Methods:** The study comprised 4 epochs: a 12-week (wk) screening epoch (E1), a 16-wk randomised treatment epoch (E2), a 24-wk randomised withdrawal epoch (E3) and a 72-wk open-label treatment epoch (E4). After lead-in in E1, pts were randomised to CAN 150 mg q4w or PBO in E2. Pts who did not flare in the CAN group in E2 were re-randomised to CAN 150 mg q8w or CAN withdrawal/PBO in E3. In E3, dose could be escalated up to 300 mg q4w for pts with a flare. For PK/PD assessments, serum samples for CAN concentrations and total IL-1β were collected at baseline (Day 1), and trough samples at wks 1, 3, 5, 9, 13 and 17. Safety assessments included adverse events (AEs) and serious AEs (SAEs).

**Results:** Of the 63 crFMF pts randomised in E2, 1 PBO pt discontinued the study. The proportion of responders at Wk 16 was significantly higher with CAN vs PBO (Table 5). Of the 59 pts who continued from E2 to E3 on any treatment regimen, 19 (E2 responders) were re-randomised to CAN 150 mg q8w or PBO/CAN withdrawl in E3 while remaining pts received open-label treatment. In E3, the proportion of pts who did not present new flares was numerically higher in the CAN vs PBO group (Table 5). In E3, 46% pts, including pts treated in open-label, maintained disease control with a prolonged dosing interval (q8w). 10% pts required up-titration to 300 mg q4w. No new safety findings or death were reported in CAN-treated pts through E3 (Table 5). In crFMF pts, the serum clearance and steady-state volume of distribution of CAN (liquid-in-vial) varied according to body weight, and were estimated to be 0.14 ± 0.04 L/day and 4.96 ± 1.35 L, respectively. The estimated half-life of CAN was 25 ± 6.4 days. Total IL-1β serum concentrations showed dose proportional increase.

**Conclusion:** Canakinumab 150 mg q4w was efficacious in resolving flare by Day 15 and preventing new flares in crFMF patients over 16 weeks. Approximately 46% patients did not flare with a prolonged dose interval (q8w) at the end of Week 40 (epoch 3) compared to 30% patients who did not flare when canakinumab was completely withdrawn.The PK/PD results observed with the liquid-in-vial form of canakinumab were similar to those observed in CAPS and SJIA. No new or unexpected safety issues were reported over 40 weeks of treatment.

^1^Gül A, et al. *Arthritis Res & Ther*. 2015;17:243.

**Trial registration identifying number:** NCT02059291

**Disclosure of Interest**

S. Ozen Speaker Bureau of: Novartis and SOBI, I. Kone-Paut Grant/Research Support from: Novartis, SOBI and Roche, Consultant for: Novartis, SOBI, Pfizer, AbbVie and Roche, M. Hofer Consultant for: Novartis and AbbVie, J. van Der Hilst: None Declared, A. Gül: None Declared, P. Brogan: None Declared, R. Manna Grant/Research Support from: Novartis, Consultant for: Donato Rigante and Elena Verrecchia, Employee of: Elena Verrecchia, S. Murias: None Declared, O. Karadag: None Declared, S. Savic Grant/Research Support from: SOBI and CSL, Consultant for: Novartis, Shire and SOBI, Y. Gong Employee of: Novartis, E. Vritzali Employee of: Novartis, G. Junge Employee of: Novartis, F. De Benedetti Grant/Research Support from: Pfizer, AbbVie, Roche, Novartis, Novimmune and BMS, E. Ben-Chetrit Consultant for: Novartis.

**Table 5 (Abstract P182).** Efficacy results and summary of safety

Proportion of responders at Week 16 (E2), n (%)	PBO N = 32	CAN 150 mg q4w N = 31	p-value
	2 (6.3)	19 (61.3)	<0.0001*
Proportion of patients with no new flare at Week 40 (E3), n (%)	PBO N = 10	CAN 150 mg q8w N = 9	p-value
	3 (30.0)	7 (77.8)	0.0513
*Safety*			
	PBO N = 32	Any CAN^#^ E2 N = 58	Any CAN^#^ E2 + E3 N = 61
Exposure to CAN, pyr	3.1	16.4	45.6
Number of AEs (AE rate/100 pyr)	54 (1754.8)	134 (816.7)	332 (728.2)
Number of SAEs (SAE rate/100 pyr)	3 (97.5)	7 (42.7)	17 (37.3)

*Indicates statistical significance (one-sided) at the 0.025 level. ^#^Any patient who received a dose of CAN during E2 or E3. n = number of patients who responded; N = number of patients evaluated for response. AE, adverse event; CAN, canakinumab; E, epoch; PBO, placebo; q4w, every 4 weeks; q8w, every 8 weeks; pyr, patient-years; SAE, serious adverse event

### P183 Severe adverse events associated with use of biologic therapy in patients with systemic juvenile arthritis: a single-center study

#### Ricardo A. Russo, María M. Katsicas 

##### Immunology & Rheumatology, Hospital de Pediatría Garrahan, Buenos Aires, Argentina

###### **Correspondence:** Ricardo A. Russo

**Introduction:** Biologic agents have revolutionized the treatment of Systemic Juvenile Arthritis (sJA) due to their high efficacy and safety. However, with the increased and prolonged use of these agents there is uncertainty regarding short- and long-term adverse events.

**Objectives:** To describe and analyse Severe Adverse Events (SAEs) occurring during biologic therapy in patients with sJA.

**Methods:** Single-center, retrospective, longitudinal study based on review of a prospectively built, ad-hoc database and medical records. Patients with sJA according to ILAR criteria who received at least one biologic agent were included Observation period was January 2007-December 2016. Demographic data, disease duration at start of biologic treatment, time from start of biologic treatment to occurrence of SAEs, type and dosage of biologic agents, total time of biologic therapy exposure, type of SAE, and outcomes were recorded. SAEs were defined as any untoward medical occurrence that occurs during observation and results in death, a life-threatening illness, hospitalization, persistent or significant disability or incapacity, or a medically significant event that jeopardized the health of the patient and required intervention to prevent one of the other outcomes listed.

**Results:** 86 biologic treatments were administered to 47 sJA patients (32 girls; age at disease onset: 3.5 years). Age at start of biologic therapy: 6 years; disease duration at start of biologic therapy: 2.3 years. One biologic agent was prescribed to 23, 2 (sequentially) to 13, 3 to 8, 4 to 2 and 5 to 1 children. Patients were exposed to: etanercept (ETA, 29 treatments, 56.8 patient-years), tocilizumab (TOC, 22, 45.8), adalimumab (ADA, 11, 25.2), anakinra (ANA, 7, 4.0), canakinumab (CAN, 6, 10.8), abatacept (5, 4.2), infliximab (4, 5.4), and rituximab (2, 1.0). Total time of therapy exposure: 152.5 patient–years; median duration of each biologic therapy course: 1.25 years. SAEs occurred at 4 (median) months after the start of therapy. Twenty-three (15 non-infectious) SAEs were recorded in 18 children: macrophage activation syndrome (MAS) fulfilling both Ravelli’s and ACR/EULAR criteria in 5 children; events including cytopenias and/or hypertransaminemia but not fulfilling MAS criteria (possible MAS) in 6 children; also, hypogammaglobulinemia (in 3) and peri-infusional rash (in 3) were observed. One patient on ETA developed a lupus-like disease. Defined MAS occurred in patients on CAN (2), ANA (1), TOC (1) or ETA (1), while possible MAS was recorded in therapies with TOC (5) and ETA (1). Incidence of defined and overall MAS was 3.3 and 7.2 events per 100 patient-years respectively. Overall MAS occurred more frequently during IL-1- or IL-6-blocking therapy than during other treatments (p = 0.004). Infectious SAEs were observed in patients receiving ETA (6), ADA (1) or TOC (1). At the time of SAEs 17 patients were receiving MTX, 11 corticosteroids. SAEs rate was 15.1 per 100 patient-years (27 in anti-IL-1, 22 in anti-IL-6 and 10 in anti-TNF therapies). All patients eventually improved after discontinuation of treatment and appropriate therapy. No malignancy, TB reactivation or deaths were observed.

**Conclusion:** SAEs arising during biologic therapy are frequently observed in children with sJA. They usually appear during the initial months of therapy. While infectious SAEs appear to be more frequent in patients receiving TNF inhibitors, other SAEs (including MAS and cytopenias/liver enzymes elevation) occur at a higher rate during anti-IL-1 or anti-IL-6 therapy. Use of anti-IL-1 or anti-IL-6 agents may modify the clinical expression of MAS. Multicenter studies and international registries may provide evidence on comparative safety of different biologic agents.

**Disclosure of Interest**

None Declared.

## Autoinflammatory diseases

### P184 Differential diagnosis of non-bacterial and acute hemotogenous osteomyelitis

#### Olga Kopchak, Mikhail Kostik 

##### Saint Petersburg State Pediatric Medical University, Saint Petersburg, Russian Federation

###### **Correspondence:** Olga Kopchak

**Introduction:** Non-bacterial and acute hematogenous osteomyelitis (NBO and AHO, relatively) are inflammatory diseases of the musculoskeletal system, affecting children and adolescent. In some cases the diseases may have a similar clinical manifestation, therefore require a differential diagnostics.

**Objectives:** The aim of the study is to consider clinical and laboratory features for patients with NBO and AHO and to determine criteria of differential diagnosis, which could discriminate NBO from AHO.

**Methods:** The study included 138 patients (91 with NBO and 47 with AHO). All patients underwent a routine blood test (WBC, platelets, ESR, C-reactive protein (CRP) and hemoglobin levels), a radiological examination and a bone biopsy with evaluation of bacteriological and morphological data

**Results:** We did not reveal any gender difference between NBO and AHO. The age of onset disease was 11.0 (6.2; 12.9) and 7.3 (2.5; 10.6) years with NBO and TBO relatively. Significant differences (p = 0,0000001) were obtained in the diagnostic delay – 0.1 (0.003;0.17) and 6.3 (2.0;17.8) months with NBO and AHO. AHO is a typical monofocal process, while monofocal lesions were noted in 1/3 cases with NBO. Fever was recorded in 43/47 (91.5%) and 31/91 (34.1%) with NBO and AHO relatively. In case of AHO higher values of WBC, ESR and CRP 12.6 (8.4; 15.5)*10^9^/l, 40.0 (25.0; 58.0) mm/h, 65.0 (20.0; 146.0) mg/l were recorded in comparison with NBO – 7.5 (6.2; 9.0) *10^9^/l, 26.0 (12.0; 40.0) mm/h, 8.0 (3.6; 30.0) mg/l respectively. After receiving the main results of the study diagnostic criteria with the highest sensitivity, specificity and diagnostic odds ratio were chosen**,** which allows to differentiate non-bacterial osteomyelitis from acute hemotogenous osteomyelitis, i.e a radiographic changes, apyrexia, number of foci >1.0, CRP ≤ 55.0 mg/l, negative bone culture. The following diagnostic rule was formulated: NBO can be differentiated from AHO in the presence of at least 2 diagnostic criteria.

**Conclusion:** Differential diagnosis of non – bacterial and acute hemotogenous osteomyelitis can be based on a combination of proposed clinical and laboratory criteria

**Disclosure of Interest**

None Declared.

### P185 New pathogenic familiar heterozygous NLRP3 gene missence variant associated with reccurent pericardatis clinical presentation

#### Dragana S. Lazarevic^1^, Jelena Vojinovic^2^

##### ^1^Pediatric Rheumatology, Clinic of Pediatrics, Clinical Center Nis, Nis, Serbia; ^2^Faculty of Medicine, University of Nis, Nis, Serbia

###### **Correspondence:** Dragana S. Lazarevic

**Introduction:** Autoinflammatory syndromes represent the wide spectrum of rare diseases (associated with genetic disturbances) characterized by the presence of chronic or recurrent systemic inflammation with diverse clinical presentation.

**Objectives:** We report a case report of child with newly confirmed heterozygous missence variant in NLRP3 gene with clinical features associated Muckle-Wells syndrome.

**Methods:** We report a case report of child with newly confirmed heterozygous missence variant in NLRP3 gene with clinical features associated Muckle-Wells syndrome.

**Results:** Ten months old boy was admitted at our hospital due to pericarditis and fever, with no skin changes, no arthritis, and no visceromegaly but with severe inflammatory markers, including serum amyloid, and hypochromic mycrocitic anemia. All cultures were negative. He was treated with antibiotics, non-steroid anti-inflammatory drugs (NSAIDs) and prednisone, but relapsed whenever steroids were tapered. Chest CT scan had confirmed presence thick pericardial and pleural effusion while pleural punctate revealed increased number of polymorphonucler cells and increase in protein content without any evidence for infection. Since his mother had a history of neurosensorial hearing loss of undefined etiology and persistently elevated inflammatory markers we suspected on autoinflammatory disease background (possible idiopathic recurrent pericarditis). That is why we have initiated continuous treatment with ibuprofen and colchicine. DNA sample was analyzed but typical MEFV mutations in egzone 2 and 3 were not found. Since boy continued to have periodical attacks of fever and pericardial effusions, approximately on 2 weeks we were forced to add methyl-prednisolon pulses on 10 days. Unfortunately this has not stopped attacks of fever and pericarditis. Additional genetic testing have revealed presence of totally new pathogenic heterozygous missence variant c.1805A > G (NM_001079821.2) in NLRP3 gene causing the substitution of amino acid glutamine with amino acid arginine at position 602. This variant has not been yet reported in previous patient’s database with NLRP3 associated autoinflammatory diseases. The presence of this variant was confirmed in the mildly affected mother with sensoneural deafness, and is compatible with the phenotype of NLRP3 associated Muckle-Wells syndrome. Meanwhile, our patient achieved disease remission that is why anakinra was not introduced and steroids could be tapered and stopped, but continued colchicine for one year. Six months after stopping colchicine boy developed persistent arthritis of the right ankle with no response to NSAIDs why methotrexate has been introduced.

**Conclusion:** Clinical manifestations of some patients can provoke differential diagnostic and treatment dilemmas. We are still in doubt whether our patient had atypical systemic JIA (fever, inflammation and serositis with late onset of arthritis and without skin, lymph node or organ involvement) or autoinflammatory disease that belongs to the cryopyrinopathy group.

**Disclosure of Interest**

None Declared

### P186 A cohort of patients with autoinflamatory diseases followed-up in a unit of paediatric and transitional rheumatology: a descriptive study

#### Maria Llop Vilatella, Alina Boteanu, Maria Angeles Blazquez Cañamero, Walter Alberto Sifuentes Giraldo, Maria Luz Gamir Gamir

##### Rheumatology, University Hospital Ramon Y Cajal, Madrid, Spain

###### **Correspondence:** Maria Llop Vilatella

**Introduction:** The autoinflammatory diseases (AD) are uncommon, most of them are presented as episodes of recurrent fever and may be accompanied by other inflammatory symptoms. This group of diseases includes polygenic entities (without a single known genetic mutation) such as Behçet’s disease (BD), systemic-onset juvenile idiopathic arthritis (soJIA), Chronic recurrent multifocal osteomyelitis (CRMO) and PFAPA syndrome. On the other hand, we found the entities that present with specific monogenic mutations, such as Familial Mediterranean Fever (FMF), TNF receptor-associated periodic syndrome (TRAPS), hyper-IgD syndrome and periodic fever (HIDS), cryopyrinopathies (FCAS, MWS, CINCA), Blau’s syndrome and PAPA. A group of patients who cannot be classified into a specific diagnosis are clustered as recurrent fever without known genetic anomaly (RFW).

**Objectives:** To describe and compare the clinical features of monogenic and polygenic AD and RFW seen in a paediatric and transitional rheumatology unit of a Spanish tertiary care hospital.

**Methods:** We performed a retrospective study including 39 patients with AD followed-up in our center.

**Results:** The distribution of diagnoses was: sJIA 19 patients (48.72%), BD 5 (12.82%), PFAPA 6 (15.38%), CRMO 3 (7.69%), RFW 4 (10.26%), HIDS 1 (2.56%) and CINCA 1 (2.56%). Patients came from different regions of Spain, being 22 of them boys (56.41%) and 17 girls (43.59%). The genetic study was performed in 12 patients, being positive in 7 (17.95%). Mean age at onset of symptoms was 5 ± 5.65 years in monogenic diseases, 7.96 ± 4.84 years in polygenic disorders and 9.5 ± 5.91 years RFW. Delay in diagnosis in monogenic diseases was higher than in polygenic diseases (67 ± 69.29 months vs. 24.03 ± 30.33 months, respectively). The clinical manifestations more frequently found were fever, followed by joint involvement, being more common in monogenic diseases than in polygenic disorders (Table 6). Haemoglobin levels were lower in monogenic than in polygenic diseases 9.95 g/dL ± 0.63 vs. 11.69 g/dL ± 2, ESR and CRP was higher in monogenic diseases 106 mm/h ± 68.5 and 80.5 mg/L ± 84.14 vs. 56.1 mm/h ± 33.78 and 57.95 mg/L ± 57.95, unlike ferritin that was more elevated in polygenic disease 896 μg/dL ± 1788.34 than in monogenic diseases 183 μg/dL ± 195.7. During his follow up 84.62% of patients received corticosteroids, 51.8% methotrexate and 46.15% biological therapy.

**Conclusion:** sJIAs was the most frequent AD in our center. All the patients had a similar gender distribution. Delay in diagnosis was greater in monogenic diseases compared with polygenic disorders. Fever and joint involvement were the more common clinical manifestations, especially in monogenic diseases. Ferritin levels were higher in polygenic diseases, whereas CRP and ESR which were higher in monogenic diseases. During the follow-up most patients required treatment with corticosteroids and approximately half of them required biological therapy.

**Disclosure of Interest**

None Declared

**Table 6 (Abstract P186).** See text for description

	Monogenic	Polygenic	Recurrent fever
Fever	100%	81.25%	100%
Joint involvement	100%	62.5%	75%
Rash	100%	59.38%	0%
Lymphoadenopathy	50%	46.88%	25%
Splenomegaly	100%	12.5%	0%
Abdominal involvement	30%	21.88%	25%

### P187 Genetic and clinical profile of a paediatric population with FMF in Sicily

#### Maria Cristina Maggio^1^, Carmelo Fabiano^2^, Roberto Scondotto^1^, Giuliana Vitaliti^1^, Giovanni Corsello^1^

##### ^1^University Department Pro.Sa.M.I. “G. D’Alessandro”, University of Palermo, Palermo, Italy; ^2^Molecular Genetic Laboratory, Haematology and Rare Diseases of blood and haematopoietic organs, Ospedali Riuniti Villa Sofia-Cervello, Palermo, Italy

###### **Correspondence:** Maria Cristina Maggio

**Introduction:** Familial Mediterranean fever (FMF) is an Autoinflammatory syndrome that is common in children in Mediterranean countries.

**Objectives:** The real prevalence of FMF in Sicilian children is unknown and need a wide population study. Furthermore, there are no data on the real prevalence of the different mutations between FMF patients and the concordance and/or discordance in clinical and biochemical parameters between patients of different generations.

**Methods:** We collected clinical, biochemical and genetic data of 77 patients in paediatric age (1-16 years) affected by recurrent attacks of fever (about 3 days long) with variable association with the clinical symptoms typical of the disease (abdominal pain, serositis, arthralgia and/or arthritis, myalgia, erysipelas like erythema, oral aphthosis).

**Results:** We found 38 patients with clinically relevant mutations and 39 patients with polymorphism in the MEFV gene (R202Q and or Nt1588-69G > A, homozygous or heterozygotes; 33 R202Q heterozygotes and 6 R202Q homozygous patients. The most frequent mutations that we observed in our patients were: E148Q (11 heterozygotes); R408Q (6 heterozygotes); P369S (6 heterozygotes); M694V (5 heterozygotes, 1 homozygous patient); I591T (3 heterozygotes). Some of them had 2 or more mutations in association. All the patients were treated with colchicine with a complete (95%) or partial response to the treatment. None of the patients developed amyloidosis. Three patients from unrelated families, had a vasculitis (2 Kawasaki Disease; 1 Schoenlein-Hoenoch Purpura) and all presented the same association: P369S and R408Q.

**Conclusion:** This finding verifies the importance of molecular diagnosis and detailed sequencing which is recommended to perform in particular for the countries with a high risk of FMF.

In several instances, family studies provided the prevalence of a single mutation in patients experiencing a pathogenic effect, with molecular evidence for pseudodominant transmission. We evidenced a variable clinical and serological pattern between patients in the same family; the genetic study was in fact extended to parents and brothers of the index case, with the recommendation to dose Serum amyloid A (SAA), blood pressure and evaluate an urine analysis to exclude proteinuria.

The M694V homozygous and heterozygous genotype was found to be associated with a higher prevalence of amyloidosis and arthritis and higher levels of SAA. The parents of our patients with M694V mutation have more severe clinical manifestations and a lower response to colchicine.

Further studies are needed to highlight generational differences of clinical spectrum and SAA levels in FMF patients of the same family, carrying the same mutations.

**Disclosure of Interest**

None Declared.

### P188 Anti-IL1 in patients with low penetrance mutations for autoinflammatory diseases: tuscany and sicilian case series from paediatric to adult age

#### Maria Cristina Maggio^1^, Luca Cantarini^2^, Giovanni Corsello^1^, Umberto Corpora^3^, Rolando Cimaz^4^

##### ^1^University Department Pro.Sa.M.I. “G. D’Alessandro”, University of Palermo, Palermo, Italy; ^2^Research Center of Systemic Autoinflammatory Diseases and Behçet’s Disease Clinic, Department of Medical Sciences, Surgery and Neurosciences, University of Siena, Siena, Italy; ^3^Universitary Department Pro.Sa.M.I. “G. D’Alessandro”, University of Palermo, Palermo, Italy; ^4^Pediatric Rheumatology Unit, Neurofarba Department, AOU Meyer, University of Florence, Florence, Italy

###### **Correspondence:** Maria Cristina Maggio

**Introduction:** Patients with low penetrance mutations for Autoinflammatory syndromes (AID) can have severe clinical manifestations, which require to be treated with biological drugs anti-IL-1.

**Objectives:** To evaluate the response of AID to treatment with the recombinant human IL-1 receptor antagonist anakinra or with the anti-IL-1b.

**Methods: W**e enrolled from 3 centers (U.O. of Rheumatology, Universitary Hospital “S. Maria Le Scotte”, Siena; S.O.D.C. of Paediatric Rheumatology, “A. Meyer” Hospital, Florence and U.O. of Paediatric Clinic, Children Hospital “G. Di Cristina”, Palermo) 26 patients with SAI and low penetrance mutations, with age: 0.8-58 years (11 M, 15 F; age:4-62 years; medium age of paediatric patients: 11.2 years). The symptoms started in paediatric age in all the patients; however adult patients received the diagnosis in adult age.

**Results:** All the patients (9 CAPS, 10 TRAPS, 2 HIDS, 2 FMF, 3 sJIA) received anti-IL-1b drugs (anakinra or canakinumab). The subjects kept a diary of symptoms at the diagnosis and at the outset, and underwent clinical and laboratory assessments, including measurement of the SAA, ESR, CRP, blood count, urinalysis.

At the outset, the 84.6% showed recurrent episodes of fever, variously associated with: rash (61.5%), abdominal pain (50%), arthralgia and/or myalgia (88%), arthritis (46%). All the patients, before starting anti-IL-1b drugs, were treated with NSAIDs, steroids, DMARDs, colchicine with a poor control of the disease. The 30.7% associate the anti-IL-1b to one or more of other drugs. The 57.7% (38.5% between children, 19.2% between adults) showed a complete remission; the 19.2% incomplete, the 23.1% did not respond. SAA was increased in 88.5% (M: 155,86; n.v. < 6,4 mg/l), reduced in 58%. CRP was increased before anti-IL-1b drug in the 50%, with a normal value in the 92% after the drug was started. ESR was increased in the 69.2%, with a normal value in the 42.3%.

Proteinuria was detected in the 8% before the anti-IL-1b drug was started and was in the normal range after they was treated with the biological drug.

In children, prevalent clinical manifestations were abdominal pain and arthritis; in adults thorax pain and pericarditis were more frequent.

**Conclusion:** The clinical features of the AID were correlated with age, also in patients with low penetrance mutations: some manifestations were more frequent in adults, others in childhood. The remarkable response on clinical and haematological parameters (76.9% of all the patients) to anakinra or canakinumab suggests that IL-1β has a fundamental role in the pathogenesis of inflammation associated with low penetrance mutations as well. In paediatric age, IL-1 blockade higher efficacy was probably linked to a more severe clinical phenotype.

**Disclosure of Interest**

None Declared.

### P189 Genetic and clinical profile of a sicilian population with R92Q mutation

#### Maria Cristina Maggio^1^, Carmelo Fabiano^2^, Giuliana Vitaliti^1^, Giovanni Corsello^1^

##### ^1^University Department Pro.Sa.M.I. “G. D’Alessandro”, University of Palermo, Palermo, Italy; ^2^Molecular Genetic Laboratory, Haematology and Rare Diseases of blood and haematopoietic organs, Ospedali Riuniti Villa Sofia-Cervello, Palermo, Italy

###### **Correspondence:** Maria Cristina Maggio

**Introduction:** Gene TNFRSF1A mutation is linked to TRAPS, autosomal dominant Autoinflammatory Disease (AID) with recurrent attacks of fever (2-3 weeks long), abdominal pain, vomiting, serositis, arthralgia and/or arthritis, myalgia, fasciitis, rash. The disease starts precociously and amyloidosis is reported in the 25% of the patients. Patients carrying the mutation R92Q usually show a mild clinical phenotype, with an extreme interindividual variability. Arthralgia and serositis are frequently less severe, however oral ulcers and pharyngitis are recurrent.

**Objectives:** We studied the clinical and biochemical impact of the mutation R92Q in our population and the treatment outcome in all the patients with clinical relevant symptoms.

**Methods:** We followed 15 patients (6 M and 9 F), 11 children and 4 adults, carrying the R92Q heterozygous mutation of the gene TNFRSF1A. The diagnosis of children were done at the age of 4-14 years, o the adults was performed following the sons diagnosis. SAA levels were significantly high in 8/11 children and in 2/4 adults.

**Results:** All the symptomatic patients were treated with NSAIDs, steroids, colchicine with a variable control of the disease. The colchicine was not sufficient in 4/5 patients and 2 of them were treated with the anti-IL-1 β biological drug canakinumab.

**Conclusion:** Functional studies performed on R92Q evidenced a changed conformational structure vs. the wild type.

Our patients showed polymorphic clinical features: some of them are asymptomatic, other record different symptoms with an intrafamilial variability. In paediatric age, the clinical phenotype is more severe also in correlation with the symptoms of parents carrying the same mutation. We stress the data that all our patients underwent the genetic study because they recorded symptoms linked to AID.

**Disclosure of Interest**

None Declared.

### P190 A paediatric FMF patient with recurrent priapism during attacks

#### Balahan Makay, Nesrin Gülez

##### Behçet Uz Children s Hospital, Izmir, Turkey

###### **Correspondence:** Balahan Makay

**Introduction:** Priapism, a prolonged penile erection lasting >4 hours, is a rare condition in childhood. The commonest causes of priapism in children are sickle cell disease, leukaemia, trauma, idiopathic, and pharmacologically induced. To date, the association of familial Mediterranean fever (FMF) and priapism has not been reported.

**Objectives:** To report an FMF patient experiencing priapism during attacks.

**Methods:** To report an FMF patient experiencing priapism during attacks.

**Results:** A 20-months-old boy was referred to our pediatric rheumatology department with the episodes of fever, monoarthritis, and pustules on the face recurring every month since 10-months of age. Because MEFV analysis revealed M694V homozygote mutation, he was started 0.25 mg/day colchicine by his pediatrician when he was 14-months-old. Despite the adjustment of colchicine to 0.5 mg/day, he continued to have recurrent fever and pustules. In his immunologic evaluation, white blood cell number, immunogobuline G,M,A levels, lymphocyte subgroup disturbation, phagoburst test was normal.He was given a short-course of corticsteroid because of fever and a painful erythema nodosum-like lesion when he was 26-months old. PSTPIP1 mutation was negative for PAPA. When he was 2.5 years-old, he had penile erection during an attack. Colchicine dose was increased to 1 mg/day. He experienced two more attacks of priapism with fever and pustules of the face. He was started an anti-interleukine 1 treatment, anakinra 1 mg/kg/day. He never had priapism, pustules, arthritis and fever attacks after the initiation of anakinra. After one year, anakinra was stopped. He is currently taking only colchicine since one year and doing well.

**Conclusion:** To the best of our knowledge, this is the first case showing an association of priapism with FMF attacks.

**Disclosure of Interest**

None Declared.

### P191 Severe arthritis as clinical presentation in a a novel case of COPA syndrome

#### Stefano Volpi^1^, Marcello Mariani^1^, Claudia Pastorino^1^, Anthony K. Shum^2^, Angelo Ravelli^1^, Marco Gattorno^1^, Paolo Picco^1^

##### ^1^Pediatria 2, Istituto Giannina Gaslini, Genova, Italy; ^2^Division of Pulmonary and Critical Care, University of California San Francisco, San Francisco, CA, USA

###### **Correspondence:** Marcello Mariani

**Introduction:** Heterozygous mutations of *COPA* gene have been recently linked to an autoimmune syndrome characterised by polyarthritis and lung disease with interstitial fibrosis or alveolar haemorrhages as complication (1). The gene encodes for a regulator of vesicular retrograde transport between Golgi and the endoplasmic reticulum (ER). In most cases the presenting symptoms are cough and tachypnea, that appear in the early childhood with articular manifestations appearing lately before di age of 10. At laboratory tests, autoimmunity is characterised by the presence of autoantibodies, with the majority of patients showing a frankly positive ANA titer (80%). Rheumatoid factor is reported to be raised in 43% of cases; a few patients showed also positivity for cANCA and pANCA (2).

**Objectives:** We report one case referring to our centre of a young girl that presented at 3 year of age with polyarticular arthritis.

**Methods:** The patient was clinically evaluated by the pediatric rheumatology unit of Istituto Giannina Gaslini. The family gave permission for publication of clinical and laboratory data and photographic images as well as molecular analysis.

**Results:** The patient firstly referred to our centre at 3 year of age for a polyarticular arthritis involving metacarpophalangeal joints, hip and cervical spine. Lab results were performed showing raised ESR and CRP, high titer rheumatoid factor and antinuclear antibodies positivity. For a newly-onset of a persistent cough with no evidence of infectious process a chest X-ray and then a lung CT scan was performed showing an interstitial lung disease with tree-in-a-bud appearance and air-filled cysts. In order to treat the arthritic process oral and intra-articular steroids were administered with good response. Methotrexate and abatacept used as steroid-sparing drugs failed to control disease progression. The molecular analysis of *COPA* gene showed the reported c.698G > A mutation. Patient intentionally discontinued therapy for 3 years as well as clinical follow-up. After that time with patient referring again at our centre, a very severe osteoarthritis with joint damage and deformities of multiple joints was found.

**Conclusion:** COPA syndrome could be more common than thought due the autosomal dominant inheritance. When molecular diagnosis is confirmed a therapeutic aggressive approach is required to prevent permanent joint deformities and reduce the risk of life threatening complications such as pulmonary haemorrhages.

1. Vece TJ, Watkin LB, Nicholas SK, Canter D, Braun MC, Guillerman RP, et al. Copa Syndrome: a Novel Autosomal Dominant Immune Dysregulatory Disease. J Clin Immunol. 2016;36(4):377–87.

2. Watkin LB, Jessen B, Wiszniewski W, Vece TJ, Jan M, Sha Y, et al. COPA mutations impair ER-Golgi transport and cause hereditary autoimmune-mediated lung disease and arthritis. Nat Genet. Nature Publishing Group; 2015;47(6):654–60.

**Disclosure of Interest**

None Declared.

### P192 Paediatric autoinflammatory diseases in cyprus: insights from the national registry

#### Despoina Maritsi^1^, Avraam Elia^2^, Maria Kolliou^2^, Maria Kousparou^2^, Margarita Onoufriou^1^

##### ^1^Rheumatology Unit, Department of Paediatrics, ‘Archbishop Makarios III” Children’s Hospital, Nicosia, Cyprus; ^2^Department of Paedatrics, “Archbishop Makarios III” Children’s Hospital, Nicosia, Cyprus

###### **Correspondence:** Despoina Maritsi

**Introduction:** Autoinflammatory diseases (AID) are rare in theory; however with raising awareness their incidence seems to be increasing. Early recognition and appropriate management prevents long-term sequelae and establishes an improved quality of life.

**Objectives:** To describe the epidemiology, demographic, clinical and disease characteristics as well as treatment options in patients with AID followed up in a national referral center for Pediatric Rheumatology.

**Methods:** Cross sectional observational study including thirty-five children with an AID regularly followed up at “Archbishop Makarios III” Paediatric Hospital of Nicosia, Cyprus from January 2012 to date. Children with PFAPA, SJIA and Behcet’s disease were excluded from this cohort.

**Results:** Of those 35 children (55% boys), 62% were diagnosed with mutation proven FMF, 3.5% with TRAPS, 3.5% with HIDS, while the remaining were classified as a multisystem autoinflammatory disease. 69% of patients were Cypriots, 8% were of Eastern Mediterranean origin, 8% were Eastern European while the remaining were of mixed ethnic origin. Mean age at disease onset was 8.4 years (range 0.4 to 17 years). Mean time from disease onset to diagnosis was 1.5 years (range 0.6 to 3.5 years). Positive family history was evident in 25% of cases. All patients presented with fever of unknown origin. Other major symptoms included abdominal pain 65%, diarrhoea 18%, thoracic pain 7%, asthenia 27%, weight loss 14%, headache 42%, arthralgia and myalgia 23%. Skin manifestations were evident at 21% of patients; arthritis was present in 27% of patients, 25% of patients developed serositis (primarily pericarditis) at some point throughout the course of their disease. Lymph node enlargement was noted in 11%, parotid enlargement in 7%, while 14% had splenomegaly. Uveitis was present in 3.4%. Renal involvement was observed in 12% of our cohort (proteinuria). Renal biopsy was performed on two occasions proving secondary amyloidosis. Oral apthous ulcers were noticed in 15% of patients while hidradenitis suppurativa in 3.5%. The majority of our patients had received NSAIDS prior to referral; 22% reported temporal alleviation of their constitutional symptoms. Colchicine remained the mainstay of treatment for the majority of these patients (79%). 67% of these children had received steroids during the course of their disease. Eight 23% of the patients received biologics (IL-1 blocking agent). Following introduction of the biologic, these patients remained in clinical remission. Anti IL-1 treatment was successfully discontinued in one.

**Conclusion:** Based on our current data the prevalence of pediatric AID in Cyprus is 0.05%, with an annual incidence of 1/10.000 in the general population. As expected, FMF is the most common diagnosis; 31% of patients have an unclassified AID. Formation of a national registry helps identifying rare disorders and acting suitably. Wider, international collaborations will further improve our knowledge and education on the pathophysiology and medical management of the AID.

**Disclosure of Interest**

None Declared.

### P193 Antiphospholipid syndrome as a diagnostic and therapeutic problem

#### Aleksandra Sobiesiak, Aleksandra Rybkowska, Maria Tomaszek, Violetta Opoka-Winiarska

##### Department of Pediatric Pulmonology and Rheumatology, Medical University of Lublin, Poland, Lublin, Poland

###### **Correspondence:** Violetta Opoka-Winiarska

**Introduction:** Antiphospholipid syndrome (APS) is a multisystem autoimmune condition characterized by vascular thromboses associated with persistently positive antiphospholipid antibodies. There is currently a paucity of data in pediatric APS and there are no validated criteria for diagnosing pediatric APS [1]. The most severe form of the disease is a catastrophic antiphospholipid syndrome (CAPS).

**Objectives:** The aim of our study is to present the diagnosis and the treatment of an 17-year-old patient with severe form of APS.

**Methods:** Retrospective analysis of patient’s history.

**Results:** The first symptoms of the disease occurred in March 2016. The boy was admitted to clinic because of the pain on the chest for 3 weeks with growing symptoms: dyspnoea, cough, fatigue, feeling weak and lack of appetite. During the first hospitalization the pulmonary embolism was diagnosed. Additionally in blood tests elevated concentrations of D-dimer, antibodies to β2-glycoprotein I in IgM class, lupus anticoagulant, antinuclear antibodies (low titer) and antibodies to dsDNA were found. Other thrombophilic risk factors were excluded, but fact, that patient was taking dietary supplements could be important. Based on the results of the study and the observed symptoms, APS was suspected. The low molecular weight heparin (LMWH) was initially used in the treatment, but without improvement. In the following days the patient’s condition was gradually deteriorated and symptoms of respiratory failure were observed. The CT pulmonary angiogram showed“ image that could correspond to multiple infarct lesions”. Although the patient did not meet the criteria for a catastrophic antiphospholipid syndrome, aggressive treatment: glucocorticosteroids (GC), immunoglobulins with antibiotherapy and chloroquine was used due to the deterioration of the boy’s condition.

The treatment resulted in a slow improvement, after four week the dose of GC was tapered and warfarin was included in the treatment, while LMWH was discontinued. After next four weeks of treatment the patient showed no symptoms of respiratory failure and moderate efford was tolerated well. Additional studies have shown low titer of anti-dsDNA antibodies, anti-cardiolipin antibodies - low profile, lupus anticoagulant was not determined due to warfarin. Control CT pulmonary angiogram showed partial regression of embolic alterations and almost complete regression of inflammatory/non-inferior lesions. Spirometry test within normal limits, moderately reduced diffusion capacity, no restriction.

Six month after first symptom we have not seen signs of respiratory failure. Control of angio-CT have shown complete regression of embolic changes and further regression of inflammatory changes. Prophylaxis has continued to be recommended for warfarin. In addition to the treatment remain chloroquine and GC (10 mg/day). The patient is now in good general condition. He remains under the care of adult rheumatology clinic.

**Conclusion:** In summary presented patient did not meet the criteria of APS and CAPS criteria, although we suspected APS, CAPS and lupus and the implementation of aggressive treatment has helped to cushion the progressive symptoms of respiratory failure and progressive improvement in the condition of the patient. He need further observation. We have a lot of questions for pediatric APS. One was about elements of diet supplement as trigger for APS.

**Disclosure of Interest**

None Declared.

### P194 Anhidrotic ectodermal dysplasia with autoinflammatory manifestations

#### F Orlando, E Bruzzese, M. I. Spagnuolo, A Guarino, M Alessio

##### Department of Translational Medical Science, Section of Pediatrics, University of Naples Federico II, Naples, Italy

###### **Correspondence:** F Orlando

**Introduction:** X-linked anhidrotic ectodermal dysplasia with immunodeficiency is linked to mutations of the gene NF-kB essential modulator (NEMO). One-fourth of the patients suffer from inflammatory disorders such as inflammatory bowel disease and arthritis.

**Objectives:** To describe the clinical manifestations of a patient with NEMO mutation and the efficacy of anti-IL-1 therapy in them.

**Methods and results:** D. (male, aged 7) born after regular term pregnancy characterized by intra-uterus growth retardation from the eighth month of pregnancy. At the age of one, the clinical situation of D. was characterized by low height and weight, delayed psycho-motion development, blindness, relapsing sepsis, eczema, limited sweating, left ankle arthritis. Due to the presence of hypogammaglobulinemia he underwent infusions of immunoglobulin from the age of one, resulting in a reduction of infectious episodes. Considering the persistence of arthritis, recurrent fever, periodical increase of inflammatory indexes, suspecting autoinflammatory syndrome, at the age of 16 months he started anti-TNFα therapy with remarkable clinical and lab improvements. At the age of 30 months he was diagnosed anhidrotic ectodermal dysplasia because of the presence of exone 10 mutation (c.1167dup) of the NEMO gene. At the age of 5 his clinical situation worsened with low growth, systemic inflammation and left ankle arthritis referred to chronic osteomyelitis. He underwent dosage of plasmatic cytokine showing elevated levels of TNFα (104 pg/ml) and IL1β (438 pg/ml). Therefore, Anakinra therapy was started with clinical benefits and reduced levels of TNFα (6.9 pg/ml) and IL1β (2.2 pg/ml).

**Conclusions:** Literature reports that defects in NEMO cause various abnormalities in signal transduction pathways involving NF-kB, TNF signalling and the IL-1 family protein receptors. Moreover, the association of NEMO C-terminal truncation with inflammatory disease, resulting in enhanced stimulation-induced proinflammatory gene expression and cytokine production, has been described recently.1 Our patient presents a mutation of NEMO gene involving C-terminal domain, which could be involved in the autoinflammatory manifestations as well as in the response to anti-IL-1 therapy. Informed consent to publish has been obtained from the parent.

1. Zilberman-Rudenko J, Shawver LM, Wessel AW et al. Recruitment of A20 by the C-terminal domain of NEMO suppresses NF κB activation and autoinflammatory disease. Proc Natl Acad Sci USA. 2016;113(6):1612-7.

### P195 The phenotypic role of heterozygous mutations in familial mediterranean fever

#### Seda Şahin, Zeynep Birsin Özçakar, Nilgun Cakar, Semanur Ozdel, Fatos Yalcinkaya

##### Pediatric Rheumatology, Ankara University, Ankara, Turkey

###### **Correspondence:** Zeynep Birsin Özçakar

**Introduction:** Familial Mediterranean Fever (FMF) is an autosomal recessive disease, characterised by recurrent, self limited attacks of fever with serositis. The gene responsible for FMF, designated as *MEFV*, encodes pyrin. Approximately 15-30% of patients with FMF had heterozygous mutations.

**Objectives:** The aim of this study was to compare the demographic, clinical, laboratory features of FMF patients with heterozygous M694V mutations to those with other heterozygous mutations.

**Methods:** Files of FMF patients who had been seen in our department (during routine follow-up visits) between January 2013 and January 2014 were retrospectively evaluated. Patients with heterozygous mutations were included to the study. Six predominant mutations (p.M694V, p.M680I, p.M694I, p.V726A, p.K695R, p.E148Q) in the *MEFV* gene were studied in our center. Patients were divided into two groups: group I included patients with heterozygous M694V mutations and group II included patients with other heterozygous mutations.

**Results:** The study group comprised 82 FMF patients (42 females, 40 males) with a median age of 12.6 (1.5-24) years. There were 47 patients in group I and 35 patients in Group II. Age at disease onset, frequency of major clinical findings and family history of FMF did not differ between the two groups. However, arthralgia, leg pain, and heel pain were more frequently detected in group I patients (p < 0.05). Median attack and attack-free acute phase reactant levels before and after colchicine therapy, median PRAS severity score and final colchicine dosages did not differ between the two groups.

**Conclusion:** FMF patients with heterozygous M694V mutations have more musculoskeletal complaints during childhood period. It seems that M694V mutation is a susceptibility factor for these type of complaints

**Disclosure of Interest**

None Declared

### P196 Evaluation of high mobility group box 1 protein as an inflamation marker in patients with familial mediterranean fever

#### Betül Öztürk^1^, Esra Baskın^1^, Kaan Gülleroğlu^1^, Nilufer Bayraktar^2^, Feride Şahin^3^

##### ^1^Pediatric Rhematology, Başkent University, Ankara, Turkey; ^2^Biochemistry, Başkent University, Ankara, Turkey ^3^Genetics, Başkent University, Ankara, Turkey

###### **Correspondence:** Betül Öztürk

**Introduction:** Familial Mediterranean Fever (FMF) is an autoinflammatory disease that commonly presents with fever, peritonitis, pleuritis, synovitis, and less commonly with pericarditis. Several recent studies reported ongoing inflammation in the attack-free period of patients with FMF. Other studies revealed that some proteins show an inflammatory response after translocation to an extracellular environment through cellular damage. These proteins are known as Damage-Associated Molecular Patterns (DAMPs); they carry out daily physiological cell functions and induce a strong inflammatory response after translocation to an extracellular area after cell damage has occurred.

**Objectives:** High mobility group box protein (HMGB1) is a frequently-investigated DAMPs molecule, with a strong diagnostic and prognostic role for several chronic inflammatory diseases. Our aim was to discover HMGB1’s role in FMF.

**Methods:** Sixty consecutive patients with FMF that were followed in the Pediatric Nephrology department of our institute were included in this study. Sixty healthy children were also included as the control group. Demographic data of patients and their parents, including the patients’ genetic analyses, and demographic data of controls were recorded. Blood samples were obtained from patients and controls for HMGB1 analysis. Laboratory analysis of patients included CRP, sedimentation, blood count, fibrinogen, creatine kinase, AST, ALT, creatinine, and microalbumin/creatinine from urine samples were recorded as well.

**Results:** Fifty-seven patients with FMF (27 female/32 male) and 60 healthy children (30 female/30 male) were included in this study. The median age of patients and healthy controls was 123 months (min-max: 20-220). Thirty-three patients (57.9%) in the FMF group had a history of disease in their family, while there were consanguineous marriages in 6 of the patients’ parents (10.5%). Furthermore, 65% of patients came from the west Blacksea region and west-middle Anatolian region. The median follow-up of patients with FMF diagnosis was 5 years (min-max: 1-12 years). The most frequent patient mutation was M694V (79%). All patients were on colchicine treatment, with the median dose being 1 mg/day (min-max: 0.5 mg-1.5 mg). Age, sex, and body weight were similar among both patients and controls. Patients’ HMGB1 levels were significantly higher when compared to controls (*p* = 0.001). There was a significant positive correlation between HMGB1 and red cell distribution width (RDW), including neutrophil to lymphocyte ratio. No significant relation was observed between patient mutations and HMGB1 and HMGB1 levels were similar during both the attack and the attack-free period of patients.

**Conclusion:** This study demonstrated that HMGB1 levels in patients with FMF are significantly higher compared to healthy controls, while these levels are not influenced by the period of the disease. Our study finds that subclinical inflammation persists in patients with FMF in the attack-free period. Further comprehensive studies, with a greater number of patients, are necessary to thoroughly examine this issue.

**Disclosure of Interest**

None Declared.

### P197 British paediatric surveillance unit (BPSU) study of Behcet’s syndrome in children & young people in the United Kingdom

#### Clare E. Pain^1^, Emma Latham^2^, Michael W. Beresford^2^, Paul Brogan^3^, Farida Fortune^4^, Robert J. Moots^2^, Ruth Murphy^5^

##### ^1^Alder Hey Children’s NHS Foundation Trust, Liverpool, UK; ^2^University of Liverpool, Liverpool, UK ^3^Great Ormond Street Hospital, London, UK ^4^Queen Mary’s School of Medicine & Dentistry, London, UK ^5^Royal Hallamshire Hospital, Sheffield, UK

###### **Correspondence:** Clare E. Pain

**Introduction:** Behçet’s syndrome is a rare multi-system inflammatory condition, prevalent in countries along the Silk Route. The burden of disease of children with Behçet’s in the UK has not been previously described.

**Objectives:** 1) describe clinical features, clinical care & outcomes of children with Behçet’s in the UK.

**Methods:** From May 2015 to May 2017, UK paediatricians & paediatric dermatologists received monthly email notification forms. Clinicians were asked to report any child under 16 years who had 2 or more of the following features not explained by an alternative diagnosis:recurrent oral aphthous ulceration; skin, eye, neurological or vascular involvement; positive pathergy; genital ulceration; family history of Behçet’s. Reporting clinicians were sent a questionnaire which was completed from case notes and returned to the study team for analysis.

**Results:** To March 2017 (23 months), 130 cases were notified & 61 completed questionnaires analysed (30 cases excluded; 16 errors & 14 duplications; 39 questionnaires to be completed). 46/61 analysed cases are confirmed Behçet’s syndrome, defined as International Criteria for Behçet’s Disease (ICBD) score of ≥4.^1^

The 46 children, 27 (59%) girls and 19 (41%) boys, confirmed as having Behçet’s have a wide array of clinical manifestations (Table 7). Mean age at diagnosis was 9.9 years (n = 41). Most patients were Caucasian (39; 84.8%). There is a family history of Behçet’s in a first degree relative in 13 cases (28.3%). 38 children (82.6%) were seen by three or more different specialties depending on disease manifestations. Only 4 (8.7%) were not on regular treatment. Whilst 3 (6.5%) were managed on topical treatment only, 38 (82.6%) required systemic immunosuppression.

**Conclusion:** This study highlights the extreme rareness of Behçet’s syndrome in UK children. The commonest sub-type was the mucocutaneous variant. There may be important differences in patterns of disease between UK and non-UK cohorts, for example a low frequency of ocular involvement. Children have complex care needs as shown by the high number of specialists involved. ^*1*^ *The International Criteria for Behçet’s Disease (ICBD). J Eur Acad Dermatol Venereol. 2014. 28: 338–347.*

**Disclosure of Interest**

None Declared

**Table 7 (Abstract P197).** Disease features and outcome (n = 46). Numbers in brackets percentages

DISEASE FEATURES	Oral ulceration	46(100)
	Genital ulceration	42(91.3)
	Skin involvement	19(41.3)
	- Pseudofolliculitis	9(19.6)
	- Erythema nodosum	6(13.0)
	- Skin ulcers	3(6.5)
	Eye involvement	9(19.6)
	- Anterior uveitis	4(8.7)
	- Intermediate uveitis	2(4.3)
	- Pan-uveitis	1(2.2)
	- Retinal vasculitis	1(2.2)
	Neurological*	4(8.7)
	Vascular^#^	3(6.5)
	Other	
	- Arthralgia	5(10.9)
	- Abdominal pain/diarrhoea	5(10.9)
	- Arthritis	4(8.7)
	- Fever	1(2.2)
OUTCOMES (Not known in 3)	Controlled on medication	29(63.0)
	Active disease on medication	10(21.7)
	Stable off medication	4(8.7)

*headaches, deafness, central venous thrombosis; ^#^leucocytoclastic vasculitis, arterial thrombosis

.

### P198 Investigating the clinical and genetic profil in a cohort of patients with familial mediterranean fever

#### Efimia Papadopoulou-Alataki^1^, Olga Vamperzi^1^, Christina Trakatelli^2^, Sokratis Katafigiotis^3^, Vasiliki Galanopoulou^4^, Anastasia Alataki^3^, Alexandros Lambropoulos^3^

##### ^1^Fourth Department of Pediatrics, Aristotle University of Thessaloniki, Greece, Thessaloniki, Greece; ^2^Third Department of Internal Medicine, Aristotle University of Thessaloniki, Greece, Thessaloniki, Greece; ^3^Molecular Biology Lab, Aristotle University of Thessaloniki, Greece, Thessaloniki, Greece; ^4^Rheumatology Unit, Papageorgiou Hospital, Thessaloniki, Greece

###### **Correspondence:** Efimia Papadopoulou-Alataki

**Introduction:** Familial Mediterranean Fever (FMF) is an autoinflammatory periodic disease, inherited in an autosomal recessive manner, characterized by recurrent febrile episodes and sterile serosal inflammation attacks.

**Objectives:** To evaluate the clinical spectrum along with FMF gene (MEFV) mutations and to investigate the potential association between genotype-phenotype.

**Methods:** We studied 62 FMF patients attended in our department for a period of 7 years (2010-2017) who fulfilled the diagnostic Tel Hashomer criteria. Demographic features, clinical and laboratory findings, MEFV mutations and evaluation of treatment were recorded. Patients were divided into two groups according to their age: Group A: >2 - ≤ 18 years old and Group B: ≥18 - <35 years old. All patients were screened for the 12 most common MEFV mutations by reverse hybridization assay (E148Q, P369S, F479L, M680I(G/C), M680I(G/A), I692del, M694V, M694I, K695R, V726A, A744S, R761H).

**Results:** A total of 62 FMF patients, 34 female (54.8%) and 28 male (45.2%) were included in the study: Group A: 30 (♀/♂:14/16) and Group B:32 (♀/♂:20/12). The mean age at the time of investigation was 9.7 ± 3.6 and 30.4 ± 4.1 years while the mean age at disease onset was 3.08 ± 2.3 and 13.3 ± 7.3 years for Group A and Group B, respectively. The mean delay of diagnosis was 1.8 ± 1 years in children whereas 11.12 ± 7.33 years in adults (significantly higher in adults, p < 0.001). All patients presented recurrent febrile episodes along with abdominal pain before treatment. The mean number of episodes per year was 11 ± 4 and 9 ± 4 while the mean duration of each episode was 3 ± 1 and 4 ± 1.5 days for Group A and Group B, respectively. Chest pain was present in 46% and musculoskeletal pain in 73% of children whilst even more in adults (78.1% suffered from chest pain and 87.5% from musculoskeletal pain).We detected mutant gene in all 62 patients. Only 8 out of the 12 MEFV mutations tested were detected (E148Q, P369S, M680I(G/C), M694V, K695R, V726A, A744S, R761H). In general, M694V was the most frequent mutation [(64.5%), 56.6% in Group A and 71.8% in Group B] followed by M680I(G/C) [(22.6%), 13.3% in Group A and 31.2% in Group B] and E148Q [(19.3%), 26.6 in Group A and 12.5% in Group B]. Overall, 39 patients (62.9%) had heterozygote mutation for a single gene, 17 (27.4%) had compound heterozygote mutations and 6(9.7%) had homozygote mutation. M694V homozygosity was seen in 2 children and 2 adults of younger age at disease onset who presented severe clinical features. M694V mutant allele was found in compound heterozygosity in 15 patients. In 3 of those, the presence of M694V/M680I(G/C) mutation was associated with severe outcome: 1child (♀,11years of age) presented persistent proteinuria and 2 adults (♂, 20 and 28 years of age) renal amyloidosis. All FMF patients were under maintenance therapy with Colchicine. Canakinumab was added in 1 child and 2 adults and Tocilizumab in 1 adult (all females with M694V mutation). Renal transplant has been offered to the 20-year-old male with amyloidosis.

**Conclusion:** M694V mutation is the most common mutation followed by M680I(G/C) and E148Q. M694 mutation is shown to be associated with younger age at disease onset and severe clinical outcome. Chest pain and musculoskeletal pain were very frequent clinical features. Delay for diagnosis was found to be shorter in children compared to adults, possibly due to increase of awareness for FMF in recent years.

**Disclosure of Interest**

None Declared.

### P199 CXCL10 and CXCL9 genetic variants in pediatric patients with periodic fever syndrome with aphthous stomatitis, pharyngitis, and adenitis (PFAPA)

#### Daša Perko^1^, Maruša Debeljak^2^, Nataša Toplak^1,3^, Tadej Avčin^1,3^

##### ^1^Department of Allergology, Rheumatology and Clinical Immunology, University Children’s Hospital, UMC Ljubljana, Ljubljana, Slovenia; ^2^Center for Medical Genetics, University Children’s Hospital, UMC Ljubljana, Ljubljana, Slovenia; ^3^Faculty of Medicine, University of Ljubljana, Ljubljana, Slovenia

###### **Correspondence:** Daša Perko

**Introduction:** Periodic fever syndrome with aphthous stomatitis, pharyngitis, and adenitis (PFAPA) represents the most common periodic fever syndrome of childhood with unknown etiology. PFAPA is considered as a sporadic disease although many studies suggest that it might have a genetic cause especially due to the high rate of positive family history. However, no genetic cause has been identified. Studies show that serum levels of chemokines, immune cells chemo-attractants, CXCL10 and CXCL9 are significantly increased in PFAPA patients during febrile state compared to PFAPA patients in afebrile state, patients with other hereditary periodic fever syndrome during flare and healthy controls. Furthermore, CXCL9 is increased in PFAPA patients during afebrile state compared to healthy controls.

**Objectives:** The aim of our study was to determine whether genetic variants in *CXCL10* gene and *CXCL9* gene are involved in PFAPA pathogenesis.

**Methods:** We performed genetic testing on children, diagnosed with PFAPA syndrome, who were followed at the University Children’s Hospital, Ljubljana, from January 2008 to June 2014. All 4 coding exons, intron/exon regions, 5′UTR, part of 3′UTR and promoter region (600 base pairs upstream of the start site) of *CXCL10* gene and *CXCL9* gene were PCR amplified and directly sequenced.

**Results:** Genetic analysis was performed in 62 patients. 39 (63%) patients were male and 23 (37%) were female. Mean age at the syndrome onset was 2.1 years and at diagnosis 4.2 years. Variants found in *CXCL10* gene and *CXCL9* genes are listed in Table 8.

Variant nomenclature based on NCBI reference sequences NM_001565.3 (*CXCL10*) and NM_002416.2 (*CXCL9*); MAF: minor allele frequency; MAF EU and global MAF-1000 Genomes data or Exac

**Conclusion:** Variant c.-201G > A in *CXCL10* gene is listed in Human Gene Mutation Database (HGMD) as a disease-associated polymorphism. Functional analyses in one study showed that the this variant alters the binding affinity of nuclear protein and regulates CXCL10 expression. Therefore it could have a minor impact on autoinflammatory process in PFAPA syndrome. Other variants found are polymorphism with probably no clinical significance

**Disclosure of Interest**

None Declared.

**Table 8 (Abstract P199).** See text for description

Gene	Variant	rs number	No of patients (het.)	No of patients (hom.)	MAF EU/MAF global/MAF patients
***CXCL10***	c.-201G > A	rs56061981	8	1	0,03/0,11/**0,08**
	c.61 + 125_61 + 128delAATA	rs146674736	2	0	0,04/0,01/**0,02**
	c.61 + 33A > G	rs4241578	34	21	0,54/0,61/**0,61**
	c.279-36C > G	rs4859584	25	30	0,48/0,6/**0,69**
	c.*140G > C	rs3921	25	30	0,49/0,31/**0,69**
	c.*321delC	rs34836828	25	30	0,49/0,31/**0,69**
***CXCL9***	c.-127T > C	rs2276885	23	10	0,16/0,23/**0,35**
	c.*94G > C	rs115116604	3	0	0,02/0,01/**0,02**
	c.*180A > G	rs10031051	3	0	0,02/0,04/**0,02**

### P200 Clinical and histological presentation of the initial skin involvement in blau syndrome

#### Julie Poline^1,2^, Olivier Fogel^3^, Corinne Miceli-Richard^3^, Carine Wouters^4^, Marie Dominique Vignon-Pennamen^5^, Eric Hachulla^6^, Ulrich Meinzer^1,2^, Emmanuelle Bourrat^1,7^

##### ^1^Department of General Paediatrics, Infectious Diseases and Paediatric Internal Medicine, Robert Debré University Hospital, Paris, France; ^2^INSERM U1149, Inflammation Research Centre, Paris, France; ^3^Department of Rheumatology, Cochin University Hospital, Paris, France; ^4^Department of Microbiology and Immunology, Pediatric Immunology, KU Leuven, University of Leuven, Leuven, Belgium; ^5^Departments of Pathology and Dermatology, Saint Louis University Hospital, Paris, France; ^6^Department of Internal Medicine, Huriez Hospital, University of Lille, Lille, France; ^7^Department of Dermatology, Reference centre of genetic cutaneous diseases MAGEC, Saint Louis University Hospital, Paris, France

###### **Correspondence:** Julie Poline

**Introduction:** Blau syndrome (BS) is a very rare monogenic disease resulting from mutations in the pattern recognition receptor NOD2. It is phenotypically characterized by the triad of granulomatous polyarthritis, uveitis and skin involvement. Although skin involvement occurs in 70% of cases, only very little is known about its initial clinical presentation and evolution during disease course.

**Objectives:** To describe the initial skin manifestations of BS.

**Methods:** We retrospectively studied six patients from three families with a genetically confirmed BS. Data on the dermatological manifestations were collected by analysis of photographs, medical records and patient interviews.

**Results:** The median age at diagnosis was 16.5 years. In all patients skin manifestations were the first symptom of the disease and occurred at a median age of 1.6 years. For 3 patients the rash set on following BCG-vaccination. Skin lesions were always diffuse millimetric micropapules with normal skin colour or erythematous or pigmented without itching. The skin lesions were diagnosed as Gianotti-Crosti syndrome or atopic dermatitis before the diagnosis of BS was established. Skin lesions disappeared in three patients, one had a flare after BCG and one had a persistent rash. All skin biopsies revealed dermic granuloma without caseous necrosis. Infectious agents stains were negative.

**Conclusion:** The initial skin lesions of BS occur in early childhood and are characterized by a stereotypical transient rash made of monomorphic micropapules with either normal skin colour or erythema. It is often unrecognized or considered as a nonspecific dermatitis. The better knowledge of inaugural skin manifestations may allow earlier diagnosis.

**Disclosure of Interest**

None Declared.

### P201 Successful use of tofacitinib in a 6-year-old patient with SAVI syndrome

#### Svetlana Rodionovskaya^1,2^, Svetlana Zaytseva^1^, Svetlana Salugina^2^, Evgenie Fedorov^2^, Irina Tsymbal^1^

##### ^1^Pediatric rheumatology, Central Children’s Clinical Hospital of the Federal Medical-Biological Agency of Russia, Moscow, Russian Federation ^2^Pediatric rheumatology, V.A.Nasonova Research Institute of Rheumatology, Moscow, Russian Federation

###### **Correspondence:** Svetlana Rodionovskaya

**Introduction:** SAVI syndrome (STING – associated vasculopathy with onset in infancy) is an autoinflammatory syndrome, associated with TMEM17 mutation. Its manifestations include the complex of symptoms of vasculitis and pulmonary involvement similar in clinical presentation to Juvenile polyarteritis.

**Objectives:** The description of clinical observation of a patient with SAVI syndrome, severe interstitial lung disease.

**Methods:** The description of clinical observation of a patient with SAVI syndrome, severe interstitial lung disease.

**Results:** We observe a 6-year-old girl. Onset of the syndrome in the age of 1: papular rash on a face and external ears followed by scars; recurrent pyretic fever. Deterioration in the condition at the age of 3: urticaria, fever with coughing and dyspnea attacks, stomatitis, conjunctivitis, stomach pain, arthralgia, Raynaud’s syndrome, WBC 13 × 10^9^, ESR 35 mm/h, CRP 40 mg/l. CRP and ESR values were within normal range in-between fever attacks. In course of 3 years the patient received budesonide for bronchial asthma which gave no effect. Bronchial asthma, mucoviscidosis, primary immunodeficiency conditions, infections, connective tissues systemic diseases, ANCA vasculitis, CAPS, TRAPS, HIDS were ruled out at the age of 5. The multi-layer spiral CT of lungs demonstrated interstitial alterations of S2 superior lobe of the right lung, S6 left lower lobe, with negative dynamics within 6 months of follow-up. SAVI syndrome was diagnosed. TMEM 173 mutation was not confirmed. The prescription included prednisolone 0.7 mg/kg/day, tofacitinib 7,5 mg/day. Fever, cough, dyspnea, Raynaud’s syndrome, arthralgia reserved after three months of treatment, the severity of rash reduced, the levels of CRP, ESR, PLT and WBC normalized. Prednisolone was cancelled after 8 months, tofacitinib treatment was continued. After 12 months of treatment positive dynamics was observed in the multi-layer spiral CT of lungs, i.e. reduction of alterations intensity in S2 superior lobe of the right lung, as well as in the area of S6,8,9 left lower lobe. No formation of bronchi and bronchiole ectasis, honeycomb lung areas was observed.

**Conclusion:** In the framework of our observation Tofacitinib treatment in SAVI syndrome patients effectively controls interstitial alterations and clinical manifestations of the disease. Safety profile is satisfactory.

**Disclosure of Interest**

None Declared.

### P202 Monogenic autoinflammatory diseases in the practice of a rheumatologist in Russia

#### Svetlana O. Salugina^1,^, Evgeny S. Fedorov^1^, Nina N. Kuzmina^1^, Elena A. Kamenets^2^, Ekaterina U. Zaharova^2^

##### ^1^Pediatrics, V.A.Nasonova Research Institute of Rheumatology, Moscow, Russian Federation; ^2^Hereditary metabolic disorders, FSBI «Research Centre for Medical Genetics», Moscow, Russian Federation

###### **Correspondence:** Svetlana O. Salugina

**Introduction:** Autoinflammatory diseases (AIDs) are characterized by periodic, occasionally self-limited attacks manifested by fever and clinical symptoms similar to rheumatic presentation without autoimmune or infectious causes. Currently, AIDs are conditionally divided into monogenic (caused by known genetic mutation) and multifactorial (polygenic). In recent years, great attention has been paid to the studies of main monogenic AIDs (mAIDs) such as Familial Mediterranean fever (FMF), Cryopyrin associated periodic syndromes (CAPS), Hyper-Immunoglobulinemia D/Mevalonate Kinasae Deficiency syndrome (HIDS/MKD), TNF-receptor-associated periodic syndrome (TRAPS). In recent years, rheumatologists have had to deal with these diseases with increasing frequency. Targeted therapy with IL-1 inhibitors has been developed and successfully used.

**Objectives:** To identify and characterize the most frequent mAIDs in the practice of pediatric rheumatologist according to the frequency of visiting Russian Federal Rheumatology Center.

**Methods:** The study included patients with fever and other signs of systemic inflammatory process after exclusion of infections, hemato-oncological and other causes who referred to V.A. Nasonova Research Institute of Rheumatology from 2007 to 2016 to clarify the diagnosis. All these patients passed the examination generally accepted in rheumatology including molecular genetic tests of MEFV, NLRP3, MVK and TNFRSF1A genes.

**Results:** Over 10 years, AIDs were diagnosed in 133 patients aged from 6.5 months to 60 years. 57 patients (42.9%) had mAIDs. The following diseases were diagnosed: FMF in 23 patients (9 men and 14 women); CAPS in 20 patients (8 men and 12 women); Muckle-Wells syndrome (MWS) in 17 patients (5 men and 12 women); CINCA/NOMID in 3 patients (3 men/0 women); HIDS/MKD in 3 patients (0 men/3 women); TRAPS in 11 patients (5 men and 6 women). 13 familial cases were detected: FMF in 7 patients, MWS in 4 patients and TRAPS in 2 patients. Most patients with FMF were Armenian (78,3%). Patients with other diagnoses (ICD codes) had mostly Russian ethnic origin. The diagnosis of mAID was confirmed by the detection of pathogenic mutation in almost every patient. Only in one patient with CINCA/NOMID genetic mutation was not found and the diagnosis was based on clinical data. 27 patients received biological therapy: TNF inhibitors were used in 3 patients with FMF, Tocilizumab in 2 patients with CAPS, 2 patients with TRAPS and 1 patient with HIDS. 13 patients (8 children and 5 adults) received IL-1 inhibitor Canakinumab (CAPS-9, TRAPS-4). Positive effect and satisfactory tolerability were observed in all these patients.

**Conclusion:** Monogenic AIDs were observed in about a half of all cases of AIDs encountered in our practice. FMF, CAPS and TRAPS were the most frequent diseases and HIDS was much more rare. To rule out a differential diagnosis, it should be taken into account that CAPS is mainly similar to systemic juvenile arthritis (sJA) by clinical and laboratory findings.Our experience has shown high efficacy of targeted therapy with IL-1 inhibitors in patients with CAPS and TRAPS. This treatment allows to completely control the disease activity and to significantly improve survival of patients with originally poor prognosis.

**Disclosure of Interest**

None Declared.

### P203 Characteristic of the familial mediterranean fever patients presented with musculoskeletal findings

#### Betul Sozeri^1^, Duygu Kurtulus^2^, Tugba Demircan Bilen^3^, Gulsah Senay Karagoz^3^, Ismail Islek^3^

##### ^1^Pediatric Rheumatology, Health Sciences University, Umraniye Training and Research Hospital, Istanbul, Turkey; ^2^Physical Medicine and Rehabilitation, Health Sciences University, Umraniye Training and Research Hospital, Istanbul, Turkey; ^3^Health Sciences University, Umraniye Training and Research Hospital, Istanbul, Turkey

###### **Correspondence:** Betul Sozeri

**Introduction:** Familial Mediterranean fever (FMF) is an autosomal recessive disorder characterized by recurrent attacks of febrile peritonitis, pleuritis and synovitis. Articular involvement is one of the common and important features of FMF. The most common type of arthritis is recurrent, self-limited and short-lived acute inflammation of the joints. The frequency of arthritis in FMF has been reported to range from 21 to 77% in different ethnic groups.

**Objectives:** In this study, we aim to demonstrate clinical features of FMF patients with musculoskeletal findings in our cohorts

**Methods:** We retrospectively reviewed the records of 194 Turkish unrelated FMF patients diagnosed at the Pediatric Rheumatology Unit, between June and December 2016. Disease activity score was measure the Auto-Inflammatory Diseases Activity Index (AIDAI).

**Results:** Musculoskeletal findings (arthralgia, arthritis, myalgia and enthesopathy) were observed in 87 (44.8%) of 194 FMF patients. Among the 87 patients, 42 (48%) were males. The mean age of onset of FMF in our patients was 58.6 ± 46.1months. In 165 (85%) patients, FMF had started before the age of 10 years, and in 29 (15%) FMF started between the age of 10 and 17 years. The mean age of onset disease in patients with musculoskeletal findings was older than other patients (65.8 ± 50.9 vs 52.7 ± 41.1 months, p = 0.048).

There were 63 patients had arthralgia of 87 (72.4%) who had musculoskeletal findings, 17 (19.5%) had peripheral arthritis, 6 (0.7%) isolated sacroiliitis and 2 had enthesopathy.

Arthritis was monoarticular in 20 (83%) patients, symmetric two-joint arthritis in 4 (17%). Knees were affected in 13 (56.5%) patients, ankles in 3 (13%), hips in 2 (0.9%), and sacroiliac in 5 (21%). However, musculuskletal findings were the only manifestation of FMF in 25 (13%) patients.

Homozygote mutation in the MEFV gene was identified in 15 (17.2%) patients and the heterozygote mutation detection was identified in 72 (82.8%) patients with musculoskeletal findings. The most dominant mutations detected were M694V (54%) and P369S (16%).

The frequency of attack and disease score were higher in the patients with musculoskeletal findings (1.67 ± 1.21 vs1.54 ± 1.08, P = 0.51 and 9.4 ± 6.84 vs 7.76 ± 6.12, p = 0.12).

**Conclusion:** The musculoskeletal pathologies of FMF are clinically presented in various forms. M694V mutation is most likely associated with arthritis as one of the severe complications of FMF. In endemic area, all arthritis patients should be evaluated for FMF in order to prevent the development of chronic arthritis and amyloidosis.

**Disclosure of Interest**

None Declared.

### P204 Children with autoinflammatory diseases in British Columbia: years of delay before diagnosis

#### Lori Tucker, Jennifer Tekano, David Cabral, Jaime Guzman, Kimberly Morishita, Kristin Houghton, Kelly Brown

##### Pediatric Rheumatology, BC Children’s Hospital, Vancouver, Canada

###### **Correspondence:** Lori Tucker

**Introduction:** Auto-inflammatory diseases (AInD)often first present in childhood, and the protean manifestations of fever, rash and other inflammatory symptoms can be confused with common childhood illness. Disease recognition and correct diagnosis may be delayed due to low recognition of AInD among community health care providers.

**Objectives:** We examined the time interval from parent-reported symptom onset to diagnosis of AInD for children seen at British Columbia’s Children’s Hospital (BCCH), Canada in the first year of a newly dedicated provincial tertiary-care AInD clinic.

**Methods:** Consecutive patients seen in the AInD clinic were consented to participate in a longitudinal patient registry based at BCCH called CAN-Fever. Subjects with completed registry enrollment data were analysed, with data including demographics, diagnosis, time intervals from symptom onset (parent report) to diagnosis and from diagnosis to registry enrollment.

**Results:** 48 patients (M:F 24:24, mean age at enrollment 8.9 y, range 1-19 y) were enrolled into CAN-Fever over 12 months in 2016. Unclassified periodic fever syndrome (n = 13) and periodic fever, aphthous stomatitis, pharyngitis and adenopathy (PFAPA) syndrome (n = 13) were the most common diagnoses; smaller numbers of patients were seen with the following: chronic recurrent multifocal osteomyelitis (9), familial Mediterranean fever (5), TNF Receptor Associated Periodic Syndrome (3), Behcet syndrome (3), Cryopyrin-Associated Periodic Syndrome (1), Mevalonate Kinase Deficiency (1). Median time from parent-reported symptom onset to diagnosis was 1.9 y (IQR 1.0, 3.6 ; range 0.4-10.6) for children with any periodic fever syndrome, compared with 1.8 y (IQR 0.7,3.2; range 0.5-5.3) for children with CRMO. There were no differences in time from symptom onset to diagnosis between different periodic fever categories. Four children with periodic fever syndromes (2 PFAPA, 2 unclassified) were not diagnosed for 8 years or more. Mean time from AInD diagnosis to study enrollment was 1.7 y (IQR 0.4, 5.1; range 0-16.5 y)

**Conclusion:** We identified that many children with AInD in BC did not recieve a diagnosis for years. Poor recognition of AInD in the general medical community and previous lack of a dedicated expert clinical centre may be contributing factors.

**Disclosure of Interest**

None Declared

## Disease outcome

### P205 Disorders of respiratory organs in children with rheumatic diseases

#### Olena A. Oshlyanska

##### Department of Connective Tissue Disorders in Children, State Institute of Pediatrics, Obstetrics and Gynecology, Academy of Medical Sciences of Ukraine, Kyiv, Ukraine

**Introduction:** Rheumatic diseases are a multi-organ pathology, in which the defeat of respiratory organs occupies an important place among target organs. In adult patients at instrumental inspection their frequency reaches 65-80% depending on a nosological unit.

**Objectives:** Analyze the frequency of clinical and instrumental manifestations of lung lesions in children with rheumatic diseases.

**Methods:** A retrospective analysis of the data of clinical register of children’s connective tissue diseases department: 253 children with rheumatic diseases (145 - JIA, 35 SSD, 32 JDM, 24 SLE, 11 MCTD, 8 SV).

**Results:** Clinical manifestations of respiratory injury were observed in 18.6% of children with JIA, 11.4% in SSD, 25.8% in JDM, 45.8% in SLE, 18.18% in MCTD (mixed connective tissue disease and overlap-syndrome), 50% SV. According to instrumental studies, lung lesions were more common: in 33.1; 91.4; 90.6; 62.5; 36.4; 50% respectively. It is unreliable that the respiratory organs were more often affected with a high degree of activity. Clinical manifestations of bronchial obstructive syndrome were found in 33.3% of children with SSD, 18.1% of MCTD and 25% of SW (1 patient with Charg-Strauss syndrome - bronchial asthma), according to spirography bronchial obstruction: 3.1% SCR, 50% SSD, CB and MCTD with the same frequency as clinical manifestations. 6.2% of children with SJUIA had pneumonitis. Clinical manifestations of persistent restrictive disorders (dyspnoea under physical exertion, cough, cyanosis) were noted in 3.1% of SLE patients, 4.1% SDS, 9.1% MCTD, auscultatory changes (migrating crepitating wheezing) were detected in 1 patient with SCR and YDM and 7.2% of the SSD. Exudative pleurisy was noted in 10.7% of patients with SLE, dry pleurisy in 14.3%, 1 case - fibrosing alveolitis with SLE, severe destructive changes in lungs - in 6 patients with JDM and SV, pulmonary hypertension in 2 children (SSD and overlap- syndrome), pulmonary tuberculosis - 1 case in a child with a JIA who received an anti-TNF drug.

Chest radiographs with JIA in 14.4% show pleural adhesions, in 33.1% of patients - strengthening pulmonary pattern. Basal pneumofibrosis in more than 10 years of observation was in 33% of patients with JDM, 17.8% of SLE, 100% of SSD and MCTD. Also, 88.8% of patients with JDM had infectious complications during the entire follow-up period, while among other patients - in 29%. In 3 patients receiving methotrexate drug-caused pulmonitis was observed.

**Conclusion:** The defeat of respiratory organs in children with RD is common. Due to the non-specificity of clinical manifestations, their differential diagnosis is not paid attention. The frequency of clinical manifestations in children is less frequent than in adults, according to the data of instrumental studies, its values are comparable, which indicates the possibility of prolonged subclinical course and the need for monitoring.

**Disclosure of Interest**

None Declared.

### P206 Features of the liver functional status in children with juvenile rheumatoid arthritis and systemic lupus erythematosus

#### Nataly S. Shevchenko, Ljudmila F. Bogmat, Irina N. Bessonova

##### Department of Cardiorheumatology, Institute of Children and Adolescents Health Care, Kharkiv, Ukraine

###### **Correspondence:** Nataly S. Shevchenko

**Introduction:** Problematic issues of modern rheumatology concern not only clinical diagnostic and therapeutic issues of major and serious in its outlook and consequences of diseases. In recent years, much attention is paid to pathological conditions that are attributed to comorbid arising from the disease or the treatment used. These include modern researchers the least attention is paid to non-specific liver.

**Objectives:** The aim of the investigation was to study the functional state of the liver in children with systemic lupus erythematosus and juvenile rheumatoid arthritis during the administration of иasic and anti-inflammatory medications.

**Methods:** It was analyzed the history of 26 persons 7-18 years with systemic lupus erythematosus (SLE) and 70 patients with juvenile rheumatoid arthritis (JRA) aged 7 -18 years of the duration of the underlying disease for more than three years. The functional state of the liver in children and adolescents had been studied using complex clinical laboratory, biochemical and instrumental methods (objective examination, ultrasound (US) of the liver and gallbladder, determine blood levels of bilirubin and its fractions, apolipoprotein, haptoglobin, activity of alanine transferase (ALT), aspartate transferase (AST) and γ-glutamate transferase (GGT).

**Results:** Symptoms of liver involvement in the pathological process of increasing body size by ultrasound found in 60.0% of patients with SLE and 10.7% - with JRA. Analysis of pigment metabolism SLE patients testified to the adequacy of the liver detoxification function, but there were growth in the average total bilirubin (p <0.05), while maintaining disease activity (r = 0,63; p <0.05). High concentrations of ACT registered 12.5% in SLE and 5.0% - with JRA. The haptoglobin concentration among patients in both groups decreased significantly (p <0.05), which reflects a decrease of the protein-synthesizing liver function in patients with prolonged disease, and high level process activity. This confirms the relationship between the degree of disease activity and the level of haptoglobin in the blood of children with JRA (r = - 0,96; p <0.05). Research excretory liver function by identifying one of the most sensitive markers of cholestasis - GGT serum showed that the concentration was elevated 40.0% of SLE patients. Half of patients with SLE occurres hyperinsulinemia (increased levels of over 25 mkMO/ml). It is a direct connection between the degree of disease activity and the level of serum apolipoprotein (r = 0,895; p < 0.05), ratio of ALT/RYR (r = 0,999; p <0.05), that is indicating the accumulation of signs of fibrosis formation in SLE.

**Conclusion:** So, the high frequency of liver involvement in the pathological process in SLE and JRA with a reduction of protein synthetic and excretory functions during long-term disease was found. The functional status of the liver are more depended on the activity of the disease.

**Disclosure of Interest**

None Declared.

### P207 Investigation of the function of external respiration in children with rheumatic diseases

#### Nataly S. Shevchenko^1^, Ljudmila F. Bogmat^1^, Marina V. Demjanenko^1^, Inna D. Savvo^2^

##### ^1^Department of Cardiorheumatology, Institute of Children and Adolescents Health Care, Kharkiv, Ukraine; ^2^Department of pediatrics, V.N.Karazin Kharkov National University, Kharkiv, Ukraine

###### **Correspondence:** Nataly S. Shevchenko

**Introduction:** Formation of the comorbid conditions in the rheumatic diseases determines the outcomes and long-term prognosis of these diseases. Often, this problem concerns occurrence in rheumatic patients with atherosclerosis, osteoporosis, diabetes, infectious complications. However, systemic inflammation, which base on the vasculitis, may be the cause of the other lesions formation, including the lung tissue damage. From another side, the destruction of the respiratory system can be caused by long-term treatment with cytotoxic basic agents. These issues have been studied mainly in adult patients.

**Objectives:** The aim of the study was to examine the condition of external respiratory function in children with systemic lupus erythematosus (SLE) and juvenile rheumatoid arthritis (JRA).

**Methods:** An examination of respiratory function in the children with JRA (17 patients) and SLE (11 patients) had done by the diagnostic spirometry and determination of the main indicators of respiratory function (vital capacity, functional vital capacity, forced expiratory volume in one second and its relation to the lungs (Tiffno Index vital capacity, minute ventilation). Duration of the disease in all patients was more than one year after diagnosis and initiation of therapy.

**Results:** The high rate of the respiratory function changes had been indicated (78.57% of all patients, 81.82% of SLE patients and 76.47% of patients with JRA). The restrictive violations regardless had been dominated among the pathological variants of the spirometry results. Patients who had obstructive-restrictive version of the external breathing (5.88%) included only children with JRA. The research has shown a significant increasing of the incidence of the pathological conditions after three years of disease duration, predominantly among patients with JIA (p <0.01).

Restrictive-obstructive variant of the external respiration was in patients with disease duration more than ten years. The high degree of restriction had been often determined in patients with JRA, the degree of restriction depended on the activity of the inflammatory process (r = 0,57; p <0.05).

**Conclusion:** Thus, the external respiration function revealed a high frequency of deviations from the normal values respectively targeted research. Restrictive disorders (decrease in respiratory surface of the alveoli) dominate and the frequency and severity of them increase with the diseases duration and depend on its activity. The restrictive-obstructive disorders have defined already in childhood, that requires the additional control of the efficacy and safety of the basic therapy.

**Disclosure of Interest**

None Declared.

### P208 Changes of the cardiovascular system in the structure of the comorbidity in children and adolescents with rheumatic diseases

#### Ljudmila F. Bogmat , Nataly S. Shevchenko, Irina N. Bessonova, Victoria V. Nikonova, Marina V. Demyanenko

##### Department of Cardiorheumatology, Institute of Children and Adolescents Health Care, Kharkiv, Ukraine

###### **Correspondence:** Nataly S. Shevchenko

**Introduction:** In the past few years much attention is focused on research of the mutual influence and reinforcement between the various diseases and pathological conditions in one patient. This is important for an individual approach and requires a comprehensive study of basic clinical picture, concomitant illness, their comprehensive evaluation and management of simultaneous treatment.

This problem becomes relevant in rheumatic diseases because it is a result of immunoinflammatory cascade of reactions and the development of systemic lesions endothelium, causing not only the clinical manifestations of the primary process, but also leads to the defeat of vital organs and systems and the development of metabolic disorders. Multi-organ lesions are associated with comorbidity, further aggravate the course of the underlying disease, its prognosis and complicate response to therapy, reduce the quality of life for patients.

**Objectives:** In order to improve the diagnosis of comorbid conditions in adolescents with the rheumatic diseases conducted the clinical and additional examination had done.

**Methods:** The results of the survey 26 persons 7-18 years with systemic lupus erythematosus (SLE) and 70 children of the same age, patients with juvenile rheumatoid arthritis (JRA) were studied. Investigation includes the study of the functional state of cardiovascular system (6-minute walk, ECG, ultrasound of the heart. Duration of the disease in all patients was more than one year after diagnosis and initiation of therapy.

**Results:** The high frequency of comorbid conditions in rheumatic diseases was detected in children (75.0%). It consist of pathology of the hepatobiliary system (in SLE - 50.0% and in JRA - 16.7%) and nervous system (68,0% and 16.7%), diffuse toxic goiter (12.5% and 7.7%), lesions of eye (cataracts in SLE - 16.7%, uveitis in JRA - 18.2%), respiratory pathology (81.5% and 72.5%) and kidneys (72.5% and 16.7%, respectively).

Changes in the cardiovascular system in children manifested by the violations of ECG parameters (tachycardia, increased systolic index), structural changes (the mitral prolapse from first to third degree) and disorders of the intracardiac hemodynamics and the cardiac remodeling. In children with SLE and JIA the impact and minute volumes of blood decrease, the size of the left atrium and end-diastolic left ventricular size increased according to terms of disease, indicating the formation of both diastolic dysfunction and systolic.

The decrease in adaptive capacity of the cardiovascular system in children with rheumatic diseases, according to test 6-minute walk, which manifested itself insufficient growth heart rate to exercise and decrease the distance that has been traveled (p <0.05).

The patients’ age, sex, duration of disease and its activity and duration of glucocorticoids treatment constituted fundamental relationship with the number of comorbid conditions in children with SLE. In children with JRA greatest weight in the formation of comorbidity among the studied parameters were: sex of the patient, the age of onset, number of affected joints, active process, duration of illness, duration of administration of glucocorticoids and methotrexate (p < 0,05).

**Conclusion:** The formation of comorbid conditions in patients with the rheumatic disease has already happened during the childhood. Their timely diagnosis is a valid reason for correction therapy to prevent the development of irreversible changes that can later cause persistent morphological and functional disorders of morbidity and mortality of patients.

**Disclosure of Interest**

None Declared.

### P209 Globalisation of paediatric musculoskeletal matters’ (PMM)

#### Nicola Smith^1^, Sharmila Jandial^2^, Ruth Wyllie^2^, Christine English^3^, Barbara Davies^3^, Raju Khubchandani^4^, Mercedes Chan^5^, Jane Munro^6^, Virginia Ferriani^7^, Claudia Saad Magalhães^8^, Ricardo Russo^9^, Jacqueline Yan^10^, Chris Scott^11^, Sirirat Charuvanij^12^, Khulood Khawaja^13^, Jelena Vojinovic^14^, Tim Rapley^15^, Helen Foster^1,2^

##### ^1^Institute of Cellular Medicine, Newcastle University, Newcastle Upon Tyne, UK; ^2^Paediatric Rheumatology, Great North Children’s Hospital, Newcastle Upon Tyne, UK; ^3^Department of Nursing, Midwifery and Health, Northumbria University, Newcastle Upon Tyne, UK; ^4^Department of Paediatrics, Jaslok Hospital and Research Center, Mumbai, India; ^5^Paediatric Rheumatology, University of Alberta, Edmonton, Canada; ^6^Paediatric Rheumatology, Royal Children’s Hospital, Victoria, Australia; ^7^Department of Paediatrics, Ribeirão Preto Medical School, University of São Paulo, Sao Paulo, Brazil; ^8^Department of Paediatrics, UNESP, Botucatu, Brazil; ^9^Service of Immunology/Rheumatology, Hospital de Pediatría, Garrahan, Argentina; ^10^Paediatric Rheumatology, Starship Children’s Health, Auckland, New Zealand; ^11^Department of Paediatrics, University of Cape Town, Cape Town, South Africa; ^12^Department of Paediatrics, Siriraj Hospital, Mahidol University, Bangkok, Thailand; ^13^Department of Immunology/Rheumatology, Al-Mafraq Hospital, Abu Dhabi, United Arab Emirates; ^14^Paediatric Rheumatology, Faculty of Medicine, University of Nis, Nis, Serbia; ^15^Institute of Health and Society, Newcastle University, Newcastle Upon Tyne, UK

###### **Correspondence:** Nicola Smith

**Introduction:** “paediatric musculoskeletal matters’ (PMM–www.pmmonline.org) is a free, evidence-based and peer reviewed open e-resource for paediatric musculoskeletal (MSK) medicine targeting non-MSK specialists. Since launch (Nov-2014) PMM has reached 183 countries with >57,000 users, >194,000 hits. Users who have declared their training background on the website are mainly non-MSK specialists. Feedback from users has requested further content to reflect international healthcare systems. PMM India was developed in collaboration with the Indian Academy of Paediatrics (IAP; Sept-2015, >2,200 users, 14,000 hits to date) and showcases successful partnership with local clinicians in developing PMM with local context.

**Objectives:** Further ‘internationalisation’ is now ongoing with additional global partners to develop ‘PMM International’. Here, we describe the process for international development.

**Methods:** Paediatric rheumatologists in countries around the world were approached to identify additional PMM content to reflect MSK medicine in their health care systems (e.g. case mix, clinical presentations, care pathways), with the focus on maintaining the level of knowledge relevant for non-MSK specialists. New content was developed by local teams identified by the paediatric rheumatologist(s) who then collated and provided expert overview before submission for editorial review. All contributions were provided in English. Additional cases and images were included with appropriate consent.

**Results:** PMM International additions to the original website brings new content predominately focused on infections/infection-related disease with MSK features or as differential diagnoses for rheumatic disease. Most content is in English with requests for translation of some content (e.g. pGALS which will be available in >7 languages). PMM International will be further peer reviewed with open access to all. A PMM app is planned to facilitate access where internet capacity is limited.

**Conclusion:** Rapid globalisation necessitates appropriate e-resources with content that reflect international health care contexts. PMM International targets non-MSK specialist audiences to raise awareness and early recognition of MSK pathology. Our work reflects strong collaborative global partnerships within the paediatric rheumatology community. PMM has been endorsed by PReS as an educational resource to aid teaching and learning. Implementation of PMM International has yet to be formally evaluated, but PMM data so far, supports wide reach and positive uptake from the target audience user groups.

**Disclosure of Interest**

None Declared.

### P210 Paediatric musculoskeletal (MSK) triage in the community – rightpath – a pilot study

#### Nicola Smith^1^, Sharmila Jandial^2^, Jill Firth^3^, Helen Light^3^, Katharine Kinsey^3^, Neil Snowden^3^, Judith McNaught^4^, Tim Rapley^5^, Alan Nye^3^, Helen Foster^1,2^

##### ^1^Institute of Cellular Medicine, Newcastle University, Newcastle Upon Tyne, UK; ^2^Paediatric Rheumatology, Great North Children’s Hospital, Newcastle Upon Tyne, UK; ^3^Pennine MSK Partnership Ltd, Oldham, UK; ^4^Physiotherapy, South Tyneside NHS Foundation Trust, South Shields, UK; ^5^Institute of Health & Society, Newcastle University, Newcastle Upon Tyne, UK

###### **Correspondence:** Nicola Smith

**Introduction:** We are piloting a children and young people (CYP) community-based triage (called Rightpath) based on a validated adult MSK triage model developed by Pennine MSK Partnership Ltd (PMSKP), Greater Manchester, i.e. triage by expert MSK nursing and allied health professionals in primary care, with triage and referral guidance developed in partnership with specialists. The aim of Rightpath is to promptly identify CYP with MSK pathology and triage them to the appropriate service (rheumatology, orthopaedics, neurodisability or urgent care), and to manage those who do not need specialist referral appropriately within primary care.

**Objectives:** To test the safety, feasibility and acceptability of Rightpath in primary care, and across two geographical areas in the UK to assess the transferability of the triage and referral guidance.

**Methods:** Piloted first at PMSKP, with iteration of the triage guidance and process followed by roll out at a second site (South Tyneside NHS Trust). Using mixed methods, evaluation focused on key areas:

*Implementation* – workshops with service providers (triagers and clinicians) held at two time points to refine triage guidance and process.

*Training* – for triagers based on their weekly log of triage experiences and regular case based discussions and feedback.

*Evaluation* – (i) Parent/patient questionnaire, incorporating the ‘Friends and Family’ test and ‘Collaborate’ (a patient reported measure of shared decision-making), completed immediately after consultation to explore expectations and satisfaction; (ii) Service providers weekly log documenting experiences and training needs; (iii) Routine patient data including demographic details, referral information, triage outcome, ultimate diagnosis/outcome and communication between triage teams and health care providers; (iv) Service providers signposted to key areas for self-directed learning (*paediatric musculoskeletal matters* [PMM] – www.pmmonline.org) and usage monitored.

This study had ethical approval and is NIHR CRN portfolio adopted.

**Results:** Total triaged 05/09/16 - 30/04/17 (101 to Rightpath, 264 to specialist paediatric services). The most common CYP MSK referrals from primary care were knee pain, foot pain, flat feet and back pain; the most common conditions triaged to Rightpath were foot pain, knee pain, flat feet, ‘clicking joints’ and in-toeing. No significant pathology has been triaged inappropriately so far to Rightpath. Feedback from 66 Rightpath family participants was positive with no complaints or requests for onward specialist referral; 100% would recommend the service, with median satisfaction (1-10) scores about primary care providers being high (1=’no effort made’ and 9=’every effort made’); ‘*helped understand your/your child’s health issues’ (8.9), ‘listened to things that matter most to you about your/your child’s health’ (8.9), ‘included what matters most to you in choosing what to do next’ (8.9)*. Primary care therapists and podiatry described the workload to be appropriate for their existing skills. Triage staff deemed the triage process manageable (*57% of decisions ‘easy/very easy’*) and triage guidance to be useful commenting that paediatric experience was important to support decision-making.

**Conclusion:** Initial data shows Rightpath to be feasible, safe and acceptable. The next phase of the pilot is in progress at the second site.

**Disclosure of Interest**

None Declared.

### P211 Hand grip strength, pinch grip strengths and manual dexterity in children and adolescents with juvenile idiopathic arthritis

#### Ela Tarakci^1^, Nilay Arman^1^, Sezgin Sahin^2^, Amra Adrovic^2^, Kenan Barut^2^, Ozgur Kasapcopur^2^

##### ^1^Faculty of Health Sciences, Division of Physiotherapy and Rehabilitation, Istanbul University, istanbul, Turkey; ^2^Department of Pediatric Rheumatology, Medical Faculty of Cerrahpasa, Istanbul University, Istanbul, Turkey

###### **Correspondence:** Ela Tarakci

**Introduction:** Juvenile idiopathic arthritis (JIA) is a chronic autoimmune condition of unknown etiology. JIA combine with joint pain and inflammation that affects children who are less 16 years of age and continue more 6 weeks. JIA is a chronic inflammatory disease resulting in joints arthritis and deformities in the hands and fingers. This leads to decrease of joint mobility and strength of the hands which can lead to physical and functional hand disability and subsequent reduction manual dexterity in daily living activities.

**Objectives:** The purposes of this study was to assessed hand grip strength, pinch grip strengths and manual dexterity in patients with JIA who have at least one affected wrist or finger joint.

**Methods:** 40 patients with JIA aged 6-16 years (33 girls and 7 boys) diagnosed according to ILAR classification criteria were enrolled in this cross-sectional study. “Jamar Hand Dynamometer” was used to evaluate the hand grip strength, “Pinchmeter” was used to evaluate the pinch grip strengths, “Nine Hole Peg Test” (NHPT) was used to evaluate the manual dexterity. Patients were asked to take the pegs from a container, one by one, and place them into the holes on board and replace back into the container. Duration of place and replace pegs was summed up and totally record for dominant side and non-dominant side. SPSS Version 21.0 program was used for statistical analysis.

**Results:** The mean age was 11.62 ± 2.41 years. 55% of patients had at least one affected finger joint. 87.5% of patients had at least one affected wrist joint. Table 9 shows hand grip strength, pinch strengths and manual dexterity in patients with JIA. Grip and pinch strengths showed a significant correlation with NHPT score for dominant side (Hand grip r = -0.34 p = 0.028, palmar pinch r = -0.52 p = 0.001, tip pinch r = -0.42 p = 0.006, key pinch r = -0.34 p = 0.03). Also, only tip pinch strength showed a significant correlation with NHPT score for non-dominant side (r = -0.33 p = 0.033).

**Conclusion:** We found correlation between manual dexterity with grip and pinch strengths in patients with JIA. Based on these results, we suppose that manual dexterity and decreased strengths of grip/pinch should be considered to increase independence of daily life activities in patients with JIA.

**Disclosure of Interest**

None Declared.

**Table 9 (Abstract P211).** Hand grip strength, pinch strengths and manual dexterity in patients wtih JIA

	Dominant side mean ± SD	Non-dominant side mean ± SD
Hand grip (Ib)	14.95 ± 10.56	11.79 ± 10.73
Palmar pinch (Ib)	4.07 ± 2.75	3.60 ± 2.88
Tip pinch (Ib)	2.24 ± 2.18	1.54 ± 1.89
Key pinch (Ib)	6.20 ± 2.87	5.42 ± 2.79
Nine hole peg test (sec)	21.13 ± 3.94	17.47 ± 3.21

## Imaging

### P212 Intra and inter observer reliability of ultrasound for the joints evaluation in children with juvenile idiopathic arthritis: the JIRECHO study

#### Linda Rossi-Semerano^1^, Alain Saraux^2^, Annette Von Scheven^3^, Sylvain Breton^4^, Marie Bossert^5^, Sorina Boiu^6^, Emmanuel Chatelus^7^, Geraldine Durand^8^, Perrine Dusser^3^, Sylvie Jean^9^, Laurence Goumy^10^, Anne Mathiot^11^, Sophie Chapelière^11^, Gaël Mouterde^12^, Frédérique Nugues^13^, Ahmed Ould Hennia^14^, Benedicte Rey^15^, Carine salliot^16^, Laetitia Sparsa^17^, Francois Hofer^18^, Sandrine Jousse-Joulin^2^

##### ^1^Department of Paediatric Rheumatology, Reference centre for Auto-inflammatory diseases, Le Kremlin Bicêtre, France; ^2^Department of Rheumatology, CHU la Cavale Blanche, Brest, France; ^3^Pediatric Rheumatology Unit of Western Switzerland, Pediatric Department, Lausanne, Switzerland; ^4^Department of Paediatric Radiology, Hôpital Necker Enfants Malades, AP-HP, Paris, France; ^5^Department of Rheumatology,Hôpital Nord Franche Comté, Trévenans, France; ^6^Department of Paediatric Rheumatology, University General Hospital Attikon, Athenes, Greece; ^7^Department of Rheumatology, Strasbourg Hospital, Strasbourg, France; ^8^Department of Rheumatology, Poitiers Hospital, Poitiers, France; ^9^Department of Rheumatology, CHR Rennes, Rennes, France; ^10^Department of Rheumatology, CHU Angers, Angers, France; ^11^Department of Paediatric Radiology, Bicêtre Hospital, Le Kremlin Bicêtre, France; ^12^Department of Rheumatology, Lapeyronie Hospital and & EA 2415, Montpellier, France; ^13^Clinique Universitaire d’Imagerie Pédiatrique - Hôpital Couple-Enfants CHU DE GRENOBLE-ALPES, Grenobles, France; ^14^Department of Rheumatologu, CHU de Chartres, Chartres, France; ^15^Department of Paediatric Rheumatology, HFME Bron, Bron, France; ^16^Department of Rheumatology, Centre Hospitalier Régional d’Orléans, Orléans, France; ^17^Department of Rheumatology, Centre hospitalier de Mulhouse, Mulhouse, France; ^18^Administrator of RES Fondation, Coordinator of the JIRcohorte, Lausanne, Switzerland

###### **Correspondence:** Linda Rossi-Semerano

**Introduction:** Over the last ten years, several studies have shown the benefits of ultrasound (US) in assessing joints in juvenile idiopathic arthritis (JIA), without discomfort for children (1). The JIRECHO study aims to ensure a systematic ultrasound follow-up for patients with JIA within the European multicentric JIRcohort. Evaluation of the reliability in the assessment of joints by different sonographers is essential to justify a multicentric cohort.

**Objectives:** To evaluate the intra and inter observer reliability on 10 joints between ultrasound JIA experts on children with different ages.

**Methods:** 17 experts (10 rheumatologists, 4 radiologists and 3 pediatricians) on US from 15 different centers were invited to participate in a 2 days-reliability exercise with acquisition of images on different JIA children’s group of ages. The expertise of participants being different, it was decided to work in couple during the two days.

The first day, a training session was organized to be agree on different scoring; then a practical session was performed on 4 children with different age groups (2-4, 5-8, 9-12, 13-16 years) with 2 rounds, in order to obtain the intra observer reliability. The second day concerns the inter observer reliability on 4 JIA children with different ages groups. The joints examined were: elbows, wrists, MCPs II, knees and ankles (examination techniques and definition of synovitis proposed by the US paediatric OMERACT) (2-4). All parents of the children participating at the study signed an informed consent.

**Results:** The intra observer reliability for detection of B mode and doppler synovitis was moderate for the majority of participants (k value 0.42-0.59 for 6), good for 1 (k 0.72) and fair for 1 couple (k 0.22). The inter observer reliability was highly variable and quite fair (k 0.16-0.50).

**Conclusion:** This is the first intra and inter observer US reliability study concerning joints in children with JIA performed in real time. Although the intra observer reliability was quite good, training sessions are necessary to interpret US images more uniformly.

**References**

1. Is ultrasound a validated imaging tool for the diagnosis and management of synovitis in juvenile idiopathic arthritis? A systematic literature review. Collado P, Jousse-Joulin S, Alcade M et al. Arthritis Care Res 2012;64:1011-9.

2. Toward standardized musculoskeletal ultrasound in pediatric rheumatology: Normal age related ultrasound findings. Collado P, Vojinovic J, Nieto JC et al. Arthritis Care Res (Hoboken), 2016 Mar;68(3):348-56.

3. Definitions for the sonographic features of joints in healthy children. Roth J, Jousse-Joulin S, Magni-Manzoni S et al. Arthritis Care Res (Hoboken). 2015 Jan;67(1):136-42.

4. Preliminary definitions for the sonographic features of synovitis in children. Roth J, Ravagnani V, Backhaus M et al. Arthritis Care Res (Hoboken). 2016 Oct 16. [Epub ahead of print].

**Disclosure of Interest**

None Declared.

### P213 Musculoskeletal ultrasonographic and magnetic resonance imaging findings in juvenile scleroderma

#### Maria Tsinti^1^, Vasiliki Dermentzoglou^2^, Elena Tsitsami^1^

##### ^1^Pediatric Rheumatology Unit, First Department of Pediatrics, University of Athens, Medical School, Children’s Hospital “Aghia Sofia”, Athens, Greece; ^2^Department of Radiology, Children’s Hospital “Aghia Sofia”, Athens, Greece

###### **Correspondence:** Maria Tsinti

**Introduction:** Musculoskeletal (MS) involvement and clinically evident arthritis occurs in up to 65% of patients with Juvenile Systemic or localized Scleroderma (JScl). It may be the first manifestation preceding even the onset of Raynaud or skin manifestations; patients presenting with arthritis, tenosynovitis or enthesistis may suffer from JScl. On the other hand clinical examination often underestimates MS involvement in JScl. Ultrasonographic (US) and Magnetic Resonance Imaging (MRI) can help distinguish arthritis with effusion from the dry tenosynovitis of JScl, define whether loss of range of motion (LOM) derives only from skin thickening or from bone and joint involvement and monitor disease progression.

**Objectives:** To describe the spectrum of MRI and US features in juvenile scleroderma with musculoskeletal involvement.

**Methods:** We describe MRI and color Doppler MSUS findings of clinically affected (with arthritis and/or LOM and/or overlying skin with edema or sclerosis) lower or upper extremities from 4 males and 2 females;2 with systemic scleroderma (ssc), 2 with linear scleroderma, 1 with generalized morphea and 1 with mixed morphea; median age 8,5 years, range 7-10,5; median time from symptom onset to MRI 11 months, range 2-24. MRI sequences; performed on a 1,5 Tesla MRI device included T1, fluid-sensitive, and T1-FS contrast-enhanced sequences. Comparisons were made to uninvolved areas of the extremity, and the contralateral extremity.

**Results:** Thickening of the dermis and infiltration of the subcutaneous fat with increase in signal intensity on fluid sensitive sequences and contrast-enhanced T1w images and hypointense signal lesions on unenhanced T1w images was apparent in 4 patients. In 2 male patients with generalized scleroderma, clinical LOM of fingers and wrists preceded skin sclerosis by 2 months. Joint and tendon sheath synovitis, indicated by initial MSUS, was detected in fluid sensitive and T1w enhanced images. The combination of tendon-sheath synovitis and muscular fascia thickening and enhancement concomitantly with contractures very characteristic of scleroderma, helped identify sclerodermatous musculoskeletal involvement in the absence of skin induration. Focal bone marrow edema depicted as high signal intensity in fluid sensitive sequences was found in 2 cases; 1 with generalized morphea without apparent overlying skin sclerodermatous lesion, 1 with linear sclerodermawith atrophic lesions in all overlying structures.

**Conclusion:** Musculoskeletal imaging features of juvenile scleroderma involving the skin, fascia, musculature and bones reflect pathomorphologic changes of this rare disorder and enable a complete assessment of the disease extent, including depth of infiltration and disease activity. Different imaging findings appear to reflect different subtypes of Juvenile Scleroderma. Whole depth lesions of the affected extremity in linear scleroderma, tendon sheath and joint synovitis in extremities with intact overlying skin structures in generalized morphea, bone marrow, fasciitis and skin infiltration in Systemic Scleroderma. Implementation of MSUS and MRI in Juvenile scleroderma led to earlier definition of the diagnosis and assisted the evaluation of disease extension.

**Disclosure of Interest**

None Declared.

## Immunodeficiency and infection related arthritis

### P214 Commune variable immunodeficiency mimicking sarcoidosis in pediatric age: a case report

**Abstract withdrawn**

### P215 Reactive arthritis triggered by yersinia – the analysis of 20 cases

#### Jacek Postępski, Aleksandra Rybkowska, Violetta Opoka-Winiarska, Aleksandra Sobiesiak, Edyta Olesińska

##### Department of Pediatric Pulmonology and Rheumatology, Medical University of Lublin, Poland, Lublin, Poland

###### **Correspondence:** Violetta Opoka-Winiarska

**Introduction:** Reactive arthritis (ReA) is the infection-related arthritis which presents the clinical picture of the seronegative spondyloarthropathy usually in HLA-B27 positive patients. Reactive arthritis triggered by *Yersinia* (*Y*ReA) is an important cause of morbidity and hospitalization in pediatric rheumatology.

**Objectives:** The aim of the study is to present the incidence, clinical characteristics, treatment and course of the disease in children with *Y*ReA.

**Methods:** A retrospective analysis of the medical records of all patients with the diagnosis of ReA was performed. All the patients were treated in the Department of Children Pulmonology and Rheumatology in Children University Hospital in Lublin, Poland between 1 January 2012 and 31 December 2016. Only patients with ReA and a positive serological test for *Yersinia spp* were included in the study. The demographic data, clinical manifestations, treatment, and course of the disease were analysed.

**Results:** In the analyzed period of time ReA was diagnosed in 117 patients. From among them, only twenty patients (17%) of the total (8 girls and 12 boys aged 5 to 17 (mean: 12.3) years) were diagnosed with *Y*ReA. The onset of *Y*ReA occurred in April - May in 7 of 20 patients and in October - December in 9 patients. Arthritis was preceded by gastrointestinal symptoms within 14 days on average. In majority of patients *Y*ReA affects the knees and ankles. 13 of 20 children developed oligoarthritis and 7 polyarthritis. In the onset of the disease ESR and CRP were elevated.Serum antibodies against *Yersinia spp* in IgM class were elevated (mean 56.6U/mL; positive > 24U/mL). Antigen HLA-B27 was positive in 19 of 20 patients.

In 13 of 20 patients the duration of acute arthritis was more than six weeks and in 10 more than 6 months. In acute illness 13 of 20 patients were treated with antibiotics. All children received non-steroidal anti-inflammatory drugs and 10 prednisone. In 13 of 20 patients intra-articular administration of methylprednisolone was performed. In the management of resistant arthritis: 13 patients were treated with sulfasalazine, 5 with methotrexate and one with cyclosporine.

In the study group the treatment was completed in 10 of 20 patients and the rest is in follow up. Six of our patients have reached adulthood. 5 of them completely recovered remain in remission without medication. One patient has been diagnosed with ankylosing spondylitis. Two of 20 patients developed psoriasis.

**Conclusion:** In the examined period of 5 years, *Yersinia* infection was the cause of 17% of all ReA. Seasonal morbidity with the onset of symptoms in autumn and spring was observed. In most patients family history of spondyloarthropathies was negative.

In majority of patients *Y*ReA was associated with HLA-B27 antigen. Some patients evolve into other spodyloarthropaties. In some patients with *Y*ReA the course of the disease is prolonged or chronic and has become a significant therapeutic problem.

**Disclosure of Interest**

None Declared.

## Infections - Immunoregulation - Biomarkers

### P216 The frequency of infections in patients with juvenile idiopathic arthritis on biologic agents: one year prospective study

#### Deniz Aygun^1^, Amra Adrovic^2^, Sule Bektas^2^, Sezgin Sahin^2^, Kenan Barut^2^, Haluk Cokugras^1^, Yildiz Camcioglu^1^, Ozgur Kasapcopur^2^

##### ^1^Pediatric infectious disease, Istanbul University, Cerrahpasa Medical School, Istanbul, Turkey; ^2^Pediatric Rheumatology, Istanbul University, Cerrahpasa Medical School, Istanbul, Turkey

###### **Correspondence:** Amra Adrovic

**Introduction:** Juvenile idiopathic arthritis (JIA) is one of the most common chronic diseases of childhood that causes significant physical disability with high morbidity and mortality. Biologic agents are commonly used in patients with JIA as well as standard disease modifying drugs to have an acceptable quality of life, to control pain, to protect the function of joint, to ensure normal growth and to decrease the systemic complications of the disease. Choosing the most effective and safest treatment regimen is the cornerstore in management of these patients. TNF-alpha inhibitors, IL-1 ve IL-6 antagonists are commonly used biologic agents. Especially, as the firstly used biologic agent, the effectivity of TNF-alpha inhibitors is well documented. However, as these drugs act as immunosuppressant and immunomodulator, they have the risk to lead serious infections. There are studies in adults investigating the infectious complications of biologic agents and many of them reported the efficacy in treatment of adult patients with rheumatoid arthritis without any serious complications.

**Objectives:** Herein, we aimed to study the infectious complications of biologic agents in JIA patients in one year period.

**Methods:** JIA patients on biologic treatment (etanercept, adalimumab, anakinra, infliximab, canakinumab and tocilizumab) were examined by the pediatric infectious disease specialist in every 2 months during one year long.

**Results:** 307 patients (171 female, 136 male) patients diagnosed as systemic JIA (60), seronegative polyarticular (83), seropositive polyartivular (18), oligoarticular (96), juvenile psoriatic arthritis (10), enthesitis related arthritis (40) were included in the study. 189 patients were on etanercept, 60 were on adalimumab, 22 were on anakinra, 11 were on infliximab, 12 were on canakinumab and 13 were on tocilizumab treatment. At the end of one year period, 57% (n:175) of patients developed infection and 43% (n:132) of them completed the treatment episode without any infection. The infection rate was highest in systemic JIA and lowest in enthesitis related arthritis. The overall infection rate was the highest on infliximab and tocilizumab, lowest with etanercept. Most of the infections were viral upper respiratory tract infection and tonsillopharyngitis (42.7%) which were treated at outpatient clinics. Only 3 of 307 patients had serious infections (two pneumonia, one pleural effusion) which required hospitalization. One of them with systemic JIA, a four year old girl, having anakinra episode with concomitant high dose steroid developed pneumonia and pleural effusion on the second and the sixth month of treatment, respectively. *Streptococcus pneumoniae serotype* 19 A was isolated in the pleural fluid culture of the patient. She was fully vaccinated with 7 valent pneumoccoc vaccine, and this serotype is not included in vaccine. The second pneumonia case was also diagnosed as systemic JIA and was on anakinra treatment. Seven of the patients had varicella infection without any complication requiring hospitalization, all of them did not have varicella vaccine, four of them were on etanercept, others were on anakinra, infliximab, canakinumab regimen respectively.

**Conclusion:** We comment that the biologic agents can be safely used in JIA. The serious infection developed only in two episode with *Streptococcus pneumoniae.* Even the healhty children vaccinated with pneumoccoc also have the same risk to develop serious infection with this serotype which is not included in vaccine. The varicella infections also occurred in the unvaccinated patients who have the risk to develops as high as healthy children and non of the varicella infections were complicated.

**Disclosure of Interest**

None Declared.

### P217 Biomarkers for the diagnosis and the identification of risk of macrophage activation syndrome (MAS) in systemic juvenile idiopathic arthritis (SJIA)

#### Claudia Bracaglia^1^, Denise Pires Marafon^1^, Ivan Caiello^1^, Kathy de Graaf^2^, Maria Ballabio^2^, Walter Ferlin^2^, Sergio Davi’^3^, Grant Schulert^4^, Angelo Ravelli^3^, Alexi A. Grom^4^, Robert Nelson^2^, Cristina de Min^2^, Fabrizio De Benedetti^1^

##### ^1^Division of Rheumatology, Ospedale Pediatrico Bambino Gesù IRCCS, Roma, Italy; ^2^SA Novimmune, Geneva, Switzerland; ^3^University of Genova, Istituto Giannina Gaslini IRCCS, Genova, Italy; ^4^Division of Pediatric Rheumatology, Cincinnati Children’s Hospital Medical Center, Cincinnati, OH, USA

###### **Correspondence:** Claudia Bracaglia

**Introduction:** We have recently reported high levels of IFNγ and of the IFNγ-related chemokines, (CXCL9 and CXCL10) in patients with MAS (1).

**Objectives:** To evaluate if blood levels of IFNγ, CXCL9, CXCL10, IL-18, neopterin and sCD25, may help to identify sJIA patients with the predisposition to develop MAS, and additionally if they may help clinicians to distinguish MAS from active sJIA.

**Methods:** Circulating levels of biomarkers were measured by Luminex assay in 57 samples obtained from 24 patients with active sJIA and in 37 samples from 20 MAS patients with variable degrees of disease severity and under different treatments at time of sampling.

**Results:** Levels of IFNγ, CXCL9, CXCL10, IL-18, neopterin and sCD25 were significantly elevated in MAS compared to active sJIA without MAS at sampling (p-values <0.0001, except for IL-18 p = 0.012) and were significantly correlated with laboratory parameters of disease severity, except for IL-18, whose levels were available only for a portion of the samples. During active sJIA without MAS at sampling, levels of the IFNg-induced chemokines (CXCL9 and CXCL10) were significantly higher in patients with a history of MAS as compared to those of patients without a history of MAS (Table 10). In order to verify if measurement of these biomarkers could help in distinguishing MAS from active sJIA, we analyzed sensitivity and specificity and the area under the curve (AUC) for each parameter. The highest AUC (=0.95) was found for neopterin levels >14.62 nmol/L (Sensitivity 85.3% Specificity 84.6%). AUC for IFNγ (>8.5 pg/ml), CXCL9 (>2677 pg/ml), CXCL10 (>725.5 pg/ml), IL-18 > (4309 pg/ml) and sCD25 (>211.65 pg/ml) were 0.77, 0.82, 0.82, 0.82 and 0.86, respectively.

**Conclusion:** Elevation of neopterin and CXCL9, both reflecting IFNγ production, and their correlation with laboratory parameters, supports the pathogenic role of IFNγ in MAS. Circulating levels of CXCL9 and CXCL10 (to a lesser extent of IL-18, though, not reaching statistical significance) are higher in patients with a history of MAS compared to patients without a history of MAS, suggesting subclinical activation of this pathway even in the absence of overt MAS and that CXCL9 and CXCL10 may identify patients at high risk for MAS; larger studies are needed. Regarding distinguishing MAS from active sJIA, levels of neopterin, IFNγ, CXCL9, CXCL10, sCD25, and IL-18 may be used and the possibility to integrate the currently available classification criteria with one or more of these biomarkers investigated in larger studies.

**Reference**

1. Bracaglia C, et al. Elevated circulating levels of interferon-γ and interferon-γ-induced chemokines characterise patients with macrophage activation syndrome complicating systemic juvenile idiopathic arthritis. Ann Rheum Dis. 2017 Jan; 76(1):166-172.

**Disclosure of Interest**

C. Bracaglia: None Declared, D. Pires Marafon: None Declared, I. Caiello: None Declared, K. de Graaf Employee of: Novimmune, M. Ballabio Employee of: Novimmune, W. Ferlin Employee of: Novimmune, S. Davi’: None Declared, G. Schulert Consultant for: Novartis, A. Ravelli: None Declared, A. Grom Grant/Research Support from: NovImmune, Novartis Pharmaceutical Corporation, Roche Pharmaceuticals, R. Nelson Employee of: Novimmune, C. de Min Employee of: Novimmune, F. De Benedetti Grant/Research Support from: Novartis, Novimmune, Hoffmann- La Roche, SOBI, AbbVie, Pfizer.

**Table 10 (Abstract P217).** Cytokine levels in sJIA patients with active disease with or without history of MAS

	sJIA with history of MAS (N = 17)	sJIA without history of MAS (N = 40)	*P*
IFNγ (pg/ml)	5.6 (3.2-14.4)	5.3 (3.2-10)	0.74
CXCL9 (pg/ml)	3889 (965-7142)	519 (385-1168)	0.0015
CXCL10 (pg/ml)	764 (323-1259)	215 (152-470)	0.0003
IL-18 (pg/ml)	4405 (582-7122)	439 (312-824)	0.067
neopterin (nmol/L)	8.2 (6.5-13.9)	8.1 (6.6-8.8)	0.94
sCD25 (pg/ml)	80 (80-229)	80 (80-193)	1

Values are expressed as median (IQR)

### P218 Active tuberculosis in children and adolescents receiving tumor necrosis factor alpha inhibitors: a pTBnet case series

#### Joan Calzada-Hernández^1,2^, Marc Tebruegge^3,4^, Nicole Ritz^4,5^, Elisabeth Schölvinck^6^, Antoni Noguera-Julian^7^

##### ^1^Systemic inflammatory response in paediatric age, Institut de Recerca Sant Joan de Déu, Esplugues de Llobregat (Barcelona), Spain; ^2^Pediatric Rheumatology Unit, Department of Pediatrics, Hospital Sant Joan de Déu, Esplugues de Llobregat (Barcelona), Spain; ^3^Faculty of Medicine & Global Health Research Institute, University of Southampton, Southampton, UK; ^4^Department of Paediatrics, The University of Melbourne, Parkville, Australia; ^5^Paediatric Infectious Diseases, Vaccinology, and Paediatric Pharmacology, University Children’s Hospital Basel, Basel, Switzerland; ^6^Department of Pediatrics, University of Groningen, University Medical Center Groningen, Groningen, Netherlands; ^7^Infectious Diseases Unit, Department of Pediatrics, Hospital Sant Joan de Déu, Universitat de Barcelona, Barcelona, Spain

###### **Correspondence:** Joan Calzada-Hernández

**Introduction:** Currently, data on active tuberculosis (TB) in children started on anti-TNFα therapy are very limited. Data from adult studies suggest that anti-TNFα therapy is associated with a 4- to 20-fold increased risk of progressing from latent TB to active TB. No data specific to children currently exist except for single case reports.

**Objectives:** To establish the first multi-centre paediatric case series of patients who have developed active TB following initiation of anti-TNFα therapy.

**Methods:** The study was approved by the Steering Committee of the Paediatric Tuberculosis Network European Trials Group (pTBnet). A total of 158 pTBnet members across 31 European countries were contacted by group email and asked to contribute cases. Inclusion criteria: any patient <18 years of age with microbiologically-confirmed (i.e. culture- or PCR-confirmed) or clinically-diagnosed active TB disease after initiation of anti-TNFα therapy.

**Results:** This project aims to compile a retrospective case series of active TB disease in children receiving anti-TNFα therapy. So far, 10 cases have been identified Europe-wide. This will provide insights into the presenting features, immunological TB test results, response to treatment, and outcome of these children. Combining cases identified via pTBnet with cases from PRES members will aid generating more robust data.

**Conclusion:** This study will provide new insights into this condition and raise awareness among clinicians providing care for children with chronic inflammatory conditions. The project facilitates joint work between pTBnet and PRES members. Contribution of patients fulfilling the inclusion criteria managed by PRES members is strongly encouraged (please contact: jcalzada@sjdhospitalbarcelona.org).

**Disclosure of Interest**

None Declared.

### P219 Biochemical markers of cardiovascular disease in children with rheumatic diseases

#### Iryna Chyzheuskaya^1^, Lyudmila Byelyaeva^1^, Tamara Yuraga^2^

##### ^1^Pediatrics, Belarusian Medical Academy of Postgraduate Education, Minsk, Belarus; ^2^Belarusian Medical Academy of Postgraduate Education, Minsk, Belarus

###### **Correspondence:** Iryna Chyzheuskaya

**Introduction:** In real clinical practice, the specialist is faced with the task not only of treating systemic rheumatic disease, but also correction of various concomitant pathologies that arise against the background of the underlying disease. Blood apolipoproteins are biochemical markers of the development of cardiovascular diseases.

**Objectives:** In this connection, it was expedient to measure the levels of apolipoproteins (ApoA, ApoB, ApoE) in the blood serum of children with rheumatic diseases.

**Methods:** Between 2008 and 2015, 167 patients with rheumatic diseases were examined, including 54 boys and 113 girls. All patients were divided into 3 clinical groups. Group I included 147 children with juvenile idiopathic arthritis (mean age 11.9 ± 3.4 years), group II comprised 40 children with juvenile scleroderma (mean age 12.4 ± 2.8 years), III - 25 children with systemic lupus erythematosus (mean age 13.1 ± 1.7 years).

All patients underwent complex clinical and laboratory examination, including the collection of genealogical anamnesis, anamnesis of life and disease, anthropometry, laboratory tests (blood lipids: high density lipoprotein, low-density lipoproteins, triglycerides, total cholesterol, levels of apolipoprotein A (ApoA), apolipoprotein B (ApoB) and apolipoprotein E (ApoE).

**Results:** Most children received long-term glucocorticosteroid and immunosuppressive therapy. Among the children examined, 72 children with juvenile idiopathic arthritis (48.9%), 10 children with systemic lupus erythematosus (40%) and 12 children with juvenile scleroderma (30%) were overweight. Obesity was diagnosed in 35 children with juvenile idiopathic arthritis (23.8%), 11 children with systemic lupus erythematosus (44%) and 6 children with juvenile scleroderma (15%).

ApoA is the main protein component of high density lipoproteins and promotes the reverse transfer of cholesterol from the walls of blood vessels to the liver. A significant (p <0.05) decrease in the concentration of ApoA in patients of all clinical groups was found in comparison with the control group.

The ratio of ApoA and ApoB is a valuable indicator for assessing the atherogenic shift and the risk of cardiovascular disease, Is under genetic control and exceeds the prognostic value of the determination of individual apolipoproteins. In norm ApoA/ApoB > 1 and is considered as a low atherogenic risk factor. Among children with rheumatic fever, ApoA/ApoB <1 was found in 16.7% of patients with systemic lupus erythematosus, in 14.8 patients with juvenile idiopathic arthritis and in 12.5% of patients with juvenile scleroderma. An inverse correlation was established between the ApoA/ApoB ratio and the serum cholesterol concentration (rs = -0.31, p <0.01).

ApoE is part of fatty particles of chylomicrons and very low density lipoproteins, initiating their capture and removal from the blood by interacting with a specific receptor on the surface of the liver cells. In the study, an increased level of ApoE in the blood serum of children with rheumatic diseases was found in comparison with the control group. A correlation was established (rs = 0.37, p <0.05) between the content of ApoE and total cholesterol in the blood serum.

Lipoprotein lipase (LPL), is a key enzyme of lipid metabolism. A direct relationship between its serum content and concentration of low density lipoproteins (rs = 0.27, p <0.05) and its inverse high level lipoprotein level (rs = -0.3, p <0.05) was established. The highest LPL content was found in children with systemic lupus erythematosus (p <0.05) compared with the control group. There was a significant difference (p <0.05) and by gender: the level of LPL in the serum in girls was 25.8 [16.7 - 54.4] ng/ml, in boys - 15.6 [7.8 - 29.0] ng/ml. A correlation was also established between the LPL level and the body mass index (rs = 0.25, p <0.05).

From 89.7% of children with juvenile idiopathic arthritis, in 82.5% of children with juvenile scleroderma and 96% of children with systemic lupus erythematosus prevailed hypercoagulability. Serum levels of fibrinogen was significantly exceeded normal values and were positively correlated with indices of disease activity: value of the ESR and serum CRP concentration.

**Conclusion:** Based on the data obtained, risk groups for the development of atherogenesis and thrombotic complications among children with rheumatic diseases have been identified.

**Disclosure of Interest**

None Declared.

### P220 Faecal calprotectin as screening tool to identify inflammatory bowel disease among juvenile idiopathic patients: results from a monocentric Italian study

#### Giovanna Ferrara^1^, Lara Sancin^1^, Chiara Bibalo^1^, Alberto Tommasini^2^, Andrea Taddio^3, 4^, Serena Pastore^4^

##### ^1^Department of pediatrics, University of Trieste, Trieste, Italy; ^2^Institute of Maternal and Child Health IRCCS Burlo Garofolo, Trieste, Italy; ^3^University of Trieste, Trieste, Italy; ^4^Institute for Maternal and Child Health Burlo Garofolo, Trieste, Italy

###### **Correspondence:** Giovanna Ferrara

**Introduction:** Arthritis is the most frequent extra-intestinal manifestation of inflammatory bowel diseases (IBD) (7-21%). Onset of IBD-related arthritis is usually before the development of gastrointestinal symptoms and hence before IBD diagnosis. Faecal calprotectin is recommended from NICE guidelines to reduce the number of useless endoscopies in patients with gastrointestinal symptoms but it is not usually performed as screening for IBD in patients with JIA. Moreover, there is no consensus about the right cut-off to obtain the better sensibility and specificity in paediatric patients.

**Objectives:** The aim of this study was to test faecal calprotectin as a screening tool to identify JIA patients with underlying IBD. Secondary outcomes were to identify clinical or laboratory findings able to predict IBD among JIA patients as well as to define a faecal calprotectin cut-off level with the best sensibility and specificity for IBD.

**Methods:** All patients with JIA admitted to the Rheumatology Service of IRCCS Burlo Garofolo (Trieste) from January 2008 to August 2016 were enrolled. We excluded patients with systemic JIA. Data collection included age at diagnosis, gender, type of JIA, presence of ANA, history of iridociclytis, JADAS-27 score, time from JIA onset, age, presence of gastrointestinal symptoms, presence of weight loss or growth retardation, recent viral infection, blood analysis, concomitant and previous treatment. Faecal calprotectin level <100 mg/kg was considered normal. Patients with faecal calprotectin ≥100 mg/kg were asked to repeat the test. Patients with a negative second test (<100 mg/kg) were considered healthy. Patients with a second positive test underwent abdominal ultrasonography, esophagogastroduodenoscopy, colonoscopy with biopsies and small bowel videocapsule undoscopy where needed. The diagnosis of IBD was confirmed only in case of a histologic findings compatible with IBD. For statistical analysis we performed Pearson’s linear correlation test and non parametric Kruskall-Wallis tests.

**Results: One-hundred thirteen** JIA patients were enrolled. Seven out of 113 (8%) were found affected by IBD and were all Crohn’s Disease. Among JIA subtypes, enthesitis related Arthritis (ERA) was the most frequent (4/7), followed by olygoarticular (2/7) and undifferentiated arthritis (1/7). ERA was found to be a significant risk factor for IBD (OR 69,33, 95% CI 8-538, p-value < 0,0001). Two patients with IBD-related arthritis were receiving anti-TNFa therapy for severe arthritis when developed IBD. Only 2 patients (29%) presented diarrhea, fecal blood or abdominal pain. Three patients had growth retardation without intestinal symptoms. One patient had an arthritis uncontrolled from therapy. Faecal calprotectin was significantly higher in patients with IBD than in patients without IBD (median value 1500 mg/kg, range 60-2000, vs 20 mg/kg range 10-50, p-value < 0,0001). The cut-off value with a better sensibility and specificity was found to be 250 mg/kg or more (positive predictive value 70%; 95% CI 0.35-0.93, specificity 98%; 95% CI 0.93-0.99, sensibility 100%; CI 0.59-1.00, negative predictive value 100%, 95% CI 0.97-1.00). Faecal calprotectin was found to be associated with disease activity valued with JADAS score (correlation index 0.69%; 95% CI 0.22-0.90; p-value 0,009)

**Conclusion:** Faecal calprotectin is a useful, economic and non invasive diagnostic tool to identify patients with subclinical IBD among patients with JIA patients.

**Trial registration identifying number:** 1)

**Disclosure of Interest**

None Declared.

### P221 An immune-regulatory role of the alarmin S100A8/A9 during collagen-induced arthritis

#### Meike Kuhlencord, Stefanie Zenker, Johannes Roth, Thomas Vogl

##### Institute of Immunology, Münster, Germany

###### **Correspondence:** Meike Kuhlencord

**Introduction:** The alarmin S100A8/A9 has long been characterized as a pro-inflammatory trigger molecule amplifying immune responses. However, recent evidence also suggests an immune-regulatory role of this protein, as observed in cancer by promoting the differentiation of myeloid-derived suppressor cells (MDSC).

**Objectives:** Since the molecular mechanisms involved in S100A8/A9 driven MDSC differentiation are poorly understood and the role of these cells in autoimmune diseases such as rheumatoid arthritis remain largely unknown, we analyzed the involvement of S100A8/A9 for MDSC differentiation under non-tumor conditions and evaluated their contribution during the disease development and progression of rheumatoid arthritis.

**Methods:** To investigate the effect of S100A8/A9 on MDSC differentiation, bone marrow cells from wild type (wt), S100A9 knockout (A9ko) and S100A9 transgenic (A9tg) mice were analyzed. Accumulation of MDSC and their phenotypical characterization was performed by FACS analysis and functional characterization included arginase activity, NO- and ROS-production and T cell proliferation assays.

The role of S100A8/A9 and MDSC in arthritis was investigated using the collagen-induced arthritis (CIA) mouse model. Accumulation of MDSC in different organs was analyzed by FACS and systemic S100A8/A9 levels were measured by ELISA. Ex vivo functional analysis of purified MDSC was performed to assess the potential of these MDSC to inhibit T cell responses. Induction of MDSC by treatment with S100A8/A9 or pharmacological inhibition of MDSC function in CIA mice was used as proof of concept experiments.

**Results**: Our in vitro studies reveal that prolonged stimulation of myeloid progenitor cells with S100A8 induces the differentiation of immature cells that phenotypically as well as functionally resemble MDSC. These cells comprise typical MDSC features such as co-expression and up-regulation of arginase activity and NO production as well as strong suppressive effects on the proliferation of both CD4- and CD8-positive T cells. Toll-like receptor 4 signaling was identified as the major signaling pathway of S100A8 induced MDSC differentiation. Furthermore, numbers of MDSC and their suppressive activity on a per-cell basis were decreased in cells lacking endogenous S100A8/A9 implying a dual role of this alarmin for MDSC differentiation and function.

Investigating the role of S100A8/A9 and MDSC in a mouse model of CIA, a clear correlation between disease scores, MDSC numbers and systemic S100A8/A9 levels was observed. Furthermore, disease activity was reduced in wt and A9tg mice compared to A9ko mice and was in line with a tissue-specific increased accumulation of MDSC in the lymph nodes. Next to an enhanced suppressive activity of MDSC isolated from lymph nodes of wt and A9tg mice, these MDSC promoted the accumulation of regulatory T cells (Treg) whilst suppressing the differentiation of TH17 cells. In contrast, MDSC isolated from lymph nodes of A9ko mice had no effect on Treg differentiation and did not inhibit TH17 emergence. Inhibition of MDSC function in CIA mice by administration of a nitric oxide synthase inhibitor exacerbated the disease severity while repeated treatment of A9ko CIA mice with S100A8 ameliorated arthritis symptoms that was in line with increased numbers of MDSC in the lymph nodes.

**Conclusion:** Our in vitro results clearly show a S100A8/A9 dependent accumulation of cells that phenotypically as well as functionally resemble MDSC. In vivo data strongly support the importance of these findings. By influencing MDSC accumulation and function, S100A8/A9 is critically involved in regulating the disease outcome in rheumatoid arthritis, implying an important role of S100A8/A9 derived MDSC in regulating immune reactions during autoimmune diseases and thus a immune-regulatory role of this originally characterized pro-inflammatory alarmin.

**Disclosure of Interest**

None Declared.

### P222 In active juvenile idiopathic arthritis the HLA-DR associated network module may drives the functionally dichotomic pathogenic immune subsets from a common precursor

#### Pavanish Kumar^1^, Jing Yao Leong^1^, Phyllis Chen^1^, Joo Guan Yeo^1,2^, Camillus Chua^1^, Suzan Saidin^1^, Sharifah Nur Hazirah^1^, Justin Tan^2^, Thaschawee Arkachaisri^2,3^, Alessandro Consolaro^4^, Marco Gattorno^4^, Alberto Martini^4^, Salvatore Albani^1,3^

##### ^1^SingHealth Translational Immunology and Inflammation Centre, Singapore, Singapore; ^2^KK Women’s and Children’s Hospital, Singapore, Singapore; ^3^Duke-NUS Graduate Medical School Singapore, Singapore, Singapore; ^4^Second Paediatric Division, University of Genoa and G Gaslini Institute, Genova, Italy

###### **Correspondence:** Pavanish Kumar

**Introduction:** We have previously identified T cell subsets in both Teff (CPLs) and Treg (iaTreg) compartments that are HLA-DR positive, antigen experienced, pro-inflammatory, correlating with disease activity and sharing strong TCR oligoclonality with synovial T cells. Their phenotype and association with clinical fate inspired out current hypothesis that these functionally discordant cells may originate from a common precursor.

**Objectives:** To understand the pathogenicity drivers, find key genes, modulated pathways or network of gene associated with pathogenic HLA-DR positive subsets.

**Methods:** Next generation RNA sequencing was performed on sorted CPLs (CD3^+^ CD4^+^ CD14^-^ HLADR^+^ CD25/CD127 Teff gate) and iaTregs (CD3^+^ CD4^+^ CD14^-^ CD25^hi^ CD127^lo^ Treg gate) from 8 active disease juvenile idiopathic arthritis (JIA) patients. CPLs and iaTregs were sorted using flow cytometer, RNA was extracted and and library was prepared for RNA-sequencing. The paired end RNA sequencing was performed using illumina HI-Seq platform. Raw reads were aligned using STAR aligner and reads were summarised at gene level using featureCount programme. Raw read counts were further analysed in R statistical programming environment.

**Results:** Comparative gene expression analysis, between CPLs vs iaTreg and that of the common pool of Teff vs Treg, and pyhlogenetic association analysis suggests that Teff and Treg converges to a common branch from where iaTreg and CPLs have emerged. TCR sequence oligoclonality in CPLs/iaTregs versus that of the common Teff/Treg pool reinforce the possibility of a common precursor. Furthermore, Weighted gene correlation network analysis (WGCNA) identified strongly coordinated HLA-DR gene network module and suggests its potential role as the driver of pathogenic T cell subsets from HLA-DR negative subsets. Gene set enrichment analysis suggest that HLA-DR module genes are involved in TNF-a signalling, Inflammatory response, complement, and apoptosis. Transcription factors (TFs) gene regulatory network (TF-GRN) analysis is performed to identify the key regulatory molecules driving the convergence of HLA-DR negative Teff and Treg population to HLA-DR positive pathogenic CPL and iaTreg population. Global TF-GRN network analysis identified FOXP3, CEBP, SPI and E2F1 as key transcription factor driving the Teff, Treg to pathogenic iaTreg and CPLs. Intriguingly, as both subsets transit in the inflamed synovium, these data underscore a potential role of the microenvironment in shaping two functionally dichotomic populations from a common precursor.

**Conclusion:** The presented study,suggests the role of identified HLA- DR gene module in convergence and development of pathogenic iaTreg and CPL T cell subsets these have emerged from the functionally dichotomous Treg and Teff cell subsets.

**Disclosure of Interest**

None Declared.

### P223 IL-18 as a diagnostic biomarker for systemic JIA: differentiating systemic JIA from childhood leukaemia, non-systemic JIA subtypes and other auto-immune conditions like vasculitis

#### Arjen Leek^1^, Nienke ter Haar^1,2^, Judith Wienke^2^, Ayman el Idrissi^1^, Dirk Holzinger^3^, Wilco de Jager^2^, Sebastiaan Vastert^1,2^

##### ^1^Department of Pediatric Rheumatology, University Medical Center Utrecht, Utrecht, Netherlands; ^2^Laboratory for Translational Immunology, University Medical Center Utrecht, Utrecht, Netherlands; ^3^Department of Pediatric Rheumatology and Immunology, University Children’s Hospital Muenster, Muenster, Germany

###### **Correspondence:** Arjen Leek

**Introduction:** Systemic onset Juvenile Idiopathic Arthritis (sJIA) is a disease characterized by systemic inflammation in addition to arthritis and its diagnosis currently still depends on the clinical ILAR classification criteria of 2003, with arthritis as a required criterium. A significant portion of patients however exhibit inflammatory symptoms weeks, sometimes even months before showing signs of any joint involvement, making the diagnosis of sJIA challenging for the treating clinician due to the broad differential diagnosis of systemic inflammation. Recent insights in the underlying auto-inflammatory disease mechanisms in sJIA have proposed several candidate biomarkers aiding in the diagnostic process, including interleukin-18 (IL-18), a member of the IL-1 famtenosyily. Here, we compare peripheral blood serum levels of IL-18 in sJIA at disease onset with several important differential diagnoses.

**Objectives:** To test the value of IL-18 as a diagnostic biomarker aiding in diagnosing sJIA and differentiating sJIA from children with systemic infection (INF), acute lymphatic leukaemia (ALL) and auto-immune conditions like vasculitis.

**Methods:** A cohort was assembled retrospectively consisting of patients with sJIA at disease onset (n = 39, presenting initially with and without arthritis, ultimately diagnosed according to ILAR criteria), oligo- and polyarticular JIA (n = 28), ALL (n = 18), various auto-immune diseases including vasculitis (n = 14) and healthy controls (HC, n = 30). Clinical and laboratory data were extracted from patient files. Peripheral blood serum IL-18 measurements were performed using a multiplex immunoassay based on Luminex technology.

**Results:** Of all sJIA patients (n = 39), 97.1% experienced fever on the day of sampling. A cutaneous rash was seen in 82.9% of patients and 40% of patients suffered from lymphadenopathy at disease onset. 68.6% of patients showed overt arthritis (median joint count 1.5, IQR 0-3) and 77.1% suffered from arthralgia (median joint count 2, IQR 1-4). At time of sampling, 57.1% of patients were being treated with NSAID.

IL-18 values (pg/ml, median, IQR) were evidently elevated in sJIA (5465, 1962-13428) and differed significantly from the levels measured in the other disease groups (oligo JIA (274, 172-378), poly JIA (177, 121-342), ALL (542, 315-869), other auto-immune diseases including vasculitis (64, 36-131)) and healthy controls (228, 175-327). The IL-18 levels in the serum of children with systemic infections are currently being measured.

We evaluated different cut-off values for IL-18 using an ROC curve. A value of 1500pg/ml performed best, with a specificity of 0.976 and a sensitivity of 0.800.

**Conclusion:** Here we show the potential value of IL-18 as a diagnostic biomarker for sJIA in the process of differential diagnosis. When sJIA is suspected in a patient with symptoms of systemic inflammation with or without arthritis, an IL-18 level of 1500 or higher seems clearly suggestive of sJIA. In patients with suspected sJIA but IL-18 levels below 1500, we recommend additional diagnostic modalities, including for example PET scan or bone marrow biopsy where indicated, to rule out other diagnoses like childhood malignancy.

**Disclosure of Interest**

None Declared.

### P224 A novel immune-regulatory function of S100A8 in developing dendritic cells

#### David Popp^1^, Frank Rühle^2^, Wilco de Jager^3^, Thomas Vogl^1^, Johannes Roth^1^

##### ^1^Institute of Immunology, Münster, Germany; ^2^Institute of Human Genetics, Münster, Germany; ^3^Multiplex Core Facility, Wilhelmina Children’s Hospital (UMC Utrecht), Utrecht, Netherlands

###### **Correspondence:** David Popp

**Introduction:** S100A8/A9 heterodimers are efficient and reliable biomarkers for monitoring disease activity in inflammatory disorders like juvenile idiopathic arthritis. On a molecular level these proteins are characterized as endogenous alarmins that, upon release by activated or necrotic phagocytes, amplify inflammation by directly binding to Toll-like receptor (TLR) 4. Surprisingly, we now identified an additional immune-regulatory function of S100A8 in developing monocyte-derived dendritic cells (moDCs).

**Objectives:** This study aims to analyze immune-regulatory mechanisms of S100A8 proteins in human DCs.

**Methods:** Monocytes isolated from human blood donors are differentiated to immature moDCs with or without exposure to S100A8. After subsequent LPS-induced maturation specific linage marker as well as activation marker expression on the cell surface is determined by flow cytometry. In addition, cytokines and chemokines, secreted during development are analyzed by Luminex cytokine arrays. The metabolic state of moDCs is examined by using Seahorse XFp Analyzer assays. On a functional level the ability of these cells to induce autologous CD4^+^, CD8^+^, and γδ T-cell proliferation is investigated. Finally, to identify molecular mechanisms leading to an immune-regulatory phenotype, the mRNA expression of moDCs is analyzed by genome-wide gene expression arrays.

**Results:** Our results clearly demonstrate that early exposure to S100A8 interferes with *in-vitro* differentiation of moDCs. Compared to controls S100A8-exposed moDCs show dramatically reduced surface expression of co-stimulatory molecules after LPS-induced maturation. In addition, early treatment of moDCs with S100A8 alters the secretion pattern of immune-regulatory cytokines and chemokines depending on the developmental state of moDCs. S100A8-induced effects on moDC maturation are not limited to TLR4 stimulation but rather trigger a common state of unresponsiveness. Furthermore, essential biochemical processes like mitochondrial respiration and glycolytic function are diminished in S100A8-treated moDCs.

As a consequence, S100A8-exposed moDCs have a reduced potential to induce autologous CD4^+^, CD8^+^, and γδ T-cell proliferation. We can show that these broad differences are mainly caused by reduced surface expression of co-stimulatory molecules on S100A8-treated moDCs.

Mechanistically, genome-wide gene expression analysis reveals dramatic differences in gene expression between S100A8-exposed and conventionally differentiated moDCs. We demonstrate that S100A8 pre-treatment of moDCs significantly blocks LPS-induced gene expression during moDC activation. Interestingly, *in-silico* analysis of transcription factor networks predicts NFκB and C/EBPδ as master regulators of S100A8-induced effects in developing moDCs. C/EBPδ on protein level, indeed, shows reduced expression in S100A8-differentiated moDCs prior and after LPS-induced maturation when compared to conventionally differentiated moDCs.

**Conclusion:** Taken together, our results demonstrate a novel regulatory mechanism of innate immunity to prevent overwhelming immune responses. C/EBPδ seems to be a novel master regulator in this process. Keeping in mind that S100A8 and S100A9 are highly abundant in synovial fluid and tissue of JIA, dysregulated repression of detrimental adaptive immune responses might represent a novel pathomechanism of this chronic inflammatory disease. Therefore, S100A8-differentiated immune-suppressive DCs potentially represent a promising therapeutic tool to treat auto-immune diseases in the future.

**Disclosure of Interest**

None Declared.

### P225 Diagnostic and prognostic value of synovial fluid in paediatric juvenile idiopathic arthritis

#### Sajida Rasul^1^, Alice Chieng^2^, Tony Freemont^3^

##### ^1^Paediatric Rheumatology, Royal Manchester Childrens’ Hospital, Manchester, UK; ^2^RMCH, Manchester, UK; ^3^Division of Cell Matrix Biology and Regenerative Medicine, University of Manchester, Manchester, UK

###### **Correspondence:** Sajida Rasul

**Introduction:** Juvenile idiopathic arthritis (JIA) is a term that encompasses a group of arthritides that vary in pattern of joint inflammation and presentation (1). The distinct characteristics found in these subtypes are likely to be a result of genetic and environmental variations. Each subtype has been found to respond differently to the different DMARD and biologic regimes currently available, based on the unique immunological mechanism involved in each subtype. New evidence shows use of proteins found in serum, as bio and prognostic markers for some JIA subtype, such as MMP-3 in ERA (2) and IL-18 predicting treatment response in systemic JIA (3). More recent studies have found a correlation between serum cytokine and chemokines and synovial fluid (SF) composition (4). Further, studies have shown results suggesting synovial cellular composition and gene expression are predictive factors for the extension of oligo-articular JIA in around 30 patients (5).

SF cellular composition has a long established use in differentiating between inflammatory and infective arthritis in adults, due to a firm understanding that cell count correlates directly with final diagnosis (6). Although we are increasingly finding new patterns of protein expression in SF of children with JIA there has been no satisfactory documentation of a diagnostic cell count profile similar to that available for adults. Current SF analysis in paediatrics uses adult reference ranges, which does not take into account the differing disease process in JIA compared with adult onset arthritis. We hypothesise that cytoanalysis of synovial fluid from children with JIA can be used to diagnose which subtype of JIA the patient will develop and further provide prognostic value. This study will look at the cytoanalysis of 200 patients with JIA and compare the subtypes of each patient and their SF cell composition. We aim to demonstrate a clear pattern of cell composition, which is distinct between the subtypes, thus providing a reference for paediatric cytoanalysis of SF that can be used as a tool in establishing the final diagnosis.

**Objectives:**

1. To examine the cellular composition of the synovial fluid of patients with JIA

2. To present characteristic features in synovial fluid for each subtype of JIA

3. To look at prognostic value of synovial fluid cytology with clinical disease patterns like ANA and HLAB27 positivity, presence of uveitis, and diseases extension

**Methods:** Collection of SF cytology results from the Osteoarticular Unit archives and retrospective collection of clinical data from case notes. The diagnosis of all patients was reclassified using the ILAR classification. Children diagnosed with septic arthritis, vasculitis and those with mechanical joints were excluded from the study. All SF were taken before treatment with DMARD and biologics.

**Results:** SF cytology for 200 patients were studied. We have complete clinical data and SF analysis in 88% of the patients. In SOJIA, the SF revealed higher to total WCC. Polymorphs were more abundant in the SF of oligo-articular persistent compared with oligo-extended patients. Further significantly fewer ragocytes were found in patients with persistent olig arthritis as compared with the extended subtype. This suggests the presence of ragocytes could be used to predict the prognosis of patients presenting with oligo-articular arthritis (Table 11).

**Conclusion:** From our results we are able to present a distinct pattern of SF characteristics which differentiates between the JIA subtypes. The large number of patients and robust analysis of cellular composition has allowed us to comprehensively document the SF features to an extent that could allow us to use cyto-analysis of SF as a tool for diagnosis. Further we have presented evidence of the prognostic value of cytoanalysis of SF, particularly looking at the pattern of ragocytes.

**Disclosure of Interest**

None Declared

**Table 11 (Abstract P225).** See text for description

JIA Subtype	White Cells (%)			
	Polymorphs	Lymphocytes	Monocytes	Ragocytes
Oligoarthritis (inc. extended) n = 80	28 (4.5-62)	37 (9-65)	4.5 (1.5-10)	5 (0-30)
Polyarticular n = 22	33 (0-82)	16 (2-16)	3 (1-14)	35 (15-50)
Systemic n = 10	78 (76-79)	13 (3-14)	4 (2-10)	5 (0-9)
Enthesitis n = 18	35 (3-69)	59 (21-67)	0 (0-7)	0 (0-0)
Psoriatic n = 14	34 (2-85)	19 (5-63)	12 (8-38)	0 (0-5)
Reactive n = 32	55 (0-87.5)	2.5 (0-14.5)	3 (0-9)	10 (0-47.5)
Total 176				

.

### P226 Neutrophils play a key role in the initial inflammatory cascade in systemic juvenile idiopathic arthritis, displaying a sepsis-like phenotype

#### Nienke Ter Haar^1,2^, Wilco de Jager^1^, Rianne Scholman^1^, Jenny Meerding^1^, Michal Mokry^1^, Bas Vastert^1,2^, Sytze de Roock^1,2^

##### ^1^Laboratory for Translational Immunology, UMC Utrecht, Utrecht, Netherlands; ^2^Pediatric Rheumatology & Immunology, UMC Utrecht, Utrecht, Netherlands

###### **Correspondence:** Nienke Ter Haar

**Introduction:** Systemic onset Juvenile Idiopathic Arthritis (sJIA) is an acquired autoinflammatory disease, characterized by arthritis, spiking fever, rash and elevation of serum S100-proteins and interleukin (IL)-18, reflecting an IL-1 pathway activation. Although it is known that sJIA patients with active disease often display marked neutrophilia, the role of neutrophils in sJIA is yet to be unravelled.

**Objectives:** To dissect the role of neutrophils in the inflammatory cascade of sJIA.

**Methods:** Clinical lab values were collected of 50 new-onset sJIA patients at onset and during therapy, whom most were treated with hrIL1RA (anakinra) monotherapy. In a subgroup of 23 patients, serum levels of S100A12 and Mrp8/14 were analysed by ELISA and other immune compounds by Luminex multiplex immunoassay. RNA-sequencing of peripheral blood derived neutrophils from 3 sJIA patients with active disease without medication and 3 healthy donors (HDs) was performed. Further exploration of neutrophil phenotype and function was assessed in 9 sJIA patients with active disease, 9 patients in remission and 20 HDs. The percentage of immature neutrophils, defined as granulocytes with banded nucleus and decreased CD16 expression, was calculated. Surface marker expression of activation markers *ex vivo* and ROS production and release of secretory vesicles after *in vitro* stimuli were measured by flow cytometry

**Results:** Neutrophil counts were elevated in 40/50 patients at onset, and these levels decreased within days after the start of anakinra to return to normal ranges in all patients with clinically inactive disease. Neutrophil counts correlated to inflammatory markers CRP (R^2^ = 0.335, p < 0.001), ferritin, (R^2^ = 0.308, p < 0.001), Mrp8/14 (R^2^ = 0.470, p = 0.003) and S100A12 (R^2^ = 0.276, p = 0.037) Further, neutrophil related proteins as neutrophil collagenase (p < 0.001), sICAM (p < 0.001), E-selectin (p = 0.002) and elastase (p = 0.005) were significantly increased in onset versus remission.

RNA-sequencing revealed a distinct transcriptome in active sJIA neutrophils compared to HDs, with specific upregulation of immune-related processes. sJIA neutrophils showed increased expression of genes encoding for S100-proteins, inflammasome components, NFκB-pathway, IL1- and IL18-related proteins. As IPA analysis identified LPS as the most significant upstream regulator of the differentially expressed genes, we compared our dataset to two known sepsis gene sets. Indeed, a remarkably strong enrichment was observed (NES = 3.6-3.9, p < 0.001), which suggests a profound overlap between the transcriptome of neutrophils from sJIA and sepsis patients.

The percentage of immature neutrophils was significantly higher in active sJIA, which is in line with findings in sepsis patients (p = 0.001). The expression of CD64, known to increase after stimulation and CD35, a marker of secretory vesicle release, were significantly increased in active sJIA compared to HDs (p = 0.004 and p = 0.010, respectively). CD62L, which is shed upon activation, was decreased in active sJIA (p = 0.014). A significant increase in the release of secretory vesicles (p = 0.010) and a trend for increased ROS production (p = 0.081) after stimulation with fMLP was observed in active sJIA. In all abovementioned experiments, neutrophils from sJIA patients in remission demonstrated normal phenotype and function, comparable to HDs.

**Conclusion:** We show here that the neutrophils of sJIA patients with active disease display an upregulated inflammatory transcriptome, increased expression of activation markers and higher release of secretory vesicles and intracellular ROS production. Altogether, our data show that sJIA neutrophils have a similar phenotype as observed in bacterial sepsis patients, suggesting that neutrophils play a key role in the initial systemic inflammatory cascade of sJIA.

**Disclosure of Interest**

None Declared.

### P227 A novel small molecule blocks IL-17-dependent, tcr-independent inflammatory pathway of synovitis in juvenile idiopathic arthritis

#### Ian D. Ferguson^1,2^, Patricia Griffin^1^, Joshua J. Michel^1^, Sarah A. Gaffen^3^, Jon D. Piganelli^4^, Margalit E. Rosenkranz^1,5^, Daniel A. Kietz^1,5^, Abbe N. Vallejo^5,6^

##### ^1^Pediatrics, University of Pittsburgh School of Medicine, Pittsburgh, PA, USA; ^2^Pediatrics, Yale New Haven Hospital, New Haven, CT, USA; ^3^Medicine and Immunology, University of Pittsburgh School of Medicine, Pittsburgh, PA, USA; ^4^Surgery, University of Pittsburgh School of Medicine, Pittsburgh, PA, USA; ^5^Division of Pediatric Rheumatology, Children’s Hospital of Pittsburgh, Pittsburgh, PA, USA; ^6^Pediatrics and Immunology, University of Pittsburgh School of Medicine, Pittsburgh, PA, USA

###### **Correspondence:** Abbe N. Vallejo

**Introduction:** The identity of antigenic trigger(s) of T cell-dependent autoimmunity in Juvenile Idiopathic Arthritis (JIA) has remained elusive. Nevertheless, T cells are major effectors in the perpetuation of synovitis. Unraveling inflammatory pathway(s) elicited by joint-infiltrating T cells therefore remains paramount.

**Objectives:** Biological evaluation of the synovial environment is a sound approach towards an improved understanding of disease activity in inflammatory arthritides. Here, we examined a T cell-mediated, but TCR-independent, inflammatory pathway underlying synovitis in JIA.

**Methods:** Research protocols were approved by Institutional Review Boards of the University of Pittsburgh. Synovial fluid (SF) samples were obtained from children with oligoarticular or RF^neg^ polyarticular JIA who were undergoing arthrocentesis. Blood samples from healthy children, and JIA patients were also obtained as internal reference for cell phenotyping. Cell-free SF and plasma were used in multiplex humoral profiling, and the corresponding cell preparations were used in multicolor cytometry. Receptor cross-linking bioassays were performed using SF mononuclear cells, and molecular outcomes were examined in multiplex platforms.

**Results:** Humoral profiling of SF showed dominance of five cytokines including IL-17. Cell phenotyping showed predominance of T cells doubly negative (DN) for CD4 and CD8 coreceptors, and also lacked expression of the CD28 costimulatory molecule. These unusual T cells expressed high levels of CD31, an adhesion molecule known to facilitate transmigration of innate cells into sites of injury. Bioassays using CD31^+^ CD28^null^ DN T cells isolated from SF showed CD31 ligation alone was sufficient to induce intracellular expression of the same cytokines observed in SF humoral profiling. These bioassays also showed phosphorylation of four signaling intermediates, and the direct *trans*-activation of *IL-17* gene promoter. Such cellular outcomes were blocked by a novel oxidoreductase mimic, which as originally designed as scavenger of tissue-destructive superoxide. In addition to DN T cells, phenotyping of SF cells showed abundance of fibrocyte-like cells (FLC). FLC expressed high levels of IL-17 receptor A (IL-17RA) and CD38, a ligand of CD31. Exposure of FLC to recombinant IL-17 resulted in the induction of several downstream effectors including TNFα, IL-6, and MMPs. These IL-17-mediated responses by FLC were dampened by the oxidoreductase mimic comparable to, and in many cases, better than those seen with the biologics IL6i and TNFi.

**Conclusion:** CD31^+^ CD28^null^ DN T cells are a unique subset of cells that shape the inflammatory environment of arthritic joints in JIA. These cells may interact with non-hematopoetic CD38^+^IL17RA^+^ FLC, creating a local inflammatory circuit that is IL-17-mediated, but totally independent of TCR triggering. Interruption of this DN T cell-FLC communication by an oxidoreductase mimic indicates inflammatory signaling in marked departure from the autoantigen paradigm. The data also provide rationale for translational efforts to explore a potential therapeutic application of this oxidoreductase mimic as a DMARD for JIA. [Supported by IRG Award from the Nancy E Taylor Foundation for Chronic Diseases].

**Disclosure of Interest**

None Declared.

## Immunoregulation and basic science

### P228 Toll-like receptor 2 (TLR2) ARG753GLN and Toll-like receptor 4 (TLR4) THR399ILE polymorphisms in children with rheumatic fever in Latvia

#### Marina Visnevska^1^, Valda Stanevica^1^, Vita Rovite^2^, Zane Davidsone^1^, Andrejs Scegolevs^3^, Ruta Santare^3^

##### ^1^Riga Stradins University, Riga, Latvia; ^2^Latvian Biomedical Research and Study Centre, Riga, Latvia; ^3^Children’s Clinical University Hospital of Latvia, Riga, Latvia

###### **Correspondence:** Marina Visnevska

**Introduction:** Rheumatic fever (RF) is an autoimmune disease which follows group A streptococcal infection, leading to the diverse clinical manifestations - most frequently arthritis (75%) and Rheumatic heart disease (RHD 30–45%) (Guilherme L, 2015). The pathogenesis of RF/RHD is complex and recent interest has been focused on the innate immune system. Toll-like receptors (TLRs) have an important role in microbial recognition and subsequent innate immune response. The single-nucleotide polymorphisms (SNPs) in TLR2 and TLR4 genes have been shown to be associated with risk of RF. The genotype of TLR2 Arg753Gln was more frequent in a Turkish RF cohort when compared to controls (Berdeli et al., 2005). However, a more recent study was not able to confirm this strong association in patients with RHD of the same ethnic background (Duzgun N, 2007).

**Objectives:** To investigate the TLR2 gene Arg753Gln (rs5743708) and the TLR4 gene Thr399Ile (rs4986790) polymorphisms in a group of patients with RF in Latvia.

**Methods:** To assess the role of TLR2 gene and TLR4 gene polymorphisms in RF etiopathology, 70 children (diagnosed with RF in 1995-2001) and 293 healthy adult controls selected from the Genome Data Base of Latvian population were studied. The RF diagnosis was confirmed according to Jones criteria. SNP genotyping was performed using TaqMan hybridization probe SNP genotyping assay. Statistical analysis was performed with Plink 1.90. All genotyped frequencies of rs5743708 and rs4986790 in controls and patients conformed to the Hardy–Weinberg equation (p = 0.3507, p = 0.6202) and initial SNP genotyping efficiency was over 98.8%.

**Results:** 48 out of 70 RF patients (68.5%) were boys, 22 (33.4%) were girls. Up to 7 years of age were 23 (32.8%) and after - 47 (67.1%) patients. Acquired RHD developed in 47 (67.1%) patients. Polyarthritis was diagnosed in 34 (48.6%) patients. Gender proportion in control group was 186 (63.5%) men and 107 (36.5%) women. There were no statistically significant difference between TLR2 polymorphism rs5743708 and TLR4 polymorphism rs4986790 and control group corresponding p values; p = 0.604 and p = 0.226, odds ratios; OR = 0.836 and OR = 1.551. Allele distribution of rs5743708 in RF patients were AA – 1, AG – 9 and GG – 59, and 2/51/240 respectively in controls, having rare allele frequency 0.079 in cases and 0.094 in controls. But polymorphism rs4986790 were more frequently observed in cases 0.079 compared to control group 0.053, however, this difference did not reach statistical significance. Allele distribution for rs4986790 was GG – 0, GA – 11 and AA – 58 in RF cases and 0/31/262 correspondingly in controls.

**Conclusion:** This study investigating TLR2 and TLR4 gene polymorphisms in patients with RF in Latvia showed the lack of genetic association of the TLR2 Arg753Gln and TLR4 Thr399Ile SNPs variants with the susceptibility to RF, however, more detailed phenotyping of cases and increased RF subject group could help elucidate TLR influence on RF clinical manifestations.

**Disclosure of Interest**

None Declared.

## JIA - Outcome measures

### P229 Validating JADAS10 cut-off values for inactive disease, low disease and moderate disease activity levels in non-systemic juvenile idiopathic arthritis: a finnish multicentre study

#### Maria Backström^1^, Pirjo Tynjälä^2^, Heikki Ylijoki^3^, Kristiina Aalto^4^, Anne Putto-Laurila^5^, Minna-Maija Grönlund^5^, Johanna Kärki^6^, Heini Pohjankoski^7^, Paula Keskitalo^8^, Sirja Sard^9^, Maiju Hietanen^7^, Silke Witter^10^, Helena Lehto^11^, Eliisa Löyttyniemi^12^, Paula Vähäsalo^9^

##### ^1^Department of Pediatrics, Vaasa Central Hospital, Vaasa, Finland; ^2^Poison Information Center, Helsinki University Central Hospital, Helsinki, Finland; ^3^Department of Pediatrics, Satakunta Central Hospital, Pori, Finland; ^4^Children’s Hospital, Helsinki University Central Hospital and University of Helsinki, Helsinki, Finland ; ^5^Department of Pediatrics, Turku University Hospital, Turku, Finland ; ^6^Department of Pediatrics, Kanta-Häme Central Hospital, Hämeenlinna, Finland; ^7^Department of Pediatrics, Päijät-Häme Central Hospital, Lahti, Finland; ^8^Medical Research Center Oulu, Department of Children and Adolescents, PEDEGO Research Unit, Oulu University Hospital and University of Oulu, Oulu, Finland; ^9^Medical Research Center Oulu, Department of Children and Adolescents, PEDEGO Research Unit, Oulu University Hospital and University of Oulu, Oulu, Finland; ^10^Department of Pediatrics, Central Finland Central Hospital, Jyväskylä, Finland; ^11^Department of Pediatrics, South-Karelian Central Hospital, Lappeenranta, Finland; ^12^Department of Biostatistics, University of Turku, Turku, Finland

###### **Correspondence:** Maria Backström

**Introduction:** Earlier we established 10 joint juvenile arthritis disease activity score (JADAS10) cut-off levels for inactive disease (ID), low disease activity (LDA) and moderate disease activity (MDA) [1]. These were slightly different from cut-off levels proposed by Consolaro et al. [2].Several definitions of high disease activity (HDA) in juvenile idiopathic arthritis (JIA) exist [3-5].

**Objectives:** The aim was to validate previously established JADAS10 cut-off levels [1] and compare them to the cut-off values suggested by Consolaro et al. [2]. Another aim was to analyze the proportions of HDA patients in a random JIA population when using the criteria defined by Bulatovic´ C´alasan et al. [4] and Consolaro et al. [5].

**Methods:** Data on the recent visit of the patients diagnosed with non-systemic JIA during two years (n = 337) was collected. Area under the ROC-curve (AUC) was determined. Correct classification rates (CCR) and too high and too low classification rates were counted using our previously defined cut-off values [1] and, for comparison, the cut-off values proposed by Consolaro et al. [2].

**Results:** Based on relatively high CCRs, the cut-offs validated in this study seemed feasible. Changing the cut-off value for LDA to 1.0 in both oligoarthritis and polyarthritis, as suggested by Consolaro et al. [2], lowered the correct classification rate of LDA and led to increase in the number of patients with LDA classified as having ID (Table 12).

In this population only 1 visit fulfilled the criteria for HDA according to Beukelman et al. [3] whereas 20 patients fulfilled the criteria for HDA according to Bulatovic´Casalan et al. [4], and 46 patients fulfilled the criteria for HDA according to Consolaro et al. [5]. Of these 46 patients 20% were categorized as LDA according to Beukelman et al. [3].

**Conclusion:** When establishing cut-off values for disease activity in non-systemic JIA, it is important that the sensitivity of the values is high enough to detect active patients from those in remission. We suggest that in non-systemic JIA, the lower JADAS10 cut-off value for ID, 0.5 in oligoarticular and 0.7 in polyarticular disease should be used. The present study also points out a need to define new uniform criteria of HDA. It is of great importance that the criteria for the disease activity levels are as objective as possible so that the levels can be used to compare different populations.

**Disclosure of Interest**

None Declared.

**Table 12 (Abstract P229).** Validation of JADAS10 cut-off values for disease activity levels of JIA based on Backström et al. [1], compared with the cut-off values suggested by Consolaro et al. [2]

		Oligoarticular disease				Polyarticular disease		
	AUC	JADAS10 Selected cut-offs	Correct classification rate%	Too low/ Too high %	AUC	JADAS10 Selected cut-offs	Correct classification rate%	Too low/ Too high %
Inactive disease		0.5 [1] 1.0 [2]	74.4 83.9	0/25.6 0/14.9		0.7 [1] 1.0 [2]	65,3 76.4	0/34.7 0/23.6
Low disease activity	0.83	0.6-2.7 [1] 1.1-2.0 [2]	40.9 22.7	27.3/31.8 34.1/43.2	0.83	0.8-3.9 [1] 1.1-3.8 [2]	63.6 54.5	12.1/24.2 21.5/24.2
Moderate disease activity	0.94	>2.7 [1] >2.0 [2]	92.5 98.1	7.5/0 1.9/0	0.89	>3.9 [1] >3.8 [2]	81.3 81.3	18.7/0 18.7/0

### P230 A literature review of outcome measures used in juvenile idiopathic arthritis trials- as part of the steroid induction of remission in JIA (SIRJIA) study

#### Samundeeswari Deepak^1^, Eileen Baildam^2^, Flora McErlane^1^ and HTA 14/167/01 SIRJIA Trial Management Group

##### ^1^Paediatric Rheumatology, Great North Children’s Hospital, Newcastle, UK; ^2^Paediatric Rheumatology, Alderhey Children’s Hospital, Liverpool, UK

###### **Correspondence:** Samundeeswari Deepak

**Introduction:** Corticosteroids (CS) are key to achieving rapid disease control in children and young people (CYP) presenting with new or flaring juvenile idiopathic arthritis (JIA). Current routes of CS administration are based on physician and patient preference, rather than scientific evidence. A clinical trial is needed to ascertain the most effective routes and doses of CS in JIA.

As part of a feasibility study of CS induction regimes in JIA, we report a literature review exploring outcome measures cited in JIA clinical trials involving CS. To date, it is not clear whether the outcomes chosen in JIA trials are meaningful and important to patients. The review will inform focus group work including consumers and health care professionals, to derive appropriate primary and secondary outcome measures for the study.

**Objectives:** To identify the range of primary and secondary outcome measures reported in JIA studies involving CS.

To assess the involvement of patients and/or carers in the choice of primary outcomes for studies involving CS.

**Methods:** Initial searches were performed in: CINAHL, Cochrane library, EMBASE, Medline, Trip database, International Clinical Trials Registry, UK CRN study Portfolio, and the Australian Clinical Trials Register. Eligibility criteria included articles published in English between 2000 and May 2016 where JIA treatment included CS.

**Results:** Of the 101 articles screened, 49 had relevant information on the outcome measures. Common primary outcome measures included single measures of disease activity (active joint count, restricted joint count, joint swelling, ESR, physician/parent reported global scores), composite measures of disease activity (JIA core outcome variables (COVs), ACR 30 or 70), number of flares, pain, medication use, function (CHAQ) and quality of life assessments.

Secondary outcomes were more variable. Examples included COVs, duration of morning stiffness, JADAS71 and minimal disease activity (MDA) at 1 year, recurrence of joint injections. Other measures were study specific, including circumference of thighs or knee, range of movements (in degrees), isokinetic strength of knee flexion and extension and gait dynamics and analysis, jaw pain intensity, localisation, dysfunction/maximal incisal opening (MIO) distance,, perceived improvement in jaw appearance.

In children with systemic JIA, multiple indicators of disease activity were employed as outcome measures (absence of arthritis, fever, rash, serositis, and generalised lymphadenopathy).

There was no evidence that consumers had informed the choice of primary/secondary outcome in any of the studies reviewed.

**Conclusion:** The heterogeneity of primary and secondary outcomes reported in JIA studies including CS suggest lack of consensus as to meaningful and relevant measures. Although there is a move to using agreed standardised outcomes in clinical trials of JIA, the optimal set of outcomes is likely to vary with JIA subtypes and type of intervention being studied.

To date, patient involvement in selection of outcome measures for studies involving CS in JIA appears to be limited. Patient involvement is a fundamental requirement for the Core Outcome Measures in Effectiveness Trials (COMET) initiative. Our literature review suggests more work is needed to inform future studies of CS regimes in JIA and measures to improve consumer involvement in study design and outcome selection.

**Trial registration identifying number: HTA:14/167/01**

**Disclosure of Interest**

None Declared.

### P231 Satisfaction with current condition in juvenile idiopathic arthritis is linked to morning stiffness but not to burden of therapy

#### Emanuela Del Giudice, P. van Dijkhuizen, S. de Roock, S. J. Vastert, S. F. Swart, N. M. Wulffraat

##### Paediatric Rheumatology, Wilhelmina Children’s Hospital, University Medical Center Utrecht, Utrecht, Netherlands

###### **Correspondence:** Emanuela Del Giudice

**Introduction:** Patient reported outcomes are increasingly being recognized as important instruments in the assessment of juvenile idiopathic arthritis (JIA) disease course. Analysis of these outcomes gives insight into factors that are important to patients and which should therefore have a bearing on the treatment decisions in the management of JIA.

**Objectives:** To identify factors that are associated with patient reported satisfaction in JIA.

**Methods:** A cross-sectional cohort of 239 anonymized JIA patients enrolled in PharmaChild in our centre and who completed at least one juvenile arthritis multidimensional assessment report (JAMAR) was evaluated. In case a patient completed more than 1 JAMAR, the first was analysed. Primary outcome was the patient’s satisfaction with their current condition.

First, every item of the JAMAR, as well as JIA subtype, demographics and disease activity parameters was analysed in univariate analysis, using the primary outcome. Secondly, a multivariable logistic regression analysis was performed using all variables with a significant univariate relationship (defined by p <0.05).

**Results:** The majority of patients was female (150, 62.8%). The median (interquartile range, IQR) age at JAMAR completion was 13 years (9.9, 15.9), the median (IQR) disease duration 5 years (2.3, 8.1) and 34.3% was affected by oligoarthritis according to ILAR criteria. The median (IQR) clinical juvenile arthritis disease activity score (cJADAS)-10 was 1.5 (0.0, 6.4). About one half of patients (126, 52.7%) judged themselves in remission and 191 (79.9%) were on therapy.

Of 239 patients, 141 (59.0%) were satisfied with their current condition. Univariate analysis identified many variables with a significant association with the patient’s satisfaction: age at disease onset, disease duration, age at completion of the JAMAR; physician’s global assessment of disease activity (PGA), number of active and limited joint evaluated by the physician, JADAS-10, JADAS-71 and cJADAS-10; patient VAS pain, VAS disease activity and VAS well-being; patient self-assessment of joint involvement, presence of neck or back involvement according to the patient, presence and duration of morning stiffness, the current state and course of patient’s illness.

Moreover, the sum of functional disabilities was associated with the patient’s satisfaction. Few patients expressed difficulties putting their hands behind their neck (13, 5.4%) or biting in a sandwich or apple (15, 6.3%) and these were not associated with satisfaction. The presence of problems at school caused by the illness and the sum of the items of quality of life were also associated with the patient’s satisfaction.

Dissatisfied patients were more frequently on therapy (88.8% vs. 73.8%, p = 0.007), specifically NSAIDs (52.0% vs. 27.0%, p = 0.004). Interestingly, being on methotrexate therapy was not associated with satisfaction (p = 0.416). Moreover, the presence of disturbances caused by medication showed only a borderline association (p = 0.052).

Due to correlations among the variables, many could not be included in the logistic regression. The final model included the following predictors: age at completion of the JAMAR, age at disease onset, disease duration, morning stiffness, physician assessment of limited joints, being on therapy, NSAIDs therapy, presence of disturbances caused by medications, problems at school and patient VAS well-being.

Only morning stiffness and VAS well-being resulted significant predictors in the model (p = 0.001 and p = 0.005, respectively).

**Conclusion:** The results underline the influence of disease activity and the quality of life on the patient’s satisfaction. Contrariwise, therapy (especially methotrexate) and its side effects were not associated with the outcome. These findings support the idea to adapt the standard of care for JIA patients and aim at a rapid induction of disease remission and consequently an improvement of the quality of life, by early and aggressive treatment.

**Disclosure of Interest**

None Declared.

### P232 Burden of disease, therapy and phenotypes of juvenile psoriatic arthritis – data from the national paediatric rheumatologic database (NPRD) over a 15-year period

#### Nina Derksen^1^, Martina Niewerth^1^, Claudia Sengler^1^, Nils Geisemeyer^1^, Rolf M Küster^2^, Ivan Foeldvari^3^, Johannes-Peter Haas^4^, Kirsten Minden^5^

##### ^1^Epidemiology Unit, German Rheumatism Research Center, Berlin, Germany; ^2^Orthopädie Zentrum Altona, Hamburg, Germany; ^3^Kinderrheumatologische Schwerpunktpraxis, Hamburg, Germany; ^4^Deutsches Zentrum für Kinder- und Jugendrheumatologie, Garmisch-Partenkirchen, Germany ^5^Charité - Universitätsmedizin Berlin, Berlin, Germany

###### **Correspondence:** Nina Derksen

**Introduction:** According to the ILAR classification, the criteria for juvenile psoriatic arthritis (PsA) are met when arthritis is accompanied either by psoriasis or at least by two of the following: dactylitis, nail abnormalities, first degree relative with psoriasis diagnosed by a physician. Juvenile PsA is a complex disease, and appropriate management is correspondingly complex.

**Objectives:** This study aimed at assessing disease burden, therapy, and phenotypes of PsA at different time points across 15 years.

**Methods:** Cross sectional data of patients with PsA recorded in the NPRD in the years 2000, 2007 (PsA add-on module with specific items concerning beginning and manifestations of the disease), and 2015 were considered. Demographic and clinical parameters, including cJADAS-10, CHAQ and therapy, in different years were compared. Disease characteristics in patients with oligoarticular (<5 joints) vs. polyarticular (≥5 joints) as well as early (age ≤ 5 years) vs. late (age >5 years) disease onset were analysed by data of the psoriasis module.

**Results:** In total, data from 165, 250 and 539 PsA cases, recorded in the year 2000, 2007 (PsA module) and 2015, respectively, could be analysed. No relevant differences in demographics were found. The proportion of patients with inactive disease (cJADAS-10 ≤ 1; 2000: 23%, 2007: 39%, 2015: 35%) and full functional capacity (CHAQ = 0; 48%, 56%, 55%) increased over time, as well as the prescription rate of biologics (2%, 15%, 23%).

Via the PsA module, 144 patients with an oligoarticular disease onset (ODO) were documented, who did not differ from the 81 patients with polyarticular onset (PDO) in regard to HLA-B27 and ANA positivity or median age of disease onset. ODO patients had significantly more often firstdegree relatives with psoriasis, PDO patients had, however, more frequently a dactylitis at disease onset. The knee joint was the most commonly affected joint in patients with ODO, while in PDO patients the small joints of the hands and feet were in addition frequently involved.

The 95 patients with early onset of PsA (EO, mean age at diagnosis 2.7 years) differed significantly from the patients with late onset (LO, n = 142, mean age at diagnosis 11.2 years) regarding ANA status (67% vs. 27% ANA+), history of uveitis (17% vs. 6%) and dactylitis (62% vs. 33%). The LO patients suffered more often from enthesitis (30% vs. 9%), inflammatory back pain (17% vs. 3%) than patients with EO. Also LO patients had more often psoriasis in the course of their disease than EO patients (44% vs. 18%). The most commonly affected joints at disease beginning were knee, metatarsophalangeal and other toe joints in EO patients whereas the wrist is mostly affected in LO patients, respectively.

**Conclusion:** Juvenile PsA is a heterogeneous disease with diverse joint involvement patterns and extraarticular manifestations, their consideration may be crucial for tailored treatments and good outcomes.

The National Paediatric Rheumatologic Database is partially funded by the German Children Rheumatism Foundation and AbbVie.

**Disclosure of Interest**

None Declared.

### P233 Validation of biomarkers to predict flare in polyarticular JIA upon stopping anti-TNF therapy

#### Daniel Lovell^1^, Sarah Ringold^2^, Scott Eastman^3^

##### ^1^Rheumatology, Cincinnati Children’s Hospital Medical Center, Cincinnati, OH, USA; ^2^Rheumatology, Seattle Chidren’s Hospital, Seattle, WA, USA; ^3^Crescendo Bioscience, South San Francisco, CA, USA

###### **Correspondence:** Daniel Lovell

**Introduction:** Currently more than 40% of children with polyarticular forms of Juvenile Idiopathic Arthritis (JIA) experience clinical inactive disease for at least 6 continuous months- clinical remission on medication (CR). No clinical variable or biomarker has been identified to predict which patients in CR can effectively stop anti-TNF biologic therapy.

**Objectives:** To evaluate the association between clinical parameters and lab biomarkers with disease flare within 10 months (mos) in JIA patients in CR who stop anti-TNF therapy.

**Methods:** In a 16 center, prospective protocol driven clinical trial, 106 JIA pts. (polyarticular RF+/RF- and extended oligoarticular) experienced CR while on treatment with etanercept, infliximab or adalimumab (anti-TNF). The anti-TNF therapy was stopped, all other meds remained stable, and patients were followed prospectively for up to 10 mos. Flare was determined by prespecified criteria. At the time of stopping anti-TNF therapy, serum was drawn for this analysis in 104 pts. Samples were analyzed for the following 12 individual biomarkers included in the multi-biomarker disease activity score (MBDA; Vectra DA) using multiplexed immunoassay methods previously described: vascular cell adhesion molecule-1 [VCAM-1], epidermal growth factor [EGF], vascular endothelial growth factor A [VEGF-A], interleukin-6 [IL-6], tumor necrosis factor receptor 1 [TNF-R1], matrix metalloproteinase-1 [MMP-1], matrix metalloproteinase-3 [MMP-3], human cartilage glycoprotein-39 [YKL-40], leptin, resistin, serum amyloid A [SAA], and CRP (1,2). The Vectra DA score (range 1 to 100) was calculated with a validated algorithm. Six additional biomarkers were also assayed: interleukin-8 [IL-8], interleukin-1B [IL-1B], macrophage-derived chemokine [MDC], interleukin-6 receptor [IL-6R], intercellular adhesion molecule 1 [ICAM-1] with a custom multiplex assay (MSD, Bethesda, MD). S100A12 levels in serum were determined by a double sandwich ELISA system as previously described (3,4). Univariate analysis with Students t-test for significance and multivariate discriminant analysis was used to calculate relationship of clinical and biomarker variables with risk for flare.

**Results:** Overall, 39/106 (37%) of the pts. flared in the follow-up period. The mean/median/SEM for follow-up for those that flared during the study was 286/287/11.4 days and for that did not flare 443/438/3.4 days. The mean/median/SEM for time to flare was 212/250/9.8 days. The univariate analysis demonstrated the correlations of the parameters with flare/no flare status (Table 13). Multivariate discriminant analysis identified a model that includes 7 variables listed in decreasing F ratio- MMP-3, JIA disease duration, age at time of JIA diagnosis, VCAM-1, Inactive Disease Duration, VEGF, and resistin. For this model the observed AUC for did not flare was 0.80, correct prediction in 79% of cases, sensitivity 79% (59/75), specificity 79% (22/28), PPV 91% (59/65), NPV 59% (22/38).

**Conclusion:** 37% of the pts. flared with 10 mos of stopping anti-TNF. The MBDA did not discriminate significantly flare/no flare in the multivariate analysis. A combination of clinical and lab biomarkers predicted not flaring within 10 mos accurately in 79% of cases.

References

1. Eastman S, et al. 2012 Journal of Pharmaceutical and Biomedical Analysis 70:415-424.

2. Curtis R, et al. 2012 Arthritis Care and Research 64:1794-1803.

3. Foell K, et al. 2003 Gut 52:847-53.

4. Frosch M, et al. 2000 Arthritis Rheum 43:628-37.

**Trial registration identifying number:** NCT00792233

**Disclosure of Interest**

D. Lovell: None Declared, S. Ringold Grant/Research Support from: Crescendo Bioscience, S. Eastman Employee of: Crescendo Bioscience.

**Table 13 (Abstract P233).** See text for description

Metric	p-value
Ln MMP-3	0.014
Ln Disease Duration	0.016
Age Diagnosis	0.037
VCAM-1	0.063
ID duration at baseline (yrs)	0.069
VEGF-A	0.21
Resistin	0.38
MBDA	0.97
Ln S100A12	1.00

### P234 Analysis of risk factors of ocular complications in children with juvenile idiopathic arthritis-associated uveitis

#### Valentina Muratore^1^, Serena Calandra^2^, Valentina Ravaschio^2^, Gabriella Giancane^2^, Alessandra Alongi^2^, Angela Pistorio^2^, Alessandro Consolaro^2^, Benedetta Schiappapietra^2^, Angelo Ravelli^2^

##### ^1^Fondazione IRCCS Policlinico San Matteo, Pavia, Italy; ^2^Istituto Giannina Gaslini, Genova, Italy

###### **Correspondence:** Valentina Muratore

**Introduction:** Juvenile idiopathic arthritis (JIA) is the most common disease causing chronic anterior uveitis in childhood. The onset of ocular inflammation is insidious and often asyntomatic; uveitis can worsen over time and lead to sight-threatening complications, such as posterior synechiae, band keratopathy, cataract, glaucoma, ocular hypotonia, phtisis bulbi, and cystoid macular edema. Despite recent advances in the treatment of JIA-associated uveitis, ophthalmologic disease still represents a common cause of eye-related morbidity.

**Objectives:** To investigate the frequency and risk factors of complications of uveitis in children with JIA.

**Methods:** A retrospective study was conducted on patients with JIA who were seen at study centers from January 1987 to October 2016. Inclusion criteria were a diagnosis of JIA based on ILAR criteria and a disease category of persistent oligoarthritis, extended oligoarthritis, RF-negative polyartrhritis, psoriatic arthritis and undifferentiated arthritis. Patients without uveitis who had a disease duration of less than 6 months were excluded. In all patients who developed uveitis, we reviewed all ophthalmologic evaluations to verify whether they had or did not have ocular complications, including posterior synechiae, band keratopathy, cataract, glaucoma, ocular hypotonia, phtisis bulbi, and cystoid macular edema. Predictors of ocular complications included age at onset of arthritis and uveitis, ILAR category, antinuclear antibody (ANA) status, disease duration at onset of uveitis, follow-up duration after diagnosis of uveitis and type of complication.

**Results:** A total of 1425 patients were included in the study; of them, 336 (23.6%) had uveitis. After a median of 2.1 years after the onset of uveitis, 104/336 (31.0%) patients with this condition developed at least one of these complications: posterior synechiae (n = 75; 22.3%), band keratopathy (n = 41; 12.2%), cataract (n = 40; 11.9%), glaucoma (n = 11; 3.3%), and cystoid macular edema (n = 12; 3.6%). The comparison of children with and without ocular complications showed that those with complications had a younger age at uveitis onset and a shorter disease duration before the onset of uveitis (p = 0.042 and p = 0.024, respectively). ILAR category and ANA status were comparable between patients with and without complications (P = 0.62 and P = 0.29, respectively). The age at onset of arthritis was also similar in the two groups (P = 0.73) (Table 14).

**Table 14 (Abstract P234).** See text for description

	Uveitis with complications (N = 104)	Uveitis without complications (N = 232)	p
Persistent oligoarthritis	58 (55.8)	128 (55.2)	P = 0.62^#^
Extended oligoarthritis	16 (15.4)	44 (19.0)	
RF-negative polyarthritis	25 (24.0)	48 (20.7)	
Psoriatic arthritis	0 (0.0)	4 (1.7)	
Undifferentiated arthritis	5 (4.8)	8 (3.5)	
Antinuclear antibody (ANA)^§^	95/102 (93.1)	210/219 (95.9)	P = 0.29
Age at the onset of uveitis, median (IQR), years	3.8 (2.5; 5.5)	4.3 (3.1; 6.5)	P = 0.042^##^
Disease duration at the onset of uveitis, median (IQR), years	0.8 (0.2; 1.9)	1.2 (0.2; 3.2)	P = 0.024^##^

^#^P: Fisher’s Exact test; ^§^undetermined values have been excluded; ^##^P: Mann-Whithney U test

**Conclusion:** Around one quarter of our patients with select categories of JIA developed uveitis. Children with a younger age at onset of uveitis and a shorter duration of articular disease before the onset of uveitis had a higher risk of developing ocular complications. This finding highlights the need for a particularly strict ophthalmologic monitoring in younger children and in the earlier disease stages.

**Disclosure of Interest**

None Declared

### P235 Discordance between child/parent assessment of well-being and pain and physician global assessment of disease activity in a multinational cohort of 9137 children with juvenile idiopathic arthritis

#### Ellen B. Nordal^1,2,3^, Alessandro Consolaro^3^, Rolando Cimaz^3^, Ruben Cuttica^3^, Fabrizio De Benedetti^3^, Yasser El Miedany^3^, Troels Herlin^3^, Maria Martha Katsicas^3^, Pierre Quartier^3^, Gordana Susic^3^, Soamarat Vilaiyuk^3^, Nico Wulffraat^3^, Alberto Martini^3^, Nicola Ruperto^3^, Angelo Ravelli^3^ and the EPOCA study group

##### ^1^Department of Pediatrics, University Hospital of North Norway, Tromsø, Norway; ^2^Department of Pediatrics, UiT The Arctic University of Norway, Tromsø, Norway; ^3^Pediatria II Reumatologia, Istituto Giannina Gaslini, PRINTO, Genova, Italy

###### **Correspondence:** Ellen B. Nordal

**Introduction:** The child/parent overall well-being and pain are essential patient-reported outcomes (PRO) in juvenile idiopathic arthritis (JIA).

**Objectives:** To study determinants and any discordance between child/parent overall well-being and pain and physician global assessment (GA) of disease activity in children with JIA.

**Methods:** The EPidemiology, treatment and Outcome of Childhood Arthritis (EPOCA) study is aimed to obtain information on the frequency of JIA subtypes in different geographic areas, the therapeutic approaches, and the disease and health status of children with JIA currently followed worldwide. More than 9137 children with JIA from 118 pediatric rheumatology centres in 49 countries were collected so far. We have investigated the determinants and any discordances of the GA visual analogue scale (VAS) (range 0-10) between child/parent and physician. The PRO was reported by the parent(s) in children <10 years of age, otherwise by the child. Discordance was defined as high for a VAS difference >3, moderate for a VAS difference >1 and ≤3, and in agreement for a VAS difference <1. Correlations was assessed with Spearman correlation coefficient r. The countries were divided in eight geographical areas.

**Results:** Child/parent and physician GA score was available in 8615/9137 (94.3%) of the EPOCA participants. Primarily pain, but also the health-related quality of life (HRQL), were determinants of the child/parent score of general well-being, while the most important determinants of the physician GA were the number of active joints, pain, ESR and morning stiffness. The overall correlation between the child/parent and the physician GA was moderate r = 0.54, but ranged from 0.28 to 0.94 in different countries. The correlation between the child/parent GA and the pain score was high, r = 0.75. The overall agreement between child/parent and physician GA was ≤1 in 5,218 (60.6%). When the child/parent GA was higher than the physician, the scores on pain and adverse effect on HRQL were significantly higher, also the number of children with morning stiffness was higher (Table 15). When the physician GA was higher than the child/parent, the active joint count was higher. The discordance was highest for JADAS scores >6. Geographical differences were found, with the highest proportion of discordantly higher child/parent scores compared to the physician in Northern and Central European regions.

Values are the median (1^st^, 3^rd^ quartile), unless indicated n (%). JADAS10, Juvenile arthritis disease activity score based on 10 joints; CGA, child/parent global assessment of overall well-being (range 0-10); PGA, physician global assessment of disease severity (range 0-10).

**Conclusion:** The child/parent overall well-being is mainly determined by pain, while the number of joints was a main determinant of the physician GA VAS. Worldwide differences in PRO and physician GA VAS determinants and discordances were found.

**Disclosure of Interest**

None Declared.

**Table 15 (Abstract P235).** Clinical characteristics of the cohort according to level of discrepancy between the child/parent global assessment of overall well-being (CGA), and physician global assessment of disease severity (PGA)

	Agreement	Low discrepancy		High discrepancy	
	CGA ≤1 PGA	CGA < -1 ≥ -3 PGA	CGA > 1 ≤ 3 PGA	CGA < -3 PGA	CGA >3 PGA
Age at visit (years)	11.3 (7.3, 14.7)	11.1 (7.1, 14.4)	12.2 (8.0, 15.4)	10.9 (6.7, 13.8)	12.8 (8.7, 15.7)
Active Joints	0 (0, 1)	1 (1, 4)	1 (0, 2)	2 (1, 6)	0 (0, 1)
Morning stiffness, n (%)	1162 (23.2)	415 (39.3)	549 (53.4)	181 (41.6)	494 (64.9)
Pain score	0.0 (0.0, 1.5)	1.0 (0.0, 3.0)	3.5 (1.5, 5.5)	1.0 (0.0, 3.5)	5 (2.5, 7.0)
JAMAR Quality of Life	2 (0, 5)	3 (1, 6)	7 (4, 10)	3 (1, 6)	9 (6, 13)
JADAS27	1.0 (0.0, 4.0)	6.2 (4.0, 11.0)	6.0 (2.5, 11.0)	10.6 (7.0, 16.0)	8.0 (5.0, 11.5)

### P236 Orofacial symptoms and oral health-related quality of life in JIA: a two-year observational follow-up study

#### Hanna Rahimi^1^, Marinka Twilt^2^, Lynn Spiegel^3^, Troels Herlin^1^, Bernd Koos^4^, Annelise Küseler^5^, Thomas K. Pedersen^1^, Peter Stoustrup^5^

##### ^1^Aarhus University Hospital, Århus, Denmark; ^2^Alberta Children’s Hospital, Calgary, Canada; ^3^The Hospital for Sick Children, Toronto, Canada; ^4^University of Tübingen, Tübingen, Germany; ^5^Aarhus University, Århus, Denmark

###### **Correspondence:** Hanna Rahimi

**Introduction:** Orofacial symptoms are common findings in patients with juvenile idiopathic arthritis (JIA). However, little is known about the chronicity of orofacial symptoms and how this influences the oral health-related quality of life (OHRQOL) in patients with JIA.

**Objectives:** 1) To study the long-term changes in self-reported orofacial symptoms, 2) To define the impact of orofacial symptoms on OHRQOL.

**Methods:** Consecutive patients were invited to participate in this study. Inclusion criteria were: 1) JIA diagnosis, 2) ≤20 years of age. At baseline (T0), the patients completed the euroTMjoint patient questionnaire that incorporates six domains related to the orofacial area. Among others, pain frequency was assessed using a 5 point Likert scale (0=“Never”, 1=“Less than once a week”, 2=“Several times a week”, 3=“Several times a day”, 4=“All the time”). A 100 mm VAS was used to assess orofacial functional disability (0=“Not affected”, 100=“Severely affected”).

At follow-up (T1), the patients completed the same items of the baseline questionnaire in addition to a validated 31-item questionnaire addressing OHRQOL, including global ratings of self-reported perception of oral health (5 point Likert scale, “Excellent” = 0 to “Poor” = 4), and the extent to which the orofacial conditions affected the overall well-being and general quality of life (5 point Likert scale, 0=“Not at all”, 4=“Very much”).

**Results:** At baseline, 157 consecutive patients completed the questionnaire. At the two-year follow-up (mean 24 months, SD 3.2 months), 111 patients repeated the questionnaire (response rate 71%). Only patients who completed the questionnaires at both time points were included in the analysis.

Baseline (T0): 53% (59/111) of the patients reported a presence of orofacial pain, and 36% (40/111) of the patients reported a disabled orofacial function. Baseline findings of excluded non-responders (n = 46) were comparable to the included group of patients.

Follow-up (T1): 76% (45/59) of the patients with orofacial pain at T0 still reported pain at T1. Changes were as follows: 29% (13/45) had less frequent pain, 40% (18/45) had comparable pain frequency, and 31% (14/45) had more frequent pain at T1. 65% (26/40) of the patients with orofacial functional disability at T0 also reported disability at T1. Changes were as follows: 27% (7/26) reported an improvement of orofacial functional disability, 42% (11/26) reported the same level of orofacial functional disability, and 31% (8/26) reported a worsening of orofacial functional disability at T1.

Global ratings of self-reported perception of oral health, and the extent to which the orofacial conditions affected the overall well-being and general quality of life were significantly reduced in patients in pain (p < 0.0001) compared to asymptomatic patients. 16% (10/61) of the patients with orofacial pain and/or functional disability reported that the condition had “Some” (Likert scale score 2) negative impact on the overall quality of life. 7% (4/61) reported that the orofacial condition reduced general quality of life “A lot” (1/61, score 3) and “Very much” (3/61, score 4).

**Conclusion:** This study shows: 1) Self-reported orofacial pain and functional disability were common findings in a cohort of JIA patients followed over two years. 2) These symptoms seem to persist over time in most patients. 3) Orofacial pain and functional disability significantly reduce OHRQOL. 4) General quality of life is substantially affected in 23% of the patients with orofacial symptoms.

**Disclosure of Interest**

None Declared.

### P237 Can we predict clinically inactive disease in patients with juvenile idiopathic arthritis?

#### Stephanie J. W. Shoop-Worrall^1,2^, Suzanne M. Verstappen^1^, Janet E. McDonagh^2,3,4^, Wendy Thomson^2,5^, Kimme L. Hyrich^1,2^ and CAPS

##### ^1^Arthritis Research UK Centre for Epidemiology, The University Of Manchester, Manchester, UK; ^2^NIHR Manchester Musculoskeletal BRU, CMFT and University of Manchester Partnership, Manchester, UK; ^3^Centre for MSK research, The University Of Manchester, Manchester, UK; ^4^Manchester Academic Health Science Centre, Manchester, UK; ^5^Arthritis Research UK Centre for Genetics and Genomics, The University Of Manchester, Manchester, UK

###### **Correspondence:** Stephanie J. W. Shoop-Worrall

**Introduction:** Identifying predictors of clinically inactive disease (CID) would allow stratified treatment decisions at diagnosis of juvenile idiopathic arthritis (JIA). This, in turn, would minimise the burden of unnecessary therapies. However, different groups of children are identified between Wallace’s preliminary criteria versus clinical Juvenile Arthritis Disease Activity Score using 10 joints (cJADAS10)) CID definitions. Thus, predictors may differ depending on which CID outcome is applied.

**Objectives:** To assess whether baseline core outcome variables for JIA predict achievement of CID according to Wallace’s preliminary criteria or the cJADAS10 cut-off in patients with JIA.

**Methods:** Children and young people who had registered to the Childhood Arthritis Prospective Study (CAPS), a UK multicentre inception cohort, before January 2011, were selected if diagnosed with oligoarticular, rheumatoid factor (RF) negative or RF-positive polyarticular JIA.

At one year following initial presentation to paediatric rheumatology, children were classified as i) CID according to Wallace’s preliminary criteria and ii) CID according to cJADAS10. Baseline core outcome variables (active joint count, limited joint count, physician’s global, parental global, functional ability (Childhood Health Assessment Questionnaire (CHAQ)) and ESR) were assessed as potential predictors. Associations were assessed via univariable and forced-entry multivariable logistic regressions, adjusting for age and symptom duration at initial presentation, gender and ILAR subtype. Multiple imputation accounted for missing data.

**Results:** Of 829 children included, 70% were female and the majority had oligoarticular JIA (68%). At one year, 28% had achieved CID according to Wallace’s preliminary criteria and 38% according to the cJADAS10 (21% CID on both).

In univariable analyses, worsening baseline CHAQ and physician’s global assessment scores predicted lower odds of achieving both CID states. In addition, a greater number of active joints and worsening patient/parent global assessment scores predicted lower odds of CID on the cJADAS10 only. In multivariable analyses, an increase in 0.125 points on the CHAQ at baseline independently predicted 4% lower odds of CID on the cJADAS10 (95% CI 0.92, 0.99). However, no baseline core outcome variables predicted CID on Wallace’s preliminary criteria. No demographic variables were significantly predictive in any model.

**Conclusion:** There were different predictors for CID on the cJADAS10 vs. Wallace’s preliminary criteria. Children with poor functional ability at initial presentation are less likely to achieve CID on the cJADAS10. These children should be identified at an early stage and receive targeted therapies to better control their disease.

**Disclosure of Interest**

None Declared

### P238 Characteristics and safety outcomes of patients with juvenile idiopathic arthritis in two us healthcare claims databases

#### T. A. Simon^1^, S Singhal^2^, N Ray^2^, Z Guo^1^

##### ^1^Bristol-Myers Squibb, Princeton, NJ, USA; ^2^Mu Sigma, Bangalore, India

###### **Correspondence:** T. A. Simon

**Introduction:** Abatacept, the first selective co-stimulation modulator approved and used for the treatment of juvenile idiopathic arthritis (JIA), has a mechanism of action that is fundamentally different from that of other biologic (b)DMARDs.

**Objectives:** The purpose of this study in two US healthcare claims databases is to describe the baseline characteristics and safety outcomes of patients with a diagnosis of JIA who were treated with abatacept and those treated with other bDMARDs.

**Methods:** Patients <18 years of age and diagnosed with JIA in the QuintilesIMS^TM^ PharMetrics Plus database (1 Jan 2006–30 Sep 2015), or in the Truven Health MarketScan® database (1 Jan 2006–30 Sep 2014) were eligible for inclusion in the analysis. Patients were required to have at least 180 days of continuous health plan enrolment prior to a diagnosis of JIA based on two International Classification of Diseases, Ninth Revision, Clinical Modification codes (714.3x) within 90 days. The abatacept cohort included patients initiating IV abatacept or switching to IV abatacept from another bDMARD. The IV bDMARD cohort included patients initiating an IV biologic apart from abatacept for the first time. Baseline characteristics including at least 15 co-morbid conditions and concomitant medications were captured within the 6-month period prior to the diagnosis of JIA. Incidence rates (IRs) of hospitalized infections (HIs), uveitis and malignancies were calculated. Patients with an event in their 180-day baseline period were excluded. For malignancies, a latency period of 180 days was added, and patients had to have two diagnosis codes for the same malignancy and events were observed from 180 days post-index date to the earliest of event date, enrolment end date or study end date. For uveitis, the same methodology was used with one diagnosis code. For HI, patients had to have one diagnosis code for an infection during a hospitalization and events were observed from index date to the earliest of event date, treatment end date, enrolment end date or study end date.

**Results:** A total of 225 and 238 patients treated with IV abatacept, and 1346 and 1252 patients treated with other IV bDMARDs were identified in PharMetrics and MarketScan databases, respectively. Most patients were female and the abatacept cohort was older than the other bDMARD cohort. IRs of HIs and uveitis are listed in the table. No abatacept patient reported a malignancy and only one other bDMARD patient had a validated malignancy with two diagnosis codes in the MarketScan database. One abatacept patient and five other bDMARD patients had a validated malignancy in the PharMetrics database.

**Conclusion:** Abatacept patients in both data sources were older and had more co-morbid conditions at baseline with the exception of uveitis; the rate of HIs was similar between abatacept and other bDMARD patients. The incidence of uveitis was not significantly different between the groups and malignancies were rare. These data demonstrate an acceptable safety profile of abatacept and are consistent with data reported in registries and ongoing clinical trials in JIA patients.^1-4^

1. Lovell DJ, et al. ACR Congress 2016: poster 389.

2. Simon TA, et al. PReS Congress 2016: poster P344.

3. Lovell DJ, et al. *Arthritis Rheumatol* 2015;**67**:2759-70.

4. Lovell DJ, et al. ACR Congress 2016: oral 948.

**Disclosure of Interest**

T. A. Simon Shareholder of: BMS, Employee of: BMS, S. Singhal Consultant for: BMS, N. Ray Consultant for: BMS, Z. Guo Shareholder of: BMS, Employee of: BMS

**Table 16 (Abstract P238).** See text for description

	PharMetrics		MarketScan	
Baseline characteristics*	Abatacept-treated patients n = 225	Other bDMARD-treated patients n = 1346	Abatacept-treated patients n = 238	Other bDMARD-treated patients n = 1252
Female	183 (81.3)	948 (70.4)	195 (81.9)	927 (74.0)
Age, mean (SD)	13.17 (3.2)	10.86 (3.5)	12.36 (3.2)	11.03 (3.5)
Baseline co-morbidities				
Asthma	18 (8.0)	63 (4.7)	21 (8.8)	46 (3.7)
Cardiovascular disease	22 (9.8)	53 (3.9)	18 (7.6)	49 (3.9)
Hospitalized infections				
Uveitis	14 (6.2)	28 (2.1)	10 (4.2)	27 (2.2)
Biologic DMARDs	22 (9.8)	140 (10.4)	22 (9.2)	159 (12.7)
Non-biologic DMARDs	109 (48.4)	2 (0.1)	84 (35.3)	2 (0.2)
Inpatient visits, mean (SD)	139 (61.8)	614 (45.6)	105 (44.1)	476 (38.0)
Outpatient visits, mean (SD)	0.21 (0.8)	0.07 (0.4)	0.18 (0.8)	0.07 (0.3)
	12.01 (10.2)	7.89 (9.0)	11.63 (10.7)	7.43 (8.9)
Outcomes				
HIs, IR (95% CI) Uveitis, IR (95% CI)	1.4 (0.3, 4.1) 1.6 (0.5, 3.8)	1.7 (1.1, 2.5) 3.1 (2.4, 3.9)	2.4 (0.9, 5.3) 3.6 (1.7, 6.9)	1.6 (1.0, 2.4) 3.3 (2.5, 4.2)

Data are n (%) unless indicated otherwise

*During the 6-month period prior to treatment. HI = hospitalized infection; IR = incidence rate

### P239 Prediction of inactive disease in a multicentre inception cohort of JIA patients

#### Pieter Van Dijkhuizen^1^, Orfeas Aidonopoulos^2^, Nienke ter Haar^1^, Denise Pires Marafon^3^, Silvia Magni-Manzoni^3^, Yannis E. Ioannidis^4^, Lorenza Putignani^5^, Sebastiaan J. Vastert^1^, Clara Malattia^6,7^, Fabrizio De Benedetti^3^, Alberto Martini^6,7^

##### ^1^Paediatric Rheumatology, Wilhelmina Children’s hospital, University Medical Centre Utrecht, Utrecht, Netherlands; ^2^Institute for the Management of Information Systems, Athena Research and Innovation Center, Athens, Greece; ^3^Paediatric rheumatology, Ospedale Pediatrico Bambino Gesù, Rome, Italy; ^4^Department of Informatics and Telecommunications, University of Athens, Athens, Greece; ^5^Human microbiome unit, Ospedale Pediatrico Bambino Gesù, Rome, Italy; ^6^Paediatric rheumatology, Istituto Giannina Gaslini, Genoa, Italy; ^7^Università degli Studi Di Genova, Genoa, Italy

###### **Correspondence:** Pieter Van Dijkhuizen

**Introduction:** It is currently impossible to predict the prognosis of juvenile idiopathic arthritis (JIA) patients at diagnosis. Correct prediction of disease prognosis would enable physicians to stratify patients and make optimal use of existing effective medication.

**Objectives:** To predict inactive disease in a cohort of JIA patients, using clinical data, gut microbiota composition and a large panel of inflammation related compounds in plasma.

**Methods:** We analysed a cohort of 152 treatment-naïve JIA patients enrolled within 6 months after the onset of disease. No systemic JIA patients were enrolled. We collected clinical parameters regarding disease activity, a faecal sample to determine the gut microbiota composition and blood to analyse a large panel of cytokines and chemokines using Luminex technology. Patients were followed up every 6 months and additionally in case of flare, for 2 years. Disease activity was determined using the Wallace criteria.

The prediction model was developed using clinical data only, clinical and microbiota data, or clinical and Luminex data. Variables were selected in univariate analysis. All models contained treatment and time elapsed since baseline as potential confounders. The models were developed in two thirds of patients and validated in the remaining one third of patients. The split in training and test data was performed 10 times and model performance was averaged over the different splits.

Various models were tried. A repeated measurements analysis was performed using generalized estimating equations (GEE). Cox proportional hazards models of time to first inactive disease and time to inactive disease using recurrent events were fitted as well. We also performed logistic regression of inactive disease at 6 months and 12 months. Additionally, to reduce the heterogeneity underlying JIA, all models were refitted in the subgroups of oligoarticular patients and polyarticular rheumatoid factor (RF) negative patients.

**Results:** The majority of patients was female (121, 80%) and presented with at most 4 active joints at baseline (109, 72%). The median juvenile arthritis disease activity score (JADAS)-71 was 13 (1^st^ quartile, 3^rd^ quartile: 8, 19).

The clinical GEE model showed moderate performance in training data (area under the curve [AUC] 0.70), but poor performance in test data (AUC 0.65). Similar or poorer performance was observed for the microbiota and Luminex GEE models, as well as for the Cox regression. The logistic regression of the 6 months and 12 months outcome did slightly better with an AUC of 0.74 in training data and 0.66 in test data for both the clinical and the microbiota model.

The highest AUCs were obtained when analysing oligoarticular patients and polyarticular RF negative patients separately, using GEE. The AUC of the oligoarticular microbiota model was 0.79 in training data and 0.69 in test data. Higher JADAS-71 score and a higher relative abundance of the operational taxonomic unit *Mogibacteriaceae* were associated with lower odds of inactive disease*.* The best model in polyarticular patients was the Luminex model with an AUC of 0.79 in training data and 0.69 in test data. Longer duration of morning stiffness and a higher plasma concentration of CXCL-9 were associated with decreased odds of inactive disease, whereas a higher haemoglobin level was associated with increased odds of inactive disease at follow up.

**Conclusion:** Prediction of the prognosis in JIA is challenging. In this well-defined inception cohort of JIA patients, we were unable to predict disease inactivity with satisfactory accuracy, using clinical data, gut microbiota composition and a panel of cytokines and chemokines. This is potentially due to the heterogeneity underlying JIA. Indeed, predictive accuracy of the models increased when oligoarticular and polyarticular RF negative patients were considered separately.

**Disclosure of Interest**

None Declared

## JIA treatment

### P240 Methotrexate as first line therapy in juvenile idiopathic arthritis-associated uveitis: myth or reality?

#### Jacopo Agnolucci, Maria E. Zannin, Fabio Vittadello, Giorgia Martini, Alessandra Meneghel, Francesco Zulian

##### Department of Woman and Child Health, University of Padova, Padova, Italy

###### **Correspondence:** Jacopo Agnolucci

**Introduction:** Methotrexate (MTX) is the most used immunomodulatory drug in Juvenile Idiopathic Arthritis-associated uveitis (JIA-U) although its efficacy has been shown only in retrospective studies with limited number of subjects, short follow up and sometimes questionable methodology (survey). Time between onset of uveitis and MTX start as well as time to switch between first and second-line therapies are still controversial.

**Objectives:** To evaluate the efficacy of MTX as first-line therapy in a monocentric inception cohort of patients with JIA-U.

**Methods:** Patients with JIA-U refractory to local eyedrops treatment were managed by a standard protocol including MTX as first line systemic treatment. Data, recorded every 3 months, included ocular flares and complications, drug-related adverse events (AE) and treatment change. The diagnosis of JIA was based upon the ILAR criteria and the diagnosis of uveitis was made according to the SUN Criteria. Uveitis flare was defined as an increase of cells in the anterior chamber of 2+ or more as compared to the baseline. Clinical remission was defined as the absence of flares for more than 6 months on treatment, without or with minimal topical treatment (corticosteroid and/or mydriatic-cycloplegic eye drops ≤ 1/day). Data of patients treated for at least 1 year were analyzed using descriptive statistics.

**Results:** 84 JIA patients (71 F, 13 M), 82 oligoarticular, 2 polyarticular, treated with MTX for JIA-U entered the study; mean age at MTX start: 5.7 years (1.8–21.6); mean f/u since MTX start 8.9 y (1–20 y). 68 patients began MTX primarily to treat uveitis (group A) (JIA duration 1.8 y), 16 patients introduced MTX for arthritis (group B) (JIA duration 0.54 y). The mean interval time between arthritis and uveitis was 0.8 y in group A and 2.1 y in group B (p < 0.01). After treatment start, 25% relapsed with a uveitis flare by 5 months, 50% by 9.7 mo and 75% by 36 mo. In general, 40 pts (47.6%) 28 in group A and 12 in group B needed anti-TNF therapy; 35 (41.7%) were in remission on medication and only 9 (10.7%) (all in group A) reached a complete remission at a mean f/u of 39.4 months.

**Conclusion:** MTX looses efficacy in the medium-long term and ensures clinical remission only in a minority of patients. When started before uveitis, MTX delays the uveitis onset but does not prevent its severe course.

**Disclosure of Interest**

None Declared.

### P241 Methotrexate use and route of administration in juvenile idiopathic arthritis: results from the childhood arthritis and rheumatology research alliance (CARRA) registry

**Abstract withdrawn**

### P242 Identification of optimal subcutaneous doses of tocilizumab in children with polyarticular-course juvenile idiopathic arthritis

**Abstract withdrawn**

### P243 The national centre for paediatric rheumatology (NCPR) experience of the use of tocilizumab (RO-ACTEMRA) in the treatment of juvenile idiopathic arthritis (JIA): a 7-year story

#### Charlene Foley, Wafa Madan, Emma-Jane MacDermott, Orla G Killeen

##### Our Lady’s Children’s Hospital, Crumlin, Dublin, Ireland

###### **Correspondence:** Charlene Foley

**Introduction:** Tocilizumab is a recombinant-humanised-monoclonal-antibody that acts as an Interleukin-6-receptor-antagonist. Approved for the treatment of children over 2 years with Systemic-onset JIA (SoJIA) and Polyarticular JIA (pJIA), Tocilizumab has been used in the NCPR for these indications since 2010.

**Objectives: Aim -** To perform a retrospective review of all children with JIA attending the NCPR treated with Tocilizumab, and report on outcomes, tolerability and efficacy.

**Methods:** A retrospective review of all JIA patients receiving Tocilizumab was performed and baseline demographics recorded. Active disease was defined by active joint count (AJC), and/or presence of raised Acute Phase Reactants (APR). Pre-Tocilizumab biologic workup, infusion frequency and prior and adjunctive treatments were reviewed. Outcome measures included clinical remission (0 AJC) and time to remission.

**Results:** Thirty-two children with JIA (81% Female, median age at diagnosis 5.1yr, 1.8-12.8yrs) have received Tocilizumab, 41% SoJIA, 34% pJIA (3/11 RF-positive), 25% Other.

Median time to commencing Tocilizumab was 3.1yrs (0.9-10.4yrs). Prior to Tocilizumab, 97% of children received Methotrexate monotherapy. Following this, 91% received at least two Biologics, 6% received four.

Pre-Tocilizumab 97% were Varicella immune, all TB negative. The average AJC was 17 (3-23joints) and APR were raised in 53%.

All received Tocilizumab fortnightly at the outset. Escalation to weekly infusions was required in 28% (56% SoJIA). Adjuvant steroids were required in 56% at commencement of Tocilizumab. Complete steroid wean was achieved in 83%.

Seventy-four percent of the cohort has achieved remission on Tocilizumab. Average time to remission, 5mths (0.5-15mths). APR normalised in 76% after one infusion, 100% after three. Tocilizumab was discontinued in 22% (7/32). Of the remaining twenty-five, 32% achieved reduced infusion frequency (3-8weekly).

**Conclusion:** NCPR experience with Tocilizumab has been positive, with high rates of remission (74%) and tolerability, 78% remaining on the drug. Rather than using multiple biologics, this encourages consideration of the drug earlier.

**Disclosure of Interest**

None Declared.

### P244 Modulating treg function with nicotinamide (VIT B3) in JIA

#### Lotte Nijhuis^1^, Janneke Peeters^1^, Arief Lalmohamed^2^, Jorg Van Loosdregt^1^, Bas Vastert^3^

##### ^1^Laboratory of Translational Immunology, UMC Utrecht, Utrecht, Netherlands; ^2^Department of Clinical Pharmacy, UMC Utrecht, Utrecht, Netherlands; ^3^Department of Pediatric Immunology, UMC Utrecht, Utrecht, Netherlands

###### **Correspondence:** Lotte Nijhuis

**Introduction:** Juvenile Idiopathic Arthritis (JIA) is hallmarked by a disturbed immunological balance between regulatory T cells (Treg) and effector T cells (Teff). Restoring this balance by enhancing the suppressive function or number of Treg might therefore be a promising novel therapeutic strategy. The transcription factor FOXP3, is essential for Treg function and development. We have previously demonstrated that the acetylation of FOXP3 prevents its degradation, thereby resulting in increased FOXP3 protein levels. The lysine deacetylase SIRT1 can deacetylate FOXP3 and therefore promote its degradation. Therefore, inhibitors of SIRT1 could lead to increased FOXP3 expression.

Interestingly, a rather specific SIRT1 inhibitor, and well known food additive, is Vitamin B3, also known as nicotinamide (NAM). Therefore, NAM has the potential to stabilize FOXP3 expression and positively influence regulatory T cell function. Translating these laboratory findings into clinical practice could result in a novel therapeutic strategy. We envision that NAM, when combined with established immunosuppressive treatment, could help regain immunological balance in JIA. NAM is well known as a dietary supplement and has been extensively studied in a variety of diseases in both children and adults. However, the safety, tolerability and optimal dosing of NAM in children with JIA is still unknown. Therefore, before such a therapeutic strategy can be tested in a placebo-controlled trial, we need to gain detailed information on dosing, tolerability, and surrogate parameters of effectiveness in JIA.

**Objectives:** We aim to gain more information regarding the mechanistic effect of NAM on the function, FOXP3 expression, and cytokine expression of Treg and Teff in both blood and synovial fluid of JIA patients and healthy controls. In addition, in preparation of a phase III clinical trial, we want to acquire essential data on pharmacokinetics/dynamics, safety, feasibility and tolerability of NAM in the proposed dose in children with JIA.

**Methods:** Human peripheral blood T cells of healthy controls (N = 3) and synovial fluid of oligo-JIA patients (N = 5)were isolated and cultured in the presence of NAM in different concentrations for 4-7 days. The percentage of FOXP3+ cells and proliferation index of CD4+ and CD8+ T cells was determined using Flow cytometry. To assess Treg function, isolated human T cells were cultured with NAM after which Treg were sorted and their function was analyzed using a suppression assay.

**Results:** *In vitro* NAM treatment of human T cells significantly increased both the percentage of FOXP3+ cells and the FOXP3 mean fluorescent intensity. Furthermore, the suppressive function of Treg treated with NAM was increased. Importantly, this was confirmed in cells isolated from the synovial fluid of JIA patients. In addition to the effect on regulatory T cells, NAM reduced the proliferation of CD4+ and CD8+ T cells.

**Conclusion:** Collectively, these data demonstrate that NAM treatment *in vitro* results in more Treg with an increased suppressive capacity. In addition, NAM directly suppressed the proliferation of Teff cells. Therefore, NAM treatment could rebalance the active adaptive immune system in patients with JIA by suppressing immune activation and increasing tolerance. Hence, NAM treatment as an adjuvant therapy has the potential to benefit patients with JIA and potentially other autoimmune diseases. To directly test this hypothesis we are now conducting a phase I/II trial as a run-up for a phase III trial in which we will test whether NAM treatment is useful in a strategy of maintaining disease remission after stopping DMARD or biologicals.

**Disclosure of Interest**

None Declared.

### P245 The analysis of a neutropenia cases in juvenile idiopathic arthritis under tocilizumab therapy

#### Maria Kaleda, Irina Nikishina, Olga Kostareva

##### Pediatrics, V.A. Nasonova Research Institute of Rheumatology, Moscow, Russian Federation

###### **Correspondence:** Irina Nikishina

**Introduction:** Neutropenia is a well-known laboratory abnormality in tocilizumab (TCZ) therapy of systemic and polyarticular juvenile idiopathic arthritis (sJIA/pJIA). There may be some differences in the timing and causes of neutropenia between pJIA and sJIA.

**Objectives:** The purpose of this work was to perform detail analysis of all cases of TCZ therapy JIA with focuses on neutropenia phenomenon.

**Methods:** In prospective study were included 27 pts (8b/19g) with pJIA (8 with uveits, 3 RF+) and 83 pts (35b/48g) with sJIA, refractory to conventional treatment, who was treated by TCZ from 2 months (mo) to 84 mo (median 22 mo for pJIA, 34 mo for sJIA). At baseline mean age of 11.1 (9.0-14.7)yrs for pJIA, 6.25 (4.8-11.25)yrs for sJIA; mean disease duration of 5.4 (2.0-8.9)yrs for pJIA, 2.5 (0.8-5.7)yrs for sJIA. Worst Common Toxicity Criteria (CTC) neutropenia grade (grade 1, ≥1.5 and <2.0 × 109/L; grade 2, ≥1.0 and <1.5 × 109/L; grade 3, ≥0.5 and <1.0 × 109/L; grade 4, <0.5 × 109/L) and lowest observed neutrophil count (109/L) were identified for each patient. TCZ used as the 1st biologic agent (BA) in 15/54 pts with pJIA/sJIA, 2nd – in 4/12, 3rd – in 10/16. Previous therapy included NSAIDs - 100%/65% pts with pJIA/sJIA, glucocorticoids (GC) per os – 20%/92.5%, methotrexate (MTX) - 93%/89%.

**Results:** 22/63 pts continue the treatment, mean duration 27.4 months (9;72). In 4/15 pts with pJIA/sJIA TCZ was cancelled due to serious adverse effects (4/12), another reasons (2/7). 60.7%/41.5% pts with pJIA/sJIA had at least 1 episode of grade 1-4 neutropenia; worst grade: 1 (n = 7/12), 2 (n = 6/14), 3 (n = 4/9), and 4 (n = 2/0). 73% of pts had the maximum decrease in the level of neutrophils at 1st day after infusion. Pts with pJIA are more likely to have neutropenia in the first months of therapy, pts with sJIA in achieving remission. 5 pts with sJIA had neutropenia upon MAS that was not related to TCZ. Rates of infections during period of normal neutrophil counts were comparable to those observed ±15 days around grade 1-4 neutropenia, with no trend toward increased risk with higher grade neutropenia. MTX use wasn’t significantly associated with lowest observed neutrophil count (p > 0.5). Older age was associated with lowest level of neutrophils (p < 0.05). Combined use of GC and TCZ exposure were associated with normal level of neutrophils (p > 0.5). Previous BA didn’t increase the risk of neutropenia (p > 0.5). All cases of neutropenia were recorded in pts with response to therapy more than 50% ACRpedi criteria.

**Conclusion:** Our data show, that there are different kinds of neutropenia phenomenon: 1. “benign”, transient neutropenia - a predictor of high efficiency of TCZ, developes primarily in the first few days after infusion; 2. neutropenia as a phenomenon of “redundancy” of therapy (more resistent) and requires correction of the dose of TCZ and concomitant therapy; 3. neutropenia, as a risk of manifestation of MAS, mainly due to an inadequate effect or its “exhaustion”, is associated with other markers of MAS. We didn’t observe association between neutropenia and increased risk of infections. The neutropenia should be constantly monitored with subsequent individual correction of therapy.

**Disclosure of Interest**

None Declared.

### P246 Subcutaneous abatacept in patients aged 6–17 years with polyarticular-course juvenile idiopathic arthritis and inadequate response to biologic or non-biologic disease-modifying antirheumatic drugs: 2-year efficacy and safety results

#### N. Ruperto^1^, H. I. Brunner^2^, N. Tzaribachev^1^, I. Louw^1^, A. Berman^1^, I. Calvo^1^, R. Cuttica^1^, G Horneff^1^, M Henrickson^2^, D J Kingsbury^2^, I. Foeldvari^1^, M. E. Paz Gastanaga^1^, B Lauwerys^1^, K Minden^1^, J Bohnsack^2^, J. C. Breedt^1^, R. van Zyl^1^, X. Li^3^, M Nys^4^, R. Wong^3^, S. Banerjee^3^, A. Martini^1^, D. J. Lovell^2^ and For Paediatric Rheumatology International Trials Organization (PRINTO)/Pediatric Rheumatology Collaborative Study Group (PRCSG)

##### ^1^PRINTO, Istituto Gaslini, Genoa, Italy; ^2^PRCSG, CHMC, Cincinnati, OH, USA; ^3^Bristol-Myers Squibb, Princeton, NJ, USA; ^4^Bristol-Myers Squibb, Braine L’Alleud, Belgium

###### **Correspondence:** N Ruperto

**Introduction:** IV abatacept 10 mg/kg every 4 weeks has proved to be well tolerated and effective in reducing the signs and symptoms of polyarticular juvenile idiopathic arthritis (pJIA).^1^ In adult RA, weight-tiered IV (~10 mg/kg) and SC abatacept 125 mg weekly have equivalent efficacy and comparable safety;^2^ therefore, SC abatacept may be similarly beneficial in patients with pJIA.

**Objectives:** To report 2-year efficacy, safety and pharmacokinetic results of SC abatacept treatment in patients aged 6–17 years with active pJIA.

**Methods:** Patients with pJIA and inadequate response/intolerance to ≥1 DMARD were enrolled in two age cohorts (2–5 years and 6–17 years) into this 2-year, single-arm, open-label (OL), Phase III study. Patients received SC abatacept weekly for 4 months based on body weight tier (10– < 25 kg [50 mg abatacept]; 25– < 50 kg [87.5 mg abatacept]; ≥50 kg [125 mg abatacept]). JIA-ACR criteria 30 (JIA-ACR30; ACR Pediatric 30) responders at 4 months could enter a 20-month OL extension. The primary endpoint was abatacept steady-state serum trough concentration (C_minss_) at 4 months in the 6–17-year cohort. Secondary efficacy outcomes included JIA-ACR30, 50, 70, 90 and inactive disease (no active joints, Physician Global Assessment of disease activity <10 mm, CRP <0.6 mg/dL) response rates. Safety was evaluated throughout the study.

**Results:** For the 6–17-year cohort (intent-to-treat, n = 173), baseline median (25^th^ percentile, 75^th^ percentile) age and number of active joints were 13.0 (10.0, 15.0) years and 10.0 (6.0, 19.0), respectively. Number of patients (%) with concomitant use of MTX was 136 (78.6) and with prior biologic use was 46 (26.6). Overall, 132 (76.3%) patients completed the 20-month OL extension period. Robust JIA-ACR30, 50, 70, 90 and inactive disease responses were seen at 2 years (n = 109): 92.7, 89.0, 83.5, 65.1 and 57.9%, respectively. No new or unexpected safety concerns were reported (Table 17). Mean (SD) C_minss_ value was stable over the 2-year period, ranging from 40.6 to 47.7 μg/mL, achieving the target therapeutic exposure (C_minss_ = 10 μg/mL) consistently across all three weight groups.

**Conclusion:** In this 2-year analysis of patients with pJIA aged 6–17 years, SC abatacept was well tolerated and demonstrated robust efficacy with no new safety concerns; target therapeutic exposure with SC abatacept was achieved and within the range of abatacept levels observed with IV abatacept in pJIA.

1. Ruperto N, et al. *Lancet* 2008;**372**:383–91.

2. Genovese MC, et al. *Arthritis Rheum* 2011;**63**:2854–64.

**Trial registration identifying number:** ClinicalTrials.gov NCT01844518

**Disclosure of Interest.**

N. Ruperto Grant/Research Support from: The G. Gaslini Hospital, which is the public Hospital where I work as full time public, Consultant for: AbbVie, Amgen, Biogen Idec, Alter, AstraZeneca, Baxalta Biosimilars, Boehringer Ingelheim, BMS, Celgene, Crescendo Bioscience, EMD Serono, Hoffman-La Roche, Speaker Bureau of: AbbVie, Amgen, Biogen Idec, Alter, AstraZeneca, Baxalta Biosimilars, Boehringer Ingelheim, BMS, Celgene, Crescendo Bioscience, EMD Serono, Hoffman-La Roche, H. I. Brunner: None Declared, N. Tzaribachev: None Declared, I. Louw: None Declared, A. Berman: None Declared, I. Calvo Grant/Research Support from: Novartis, Speaker Bureau of: AbbVie, Roche, Novartis, Sobi, R. Cuttica: None Declared, G. Horneff Speaker Bureau of: AbbVie,Pfizer, Chugai, Roche, Novartis, M. Henrickson: None Declared, D. J. Kingsbury: None Declared, I. Foeldvari: None Declared, M. E. Paz Gastanaga: None Declared, B. Lauwerys: None Declared, K. Minden: None Declared, J. Bohnsack: None Declared, J. C. Breedt: None Declared, R. van Zyl: None Declared, X. Li Employee of: BMS, M. Nys Shareholder of: BMS, Employee of: BMS, R. Wong Shareholder of: BMS, Employee of: BMS, S. Banerjee Shareholder of: BMS, Employee of: BMS, A. Martini: None Declared, D. J. Lovell Grant/Research Support from: National Institutes of Health, NIAMS, Consultant for: AstraZeneca, Bristol-Myers Squibb, AbbVie, Pfizer, Roche, Novartis, UBC, Forest Research Institute, Horizon, Johnson & Johnson, Biogen, Takeda, Genentech, GlaxoSmithKline, Boehringer Ingelheim, Celgene, Janssen, Speaker Bureau of: Genentech.

**Table 17 (Abstract P246).** Summary of Patients with AEs During the Combined Initial 4-Month and 20-Month Extension Period in the 6–17-Year Cohort (n = 173)

Event	n = 173
Deaths	0
SAEs	14 (8.1)*
Related SAEs	1 (0.6)^†^
SAEs leading to discontinuation	4 (2.3)^‡^
All AEs^§^	152 (87.9)
Related AEs	54 (31.2)
AEs leading to discontinuation	7 (4.0)^¶^
AEs of special interest	
Malignancies	1 (0.6)**
Autoimmune disorders	3 (1.7)
Local injection-site reactions	12 (6.9)
Infections	42 (24.3)

Data are n (%)

*Sepsis, abdominal pain and upper respiratory tract infection (occurred in one patient), appendicitis, pneumonia, pyelonephritis, concussion, radius fracture, urinary calculus, nephrolithiasis, anaemia, vertigo, chest pain, synovitis, hypomagnesaemia and ovarian germ cell teratoma stage III (occurred in one patient), autonomic nervous system imbalance

^†^Sepsis

^‡^Sepsis, vertigo, ovarian germ cell teratoma stage III, autonomic nervous system imbalance

^§^No opportunistic infections, including extra-pulmonary tuberculosis occurred during the study. One case of latent tuberculosis was reported (mild, not related to abatacept treatment, drug was interrupted, treatment was required)

^¶^Ovarian germ cell teratoma stage III, fatigue, sepsis, exanthema, autonomic nervous system imbalance, aphthous ulcer, vertigo

**Ovarian germ cell teratoma stage III

SAE = serious AE

### P247 Comparative assessment of growth in children with juvenile idiopathic arthritis treated with biological therapy with a different mechanism of action (tocilizumab, etanercept, adalimumab, abatacept)

#### Valentina Seraya, Elena Zholobova, Alisa Vitebskaya

##### I.M.Sechenov First Moscow State Medical University, Moscow, Russian Federation

###### **Correspondence:** Valentina Seraya

**Introduction:** In children with severe juvenile idiopathic arthritis (JIA) often occur impaired growth and growth retardation (up to 10-20%). It is of importance to investigate the influence of biological therapy (BT) on growth in children with JIA.

**Objectives:** to assess the effect of BT with different mechanisms of action on growth and growth rate in children with JIA.

**Methods:** The study included 125 children with JIA and high activity (48 boys, 77 girls, 50 had systemic JIA (sJIA), 55 - polyarticular, 20 - oligoarticular, mean age 12,4 yrs, mean duration of the disease before therapy with BT 5,1yrs). The patients were divided into groups according to the BT produced: 25 children with sJIA (group I) received different BT (etanercept, adalimumab, abatacept), 25 with sJIA (group II) - tocilizumab, 75 children with poly- and oligoarticular JIA respectively received: 25 patients (group III) - etanercept, 25 children (group IV) - adalimumab and 25 children (group V) - abatacept. The study evaluated growth SDS, growth rate and growth rate SDS at baseline (prior BT) and after 6, 12 and 24 months of therapy.

**Results:** Growth retardation at the baseline (SDS < -2) was detected in 29 patients (23%). By 24 months of BT, the number of children with short stature decreased to 19 (15%).

Dynamics of growth rate and growth rate SDS are presented in the Table 18.

Growth rate prior BT in all groups are comparable (p > 0.05). The lowest growth rate was observed in children with sJIA. In all patient receiving BT, the growth rate increased and remained higher than growth rate at the baseline. Growth rate in children receiving tocilizumab (group II) by 12 months of treatment and in children receiving adalimumab (group III) by 6 months of therapy is significantly higher comparing to other groups (p <0.05).

In group 1, the mean growth SDS at the baseline was -1.96 ± 0.52. By 24 months of BT growth SDS increased to -1.61 ± 0.39 which was comparable to baseline (p = 0.058).

In group 2, the mean growth SDS at the baseline was -0.51 ± 0.37. By 24 months of BT growth SDS significantly increased to -0.16 ± 0.30 (p = 0.043).

In group 3, the mean growth SDS at the baseline was -0.35 ± 0.34. By 24 months of BT growth SDS amounted -0.22 ± 0.34 which was comparable to baseline (p = 0.108).

In group 4, the mean growth SDS at the baseline was -0.63 ± 0.29. By 24 months of BT growth SDS significantly increased to -0.31 ± 0.31 (p = 0.020).

In group 5, the mean growth SDS at the baseline was -0.06 ± 0.22.

By 24 months of BT growth SDS amounted 0.08 ± 0.21, which was comparable to baseline (p = 0.088).

**Conclusion:** Treatment with biological therapy increases growth rate not only in the first 6 month, but in the subsequent period of treatment. The highest growth rate is observed in children receiving tocilizumab and adalimumab. The higher growth rate results in increased growth SDS by 24 months of treatment. BT in children with severe JIA and impaired growth leads to a decrease of incidence and severity of short stature.

**Disclosure of Interest**

None Declared.

**Table 18 (Abstract P247).** Dynamics of growth rate and growth rate SDS, N = 125

mean growth rate	baseline, sm/yr (SDS)	after 6 months sm/yr, (SDS)	after 12months, sm/yr, (SDS)	after 24months sm/yr, (SDS)	p (baseline/after 24 mounth)
groups, N = 25					
group I	**3,16 ± 0,52** (-2,02 ± 0,70)	**3,98 ± 0,38** (-0,42 ± 0,54)	**6,30 ± 0,69** (2,97 ± 1,04)	**5,27** ± **0,65** (2,36 ± 0,92)	p = 0,011 p = 0,002
group II	**4,30 ± 0,30** (-2,30 ± 0,20)	**5,07 ± 0,36** (-0,29 ± 0,79)	**8,26 ± 0,98** (3,18 ± 1,18)	**6,67 ± 0,80** (2,40 ± 0,60)	p = 0,024 p = 0,009
group III	**3,28 ± 1,36** (-1,70 ± 1,38)	**6,50 ± 0,62** (0,63 ± 0,57)	**6,70** ± **0,71** (1,09 ± 0,79)	**5,38** ± **0,56** (0,11 ± 0,40)	p = 0,036 p = 0,015
group IV	**4,90 ± 0,77** (0,02 ± 0,62)	**7,40** ± **0,79** (1,80 ± 0,88)	**6,90** ± 0**,78** (2,17 ± 1,06)	**6,44** ± **0,58** (1,40 ± 0,70)	p = 0,058 p = 0,051
group V	**5,21 ± 1,01** (-0,25 ± 1,41)	**7,21** ± **0,76** (2,21 ± 0,96)	**5,64** ± **0,43** (0,35 ± 0,70)	**5,14 ±** 0,57 (0,26 ± 0,53)	p = 0,083 p = 0,088

### P248 Families’ views on the feasibility of a corticosteroid trial in JIA: a qualitative study

#### Frances C. Sherratt^1^, Louise Roper^1^, Eileen Baildam^2^, Michael W. Beresford^2^, Simon R. Stones^3^, Athimalaipet Ramanan^4^, Helen Foster^5^, Flora McErlane^5^, Madeline Rooney^6^, Matthew Peak^2^, Bridget Young^1^

##### ^1^Department of Psychological Sciences, University of Liverpool, Liverpool, UK; ^2^Alder Hey Children’s NHS Foundation Trust, Liverpool, UK; ^3^School of Healthcare, University of Leeds, Leeds, UK; ^4^Bristol Royal Hospital for Children, Bristol, UK; ^5^Great North Children’s Hospital, Newcastle upon Tyne, UK; ^6^School of Medicine, Dentistry and Biomedical Sciences, Queens University of Belfast, Belfast, UK

###### **Correspondence:** Frances C. Sherratt

**Introduction:** Current delivery routes and doses of corticosteroid (CS) treatment in juvenile idiopathic arthritis (JIA) are principally based on clinician and patient preference, rather than scientific evidence. A clinical trial is needed in the future to ascertain the most effective routes and doses of CS in JIA.

**Objectives:** To explore families’ experiences of four CS delivery routes in JIA and to assess their views regarding feasibility of a future trial evaluating the most effective routes and doses of CS.

**Methods:** Semi-structured interviews with a purposive sample of young people and their parents (N = 28; 9 young people and 19 parents), recruited from four UK Paediatric Rheumatology providers. Data were analysed using thematic analysis informed by the Framework Approach.

**Results:** Families had first-hand experience of oral prednisolone (n = 10), intravenous (IV) methylprednisolone (n = 7), intra-articular (IA) injections (n = 14) and intra-muscular (IM) injections (n = 3). The majority of families (n = 13/15) had experience of at least two delivery routes. Parents and young people tended to view oral prednisolone as less suitable for more advanced JIA and IA was viewed as less appropriate for patients with poly or systemic arthritis. Young people were more likely than their parents to report willingness to participate in a trial of CS. Overall, participants tended to be more willing to participate at time of diagnosis than at time of JIA flare; previous positive and negative experiences of delivery routes influenced perceptions of future treatment efficacy. Key barriers to participation identified by both parents and young people included: the perception that some delivery routes would be more or less appropriate due to factors such as age and JIA sub-type; being randomly allocated to receive a treatment, rather than the treatment being clinician-informed; concerns regarding the quality of care they would receive as a trial participant.

**Conclusion:** Recruitment of children and young people with flaring JIA to a future trial will likely be more challenging than recruitment at diagnosis. The findings can be used to inform the design of a future trial of CS. In order to optimise the recruitment process for a future trial, clinicians will need to provide families with comprehensive information on treatment arms, the rationale for randomisation (including emphasising equipoise), and reassurance of quality of care whether they participate or not. Doing so would reduce families’ anxieties, improve informed consent and potentially optimise recruitment. Researching families’ perspectives about future paediatric rheumatology trials can help to assess the feasibility of these trials and optimise their design.

**Disclosure of Interest**

None Declared.

### P249 Patterns and switching of first-line biologics in patients with juvenile idiopathic arthritis in two us healthcare claims databases

#### T. A. Simon^1^, S Singhal^2^, N Ray^2^, Z Guo^1^

##### ^1^Bristol-Myers Squibb, Princeton, NJ, USA; ^2^Mu Sigma, Bangalore, India

###### **Correspondence:** T. A. Simon

**Introduction:** Abatacept, the first selective co-stimulation modulator approved and used for the treatment of juvenile idiopathic arthritis (JIA), has a mechanism of action that is fundamentally different from that of other biologic (b)DMARDs.

**Objectives:** This study describes the patterns of biologic use in two US healthcare claims databases among patients with a diagnosis of JIA who were treated with either abatacept or other bDMARDs.

**Methods:** Patients <18 years of age and diagnosed with JIA in the QuintilesIMS™ PharMetrics Plus database between 1 Jan 2006 and 30 Sep 2015, or the Truven Health MarketScan® database between 1 Jan 2006 and 30 Sep 2014, were eligible for inclusion in the analysis. Patients were required to have at least 180 days of continuous health plan enrolment prior to a diagnosis of JIA, based on two International Classification of Diseases, Ninth Revision, Clinical Modification codes (714.3x) within 90 days. The first biologic was defined as the first biologic treatment claim for the patient. Switchers were defined as patients with a claim for a different bDMARD than the index bDMARD within a grace period of 90 days after the end of days-supply of the previous bDMARD claim. Patients who discontinued had no claim for another bDMARD within a grace period of 90 days after the end of days-supply of the previous bDMARD claim.

**Results:** The study included 91 patients treated with abatacept from the PharMetrics and 137 from the MarketScan database. In both databases, patients treated with abatacept were older, more likely to be female and less likely to have baseline uveitis than those treated with other bDMARDs (Table 19). Differences between the two treatment cohorts for most characteristics were consistent between the two databases. However, patients treated with abatacept were more likely to have baseline asthma and less likely to be on non-bDMARDs than those with other bDMARDs in the MarketScan database. In both databases, the rate of those who switched to a non-bDMARD and the discontinuation rate were all slightly lower and the number of patients who stayed on index drug was higher among abatacept-treated patients compared with patients on other bDMARDs (Table 19).

**Conclusion:** First-line abatacept users with JIA were different from other bDMARD users in age, sex and a few baseline co-morbidities. Additionally, patients with JIA taking abatacept are more likely to stay on abatacept treatment compared with bDMARDs.

**Disclosure of Interest**

T. A. Simon Shareholder of: BMS, Employee of: BMS, S. Singhal Consultant for: BMS, N. Ray Consultant for: BMS, Z. Guo Shareholder of: BMS, Employee of: BMS.

**Table 19 (Abstract P249).** See text for description

	PharMetrics		MarketScan	
Baseline characteristics	Abatacept n = 91	Other bDMARDs n = 3934	Abatacept n = 137	Other bDMARDs n = 3559
Female	79 (86.8)*	2820 (71.7)*	119 (86.9)*	2607 (73.3)*
Age, mean (SD)	13.4 (3.0)*	11.2 (4.2)*	12.6 (3.2)*	11.4 (4.2)*
Hospitalized infections	3 (3.3)	128 (3.3)	5 (3.7)	81 (2.3)
Uveitis	3 (3.3)*	434 (11.0)*	10 (7.3)	420 (11.8)
Non-bDMARDs	43 (47.3)	2063 (52.4)	37 (27.0)*	1801 (50.6)*
Treatment pattern Switching to non-bDMARD Discontinuing Staying on abatacept or index bDMARD Switching to other bDMARD	34 (37.4) 6 (6.6) 35 (38.5)* 16 (17.6)	1727 (43.9) 362 (9.2) 1114 (28.3)* 731 (18.6)	41 (29.9)* 14 (10.2) 62 (45.3)* 20 (14.6)	1680 (47.2)* 364 (10.2) 948 (26.6)* 567 (15.9)

Data are n (%) unless indicated otherwise

*p < 0.05 for the difference between the two treatment groups in each of the two databases

### P250 An internet-based self-management program for adolescents with juvenile idiopathic arthritis (JIA): a randomized controlled trial (RCT)

#### Lynn R. Spiegel^1^, Jennifer Stinson^2^, Sarah Campillo^3^, Tania Cellucci^4^, Paul Dancey^5^, Ciaran Duffy^6^, Janet Ellsworth^7^, Brian Feldman^1^, Adam Huber^8^, Nicole Johnson^9^, Patrick McGrath^10^, Alan Rosenberg^11^, Nathalie Shiff^12^, Shirley Tse^1^, Lori Tucker^13^, Charles Victor^14^, Stephanie Luca^15^

##### ^1^Pediatrics, Hospital for Sick Children, Toronto, Canada; ^2^Anaesthesia and Pain Medicine, Hospital for Sick Children, Toronto, Canada; ^3^Rheumatology, Montreal Children’s Hospital, Montreal, Canada; ^4^Pediatrics, McMaster Children’s Hospital, Hamilton, Canada; ^5^Pediatrics, Memorial University of Newfoundland, St John’s, Canada; ^6^Pediatrics, Children’s Hospital of Eastern Ontario, Ottawa, Canada; ^7^Pediatrics, Stollery Children’s Hospital, Edmonton, Canada; ^8^Pediatrics, IWK Health Center, Halifax, Canada; ^9^Pediatrics, Alberta Children’s Hospital, Calgary, Canda; ^10^Psychology, IWK Health Sciences Center, Halifax,Canada; ^11^Pediatrics, Royal University Hospital, Saskatoon, Canada; ^12^Pediatrics, UF Health-Shands Hospital, Gainesville, FL, USA; ^13^Pediatrics, BC Children’s Hospital, Vancouver, Canada; ^14^Institute for Clinical Evaluative Sciences, Toronto, Canada; ^15^Child Health Evaluative Sciences, The Hospital for Sick Children, Toronto, Canada

###### **Correspondence:** Lynn R. Spiegel

**Introduction:** Juvenile Idiopathic Arthritis (JIA) is a common chronic childhood illness that can negatively impact health-related quality of life (HRQL). Adolescents are expected to assume greater responsibility in disease management as they mature. However, the vast majority of adolescents with JIA do not receive comprehensive self-management education. Internet-based interventions provide an innovative approach to improve the *accessibility* and *acceptability* of self-management programs for adolescents with chronic illnesses.

**Objectives:** A randomized control trial was conducted to determine the effectiveness of an Internet-based self-management intervention. We hypothesized that youth with JIA in the Internet self-management intervention (Teens Taking Charge or TTC) group will demonstrate: (a) reduced pain and improved HRQL (primary outcomes); (b) reduced emotional (anxiety and depression) symptoms; and (c) Increased treatment adherence, pain coping, JIA-specific knowledge, and self-efficacy compared to adolescents in the control group (static web-based education).

**Methods:** Participants were between 12-18 years old with JIA across 11 pediatric centers in Canada. Teens participated with a parent/caregiver. Teens in the intervention reviewed 12 modules focused on disease education, self-management strategies and social support. Teens in the control condition reviewed standard disease education modules without self-management skills or social support. As part of the 3-month program, teens had monthly telephone check-ins with health coaches, but only intervention participants reviewed modules with their coach. Parents in both groups reviewed modules focused on promoting independence and disease self-management in their teen. Teens and parents completed outcome measures at 4 time points: baseline, at program completion (3-months), 6-months, and 12-months after the program.

**Results:** In total, 333 teens (n = 109 male, n = 224 female; mean age = 14.5, SD = 1.7) and 306 parents (n = 52 male, n = 254 female) were enrolled. Of the 164 participants in the intervention group, 62.8% (n = 103) completed the study over an average 189.8 days (SD = 113.5). Of the 169 control participants, 87.0% (n = 147) completed the study over an average 123.6 days (SD = 70.6). Results indicated that average weekly pain interference in enjoying daily life (p = 0.004) and PedsQL treatment problems (p = 0.010) improved significantly in the intervention group as compared to the control group. Improvements in self-efficacy, disease knowledge, health related quality of life and pain coping were shown at 3-month follow-up, when compared to baseline for both intervention and control groups. There were no changes in either group for anxiety, depression or adherence.

**Conclusion:** Teens with JIA overall are satisfied with using an online program for education and learning self-management skills, with improvement in pain interference and HRQOL deriving in part from increased knowledge and self-efficacy. Education and nonspecific factors (e.g., feeling part of a community) may be key determinants of improving important patient reported outcomes through the online self-management program.

**Disclosure of Interest**

None Declared.

### P251 3 year follow-up of acute-JIA cohort - aggressive combination drug therapy in very early polyarticular juvenile idiopathic arthritis

#### Pirjo Tynjälä^1,2^, Maarit Tarkiainen^1,3^, Paula Vähäsalo^4^, Liisa Kröger^5^, Kristiina Aalto^1^, Katariina Rebane^1^, Merja Malin^6^, Kati Markula-Patjas^6^, Eija Piippo-Savolainen^5^, Pekka Lahdenne^1^

##### ^1^Pediatric Rheumatology, Children’s Hospital, Helsinki University Central Hospital, Helsinki, Finland; ^2^Poison Information Centre, Helsinki University Central Hospital, Helsinki, Finland; ^3^University of Helsinki, Helsinki, Finland; ^4^Pediatrics, Oulu University Hospital, Oulu, Finland; ^5^Pediatrics, Kuopio University Hospital, Kuopio, Finland; ^6^Pediatrics, Tampere University Hospital, Tampere, Finland

###### **Correspondence:** Maarit Tarkiainen

**Introduction:** We have previously reported 54-week results of ACUTE-JIA trial, comparing 3 treatment arms without initial oral steroids; single methotrexate (MTX), synthetic combination DMARDs of methotrexate, sulfasalazine and hydroxychloroquine (COMBO), and infliximab plus methotrexate (TNF) in very early, DMARD-naïve polyarticular JIA (juvenile idiopathic arthritis). Prior to study onset, the mean duration of arthritis was 1.9 months (SD 0.1) [1] The extension trial included follow-up visits at 3 and 5 years. We present now 3-year results.

**Objectives:** To detect potential clinical differences at 3 years between the initial treatment arms.

**Methods:** Open label extension study. 60 patients continued on their originally randomized treatment until they failed the strategy or achieved inactive disease (ID) on medication for at least 6 months. When applicable, data was analyzed both on-group (within original treatment arm) and on-strategy (within treatment strategy, last observation carried forward if switch of strategy was chosen), using χ^2^, nonparametric testing and regression analyses. ACRp 30-100 (on-strategy) and achieving (ID) were assessed.

**Results:** All 60 patients in the original trial completed the 3-year follow-up. 50 patients (83%) switched treatment strategies. Changes were due to ID in 27 (45%), inefficacy in 20 (33%) and adverse events in 3 (5%). TNF was not discontinued in any patients due to inefficacy, although in 4 infliximab was switched to other anti-TNFs due to side-effects or patient preference.

At 3 years, 8 patients (40%) on TNF, 6 (30%) on COMBO and 6 (30%) on MTX arm were still receiving the original drug strategy. 21 patients (35%) were receiving biological therapy, including 13 failures from COMBO and MTX arms.

ACRp100 (on-strategy) was achieved in 80% of those receiving TNF, 40% on COMBO and 35% on MTX (p = 0.008). ACRp90 was achieved in 90% on TNF, 50% on COMBO and 50% on MTX (p = 0.025); ACRp70 in 95%, 55% and 50% (p = 0.028); and ACRp50 and 30in 100%, 55% and 50% (p = ns), respectively.

At 3 years, 16 patients (80%) receiving TNF, 11 (55%) COMBO and 6 (30%) MTX reached ID (on-strategy, p = 0.008). When the number of patients reaching ID in each original group was assessed between groups, no differences were detected (p = 1.000). However, mean time spent in ID (on-group) was 23.5 months on TNF, 16.3 on COMBO, and 14.7 on MTX (p = 0.029). Strategy and ID correlated strongly based on Spearman’s rho (p = 0.002). Furthermore, when adjusted with duration of biologic treatment in each group, time spent in ID was higher in those receiving TNF than COMBO (p = 0.001) or MTX (p < 0.001).

**Conclusion:** Clinical inactivity in JIA seemed to correlate with both on-going and early initial drug treatment. Although very early first-line infliximab was highly effective to control the activity of JIA and protective against long-term damage, it was not able to completely turn off rheumatic activity. In most patients, JIA flared after discontinuation of anti-TNF agents.

**Reference**

Tynjälä, Vähäsalo, Tarkiainen, et al. Aggressive combination drug therapy in very early polyarticular juvenile idiopathic arthritis (A-CUTE-JIA). A multicenter randomized clinical trial. *Ann Rheum Dis* 2011;70:1605-12.

**Trial registration identifying number:** NCT01015547

**Disclosure of Interest**

None Declared.

### P252 Oral or subcutaneus methotrexate: comparison of the efficacy in inducing sustained disease remission in children with oligoarticular JIA

#### Chiara Trincianti^1^, Maria Francesa Gicchino^1^, E. H. Pieter van Dijkhuizen^2^, Benedetta Schiappapietra^1^, Eleonora Zaccheddu^1^, Gabriella Giancane^1^, Giulia Bracciolini^1^, Denise Pires Marafon^3^, Silvia Magni-Manzoni^3^, Luca Villa^1^, Carlo Gandolfo^1^, Fabrizio De Benedetti^3^, Nicolino Ruperto^1^, Angelo Ravelli^1^, Alessandro Consolaro^1^

##### ^1^Istituto Giannina Gaslini, Genova, Italy; ^2^University Medical Center, Utrecht, Netherlands; ^3^Ospedale Pediatrico Bambino Gesù, Roma, Italy

###### **Correspondence:** Chiara Trincianti

**Introduction:** Methotrexate (MTX) is widely adopted as a first line treatment in moderate to severe forms of juvenile idiopathic arthritis (JIA), when NSAIDs and intra-articular corticosteroid injections are not sufficient to control joint disease. MTX is generally prescribed at 10-15 mg/m^2^ weekly and its administration can be either oral or parenteral (subcutaneous (SC) or intramuscular). Contrasting evidence is available in the literature about the difference in efficacy and safety of MTX, according to the route of administration.

**Objectives:** Aim of the study is to compare the efficacy of oral versus SC MTX in inducing sustained disease remission in children with oligoarticular JIA enrolled in two prospective cohorts.

**Methods:** Children with oligoarthritis included in 3 prospective studies were considered for inclusion: a) the TRIMECA trial (1), b) the MD-Paedigree study (2), c) the PharmaChild registry. Patient evaluated at the IRCCS Istituto Giannina Gaslini and at the Ospedale Pediatrico Bambino Gesù were included if they had received methotrexate treatment as a first line systemic medication within 6 months after disease onset and if a follow up of at least 12 month after treatment initiation was available. Patients were then grouped according to the route of MTX administration. Baseline demographic and disease features were compared between the 2 groups. Efficacy was assessed by comparing the rate of inactive disease (ID) and clinical remission on medication (CRM) at 12 months, the rate of patients changing the route of MTX administration or requiring a biologic medication due to treatment failure. Safety was assessed by comparing the frequency of treatment interruption due to side effects of MTX.

**Results:** 79 patients were included in the study: 43 received oral MTX, 36 received SC MTX. At treatment initiation, disease duration was not different in the two groups; children receiving SC MTX were older at baseline (4.6 yrs vs. 2.5 yrs) and at disease onset (4.2 yrs vs. 2.3 yrs). Disease activity was comparable in the 2 groups, with a median of 2 active joints in both groups. Median MTX dose was 14.4 mg/m^2^ for oral MTX group and 15.2 mg/m^2^ for SC MTX (Mann-Whitney U test, p < 0.01). At 12 months, children receiving SC MTX achieved more frequently ID (84.9% vs 43.8%, Chi squared test p < 0.001) and CRM (54.5% vs 28.3%, p = 0.002). Patients in SC MTX group were more often prescribed a biologic medication (22.9% vs 6.5%, p = 0.024), but none of them were switched to the oral administration while 37% of children in oral MTX group were turned to SC MTX. One patient in both groups had MTX treatment suspended due to side effects.

**Conclusion:** Our preliminary results support the evidence of an increased efficacy of MTX in inducing sustained disease remission when it is administered subcutaneously.

**Disclosure of Interest**

None Declared.

## JIA (oligo, poly, psoriatic)

### P253 How do paediatric pain experts and healthcare professionals prioritise patient-reported pain information from children with juvenile idiopathic arthritis?

#### Rebecca R. Lee, Amir Rashid, Ghio Daniela, Wendy Thomson, Lis Cordingley

##### NIHR Manchester Musculoskeletal Biomedical Research Unit, CMFT, Arthritis Research UK Centres for Epidemiology and Genetics and Genomics, Centre for Musculoskeletal Research, Division of Musculoskeletal and Dermatological Sciences, University of Manchester, Manchester, UK

###### **Correspondence:** Rebecca R. Lee

**Introduction:** Patient self-report of pain using multi-dimensional assessment is best practice, although uncertainty about which pain aspects are prioritised in guiding pain management decisions may prevent the uptake of this approach in paediatric rheumatology.

**Objectives:** To determine which features of multidimensional pain information from individuals with Juvenile Idiopathic Arthritis (JIA) were viewed as most salient by pain experts and paediatric rheumatology healthcare professionals.

**Methods:** Participants were recruited via online advertisements posted by their professional organisations to take part in two separate group workshops. Workshops were hosted at an international conference on pain and a national conference on paediatric rheumatology. Participants were asked to rank order nine vignette scenarios from ‘highest pain’ to ‘lowest pain’ using patient data collected with an electronic multidimensional pain assessment tool (My Pain Tracker). The pain components collected by the tool were developed by children with JIA and included pain intensity, severity, location and spread (demonstrated through colour/shading), pain quality descriptors (including symbols such as fire and sharpness) and emotions. Participants were asked to explain their individual reasoning for their rankings of pain experiences aloud within workshops. Recordings were transcribed and analysed using content analysis.

**Results:** Nineteen participants took part: nine paediatric pain experts and ten paediatric rheumatology healthcare professionals. The high prioritisation of pain intensity and severity was uncontentious between and within groups. Differences between groups emerged in the prioritisation of specific pain location information. For pain experts, the number of sites affected was important. However, for healthcare professionals, localised pain around specific joints was seen as high priority. This suggests that this group of participants couple pain information with disease activity indicators to guide pain management decisions in children with JIA. Despite the importance of location information for both groups, spread of pain (e.g. radiating pain demonstrated through the use of colour) was not important for either group. Another area of disparity was in the prioritisation of pain emotion. Both groups talked about needing further confirmatory evidence for them to interpret emotion scores with confidence as this component could often be inconsistent with other pain information. However, for pain experts, data about function and interference was important and for healthcare professionals, corresponding information about disease activity was valuable. Lastly, qualitative descriptors of pain were a low priority for both groups unless children used the ‘fire’ symbol. This again suggests that evidence of inflammatory pain processes were viewed as significant.

**Conclusion:** Not all of the pain components collected by My Pain Tracker are used to inform pain management decisions, although children identify these as important in helping them to effectively communicate about their pain experiences. Results demonstrate which pain facets are most salient in the visualisation of electronic multi-dimensional pain data. Our findings will be incorporated into interpretive guidelines for such assessments in children with musculoskeletal pain.

**Disclosure of Interest**

None Declared.

### P254 Pain and the effect on every day life in children with juvenile idiopathic arthritis

#### Birgitte Mahler, Simon Esbersen, Troels Herlin

##### Pediatric Rheumatology, Arhus University Hospital, Arhus, Denmark

###### **Correspondence:** Birgitte Mahler

**Introduction:** Despite the introduction of new and effective biological disease-modifying anti-rheumatic medication, children with juvenile idiopathic arthritis still report pain and discomfort during every day life. Pain has been found to be a major contributor to morbidity in this group of children.

**Objectives:** We wanted to access the effect of pain and disease activity on perceived health and strain on the family.

**Methods:** Inclusion criteria were age 6-16 years and JIA with active disease or in remission on medication. During routine visits at pediatric rheumatology clinic, the parents and child were asked to fill out validated questionnaires - Juvenile Arthritis Multidimensional Assessment Report (JAMAR), Well-being index WHO-5 (score 5-25) and to answer questions about social status and activities of daily living. For assessment of disease activity “Juvenile arthritis disease activity score” (JADAS-27) was completed.

**Results:** Data is reported as median (minimum; maximum):

From December 2016 to April 2017, 92 consecutive JIA patients met the inclusion criteria. 13 were excluded due to other significant disease or inability to communicate in Danish, 7 declined to participate. A total of 51 patients completed the questionnaires. Patient characteristics are seen in Table 20. Disease activity according to JADAS27 was 2. (0;16.6). Days spent in relation to care of arthritis or arthritis related symptoms in a year was 12 (0;104). The parents reported need of special assistance in relation to arthritis symptoms in 30% of school children with JIA and 15% of children with JIA in day-care. In children in need of special attention or extra follow-up visits, there was pronounced diversity with regards to how the families perceived this as stressful or not. Thus 19 out of 51 parents reported a score of 4 or above (on a 0-10 VAS scale) when asked whether finding help for follow up or home-care was difficult or not median 3 (0; 6.9).

Pain during the previous week (according to JAMAR) was VAS 1.5 (0; 7.9). In psychological well-being according to WHO-5 they generally performed well 76 (36;100), no patients were at high risk of depression, but a small group of patients (9%) were found to have low mood and at moderate risk of depression.

There was no correlation between pain, disease activity and low WHO-5 score or the number of days spend taking care of the arthritis.

**Conclusion:** Self-reported maximum pain and disease activity did not explain the variance in family time spend on JIA related disease and disease management. Despite a high score in well-being compared to general childhood population, a small group of JIA patients are still at moderate risk of depression.

**Disclosure of Interest**

None Declared.

**Table 20 (Abstract P254).** See text for description

Number of patients	51
Girls/Boys	39/12
Age (years)	13.12 (6.75;16.74)
DMARD and/or biological medication	35
sJIA, poly RF+, poly RF-, oligo, enthesitis, PsA, Undifferentiated	10%, 0, 27%, 37%, 4%, 11%, 11%

### P255 Prescribing patterns in juvenile idiopathic arthritis: a survey of current practice in the United Kingdom

#### Daniel Hawley^1^, Helen E. Foster^2^, Michael W. Beresford^3^, Athimalaipet Ramanan^4^, Tim Rapley^5^, Flora McErlane^6^

##### ^1^Paediatric Rheumatology, Sheffield Children’s Hospital, Sheffield, UK; ^2^Institute of Cellular Medicine, Newcastle University, Newcastle upon Tyne, UK; ^3^Institute of Translational Medicine, University of Liverpool, Liverpool, UK; ^4^Paediatric Rheumatology, Bristol Royal Hospital for Children, Bristol, UK; ^5^Institute of Health and Society, Newcastle University, Newcastle upon Tyne, UK; ^6^Paediatric Rheumatology, Great North Children’s Hospital, Newcastle upon Tyne, UK

###### **Correspondence:** Flora McErlane

**Introduction:**

The 2015 NHS-England (NHSE) interim policy statement and 2015 NICE Technology Appraisal for the treatment of JIA (TA373) are national treatment guidelines informing optimal prescribing of biologics in JIA in the UK. It is not yet clear whether UK based clinicians routinely treat new patients according to the NHSE interim statement/NICE TA373. Although funding for biologics in England and Wales is dependent on adherence to the interim policy statement, there is no obligation for UK clinicians to follow NICE guidance.

Understanding current prescribing patterns in children and young people (CYP) with JIA is a necessary precursor to development of targeted early treatment pathways (treat-to-target regimes).

**Objectives:**

1. To describe prescribing patterns in CYP presenting to UK paediatric rheumatology (PRh) teams with a new diagnosis of JIA, with reference to the national treatment guidelines.

2. To collate opinion regarding targeted early treatment pathways in CYP with new-onset polyarticular-pattern JIA.

**Methods:**

In March 2016, 19 UK-based PRh consultants (1 representative from each NHSE PRh provider) were invited to complete an online survey. Respondents were asked to provide data regarding numbers of patients diagnosed between 2015 and 2016 with oligoarticular and polyarticular-pattern JIA, current treatment approaches (with reference to national treatment guidelines) and opinion regarding targeted treatment pathways and earlier use of biologics.

**Results:**

14/19 (74%) responders from England (n = 12), Scotland (n = 1) and Northern Ireland (n = 1) returned the questionnaire. The median number of patients per centre with new-onset oligoarticular and polyarticular-pattern JIA was 19 (IQR 14-40) and 10 (IQR 6-20) respectively. 85% respondents reported ‘always’ or ‘mostly’ treating patients according to the NHSE interim policy.

The following four practices relate directly to key guidelines set out in the NHSE interim policy:

1. Prescribing of intra-articular corticosteroids during initial treatment of new presentation JIA: Reported in 100% and 92% of oligoarticular and polyarticular-pattern disease respectively. Co-administration of oral or intravenous corticosteroids was reported. Theatre availability, joint involvement, disease severity and patient choice influenced choice of steroid regime.

2. Prescribing of first-line DMARD in oligoarticular-course disease: Methotrexate (MTX) was the first line DMARD for all patients, in accordance with national guidance. MTX was prescribed for persistently severe disease and/or hip, wrist, finger or temporo-mandibular joint involvement were the most common reasons for introduction of MTX.

3. Prescribing of first-line DMARD (MTX) in polyarticular-course disease: 100% respondents commenced MTX at presentation, in accordance with national guidance.

4. Prescribing of anti-TNF at baseline: The NHSE interim statement supports anti-TNF at presentation if sacroiliitis or axial arthritis is present. Four (29%) respondents reported using anti-TNF as first-line treatment and prescribing patterns did not always follow national guidance (reasons included spondyloarthropathy, enthesitis, sacroiliac involvement or positive HLA-B27).

Noting the small numbers of responders, there were trends of lower adherence to the NHSE interim policy statement in Scotland and Northern Ireland.

45-77% respondents (variation by ILAR subtype) would consider prescribing anti-TNF and MTX at presentation if guidelines/funding allowed.

All respondents reported interest in recruiting to a study trialing a targeted treatment pathway in children with polyarticular-pattern JIA.

**Conclusion:**

1. The majority of responding UK PRh centres prescribe biologics according to the national treatment guidelines (NHSE interim policy statement and NICE guidance).

2. Clinicians already prescribe anti-TNF medication at baseline and prescribing patterns do not always follow national guidance.

3. There is overwhelming support for a targeted care pathway study in polyarticular-pattern JIA.

**Disclosure of Interest**

None Declared.

### P256 The usability of cell phone based automated monitoring of patients with juvenile idiopathic arthritis

#### Katriina Mikola^1^, Kristiina Aalto^1^, Pekka Lahdenne^1^, Hannu Kautiainen^2^

##### ^1^Children’s Hospital, Helsinki University Central Hospital, Helsinki, Finland; ^2^Medcare Oy, Helsinki, Finland

###### **Correspondence:** Katriina Mikola

**Introduction:** The early diagnosis and institution of drug treatment in early phase improve the outcome in juvenile idiopathic arthritis (JIA). The new drug treatment requires intensive education and following. In healthcare there are many ways to promote adherence. Studies also suggest that frequent contacts between the patient and the clinic also enhance the adherence but resources are often limited. There is obvious need to improve communication between the patient and the clinic.

**Objectives:** To study the validity and feasibility of the SandRA. A Finnish work group has recentely developed a treatment monitoring software called SandRA (Showing any need for Re-Assessment). It is based on automated cell phone text messages and has proved to be a valid and useful tool among the adult rheumatoid arthritis patients.

**Methods:** Patients visiting the rheumatologic outpatient clinic of Helsinki Children’s Hospital were asked to participate the study at the time when new medication was introduced. 21 JIA patients volunteered to this pilot study with 20 methotrexate and 1 etanercept inceptions. Patients fulfilled the criteria of the International League of Association for Rheumatology (ILAR) for JIA. 10 (48%) had oligoarthritis, 9 (43%) polyarthritis, one rheumatoid factor positive disease and one psoriarthritis. None had uveitis.

The SandRA-system automatically sends text messages (questions) to the patient’s/parent’s cell phone every two weeks for six months. The questions concern medication, possible side-effects and laboratory results. The follow-up parameters we collected at routine visits (0, 3 and 6 months).

The outcome was evalueted with clinical criteria of remission, JADAS-10 and CHAQ.

**Results:** Of the 21 patients 15 were female (76%). Mean age was 8,6 years (SD 4,2 years). One patient was ANAab positive and one rheumatoid factor and ACPA positive. HLAB27 positivity was found in 7 patient (37%).

95% of parents found SandRA-system easy to use. 3 parents (14%) wished more direct contacts with the clinic. 12 (57%) considered SandRAas a useful additional tool and 76% were interested to use it in the future.

At 6 months after drug treatment initiation, 11 (52%) patients were in clinical remission. JADAS-10 was 8,2 and 3,1 in the baseline and in the 6 months visit, respectively (P < 0,001) (Table 21).

**Conclusion:** SandRA was considered as a promising tool in enhancing the adherence to treatment and thus, achieving remission in JIA among families and health care workers. Next, we plan to introduce a 6-12 months randomized controlled study. Presumably some modifications will be made in the system, for example timing of the questions needs adjustment. Other digital methods than text messages might also improve participation of teenagers.

**Disclosure of Interest**

None Declared.

**Table 21 (Abstract P256).** Results

S	Baseline	3 month visit	6 month visit
Number of active joints mean (SD)	4 (3)	1 (1)	1 (2)
Number of joints with limited movement mean (SD)	3 (3)	1 (1)	1 (2)
ESR (mm/Hg) mean (SD)	15 (11)	7 (6)	7 (3)
CHAQ mean (SD)	0,37 (0,48)	0,18 (0,32)	0,23 (0,34)
JADAS10 mean (SD)	8,2 (2,4)	3,8 (3,5)	3,1 (3,8)
Patient global VAS mean (SD)	31 (20)	15 (17)	13 (16)
Patient pain VAS mean (SD)	17 (19)	17 (19)	15 (21)
Physicians global VAS mean (SD)	30 (11)	9 (10)	9 (14)

### P257 Adipokines in juvenile idiopathic arthrtis, are they usefull as inflammatory activity markers?

#### Esmeralda Nuñez-Cuadros^1^, Rocio Galindo Zavala^1^, Gisela Díaz-Cordovés Rego^1^, Lourdes Artacho González^2^, Antonio L. Urda Cardona^2^

##### ^1^Paediatric Rheumatology, Universitary Regional Malaga Hospital, MALAGA, Spain; ^2^Paediatrics, Universitary Regional Malaga Hospital, MALAGA, Spain

###### **Correspondence:** Esmeralda Nuñez-Cuadros

**Introduction:** Adipokines are hormones, mostly secreted by adipose tissue, whose metabolic effects in inflammatory diseases are controversial. There are few researches about adipokines in Juvenil Idiopathic Arthritis (JIA), being unclear their relation with inflammatory activity.

**Objectives:** To compare plasma levels of adipokines between inactive/in remission and active JIA patients and to analize clinical and analytical factors potentially related to inflammatory activity in this disease.

**Methods:** Observational cross-sectional study in JIA Spanish patients from 4 to 15 years old, monitored by a Pediatric Rheumatology Unit. Monoarticular forms, psoriasic, enthesitis related arthritis, indifferenciated subtypes and patients with other chronic diseases were excluded.

Anthropometric, clinical and treatment data were recorded and Juvenile Arthritis Disease Score 27 (JADAS27) was calculated for each patient. Acute phase reactants, including high sensitive RCP (hsCRP), TNF -α and IL-6, and adipokines as leptine, adiponectine and resistine were determined.

Activity, inactivity and remission were defined using Wallace criteria (2011).

**Results:** 80 patients were enrolled, 70% women, median age 11 years (IQR 3.7-15.7). Mean time of disease evolution was 6,5 years ((±3,7 DS) and mean JADAS27 was 2 ((±4DS).63.8% were oligoaricular (47.5% persistent), 25.1% polyarticular and 11.3% systemic JIA. Only 26.3% showed active disease when the research was performed.

No differences in mean adipokines levels were found between different JIA subtypes, depending on received treatments, neither between active and inactive/in remission patients (Table 22).

In bivariant analysis possitive relation between resistine and hsCRP (Rho 0,40; p < 0,001), between resistine and IL-6 (Rho 0,40; p < 0,001) and between adiponective and TNF -α (Rho 0,29; p 0,009) were found. Afterwards, a multivariate analysis was performed using JADAS27 as dependent variable. We found a direct relation to IL-6 (ß 0.364; p <0.001) and leukocyte count (ß 0.484; p <0.001; CI 95%).

**Conclusion:** Adipokines are not diferent between active and inactive/in remission JIA patients in our sample, perhaps because of its low inflammatory activity (mean JADAS27 = 2).

Resistine and adiponectine could have a proinflammatory function.

Although most patient in our sample had oligoarticular subtype, IL-6 showed to be directly related to inflammmatory activity measured by JADAS27, what could have therapeutic implications.

**Disclosure of Interest**

None Declared.

**Table 22 (Abstract P257).** See text for description

	Inactive/Remission (n = 59)	Active (n = 21)	p
IL-6 (pg/ml), median (IQR)	1.3 (0.2-31.2)	2.6 (0.3-92.2)	0.24
TNF - α (pg/ml), median (IQR)	2.3 (0.1-384.6)	2.6 (1.1-182.1)	0.64
Adiponectine (ng/ml), mean (±SD)	12363.7 (5054.1)	10804.7 (5054.8)	0.13
Resistine (ng/ml), mean (±SD)	5.1 (1.8)	5.5 (1.6)	0.29
Leptine (ng/ml), mean (±SD)	8.7 (11.2)	13.3 (13.3)	0.31
hsRCP (mg/l), median (IQR)	0.5 (0 -14.7)	1.84 (0.1-16.8)	0.01
RCP (mg/l), mean (±SD)	3.6 (3.12)	9 (19.5)	0.05
ESD (mm/h), mean (±SD)	7.3 (4.6)	13 (11.3)	0.11

### P258 A population based study on screening for celiac disease and levels of celiac-related antibodies in children with juvenile idiopathic arthritis (JIA)

#### Anders Öman^1^, Tony Hansson^1^, Martin Carlsson^2^, Lillemor Berntson^1^

##### ^1^Pediatric Department, Uppsala University Children’s Hospital, Sweden, Uppsala, Sweden; ^2^Department of Women’s and Children’s Health, Uppsala University Children’s Hospital, Sweden, Uppsala, Sweden

###### **Correspondence:** Anders Öman

**Introduction:** In recent studies the immunological situation in the intestinal canal in children with JIA have been discussed in aspects of dysbiosis of microflora, increased permeability and an aberrant regulation of lymphocytes in the mucosa. JIA is also associated with an increased risk of other autoimmune diseases such as type 1 diabetes mellitus and thyroid disorders. It has been suggested that children with JIA also have an increased risk of developing celiac disease but no population based studies have been made and exclusion of IgA deficiency has not always been part of the protocol.

**Objectives:** To study the prevalence of celiac disease in a population based cohort of children with JIA, by screening for IgA anti-TG2 antibodies, total IgA and IgG anti-TG2 antibodies and to study levels of those antibodies in the different categories of the disease.

**Methods:** Every child diagnosed with JIA in three Swedish counties (Uppsala, Gävleborg and Dalarna), with disease onset between January 1, 2007 and December 31 2014, were included prospectively. Classification according to the ILAR criteria and analysis of IgA, IgG anti-TG2 antibodies and total IgA from serum samples were performed.

Children with IgA anti-TG2 antibodies above 3 U/mL and children with IgA deficiency in combination with IgG anti-TG2 antibodies above 3 U/mL were referred to the pediatric gastroenterology unit for further investigation with gastroscopy and small intestine biopsy.

**Results:** 216 children with JIA were included and analysis of IgA and IgG anti-TG2 antibodies was performed on 214 of these children. Three children had a diagnosis of celiac disease prior to the diagnosis of JIA but by screening the cohort we found three additional children with raised levels of IgA anti-TG2 antibodies. Small intestinal biopsy confirmed the diagnosis of celiac disease for all of them. One of these children had gastrointestinal symptoms prior to our screening, but the other two children were asymptomatic. Total serum level of IgA was analysed in 213 children and two children had an IgA deficiency (0.94%). One of the children with IgA deficiency had a raised IgG anti-TG2 level and was referred to the pediatric gastroenterology unit, but small intestinal biopsy showed no signs of celiac disease.

In this population based study we found a point prevalence of 2.8% (n = 6) for celiac disease in children with JIA, and the prevalence increased by screening. Levels of IgA correlated with age at sampling.

**Conclusion:** The present study does not support a higher occurrence of celiac disease in JIA compared to healthy children but it reminds us that screening for celiac disease can detect asymptomatic cases. We believe that all children with JIA should be screened for celiac disease.

**Disclosure of Interest**

None Declared.

### P259 Early onset ana-positive JIA in the swedish paediatric rheumatology registry

#### Bo Magnusson^1^, Soley Omarsdottir^1^, Eva Hagel^2^ and Board of the Registry

##### ^1^Astrid Lindgren Children’s Hospital, Stockholm, Sweden; ^2^Karolinska institutet, Stockholm, Sweden

###### **Correspondence:** Soley Omarsdottir

**Introduction:** Early onset ANA-positive JIA has been suggested as a potential subgroup of JIA. It must be proved whether this subgroup is a separate entity regarding basic characteristics, disease mechanisms, uveitis, prognosis, risk for damage and need for treatment.

**Objectives:** To describe early onset ANA-positive JIA as a subgroup of JIA with data from the Swedish Paediatric Rheumatology Registry and compare the study group with the remaing group of JIA.

**Methods:** The Swedish Paediatric Rheumatology Registry started in 2009. The national coverage is above 60% for all JIA and above 90% for JIA treated with antirheumatic drugs. We have identified a subgroup of JIA with positive ANA and onset ≤ 6 years of age with exclusion of ERA, RF pos arthritis and systemic JIA and used data in the registry to describe the selected group regarding sex, treatment, uveitis and JADAS.

**Results:** 334 patients with early onset ANA positive JIA where identified out of 2667 patients with JIA. In 1464 patients there were missing data on ANA in the registry. 78% in the study group were females (64% in remaining group). The mean age at onset was 2.7 years (8.2 years in remaining group). Uveitis at any time in 17% (2% in remaining group) but data missing in 58% (87% remaining group). Treatment with methotrexate at any time in 74% (remaining group 62%) and biologics 54% (remaining group 43%). No differences in JADAS from onset during first 15 months could be found.

**Conclusion:** Early onset ANA-positive JIA patients extracted from the Swedish Paediatric Rheumatology Registry have most but not all cases of uveitis. There are no big differences in treatmen with methotrexate or biologics compared to the remaining group of JIA. JADAS during the first 15 months from onset is equal to remaining patients. Future will show whether prognosis and remission is different in early onset ANA-positive JIA compared to other subgroups of JIA.

**Disclosure of Interest**

None Declared.

### P260 Associated autoimmune diseases in children with juvenile idiopathic arthritis

#### Aida Omerčahić Dizdarević^1^, Velma Selmanović^1^, Adisa Čengić^1^, Snježana Šabanović^2^, Mithat Mujić^2^

##### ^1^Allergology, Rheumatology, Clinical Immunology, Childrens hospital, University clinical center, Sarajevo, Bosnia and Herzegovina; ^2^Endocrinology, Childrens hospital, University clinical center, Sarajevo, Bosnia and Herzegovina

###### **Correspondence:** Aida Omerčahić Dizdarević

**Introduction:** Juvenile Idiopathic Arthritis (JIA) is autoimmune disease. Children with JIA are prone to have associated autoimmune diseases (AIDs).

**Objectives:** To identify JIA children with associated autoimmune diseases diagnosed in our tertial center.

**Methods:** This is a retrospective study on JIA children diagnosed in department of pediatric rheumatology from April 2007 until March 2017 years. The medical records were reviewed. In each case gender and JIA subgroup were identified. Thyroid hormones, thyroid stimulating hormone – TSH and thyroid antibodies were performed in all children with systemic JIA (sJIA), in others, when thyroid disease was suspected.

**Results:** During the study period, 149 JIA children were diagnosed (Oligoarthritis-56; Polyarthritis-40; Enthesitis realted arthritis-18; Psoriatic arthritis-7; Systemic arthritis-28). Distribution of genders were: females 108, males 41. Sixteen (females 11, males 5) JIA children (10,7%) had one or more associated AIDs. Fourteen children had autoimmune thyroid disease (totally 9,4%, in sJIA group 17,8%). One child had four (polyarticular RF negative JIA, autoimmune thyroid disease, type 1 diabetes and coeliac disease) and one had three (polyarticular RF negative JIA, autoimmune thyroid disease, type 1 diabetes) AIDs. In one child, with polyarticular RF negative JIA, allopecia and vitiligo were associated and other one, with polyarticular JIA, had celiac disease. JIA subgroups in JIA associated AIDs group were: one olygoarthritis (ANA negative), seven polyarhritis (five RF negative, one RF positive), three enthesitis related arthritis (one HLA B27 positive, two negative) and five sJIA.

**Conclusion:** Most frequent associated AID was autoimmune thyroid disease. Two children had three nad four autoimmune diseases. These data suggest monitoring of thyroid function in JIA children.

**Disclosure of Interest**

None Declared.x

### P261 Long-term impact of juvenile idiopathic arthritis on quality of life of adult patients in Greece

#### Georgios Charis Paskalis^1^, Despoina Dimopoulou^2^, Maria Trachana^3^, Polykseni Pratsidou-Gertsi^3^, M Stavrakidou^2^, Theodora Oikonomou^2^, Theodoros Dimitroulas^2^, Alexandros Garyfallos^2^

##### ^1^Aristotle University of Thessaloniki, Thessaloniki, Greece; ^2^4th Department of Internal Medicine, Pediatric Immunology and Rheumatology Referral Centre, Aristotle University of Thessaloniki, Thessaloniki, Greece; ^3^1st Department of Pediatrics, Pediatric Immunology and Rheumatology Referral Centre, Aristotle University of Thessaloniki, Thessaloniki, Greece

###### **Correspondence:** Georgios Charis Paskalis

**Introduction:** Juvenile Idiopathic Arthritis (JIA) is the most common rheumatic disease in childhood and affects negatively both physical and psychosocial functioning.

**Objectives:** To explore the long-term impact of JIA on quality of life of adult patients in Northern Greece.

**Methods:** Adult patients with a definite diagnosis of JIA were assessed by the SF-12v2 questionnaire in the outpatient transition (from Pediatric to adult Rheumatology) clinic of Hippokratio Hospital of Thessaloniki. SF-12 questionnaire is a quality of life assessment tool and consists of a Physical Component Summary (PCS) and a Mental Component Summary (MCS). PCS and MCS of the patient group were compared to an age-matched control group, using *t-test* or *Mann-Whitney U test*, as appropriate. Moreover, percentages of patients and controls who were severely affected (<45 points in PCS or MCS) were compared, using *Chi-squared test*. Finally, correlation between the two summary components of SF-12 of patients was measured, using *Spearman’s rho*. Statistical analysis was done using SPSS software. Differences more than 3 points in each component are considered clinically significant, and level of statistical significance is p < 0.05.

**Results:** A total of 50 patients and 135 controls were enrolled in the study. The median (IQR) patient age was 32 (10) years and the median (IQR) disease duration was 24 (9,5) years. The PCS scores of the patient group were statistically significantly lower compared to the control group (P = .021), but the difference didn’t reach the minimum clinical significance. Nevertheless, more patients than controls scored low values (<45) in PCS (22% versus 6.7%, odds ratio, 3.95 [95% CI, 1.53, 10.22]; P = .005). MCS scores of patients were slightly better than scores of controls (mean difference, 3.67 [95% CI, 0.44, 6.90]; P = .026), but the severely affected were similar in both groups (odds ratio, 0.59 [95% CI, 0.30, 1.17]; P = .13). No correlation between PCS and MCS was found (P = 0.48).

**Conclusion:** There is an apparent impact of JIA on many patients’ quality of life that persists for many years after disease onset, which is in line with studies from other countries. More than one fifth of the patients were found severely affected in terms of their physical functioning. This could be attributed to disease severity, disease subtype and duration, socioeconomic status and availability of treatment options. Interestingly, JIA was not found to affect the patients’ mental functioning. A more specific psychometric test would be appropriate for in-depth analysis and confirmation of this result. Study design did not allow sub-analysis of the results according to JIA subtype, disease severity or duration, highlighting the need for long-term outcome studies focusing on the risk factors which may be involved.

**Trial registration identifying number:** 1. Foster H, Marshall N, Myers A, Dunkley P, Griffiths I. Outcome in adults with juvenile idiopathic arthritis: A quality of life study. Arthritis & Rheumatism. 2003;48(3):767-775

2. Barth S, Haas J, Schlichtiger J, Molz J, Bisdorff B, Michels H et al. Long-Term Health-Related Quality of Life in German Patients with Juvenile Idiopathic Arthritis in Comparison to German General Population. PLoS One. 2016; 11(4)

3. Kontodimopoulos N, Pappa E, Niakas D, Tountas Y. Validity of SF-12 summary scores in a Greek general population. Health Qual Life Outcomes. 2007 Sep 28;5:55

4. User’s Manual for the SF-12v2 Health Survey, Third Edition, QualityMetric

5. Minden K, Niewerth M, Listing J, Biedermann T, Bollow M, Schöntube M et al. Long-term outcome in patients with juvenile idiopathic arthritis. Arthritis & Rheumatism. 2002;46(9):2392-2401

6. Anink J, Prince F, Dijkstra M, Otten M, Twilt M, ten Cate R et al. Long-term quality of life and functional outcome of patients with juvenile idiopathic arthritis in the biologic era: a longitudinal follow-up study in the Dutch Arthritis and Biologicals in Children Register. Rheumatology. 2015;54(11):1964-1969

**Disclosure of Interest**

None Declared.

### P262 Patients’ reported impact of temporomandibular joint (TMJ) involvement in juvenile idiopathic arthritis (JIA): development of a juvenile arthritis tmj self-reported assessment (JATA) questionnaire

#### Denise Pires Marafon^1^, Alessia Arduini^2^, Laura Tanturri de Horatio^3^, Domenico Barbuti^3^, Rebecca Nicolai^1^, Fabrizio De Benedetti^1^, Silvia Magni Manzoni^1^

##### ^1^Rheumatology, IRCCS Bambino Gesù Hospital, Rome, Italy; ^2^Pediatrics, Policlinico Umberto I, “La Sapienza” University, Rome, Italy; ^3^Radiology, IRCCS Bambino Gesù Hospital, Rome, Italy

###### **Correspondence:** Denise Pires Marafon

**Introduction:** Few standardized patients/guardians’ reported assessments include the impact of TMJ involvement in daily life in patients with JIA. Moreover, this evaluation is often limited and do not cover aspects which are considered relevant by JIA patients with TMJ arthritis.

**Objectives:** To develop a concise and comprehensive self-reported assessment of TMJ in patients with JIA.

**Methods:** Patients with JIA and clinical involvement of TMJ consecutively seen at the study center in September-December 2016, with at least 8 years of age, were asked to identify the daily activities most affected by TMJ arthritis. From the collected items, we asked them to select the most relevant and representative of “TMJ well-being/worsening” in their daily life. We included in the JATA the items that were most frequently selected and a patient’s global assessment (GA) of TMJ (VAS 0-10; 0 = worst; 10 = best). Finally, we asked to a subgroup of patients that underwent to intraarticular corticosteroid injection (IACI) of one or both TMJ to complete the report, based on their abilities before the therapeutic intervention (VAS 0-10; 0 = totally unable, 10 = fully able). Clinical TMJ assessment, dental and TMJ magnetic resonance (MR) reports just before TMJ IACI were blindly reviewed and associated to a score, based on the presence/absence of pathological features: clinical severity (CS) of TMJ, as presence (1) or absence (0) of swelling + pain on motion/tenderness + limited-range-of-motion (range 0-3); dental-score, as presence (1) or absence (0) of cavities + insufficient hygiene + pain on TMJ palpation + pain on muscle palpation + lateral deviation/micrognatia (range 0-6); MR-score, as presence (1) or absence (0) of joint effusion + synovial pannus + enhancement + condylar deformities + erosions + bonemarrow oedema (range 0-6). Correlations between JATA-score (range 0-30) and clinical, dental, and MR scores were calculated using Spearman coefficient test (statistical significance: p <0.05).

**Results:** Twenty (77%) out of the 26 eligible JIA patients eligible identified daily activities most relevantly affected by TMJ involvement. Then, the items most frequently selected were included in the JATA (Table 23). Eight patients (100% female, 62.5% with persistent oligoarthritis and 12.5% with extended oligoarthritis, polyarticular RF-negative, and polyarticular RF-positive, respectively) filled the JATA questionnaire reporting their abilities and global assessment on TMJ before TMJ IACI. The time to complete each questionnaire varied around 3-5 minutes. The median (IQ) JATA-score was 14.8 (11.9-17.8), JATA-GA 5 (3-5.5), CS-TMJ 2 (1.8-2), dental-score 1 (1-2.5), and MR-score 5 (3.5-5), respectively. Statistically significant correlation was found only between JATA-score and CS-TMJ (p = 0.04).

**Conclusion:** We developed a JATA questionnaire for the self-reported assessment on TMJ in patients with JIA, feasible and easy to apply. Further studies in larger cohorts are planned to establish its reliability and sensitivity to change.

**Disclosure of Interest**

None Declared.

**Table 23 (Abstract P262).** Items selected by patients and included in the Juvenile Arthritis Temporomandibular joint self-reported Assessment (JATA) questionnaire

1. Ability to bite a sandwich/an apple/a “bombolone”	
2. Ability to eat a pizza or a steak	
3. Ability to accurately wash the teeth	

### P263 Share Latin America – first steps

#### Ingrid H. Rotstein Grein^1, 2^, Cláudia S. Magalhães^3^, Benito G. Martin^4^, Alexis Strickler^5^, Ximena Norambuena^6^, Carmen Navarrete^7^, Yanira Gavidia^8^, Maurício Alegria^8^, Maria M. Katsicas^9^, Ricardo Russo^9^, Ana Carolina L. Held^10^, Luan C. Coelho^10^, Isaac Ferreira^11^, Hiago B. Gomes^12^, Nicolino Ruperto^13^, on behalf of PRINTO, Nico Wulffraat^2^

##### ^1^Pediatric Rheumatology, Hospital Infantil Pequeno Príncipe, Curitiba, Brazil; ^2^Pediatric Rheumatology, Wilhelmina Children’s Hospital/University Medical Centre Utrecht, Utrecht, Netherlands; ^3^Pediatric Rheumatology, Universidade Estadual Paulista/UNESP, Botucatu, Brazil; ^4^Pediatric Rheumatology, Hospital Calvo Mackenna y Clínica Las Condes, Santiago, Chile; ^5^Pediatric Rheumatology, Hospital Eduardo Schutz, Puerto Montt, Chile; ^6^Pediatric Rheumatology, Hospital Exequiel González Cortés, Santiago, Chile; ^7^Pediatric Rheumatology, Hospital Roberto del Rio, Santiago, Chile; ^8^Pediatric Rheumatology, Hospital Nacional de Niños Benjamín Bloom, San Salvador, El Salvador; ^9^Pediatric Rheumatology, Hospital de Pediatría Garrahan, Buenos Aires, Argentina; ^10^Pediatric Rheumatology, Universidade Federal de São Paulo/UNIFESP, São Paulo, Brazil; ^11^Pediatric Rheumatology, Instituto de Puericultura e Pediatria Martagão Gesteira/UFRJ, Rio de Janeiro, Brazil; ^12^Pediatric Rheumatology, Universidade Federal do Pará, Belém, Brazil; ^13^Pediatric Rheumatology, Instituto Gaslini, Genoa, Italy

###### **Correspondence:** Ingrid H. Rotstein Grein

**Introduction:** Since 2016 PRES has initiated a project called SHARE LA (Single Hub and Access point for Paediatric Rheumatology in Europe and Latin America, PI Wulffraat). This project is a collaboration between European and Latin American (LA) countries that aims to identify the specific needs for optimal care in Juvenile Idiopathic Arthritis (JIA) patients in different continents. The delay in diagnostic and treatment of JIA may cause permanent joint damage, which will negative impact the present and future life of the patients. In order to avoid the burden of disease, it is extremely important to optimise health care providing satisfactory access to the specialist, implementing multidisciplinary team follow up, as well as to provide easy access to adequate treatment. The quality of these variables will show how efficient the health care is. The first LA country to join this initiative was Brazil, followed by Argentina, Chile and El Salvador.

**Objectives:** To identify which health care aspects needs improvement in order to plan further optimal care to children with JIA in LA countries.

**Methods:** There is an international steering committee consisting of Dr. Ingrid H R Grein (Brazil and The Netherlands), Prof. Dr. Cláudia Saad Magalhães (Brazil), Dr. Ricardo Russo (Argentina), and Prof. Dr. Nico Wulffraat (The Netherlands). LA pediatric rheumatologists registered on PRINTO (Pediatric Rheumatology INternational Trials Organisation) were invited to participate in the project by email, in order to provide the liaison with established investigators and practicing pediatric rheumatologists in each of the participating countries. The surveys focused on families view on standard of care for children with JIA. Families answered a single time-point survey at pediatric rheumatology centers of the countries that joined the project. The original European questionnaire was translated into Portuguese and Spanish, and adapted with minor changes: questions about health insurance type, parents education level, transportation to the medical appointment, and access to standard anti-rheumatic medications were added. Families filled in the questionnaires anonymously on paper or on PRINTO website.

**Results:** Until May 2017, 535 families from Brazil, Chile, Argentina and El Salvador answered the questionnaires. The table shows the participant countries and the number of answered surveys of each one of them. Families view showed satisfactory follow up with pediatric rheumatologists, uveitis screening and access to high cost medications. However, the families that replied this survey may present a bias to urban population, where the pediatric rheumatology centers are.

**Conclusion:** The families view is an important tool to identify the topics on standard of care that need improvement, in order to achieve a high quality health care in any region of the world. The results of the first LA countries that participated in the SHARE LA project showed satisfactory follow up with pediatric rheumatologists. However, it is needed that more LA countries join the survey in order to achieve a more reliable outcome of the LA quality of care. In the future, the aim of SHARE LA is to expand the families survey to a larger number of LA countries, and compare these data to those of the European countries.

**Disclosure of Interest**

None Declared.

**Table 24 (Abstract P263).** See text for description

SHARE LA - First participant countries	
Brazil	400
Chile	80
Argentina	38
El Salvador	17
Total = 535	

### P264 Share Latin America – Brazilians’s families view on standard of care for children with juvenile idiopathic arthritis

#### Ingrid H. Rotstein Grein^1,2^, Marcia Bandeira^1^, Claudia S. Magalhães^3^, Luciana G. Portasio^3^, Claudio A. Len^4^, Maria Teresa R. A. Terreri^4^, Sheila K. F. D. Oliveira^5^, Luciana B. P. Marques^6^, Gecilmara Pillegi^7^, Simone Appenzeller^8^, Flavio R. Sztajnbok^9^, Teresa Cristina M. V. Robazzi^10^, Ana Julia P. D. Moraes^11^, Melissa M. Fraga^12^, Aline Islabão^13^, Nico Wulffraat^2^

##### ^1^Pediatric Rheumatology, Hospital Infantil Pequeno Príncipe, Curitiba, Brazil; ^2^Pediatric Rheumatology, Wilhelmina Children’s Hospital/University Medical Centre Utrecht, Utrecht, Netherlands; ^3^Pediatric Rheumatology, Universidade Estadual Paulista/UNESP, Botucatu, Brazil; ^4^Pediatric Rheumatology, Universidade Federal de São Paulo/UNIFESP, São Paulo, Brazil; ^5^Pediatric Rheumatology, Instituto de Puericultura e Pediatria Martagão Gesteira/UFRJ, Rio de Janeiro, Brazil; ^6^Pediatric Rheumatology, Hospital Infantil Albert Sabin, Fortaleza, Brazil; ^7^Pediatric Rheumatology, Hospital das Clínicas da FMRP/USP, Ribeirão Preto, Brazil; ^8^Pediatric Rheumatology, Faculdade de Ciências Médicas da Unicamp, Campinas, Brazil; ^9^Pediatric Rheumatology, Universidade Estadual do Rio de Janeiro/UERJ, Rio de Janeiro, Brazil; ^10^Pediatric Rheumatology, Hospital Universitário Prof. Edgard Santos/UFBA, Salvador, Brazil; ^11^Pediatric Rheumatology, Universidade Federal do Pará, Belém, Brazil; ^12^Pediatric Rheumatology, Hospital Infantil Darcy Vargas, São Paulo, Brazil; ^13^Pediatric Rheumatology, Hospital da Criança de Brasília José Alencar, Brasília, Brazil

###### **Correspondence:** Ingrid H. Rotstein Grein

**Introduction:** Since 2013 PRES has initiated an European project called SHARE (Single Hub and Access point for Paediatric Rheumatology in Europe, PI Wulffraat). The project aimed to identify the specific needs for optimal care in pediatric rheumatic diseases (PRD) in each European country. Since the SHARE project have showed to be an important tool for examining the health care standards in Europe, the idea of expanding the project to another continents gave place to the SHARE Latin America (LA) project in 2016. This new project focused on families view on standard of care for children with Juvenile Idiopathic Arthritis (JIA) in LA countries. The first LA country to join this initiative was Brazil.

**Objectives:** To identify which health care aspects needs improvement in order to plan further optimal care to children with JIA in different regions of Brazil.

**Methods:** Families surveys have been performed at pediatric rheumatology centers of Brazilian’s different regions. Families of patients with diagnosis of JIA according ILAR (International League of Associations for Rheumatology) criteria answered a single time-point survey that encompasses information about specialists referral patterns and referral delays, disease diagnosis, multidisciplinary health care team follow up, access to treatment, participation in clinical research and transition of care to the adult service. Families filled in the questionnaires anonymously on paper or on PRINTO (Pediatric Rheumatology INternational Trials Organisation) website.

**Results:** Until May 2017, 400 families from Brazil answered the questionnaires. The Table 25 shows the participant centers and the number of answered surveys of each one of them. Twelve rheumatology centers from seven states of the country participated in the survey. After the first appointment with the pediatric rheumatologist, families view showed regular follow up, with good access to rheumatic medications and satisfactory uveitis screening. However, some families had to wait a long time for the first appointment with the specialist, and also had to travel very long distances to the rheumatology center.

**Conclusion:** The difference in the quantity of answered questionnaires among Brazilian regions reflects the distribution of rheumatology centers in the country. It is well known that there are extensive areas of the country that don’t have a rheumatology center nearby. For this reason, it’s difficult to the patient to reach the specialist in the beginning of the disease in some areas. Once the patients have reached the rheumatology care, they had a satisfactory follow up with the specialist.

**Disclosure of Interest**

None Declared.

**Table 25 (Abstract P264).** See text for description

Rheumatology centers by Brazilian regions (n = 400)			
North		Southeast	
Pará - UFP	25	São Paulo - UNIFESP	50
Northeast		São Paulo - HIDV	15
Fortaleza - HIAS	47	Botucatu - UNESP	27
Bahia - UFBA	22	Campinas - UNICAMP	30
West Central		Ribeirão Preto - USP	30
Brasília - ICB	15	Rio de Janeiro - IPPMG	50
South		Rio de Janeiro - UERJ	29
Curitiba - HPP	60		

### P265 Safety of adalimumab ± methotrexate for the treatment of polyarticular juvenile idiopathic arthritis (PJIA): strive registry

**Abstract withdrawn**

### P266 Anti-citrullinated protein/peptide antibodies (ACPA) in juvenile idiopathic arthritis - a Swedish cohort

#### Raya Saleh^1^, Erik Sundberg^2^, Monika Hansson^1^, Bo Magnusson^2^, Leonid Padyukov^1^, Helena Erlandsson Harris^1^

##### ^1^Rheumatology Unit, Department Women and Children’s health, Karolinska Institute, Solna, Sweden; ^2^Pediatric rheumatology Unit, Department Women and Children’s health, Karolinska Institute, Solna, Sweden

###### **Correspondence:** Raya Saleh

**Introduction:** Anti-cyclic citrullinated peptide (anti-CCP) antibodies have a diagnostic value for RA, with a specificity of 96-100% and a sensitivity of 40-85%. The presence of anti-CCP Abs is also included in the revised ACR 2010 criteria for RA. However, the presence of anti-CCP Abs in JIA and their diagnostic value is not as well described.

**Objectives:** To investigate the presence of anti-CCP antibodies in a cohort of JIA patients collected in the Stockholm area, Sweden (the JABBA cohort). Furthermore, to define the ACPAs presence and to correlate their occurrence to clinical parameters recorded in the Swedish juvenile arthritis quality register.

**Methods:** Detection of anti-cyclic citrullinated peptide antibodies was performed for 352 JIA patients and 66 age and sex matched healthy controls using anti-CCP2 ELISA (Euro-Diagnostica). ACPAs were analysed in 81 patients with CCP2 reactivity ranging from ≥24AU/ml (n = 11), 24 > CCP2 ≥ 14 AU/ml (n = 7), 14 > CCP2 ≥ 10 AU/ml (n = 8) and CCP2 < 10 AU/ml (n = 55). Additionally, 191 healthy controls were analysed for the presence of ACPAs. For ACPA analysis, a multiplex analytic microarray system (Phadia AB, Uppsala, Sweden) that analyzes simultaneous occurrence of antibodies to 18 different citrullinated peptides including filaggrin (CCP-1/Fil307-324), vimentin (Vim2-17, Vim60-75), fibrinogen α573 (Fibα573), Fibα591, Fibβ72, Fibβ74, Fibβ36–52, Citrullinated enolase peptide 1 (CEP-/α-Enolase) and CPP3 (Porphromonas gingivalis peptidylarginine deaminase) was used.

**Results:** 22 patients (6%) and 2 healthy controls (3%) had ≥24 AU/ml recorded in the anti-CCP2 assay, being significantly higher in patients (P < 0.0001). The ACPA micro array analysis defined 27 out of 81 JIA patients positive to at least one citrullinated peptide. 10 patients (12%) were positive to Vim60-75, CEP-1 and CPP3. Most ACPA reactivities were evident in the CCP2 ≥ 24 AU/ml group with highest reactivities against CCP1, Vim60-75, CCP3, Vim 60-75, Fib Alpha36-50 and Fib beta60-74. CCP2 antibodies were mainly positive in female patients and the levels were significantly higher in them compared to males (P < 0.0032). Levels did not correlate with age in patients group (P = 0,4659) or in healthy controls (P = 0,8900). Anti-CCP2 positive JIA patients have increased number of ACPAs with different fine specificities (p < 0.001).

**Conclusion:** Anti-CCP2 positive JIA patients display a greater number of ACPAs with different fine specificities. More studies need to be done in order to investigate the diagnostic or prognostic value of defining ACPA fine specificities in JIA patients.

**Disclosure of Interest**

None Declared.

### P267 Incidence of juvenile idiopathic arthritis in Finland, 2000-2013

#### Sirja Sard, Tytti Pokka, Paula Vähäsalo

##### Department of Children and Adolescents, Medical Research Center Oulu, Oulu University Hospital, PEDEGO Research Unit, University of Oulu, Oulu, Finland

###### **Correspondence:** Sirja Sard

**Introduction:** There is a worldwide variation in the incidence of juvenile idiopathic arthritis (JIA) which may be affected by genetic and environmental factors. The incidence of autoimmune diseases like type I diabetes or inflammatory bowel disease has increased during the last decades in Finland and all over the world.

**Objectives:** The aim of this study was to assess the annual incidence of JIA in Finland. We also aimed to study trends in the incidence of JIA in Finland.

**Methods:** We identified children <16 years with ≥1 relevant diagnosis code of M08.0, M08.1, M08.2, M08.3, M08.4, M07 or M09.0 (International Classification of Diseases, 10th edition) in computerized Care Register for Health Care maintained by National Institute for Health and Welfare during 2000-2013. Nationally all inpatient and specialised outpatient visits are registered in this register. The diagnosis had to occur at least in two separate visits of the patients in a hospital or in an outpatient clinic. In Northern Ostrobothnia hospital district we reviewed the medical records of all the patients to confirm the diagnosis of JIA.

**Results:** A total of 4597 new JIA cases were identified during 2000-2013. The annual incidence rate was 31,8 per 100 000 children/year of the population <16 years of age for the whole study area varying from 28,0 in 2007 to 36,3 in 2012. In Northern Ostrobothnia hospital district the incidence rate was 34,4 per 100 000 children/year according to care register for health care and 29,3 per 100 000 children/year according to medical records during 2000-2013.

**Conclusion:** This is the first nation wide study on the epidemiology of JIA in Finland. Incidence rates are found higher than those reported earlier in parts of Finland and in Nordic countries.

**Disclosure of Interest**

None Declared.

### P268 Developing, evaluating and implementing smartphone applications for young people living with rheumatic and musculoskeletal diseases: a scoping review of empirical research

#### Simon R. Stones

##### School of Healthcare, University of Leeds, Leeds, UK

**Introduction:** Childhood-onset rheumatic and musculoskeletal diseases (RMDs) can influence multiple aspects of a young person’s life. The journey towards adulthood spans several years, where young people develop a set of tools to cope with new situations. Self-management is an integral part of this process, and smartphone applications (apps) represent an innovative and efficient method of promoting self- and shared-management of RMDs.

**Objectives:** This scoping review aims to understand how existing smartphone apps have been developed and evaluated for young people with RMDs.

**Methods:** Eligible studies published between 2000 and 2017 were identified through a comprehensive search of four bibliographic databases. The search strategy included MeSH subject headings and free-text search terms relating to: young people, digital technology and RMDs. The quality of included studies were assessed using the Mixed Methods Appraisal Tool.

**Results:** The combined bibliographic searches identified 576 articles. Once duplicates were removed, 441 titles and abstracts were were screened, 19 of which were eligible for a full-text review. Following full-text review, 11 articles were suitable for inclusion. Of these, three articles were peer-reviewed journal articles, while eight were conference proceedings. Out of 11 included articles, eight studies were described: six involving juvenile idiopathic arthritis (JIA) and two involving systemic lupus erythematosus (SLE). All eight studies focused on educating young people with RMDs about their conditions. Three studies had developed apps to record and communicate pain experiences associated with JIA, while others focused on wider self-management skills. The heterogeneity in the design, evaluation and delivery of apps was noticeable, with clear similarities in the needs of young people with different RMDs. Common findings from all studies included the need to develop apps across multiple platforms and devices which do not require wireless or cellular data to be functional. There were, however, some shared styles to developing apps among the included studies. Some method of user testing and iterative cycle of designing and refining the apps was observed. The Assessment, Design, Development, Implementation and Evaluation (ADDIE) model was used in one study, while four studies used an iterative process of designing and refining, based on the hermeneutical circle concept. A collaborative concept-generating and requirements- gathering methodology was used in another study, while the Medical Research Council’s framework for the development and evaluation of complex interventions was used in another.

**Conclusion:** This scoping review highlights that there is little empirical evidence published which describes the development of smartphone apps for young people with RMDs. Of the available evidence, approaches appear fragmented and pragmatic, suggesting the need for standardisation to facilitate the convoluted and demanding processes required to develop apps.

**Disclosure of Interest**

None Declared.

### P269 T cell response after varicella vaccination in children with JIA treated with biologic therapy

#### Natasa Toplak^1,2^, Andreja N. Kopitar^3^, Alojz Ihan^3^, Tadej Avcin^1,2^

##### ^1^Department of Allergology, Rheumatology and Clinical Immunology, University Children’s Hospital, University Medical Centre Ljubljana, Ljubljana, Slovenia; ^2^Faculty of Medicine, University of Ljubljana, Ljubljana, Slovenia; ^3^Institute of Microbiology and Immunology, Faculty of Medicine, University of Ljubljana, Slovenia, Ljubljana, Slovenia

###### **Correspondence:** Natasa Toplak

**Introduction:** Varicella infection is one of the most dangerous infections in children treated with immunomodulatory drugs, including biologics. Varicella vaccine is a live attenuated vaccine and as such not recommended in children treated with biologic therapy except in a case to case basis when the risk of infection is greater that the risk of vaccination.

**Objectives:** To evaluate T cell response after varicella vaccination in children with juvenile idiopathic arthritis (JIA) treated with biologics and to determine the presence of T cell immune response after the disappearance of protective antibodies (pAb).

**Methods:** We performed a cross-sectional study in children with JIA treated with biologic therapy who were vaccinated with attenuated live varicella vaccine. The majority of children were tretaed also with methotrexate. pAb were periodically assessed during a long term follow-up and T cell response was determined at the last clinic visit. T cell response was tested on PBMCs stimulated with Varicella-Zoster virus (strain Oka vaccine HHV-3) from Innovative Peptide Solutions at the final concentration 1μg/ml. Unstimulated cells were used as negative control and 10 μg/mL of SEB (Sigma) served as positive control. Among the CD3 + CD4 and CD3 + CD8+ T lymphocytes, the activated CD69+ cells were analysed for intracellular production of IFN-γ. Ethic committee approved the study, parents of vaccinated children signed informed consent for vaccination.

**Results:** By May 2017 varicella vaccine was given to 11 children with JIA (median age at the time of the first vaccination 4, range 2,5-11 years) treated with biologic therapy (6 etanercept, 3 tocilizumab, 1 adalimumab and 1 infliximab). All except one patient already received at least 2 doses of varicella vaccine, and the last enrolled patient who was recently vaccinated with the first dose is scheduled to receive the next dose in 6 weeks’ time. The latter child was excluded from further evaluations. Three patients received the third, booster varicella vaccination because the pAb decreased below protection level during a long term follow up. There were no adverse effects of vaccination and none of the vaccinated children got varicella infection in a time span of three months after vaccination. Two children got varicella infection later; one without pAb 5 years after the second dose and one with low pAb values 4 months after the second dose. In both cases the course of varicella infection was mild. pAb were tested 5-10 weeks after the second dose of vaccine. Protection was achieved in 8/10 vaccinated children. T cell response was tested after the last dose of vaccine (median 11 months, range 3-64 mo). Two children who got varicella infection were not included. At the time of T cell response determination 4/7 children were still having pAb, among them 2 were also having T cell response. Within the group of 3/7 patients without detectable pAb, 2 patients had positive T cell immune response.

**Conclusion:** Varicella vaccination appears to be safe in JIA patients treated with biologics, but the production of pAb and T cell response appears to be heterogeneous and vaccination does not always provide protection against varicella infection.

**Disclosure of Interest**

None Declared.

### P270 2D shear wave elastography to reveal methotrexate-induced hepatic fibrosis in patients with juvenile idiopathic arthritis

#### Maria Tsinti^1^, Vasiliki Dermentzoglou^2^, Elena Tsitsami^3^

##### ^1^Pediatric Rheumatology Unit, First Department of Pediatrics, University of Athens, Medical School, Children’s Hospital “Aghia Sofia”, Athens, Greece; ^2^Department of Radiology, Children’s Hospital “Aghia Sofia”, Athens, Greece; ^3^Pediatric Rheumatology Unit, First Department of Pediatrics, University of Athens, Medical School Children’s Hospital “Aghia Sofia”, Athens, Greece

###### **Correspondence:** Maria Tsinti

**Introduction:** Transient transaminase elevations are frequently observed in patients with juvenile idiopathic arthritis (JIA) receiving long-term methotrexate (MTX) treatment, occasionally followed by changes in liver histology. Despite that MTX-induced hepatic alterations are considered to be reversible, their significance remains as yet unclear.

**Objectives:** To evaluate the ability of 2D Shear Wave Elastography (2DSWE) to uncover residual hepatic changes induced by the long-term treatment with MTX in JIA patients.

**Methods:** Thirty JIA patients treated MTX longer than 2 years, 25 girls and 5 boys aged 2,8-17,2 years (median 8,6), 21 with oligoarthritis, 8 with polyarthritis, 1 with enthesitis related arthritis were enrolled in the study. Liver function tests had been performed bimonthly to all of them since the beginning of MTX treatment. At the time of the study all patients had normal platelet count, prothrombin time, and transaminases, albumin and bilirubin levels while none of them had evidence of non-alcoholic steatohepatitis. Ninety-two age- and sex-matched healthy children were examined as controls. Complete evaluation of the liver was performed by B mode and Doppler US followed by 2DSWE. At least three consecutive different stiffness color maps were obtained of both liver lobes with four regions of interest selected resulting in 12 liver stiffness measurements for each child. Final 2DSWE value (kPa) represents the mean value of measurements (MEV). MEV values in healthy children were independent of sex, age and body mass index, and values over 5 KPa were defined as abnormal. Pearson’s correlation or independent t-test by the use of SPSS were used to evaluate changes in MEV values in relation with total MTX dose, treatment duration, anti-TNFa coadministration, occurrence of transaminasemia events (2x the upper limit of normal).

**Results:** Normal MEV was found in 27/30 JIA patients (mean 4,33 Kpa) while 2/30 presented with normal upper limits (4,99 and 5,01), and 1/30 with mildly elevated values (5,45 Kpa). Although not with maximum MTX cumulative dose or prolonged administration, the last three patients had a history of several transaminasemia events (2-8). However, on re-examination, seven months later, elasticity values of the last case remained mildly elevated (5,33 Kpa) while those of the other two were found within normal limits (4,48 and 4,67, respectively). Amongst the remaining 27 patients with normal 2DSWE values, only one had a history of transaminasemia events (n = 8). No correlation was found between MEV and total MTX dose (median 1733 mg, range 392-7301; p = 0,365), or treatment duration (median 27,5 months, range 12-114; p = 0,357). Finally, no difference in elasticity was detected between patients treated with MTX alone and those with MTX plus anti-TNFa (p = 0,675).

**Conclusion:** We provide preliminary evidence that 2DSWE could be used as a non-invasive tool for the detection of hepatotoxicity in JIA patients receiving MTX for a long time. Liver changes detected by 2DSWE seem to be independent of the cumulative MTX dose, the duration of its administration or the number of transaminasemia events. Provided evidence is worthy to be further evaluated.

**Disclosure of Interest**

None Declared.

### P271 Efficacy and tolerability of switching from subcutaneous to oral route of methotrexate administration in juvenile idiopathic arthritis

#### Maria Tsinti^1^, Vasiliki Dermentzoglou^2^, Elena Tsitsami^3^

##### ^1^Pediatric Rheumatology Clinic, First Department of Pediatrics, University of Athens, Medical School, Children’s Hospital “Aghia Sofia”, Athens, Greece; ^2^Department of Radiology, Children’s Hospital “Aghia Sofia”, Athens, Greece; ^3^Pediatric Rheumatology Unit, First Department of Pediatrics, University of Athens, Medical School, Children’s Hospital “Aghia Sofia”, Athens, Greece

###### **Correspondence:** Maria Tsinti

**Introduction:** Methotrexate (MTX) is the cornerstone disease-modifying anti-rheumatic drug in juvenile idiopathic arthritis (JIA). Existing evidence concerning MTX tolerance between oral and subcutaneous MTX in JIA patients is contradictory. Moreover, there is some evidence suggesting different MTX bioavailability and efficacy after oral and subcutaneous administration.

**Objectives:** To evaluate MTX efficacy and tolerability after switching from the subcutaneous (SC) to the oral (PO) route of administration in children with JIA.

**Methods:** We switched the route of MTX-administration from SC to PO in 27 randomly selected JIA patients with clinically inactive disease for >1 year (median 5 years, range 1-5.1) under MTX monotherapy (15 mg/m^2^). Four boys and 23 girls, median age 10,4 years (range 3.4-18.2), median disease duration 4.6 years (range 2-16); 6 with polyarthritis, 18 with oligoarthritis, and 3 with extended oligoarthritis, 15 were presenting with positive ANA. Wallace Criteria and power Doppler ultrasound examination were applied to define inactive disease. Fourteen of 27 patients had 1-3 disease flares during a previous 5-year follow-up period while the median time on clinically inactive disease under SC-MTX was 2 years (range 0,5-3,8). Following the switching of the route of MTX-administration, patients were followed-up every 3 months for at least two years.

**Results:** Switching from SC to PO route of MTX-administration was effective in 26/27 patients (96%). Only one patient returned to SC-MTX due to the appearance of a disease flare 7 months after switching. One more 13 year-old female patient returned to SC-MTX after 1 month PO-MTX due to worsening of nausea. With regard to tolerability of switching, while on SC-MTX, seventeen of the remaining 25 patients (68%) were presenting with persistent gastrointestinal symptoms, dizziness and/or behavioral symptoms occurring before (anticipatory/associative) MTX-administration. In 15/17 (88%) of patients these complaints subsided completely after switching whereas in 2/19 (12%) the complaints improved but did not disappear.

**Conclusion:** Our results indicate that in JIA patients on longstanding clinically inactive disease under monotherapy with SC-MTX, switching from SC to PO route of MTX-administration is effective in the vast majority of patients. Additionally, due to the high degree of tolerability after PO administration switching to PO route seems to be easily achievable with concomitant improvement of patients’ and families’ dissatisfaction and subjective complaints due to injections (not measured with a validated tool in this study). According to our previous findings, MTX withdrawal in JIA patients in longstanding remission is accompanied by a 50% relapse rate. In conclusion, switching from SC to PO route of Methotrexate administration offers a safe treatment modality to reassure the continuation of disease remission with an acceptable improvement of the patients’ quality of life.

**Disclosure of Interest**

None Declared.

### P272 Many pediatric psoriatic arthritis patients are associated with fibromyalgia syndrome compared to other subtypes of JIA

#### Ken-Ichi Yamaguchi, Tokutaroh Tsuda, Mitsumasa Kishimoto, Masato Okada 

##### Immuno-Rheumatology Center, St.Luke’s International Hospital, Tokyo, Japan

###### **Correspondence:** Ken-Ichi Yamaguchi

**Introduction:** Pediatric patients with psoriatic arthritis (PsA) or enthesitis related arthritis (ERA), which are subtype of Juvenile Idiopathic Arthritis (JIA), had been rare in Japan. But the number of patients with these diseases is increasing in recent years.

**Objectives:** We aimed to clarify the clinical picture of patients with PsA compared to other subtypes of JIA.

**Methods:** We examined clinical symptoms, examination results and treatment retrospectively using electronic medical charts for JIA patients treated in our center. To diagnose JIA and subtypes, we used the ILAR Criteria, Assessment in Spondyloarthritis International Society (ASAS) Classification Criteria, the Classification of Psoriatic Arthritis (CASPAR) Criteria and Vancouver Criteria.

**Results:** Of 48 JIA cases, we had 7 cases (15%) of PsA, 9 cases (19%) of ERA, 5 cases (10%) of systemic, 8 cases (17%) of oligo, 8 cases (19%) of rheumatoid factor (RF) negative polyarthritis and 9 cases (19%) of RF positive polyarthritis. The ratio of PsA and ERA were higher than previous study about Japanese JIA patients. 3 cases (6%) were undifferentiated arthritis. 71% of PsA patients were associated with fibromyalgia syndrome, while other subtype group patients were 33%, 0%, 0%, 33%, 25% respectively. We took longer time to make diagnose of PsA and ERA cases than other subtype patients.

**Conclusion:** Pediatric PsA patients were increasing in Japanese JIA. Many of PsA patients are associated with fibromyalgia syndrome compared to other subtypes of JIA.

**Disclosure of Interest**

None Declared.

## Juvenile dermatomyositis - Scleroderma

### P273 New insights in cardiac involvement of juvenile systemic sclerosis patients: a 3D echocardiographic assesment unveils subclinical dysfunction of ventricules

#### Reyhan Dedeoglu^1^, Amra Adrovic^2^, Sezgin Sahin^2^, Kenan Barut^2^, Funda Oztunc^1^, Ozgur Kasapcopur^2^

##### ^1^Pediatric Cardiology, Istanbul University, Cerrahpasa Medical School, Istanbul, Turkey; ^2^Pediatric Rheumatology, Istanbul University, Cerrahpasa Medical School, Istanbul, Turkey

###### **Correspondence:** Amra Adrovic

**Introduction:** Juvenile systemic sclerosis (JSS) is a rare disease with possible vital organ involvement and severe complications. Cardiac involvement in particular tends to result in severe clinical complications, such as congestive heart failure, arrhythmias, and sudden cardiac death. Cardiac manifestations that often begin during the early stages of the disease but remain clinically asymptomatic at onset. Therefore, the identification of high-risk patients would benefit from early medical intervention for cardiac complications. New echocardiography modalities, like two- and three-dimensional (2D, 3D) speckle tracking (STE) employing strain analysis, provide an assessment of myocardial deformation in the early stage of the disease.

**Objectives:** We aimed to use the (2D with 3D) STE for the detection of subclinical cardiac involvement of the ventricles in patients with JSS. Therefore, the identification of high-risk patients would benefit from early medical intervention for cardiac complications.

**Methods:** We assessed patients with JSS together with age- and sex-matched controls. All the target patients met the Pediatric Rheumatology European Society/American College of Rheumatology/European League against Rheumatism provisional classification criteria for JSS. A complete medical history was taken and physical examination and laboratory evaluation performed for each patient at the time of enrollment. All images were acquired with a Philips iE33 echocardiographic system (Philips Medical Systems, Andover, MA, USA), using an X3-1 matrix-array transducer. According to the international recommendations, 3D left and right ventricle (LV, RV) full volume sweeps were acquired from four views, during an end-expiratory breath-hold when possible: the apical view, the sagittal (to outline the tricuspid valve), the four-chamber (to outline the apex), and the coronal (to outline the RV outflow tract).

**Results:** We assessed 21 patients with JSS and 19 healthy controls. The mean ages of the JSS patients and healthy controls at the time of study were 15.38 ± 2.74 and 14.33 ± 3.48 years, respectively. The mean disease duration was 2.65 ± 2.6 years (range: 0.6 –10 years). The left ventricular end diastolic volume (LVEDV), end systolic volume (LVESV) and ejection fraction (EF) were different between patients and control group (99.2 ± 23.8 vs. 52 ± 23.8, 40.6 ± 16.0 vs 20.2 ± 17.4, 59.2 ± 7.5 vs 65.6 ± 5.2). Global longitiduinal strain (GLS), Global circumferential strain (GCS) were lower in patients (18.4 ± 4.7 vs 22.4 ± 3.7 and 26.4 ± 5.8 vs 31.4 ± 3.5), peak systolic strain values of right ventricular septal (RVLS septal) and free wall (RVLS freewall) were lower in patients with JSS (18.1 ± 6.8 vs 24.8 ± 6.0, 22.8 ± 5.9 vs 28.0 ± 6.9). 3D measurements of RVEDV, RVESV, RVSV (88.2 ± 31,3 vs 50.8 ± 23.5, 43.1 ± 17.6 vs 19.0 ± 12,2 and 45.0 ± 16.2 vs 31,7 ± 12,6) were higher in patients with JSS. RVLS freewall were lower in JSS patients having interstitial lung fibrosis, arthritis, muscle weakness, weight loss, high level of anti-Scl 70 antibodies than the JSS patients without these variables. We found that a GCS < 34.5% could identify patients for LV dysfunction with a sensitivity of 93.3, specificity of 92.9 and RVEF < 60.7% could identify patients for RV dysfunction with a sensitivity of 92.9 and specificity of 21.4%.

**Conclusion:** Currently, there is a lack of data on the presence and development of functional myocardial dysfunctions in JSS patients. Our results highlight key advantages of 3D STE for the tracking of early systolic dysfunction in patients with JSS, which may be useful in the clinical setting. More aggressive immunosuppressive treatment may be recommended to prevent progression of myocardial dysfunction, as shown with new echocardiography modalities like 2D and 3D STE.

**Disclosure of Interest**

None Declared.

### P274 The relationship between hand grip strength, pain level and hand functionality in children with juvenile scleroderma

#### Arzu Dag^1^, Ela Tarakci^2^, Amra Adrovic^3^, Ozgur Kasapcopur^3^

##### ^1^Faculty of Health Sciences, Division of Physiotherapy and Rehabilitaiton, Istanbul Yeni Yuzyil University, İstanbul, Turkey; ^2^Physiotherapy and Rehabilitation, Istanbul University, Faculty of Health Science, İstanbul, Turkey; ^3^Department of Pediatric Rheumatology, Istanbul University Medical Faculty of Cerrahpasa, İstanbul, Turkey

###### **Correspondence:** Arzu Dag

**Introduction:** Juvenile scleroderma (JS) is a group of rare and complex autoimmune diseases characterized by microvascular injury and excessive fibrosis of the skin and internal organs and induration, the localized form (JLS) of which is related only to the skin, while the systemic type (JSS) is connected with of the other organs. The most striking clinical findings of the diseases is skin thickening, hardening and sclerosis. Although there is considerable individual variation, skin involvement tends to reach a maximum within the first 3 years, after which the skin becomes thinner, although complete remission in the fingers and hands is less common. Skin thickening is a manifest consequence of JS. The hands account for only a small fraction of the total skin area of the body, but are necessary for many important functions in daily life.

**Objectives:** The aim of this study was to assess the relationship between hand grip strength, pain level and hand functionality in children with JS.

**Methods:** 28 patients (24 girls, 4 boys) attending to the Istanbul University Cerrahpasa Faculty of Medicine, Department of Pediatrics, Division of Pediatric Rheumatology, Istanbul, Turkey between February 2016 and May 2017 were included in this study. Cross-sectional study design including 28 patients with JS, 12 patients the systemic type, while 16 patients localized form, was used. Sociodemographic data and clinical features were assessed. Hand-key-palmar and tip grip strength and were obtained by Jamar hydrolic hand dynamometry and pinchmeter. Children rated their pain severity on a six-item Wong-Baker FACES® Pain Rating Scale (WBS) from none to worst. Hand functional ability assessed with Duruoz Hand Index (DHI).

**Results:** The mean ages of patients were 14.75 ±   3.22 years for JSS and 13.81  ± 3.39 years for JLS respectively.  Fifteen (53.4%) out of 28 patients had hand involvement and this was indicated as pain, limitation of movement and presence of loss of upper extremity muscle strength. Four (25%) out of 16 patients with JLS form patients had hand involvement, while eleven (91.66%) out of 12 patients with JSS type had hand involvement (Fig 1).The rates of children with JS feeling pain at rest and at the time of activity were 3,6% and 47,4%, respectively. Pearson’s correlation analysis showed the positive correlation relationship between the hand functionality and pain level values (p = 0.01, r = 0,25). There was no positive correlation relationship between hand functionality and hand-key-palmar and tip grip strength (Grip Strenght Right hand p = 0.88 r = -0,30, Grip Strenght Left Hand p = 0.71 r = 0,73, Hand key grip p = 0.20 r = 0,24) (Table 26).

**Conclusion:** In this study, it is demonstrated that increases in hand functionality values are especially directly related with pain level rather than hand-key-palmar and tip grip strength. Pain has been determined as the most influential factor in the daily use of the hand. Therefore it was determined that pain is the fist symptom to be treated using hand effectively in daily life activities in children with JS, with espeailly in JSS.

**Disclosure of Interest**

None Declared.

**Table 26 (Abstract P274).** The relationship between hand grip strength, pain level and hand functionaliy

	Pain	Hand Grip Strenght	Hand Key Grip
Duruoz Hand Index	r = 0.25 p = 0.01	right hand, r = -0.30 p = 0.88	right hand, r = 0.24 p = 0.20
		left hand, r = -0.73 p = 0.71	left hand, r = 0.17 p = 0.36

### P275 Long term outcome of juvenile localized scleroderma: data from a single pediatric rheumatology center

#### Gloria Fadanelli, Anna Agazzi, Fabio Vittadello, Francesco Zulian, Giorgia Martini

##### Pediatric Rheumatology Unit, Department of Woman and Child Health, University Hospital of Padua, Italy, Padua, Italy

###### **Correspondence:** Gloria Fadanelli

**Introduction:** Juvenile Localized Scleroderma (JLS) comprises a group of autoimmune fibrosing conditions involving skin and subcutaneous tissues after initial inflammatory reaction. The treatment of JLS is challenging for pediatric rheumatologists and, to date, available data on long-term outcome are partial and incomplete.

**Objectives:** to evaluate the long-term outcome of patients with JLS based on clinical form, type and time of treatment and to identify possible predictors of outcome, that can guide the treatment choice at diagnosis.

**Methods:** a retrospective study with collection of clinical, radiological and laboratory data from patients with JLS, followed at the Paediatric Rheumatology Unit of the University Hospital of Padua has been performed. We included all patients that had at least 6 months follow-up and had been evaluated at least once in the last 2 years. For each patient, clinical assessment of the disease activity and of severity of tissue damage and functional impairment was performed by using the LoScat score combined with thermography. Univariate tests and a multivariate model were used for statistical analysis.

**Results:** 133 patients entered the study: 84.6% of patients had a partial remission of disease (inactive disease on treatment) within the first year of follow-up, 55.6% had a complete remission (inactive disease and more than 2 years without any treatment) within 5 years of follow-up and 85.7% within 7 years of follow-up; 12.5% has a still active disease after over 10 years of follow-up and all them have the linear subtype. 59.5% of patients had no relapses in the disease course, 22.2% had at least one relapse on average 20 months after discontinuation of treatment, and 12.7% presented repeated relapses. Overall, 18.3% of patients did not respond to topical or systemic therapy. 49.2% of all patients developed only a mild tissue damage, 25.4% moderate and 23% reported a severe residual skin involvement. Only 2.4% patients have no aesthetic sequelae. Up to 19.8% present a functional limitation and, in particular, linear scleroderma and pansclerotic morphea are those associated with more severe aesthetic and functional sequelae. The entity of tissue and functional damage does not change over time and is not related to the duration of follow-up: 27.8% of patients with <2 years of follow-up has a marked damage, 20.6% in those with a follow-up of 5-10 years and 25% with a follow-up > 10 years. A delay in systemic treatment is associated with a worse outcome in terms of activity duration and number of disease relapses.

**Conclusion:** The majority of patients with JLS achieves complete remission and do not present disease relapses. Patients with the linear subtype have more aggressive disease course with active disease after more than 10 years and more disease relapses as compared to other forms. The entity of skin and subcutaneous tissue damage seems to establish at the early stages of the disease and to remain stable over time. A delay in the start of treatment is related to a worse outcome. A close follow-up is mandatory in particular during the first 2 years after discontinuation of treatment when disease relapses occur more frequently.

**Disclosure of Interest**

None Declared.

### P276 Update on the juvenile systemic sclerosis inception cohort project. characteristics of the first 97 patients at first assessment. www.juvenile-scleroderma.com

**Abstract withdrawn**

### P277 Systemic capillary leak syndrome in juvenile dermatomyositis

#### Alessandra Meneghel^1^, Giorgia Martini^1^, Carolina Birolo^1^, Alvise Tosoni^2^, Andrea Pettenazzo^2^, Francesco Zulian^1^

##### ^1^Department of Woman and Child Healt, University Hospital of Padua, Pediatric Rheumatology Unit, Padova, Italy; ^2^Department of Woman and Child Healt, University Hospital of Padua, Pediatric Intensive Care Unit, Padova, Italy

###### **Correspondence:** Alessandra Meneghel

**Introduction:** Juvenile dermatomyositis (JDM) is a rare disease characterized by chronic inflammation of muscle and skin [1]. Systemic capillary leak syndrome (SCLS) is a rare life-threatening condition, characterized by sudden onset of hypotension, hypoalbuminemia and hemoconcentration due to vascular endothelium alterations that cause leakage of plasma and protein into the interstitial space leading, in some cases, to hypovolemic shock and death [2]. Plasma leakage into muscle and fascia can cause increased intra compartmental pressure inducing rhabdomyolysis and compartment syndrome [3].

**Objectives:** We describe three cases of JDM complicated by SCLS requiring intensive care and aggressive immunosuppressive treatment.

**Methods:** Data on clinical and laboratory parameters were collected from three patients evaluated at our Pediatric Rheumatology Unit in the last five years.

**Results:** All the patients were male, aged between 3 and 13 years-old, diagnosed with JDM according to Bohan and Peter criteria [2]. All the patients presented with acute onset of unexplained edema associated with hypotension, myalgias with rhabdomyolisis, neutrophilic leukocytosis, hyponatriemia, hypoalbuminemia and hemoconcentration (Table 27). They developed SCLS while on high dose MPDN therapy. Two of these patients (case 1 and 3) needed pediatric intensive care support. One patient developed an acute compartment syndrome of the upper extremities that needed emergency fasciotomy. All patients had an optimal response to intravenous immunoglobulin therapy (IGIV) with complete recovery of the SCLS.

**Conclusion:** SCLS is a rare but life-threatening condition calling for early detection and treatment. The pathogenesis of this disorder seems to be related to abnormalities in vascular endothelial growth factor (VEGF), endothelial cell apoptosis and increased expression of pro-inflammatory molecules [4]. More recently SCLS has been reported in association with autoimmune disease [5]. A confirmation of the possible immunologic pathogenesis of this disorder is the reported efficacy of the IVIG treatment which recently revealed to be a promising therapy for SCLS [6]. Our patients with JDM developed SCLS while on high dose MPDN therapy, confirming that corticosteroids cannot prevent this complication that should be suspected in a JDM patient with an atypical course characterized by acute unexplained onset of edema and myalgias with rhabdomyolysis, neutrophilic leukocytosis, hypoalbuminemia, hemoconcentration and hypotension. In these cases, intensive symptomatic treatment and prompt intravenous immunoglobulin (IVIG) administration are mandatory.

**Informed consent**

The subject’s and parents’ written consent was obtained according to the Declaration of Helsinki.

**References**

1. Rider LG, Lindsley CB, Miller FW. Juvenile dermatomyositis. In: Petty RE, Laxer RM, Lindsley CB, Wedderburn LR. Textbook of Pediatric Rheumatology. 7th edition. Philadelphia; Elsevier eds. 2016; pp 351-383

2. Clarkson B, Thompson D et al. Cyclical edema and shock due to increased capillary permeability. *Am J Med* 1960; 29:193

3. Matsumura M, Kakuchi Y, Hamano R et al. Systemic capillary leak syndrome associated with compartment syndrome. *Int Med* 2007; 46: 1585-87

4. Druey KM, Parikh SM. Idiopathic systemic capillary leak syndrome (Clarkson disease). *J Allergy Clin Immunol* 2016. pii: S0091-6749(16)32460-5

5. Guffroy A, Dervieux B et al. Systemic capillary leak syndrome and autoimmune disease: a case series. *Sem Arthritis Rheum* 2017: 46: 509-12

6. Xie Z, Frith K et al. Idiopathic Systemic Capillary Leak Syndrome in Children. *Pediatrics* 2015; 135: e730-35

**Disclosure of Interest**

None Declared.

**Table 27 (Abstract P277).** Laboratory changes during the systemic capillary leak syndrome in JDM

	Case 1		Case 2		Case 3	
	On admission	SCLS	On admission	SCLS	On admission	SCLS
CK (U/L)	2291	32407	17744	18900	1022	11360
AST (U/L)	214	1500	561	822	1039	603
ALT (U/L)	95	570	165	112	487	108
Albumin (g/L)	36	22	32	21	38	20
Hct (%)	38.5	54.7	40.2	48.2	35	47.6
WBC (el/mm3)	9250	46250	13210	22560	8810	29010
Neutrophils el/mm3)	5490	40240	10410	18533	5250	20307
Na^++^ (mmol/L)	140	122	136	125	138	123

### P278 Clinical and laboratory assessment of 87 patients with juvenile dermatomyositis

#### Juan Manuel Mosquera Angarita^1^, Estefanía Quesada^2^, Daniel Clemente^3^, Alina Boteanu^4^, Juan Carlos Nieto^5^, Leyre Riancho-Zarrabeitia^6^, Judith Sánchez-Manubens^7^, Esmeralda Nuñez^8^, María José Lirola^9^, Sara Copete^10^, Marisol Camacho-Lovillo^11^, Melania Martínez-Morillo^12^, Paula Alcañiz^13^, Marta Medrano^14^, María Antonia Carballo^15^, Jordi Antón^1^, Estíbaliz Iglesias^1^

##### ^1^Hospital Sant Joan de Déu, Barcelona, Spain; ^2^Hospital Vall d’Hebrón, Barcelona, Spain; ^3^Hospital Niño Jesús, Madrid, Spain; ^4^Hospital Ramón y Cajal, Madrid, Spain; ^5^Hospital Gregorio Marañón, Madrid, Spain; ^6^Hospital Marqués de Valdecilla, Santander, Spain; ^7^Corporació Sanitaria Parc Taulí, Sabadell, Spain; ^8^Hospital Materno-Infantil Carlos Haya, Málaga, Spain; ^9^Instituto Hispalense de Pediatría, Sevilla, Spain; ^10^Hospital Universitario Reina Sofía, Córdoba, Spain; ^11^Hospital Infantil Virgen del Rocío, Sevilla, Spain; ^12^Hospital Germans Trias i Pujol, Badalona, Spain; ^13^Hospital Virgen de la Arrixaca, Murcia, Spain; ^14^Hospital Miguel Servet, Zaragoza, Spain; ^15^Hospital Xeral-Cíes, Vigo, Spain

###### **Correspondence:** Juan Manuel Mosquera Angarita

**Introduction:** Juvenile Dermatomyositis (JDM) is the most common inflammatory myopathy in children. Diagnosis is based on Bohan&Peter criteria. Stepwise treatment improves control and reduces disease related complications.

**Objectives:** To describe clinical course of 87 patients with JDM and determine whether clinical or laboratory features could be used as prognostic items

**Methods:** Clinical and laboratory data of 87 patients included in the Spanish registry of JDM between 2013 and 2016 were reviewed. Duration of untreated illness was designated as the time from first sign of rash or weakness to a diagnostic visit. Laboratory, muscle and cutaneous inactivity were defined as normal muscular enzymes values, absence of muscle symptoms and normal Childhood Myositis Assessment Scale (CMAS), when available, and absence of skin rashes respectively.

**Results:** 55 of the 87 (63%) patients were girls. Gottron’s papules were the most common skin rash at diagnosis (89.5%). Heliotrope rash was present in 67%. Eighty seven percent patients presented muscle symptoms (82% hips, 76% shoulders, 61% axial). CMAS was available in 26 patients at diagnosis with a median value of 34/52 (minimum 9/52). Baseline demographic data and laboratory findings are described in Table 28. Eighty eight percent patients had Von Willebrand Factor Antigen elevated at diagnosis. Electromyography was available in 71 patients with myopathic pattern in 62 (87%). Magnetic resonance imaging (MRI) was available in 51/87 patients at diagnosis: Ninety percent patients had pathological MRI (80% hips, 76% shoulders, 63% paravertebral); Sixty two percent had diffuse MRI pattern; Forty nine percent had soft tissue edema. Videofluoroscopy was available in 12 patients with pathological results in 9 (75%). Ninety two percent (31/34) had nailfold capillary changes, 15% (2/13) abnormal pulmonary function test and 4% (2/52) abnormal echocardiography. Ninety eight percent (49/50) had pathological muscle biopsy.

Laboratory, muscle and cutaneous inactivity were obtained at 8.9, 14.1 and 21.3 months respectively (Standard deviation (SD) 11.5, 26.8, 31.4 respectively). Forty three percent had JDM relapse, mostly cutaneous relapse, in a median time of 13.2 months. Thirty three percent had presented JDM associated complications (69% calcinosis, 10% macrophage activation syndrome).

Heliotrope rash and abnormal swallowing were associated with presence of complications (heliotrope rash (n = 58/86 p): complications: 39.7 vs 17.9%, p = 0.043, OR 3.02; abnormal swallowing (n = 9/12 p): complications: 44.4 vs 0%, p = 0.49). Higher erythrocyte sedimentation rate (ESR), longer time of active disease until treatment, and lower CPK values were associated with longer time to reach inactivity.

Yr: years, m: months, ESR: erythrocyte sedimentation rate, CRP: C reactive protein, CPK: creatine phosphokinase, AST: aspartate aminotransferase, ALT: alanine aminotransferase, LDH: lactate dehydrogenase.

**Conclusion:** Outcome of JDM is heterogenous. Heliotrope rash, abnormal swallowing, higher ESR, longer time of active disease until treatment and lower CPK values were associated with poor prognosis. Identifying these features at diagnosis could improve treatment and monitoring of these patients.

**Disclosure of Interest**

None Declared.

**Table 28 (Abstract P278).** Baseline demographic, clinical characteristics and laboratory findings

	Mean	Median	Standard deviation
Age at disease onset (yr)	7.1	6.4	3.8
Time between disease onset and diagnosis (m)	6.3	2.6	10.6
ESR (mm/h; <15)	21.7	16	21
CRP (mg/L; <15)	4.4	2.9	6.9
CPK (UI/L;62-235)	1889.3	353	2968
AST (UI/L;2-50)	172.2	92	219.1
ALT (UI/L-2-31)	104.9	66.5	97.4
LDH (UI/L; <647)	853.9	645	616.5

### P279 Expression of type I and type II interferons is increased in muscle biopsies of juvenile dermatomyositis patients and related to clinical and histological features

#### Rebecca Nicolai^1^, Gian Marco Moneta^1^, Silvia Rosina^2^, Chiara Fiorillo^2^, Denise Pires Marafon^1^, Margherita Verardo^3^, Luisa Bracci-Laudiero^1,4^, Carlo Minetti^5^, Angelo Ravelli^5^, Fabrizio De Benedetti^1^

##### ^1^Division of Rheumatology, Ospedale Pediatrico Bambino Gesù IRCCS, Roma, Italy; ^2^University of Genova, Genova, Italy; ^3^Unit of Neuromuscular and Neurodegenerative Disease, Ospedale Pediatrico Bambino Gesù IRCCS, Roma, Italy; ^4^Institute of Translational Pharmacology, CNR, Roma, Italy; ^5^University of Genova, Istituto Giannina Gaslini IRCCS, Genova, Italy

###### **Correspondence:** Rebecca Nicolai

**Introduction:** There is growing evidence for an involvement of interferons (IFNs) in the chronic inflammation that characterizes juvenile dermatomyositis (JDM). Additional information is needed to support the hypothesis of a pathogenic role of IFNs in JDM in order to provide the rationale for therapies aimed at specifically targeting type I IFN and/or type II IFN pathway in JDM.

**Objectives:** The aim of this study was to investigate muscle expression of type I (IFNα/β) and type II (IFNγ) IFN inducible genes in muscle biopsies of JDM patients and their correlations with clinical and histological aspects of the disease.

**Methods:** In a retrospective cohort of patients diagnosed with JDM (n = 35), expression of the six genes part of the so called type I IFN score (IFI27, IFI44L, IFIT1, ISG15, RSAD2, SIGLEC1), as well as of IFNγ and of CXCL9, CXCL10, CXCL11, CIITA, were analysed by real-time PCR on snap-frozen muscle biopsies and compared with biopsies from Duchenne muscular dystrophy (DMD) patients (n = 24) and 4 healthy controls (HC). Expression levels of CXCL9, CIITA (genes specifically induced by IFNγ) and IFNγ itself were used to generate a type II IFN score. We also analyzed mRNA expression of the pro-inflammatory cytokines interleukin-1β (IL-1β), tumor necrosis factor-α (TNFα) and interleukin-6 (IL-6). Patient charts were reviewed to record clinical features at diagnosis and long term outcomes: physician’s global assessment of disease activity, serum levels of muscle enzymes, erythrocyte sedimentation rate, C-reactive protein level, antinuclear antibodies status, time to inactive disease and time to prednisone (or equivalent) dose 0.2 mg/kg/daily, and relapses. We furthermore evaluated typical histological aspects of JDM (inflammatory infiltrate, necrosis, perifascicular atrophy and fibrosis) on tissue sections of the muscle biopsies.

**Results:** JDM patients treated (n = 12) with systemic glucocorticoids before biopsy were excluded from analysis, because expression levels of the studied genes were markedly reduced compared to untreated patients. The type I IFN score and type II IFN score were significantly higher in the muscle of untreated JDM patients (n = 23) compared with controls (p < 0.0001, p < 0.001 respectively). Expression of TNFα, but not of IL-1β and IL-6, was significantly (p < 0.05) higher in untreated JDM muscle biopsies compared with those of controls. Type I IFN score correlated with inflammatory infiltrate and necrosis, while the type II IFN score correlated with inflammatory infiltrate, perifascicular atrophy and fibrosis. Type I IFN score, type II IFN score and TNFα expression significantly correlated with physician’s global assessment at diagnosis (r = 0.38, r = 0.42, r = 0.62, respectively). When analyzing correlations with long term outcomes, we found that patients with elevated type II IFN score reached clinically inactive disease significantly later than patients with low type II IFN score (log-rank chi square value = 10.1, p < 0.01).

**Conclusion:** IFN type I and type II scores in muscle biopsies of JDM patients correlate with clinical and histological features suggesting a pathogenic role of IFNs in muscle damage and inflammation in JDM. Noteworthily the type II score correlated with time to clinically inactive disease, suggesting a correlation with disease severity.

**Disclosure of Interest**

R. Nicolai: None Declared, G. M. Moneta: None Declared, S. Rosina: None Declared, C. Fiorillo: None Declared, D. Pires Marafon: None Declared, M. Verardo: None Declared, L. Bracci-Laudiero: None Declared, C. Minetti: None Declared, A. Ravelli: None Declared, F. De Benedetti Grant/Research Support from: Novartis, Novimmune, Hoffmann-La Roche, SOBI, AbbVie, Pfizer

### P280 Growth and puberty in juvenile dermatomyositis, a longitudinal multicenter printo study

#### Ellen Nordal^1,2,3^, Angela Pistorio^3^, Marite Rygg^3^, Gabriella Giancane^3^, Michael Hofer^3^, Jose Antonio Melo-Gomes^3^, Blanca Bica^3^, Ximean Norambuena^3^, Valda Stanevicha^3^, Rebecca Ten Cate^3^, Olga Vougiouka^3^, Jurgen Brunner^3^, Guenther Dannecker^3^, Polyxeni Pratsidou-Gertsi^3^, Gabriele Simonini^3^, Helen Venning^3^, Serena Pastore^3^, Angelo Ravelli^3^, Alberto Martini^3^, Nicolino Ruperto^3^ and the Paediatric Rheumatology International Trials Organisation (PRINTO)

##### ^1^Department of Pediatrics, University Hospital of North Norway, Tromsø, Norway; ^2^Department of Pediatrics, UiT The Arctic University of Norway, Tromsø, Norway; ^3^Pediatria II Reumatologia, Istituto Giannina Gaslini, Genova, Italy

###### **Correspondence:** Ellen Nordal

**Introduction:** Children with juvenile dermatomyositis (JDM) are at risk of growth failure and delayed puberty because of inflammatory disease activity and side effects of corticosteroid treatment. Knowledge on growth and pubertal development in JDM is very limited.

**Objectives:** To study growth and puberty in a multicenter longitudinal prospective cohort of children with JDM.

**Methods:** Anthropometric and pubertal data in children ≤18 years with recent onset or flare of JDM from 31 countries were studied. Growth failure was defined as parent-adjusted z-score < -1.5, and height deflection z-score < -0.25/year. Delayed menarche was defined as age of menarche >15 years. Late and delayed pubertal onset was defined as age at Tanner B2 > 11 years and >13 years for girls, respectively, or age at Tanner T2 > 12 and >14 years for boys, respectively. Delayed puberty was defined as a delay in pubertal onset, pubertal tempo or menarche.

**Results:** Height and weight from four follow-up visits during two years in 196/275 (71.3%) of the children included in the original JDM study were analyzed. There was a significant reduction in parent-adjusted height z score over time in females (p < 0.0001) and males (p = 0.001) with significant gender difference (p < 0.05), but also catch-up growth at the final study visit were seen. Median body mass index (BMI) z score peaked at 6 months (p < 0.0001) and was still significantly above baseline with no gender difference at the final study visit at a median of 26 months after baseline (p = 0.007). Females with a disease duration ≥12 months after onset had significantly lower parent-adjusted height z score (p = 0.002) with no catch-up growth. Delayed pubertal onset, pubertal tempo and delayed menarche was found in a substantial number of the participants as shown in the table. Growth failure at base line was the main determinant of growth failure at the final study visit (OR: 53.4).

Numbers are frequencies (%) unless otherwise specified.

**Conclusion:** Children with JDM of long duration are at risk of having a lower than expected height, higher BMI, and a delayed pubertal development.

**Disclosure of Interest**

None Declared.

**Table 29 (Abstract P280).** Growth and pubertal characteristics at the final study visit of participants in a longitudinal JDM cohort

	Girls (N = 55)	Boys (N = 31)
Growth failure	20/97 (20.6%)	11/73 (15.1%)
Height deflection	29/116 (25.0%)	25/80 (31.3%)
Age at B2 females (n = 37)/T2 males (n = 23), mean (SD)	11.1 (1.9)	12.8 (1.6)
Menarche registered	31/55 (56.4%)	n.a.
Delayed menarche	7/31 (22.6%)	n.a.
Late pubertal onset	23/37 (62.2%)	15/23 (65.2%)
Delayed puberty	20/55 (36.4%)	11/27 (40.7%)

### P281 Quality of life evaluation in new onset juvenile dermatomyositis patients from the PRINTO trial

#### Andressa Guariento, Elena Fueri, Gabriella Giancane, Francesco Zulian, Angelo Ravelli, Bo Magnusson, Tadej Avcin, Fabrizia Corona, Valeria Gerloni, Claudia Bracaglia, Rolando Cimaz, Antonella Meini, Silvana Martino, Anne Pagnier, Michel Rodiere, Christine Soler, Valda Stanevicha, Rebecca Ten Cate, Yosef Uziel, Jelena Vojinovic, Simona Angioloni, Luca Villa, Michele Pesce, Irene Gregorini, Chiara Pallotti, Alberto Martini, Angela Pistorio, Nicolino Ruperto

##### Istituto Giannina Gaslini, Pediatria II, Reumatologia – PRINTO, Genoa, Italy

###### **Correspondence:** Nicolino Ruperto

**Introduction:** Juvenile dermatomyositis (JDM) is the most common clinical pediatric idiopathic inflammatory myopathy and it may severely compromise the quality of life of affected patients. Early adequate immunosuppressive treatment can improve the quality of life in JDM as in other rheumatic diseases like juvenile idiopathic arthritis (JIA).^1^

**Objectives:** To evaluate the HRQoL change over time in children with new-onset JDM patients enrolled in the PRINTO trial^2^.

**Methods:** In the PRINTO JDM trial, children aged 18 years old or younger with newly diagnosed and untreated probable or definite JDM were enrolled. All patients received three daily pulses of intravenous methylprednisolone at onset, and then they were randomized to one of the following three different treatment groups: prednisone (PDN), prednisone plus ciclosporin (PDN + CSA) and prednisone plus methotrexate (PDN + MTX).

In our study, we considered patients who had a complete HRQoL assessment at onset through the Child Health Questionnaire (CHQ). We compared quality of life among the responders (PRINTO20-50-70) belonging to the three treatment groups, and between responder and not responder patients. Moreover we compared the affected patients to healthy children.

**Results:** Out of a total of 139 patients enrolled in the PRINTO JDM trial, 129 (92.8%) were retained for the analysis (41.9% males and 58.1% females; median age 7.4 years). At baseline, patients with JDM showed poorer scores in quality of life (PhS = 15.6 and PsS = 40.0) than healthy children. In particular, “Physical Summary score” (PhS) turned out to be significantly lower than 2 standard deviations with respect to the mean value of the healthy children with no difference among the 3 treatment groups (PDN, PDN + MTX, PDN + CSA). Less compromised scores were observed for the PsychoSocial summary Scale, with values between 1 and 2 standard deviations below the mean values of healthy children. We found similar values between «responder 20» and «not responders». A statistically significant improvement was observed after 6 months of treatment both in the PhS and in the PsS subscales (P < 0.0001) irrespective of the treatment group, and in all items of the CHQ with the exception of the GBE (Global behavior parameter). A significant improvement over time was observed regardless of the treatment group and the level of response (PRINTO 20, 50, 70). “Responder” patients showed PhS values significantly higher than “not responder”; on the contrary, PsS values did not reveal a significant difference between “responders” and “not responders”, despite an improvement over time.

**Conclusion:** Children with new-onset JDM, treated with PDN alone or in combination with other therapies, showed a significant improvement in quality of life during a two-year follow-up. According to the “PRINTO 20-50-70” criteria, “responder” patients showed a statistically significant improvement in the PhS score compared to the “not responders”.

**References**

1. Ce´spedes-Cruz A et al. Ann Rheum Dis 2008;67:309–314. 

2. Ruperto N et al. Lancet 2016;387:671–78.

**Disclosure of Interest**

A. Guariento: None Declared, E. Fueri: None Declared, G. Giancane: None Declared, F. Zulian: None Declared, A. Ravelli Grant/Research Support from: BMS, Hoffman-La Roche, Janssen, Novartis, Pfizer, Sobi., Speaker Bureau of: AbbVie, BMS, Pfizer, Hoffman LaRoche, Novartis, Centocor., B. Magnusson: None Declared, T. Avcin: None Declared, F. Corona: None Declared, V. Gerloni: None Declared, C. Bracaglia: None Declared, R. Cimaz: None Declared, A. Meini: None Declared, S. Martino: None Declared, A. Pagnier: None Declared, M. Rodiere: None Declared, C. Soler: None Declared, V. Stanevicha: None Declared, R. Ten Cate: None Declared, Y. Uziel: None Declared, J. Vojinovic: None Declared, S. Angioloni: None Declared, L. Villa: None Declared, M. Pesce: None Declared, I. Gregorini: None Declared, C. Pallotti: None Declared, A. Martini: None Declared, A. Pistorio: None Declared, N. Ruperto Grant/Research Support from: 2. The G. Gaslini Hospital, which is the public Hospital where I work as full time public employee, has received contributions from BMS, Hoffman-La Roche, Janssen, Novartis, Pfizer and Sobi for the coordination activity of the PRINTO network. This money has been reinvested for the research activities of the hospital in fully independent manners besides any commitment with third parties., Speaker Bureau of: Abbvie, Ablynx, AstraZeneca, Baxalta Biosimilars, Biogen Idec, Boehringer, Bristol Myers Squibb,, Eli-Lilly, EMD Serono, Gilead Sciences, Janssen, Medimmune, Novartis, Pfizer, Rpharm, Roche, Sanofi.

### P282 Comparison of treatment approach, side effects and cumulative damage of children with juvenile dermatomyositis followed in two European tertiary care referral centers

#### Giulia Camilla Varnier^1^, Alessandro Consolaro^1^, Clarissa Pilkington^2^, Sue Maillard^2^, Silvia Zaffarano^1^, Alberto Martini^1^, Angelo Ravelli^1^ and Juvenile Dermatomyositis Reaserch Group, JDRG, London, UK

##### ^1^Paediatric Rheumatology, IRCCS Istituto Giannina Gaslini, Genoa, Italy; ^2^Paediatric Rheumatology, Great Ormond Street Hospital, london, UK

###### **Correspondence:** Giulia Camilla Varnier

**Introduction:** Juvenile dermatomyositis (JDM) is a rare condition with a wide severity range, from self-limiting to life threatening. Its treatment will vary between different pediatric rheumatology centers as no uniform therapeutic protocols are available, especially based on the severity of disease presentation. Previous studies have documented the improvement of disease prognosis, but the impact of the most recent treatment strategies on side effects and cumulative damage is still insufficiently documented.

**Objectives:** To compare the treatment approach based on the disease severity, the side effects and the cumulative damage of children with JDM followed in two large European tertiary care centers.

**Methods:** To be included in the study, patients had to be diagnosed with JDM between 2000 and 2015 at the Istituto Giannina Gaslini (IGG) Genoa, Italy or the Great Ormond Street Hospital (GOSH), London, UK. Data regarding clinical outcome measures, treatment interventions, side effects and cumulative damage, assessed with the Myositis Damage Index (MDI), were collected at first observation and then at 6, 12 and 24 months.

**Results:** A total of 127 patients were included, 88 at GOSH and 39 at IGG. Demographic data, including age of onset and disease duration at first visit, were almost identical between patients followed at the two centers. Based on the physician clinical judgment, we divided our patients into Mild, Moderate or Severe disease activity. At GOSH and IGG the Mild and Moderate patients were initially treated with corticosteroids (88% vs 100%) and methotrexate (98% vs 100%), then at GOSH azathioprine (37%), and anti-TNF (31%) were used in the second year of follow up, whilst cyclosporine (50%) and iv immunoglobulin (33%) were preferred at IGG. Children with Severe disease activity at GOSH also received iv cyclophosphamide (67%), whilst cyclosporine (46%) and iv immunoglobulin (23%) were administrated at IGG. The corticosteroid toxicity has been the most frequent side effect reported in both centers in 25% and 43.6% of patients, followed by sickness due to methotrexate showed in 25% and 20.5% of children at GOSH and IGG respectively. At GOSH there were more reports of increased blood pressure (6.8% vs 2.5%) and mood swing (10.2% vs 2.5%). At IGG increased body hair (30.8% vs 3.5%) and raised liver enzymes (10.2% vs 5.7%) were more commonly seen. Vertebral fracture occurred in 3.5% and 2.5% of patients at GOSH and IGG respectively. The percentage of patients with evidence of damage (MDI ≥ 1) in any organ or system at baseline, 6, 12 and 24 months of follow up was 9%, 13.6%, 12.3%, 12.6% at GOSH and 5.1%, 5.5%, 11.4%, 8.8% at IGG. The details of the most common signs of cumulative damage are shown in the table.

**Conclusion:** xIn spite of the differences in the treatment approach, the frequency of damage was low (< 16%) in both patient cohorts. The side effects of treatment were mostly transient and never life-threatening. The commonest side effects were due to corticosteroids: treatment protocols that reduce the use of corticosteroids would be beneficial.

**Disclosure of Interest**

None Declared.

**Table 30 (Abstract P282).** See text for description

	GOSH		IGG	
	N	N (%)	N	N (%)
Calcinosis	88	14 (16)	39	3 (7.7)
Cutaneous Scarring	88	13 (14.8)	39	6 (15.4)
Muscular Weakness	88	5 (5.7)	39	2 (5.1)
Joint contracture	88	4 (4.5)	39	3 (7.7)
Lipodystrophy	88	4 (4.5)	39	1 (2.5)
Chronic infections	88	3 (3.4)	39	1 (2.5)
Vertebral fracture	88	3 (3.4)	39	1 (2.5)
Others	88	6 (6.7)	39	8 (2.3)

### P283 Cone beam computed tomography for the assessment of linear scleroderma of the face

#### Chiara Di Giovanni^1^, Stefano Puggina^2^, Alessandra Meneghel^1^, Sabina Trainito^3^, Giorgia Martini^1^, Francesco Zulian^1^

##### ^1^Department of Woman and Child Health, University of Padova, Padova, Italy; ^2^Unix Radiology Service, Affidea Group, Piove di Sacco, Italy; ^3^Division of Physical Medicine and Rehabilitation, Department of Neuroscience, University of Padova, Padova, Italy

###### **Correspondence:** Francesco Zulian

**Introduction:** Linear scleroderma of the face (LSF) is a very disabling condition and, to date, standardized and validated methods for assessing and monitoring the disease progression are lacking.

The Cone Beam Computed Tomography (CBCT) is an imaging technique, currently used in dentistry and maxillofacial surgery, with good sensitivity for both soft and bony tissues, it is fast to be performed therefore does not require patient sedation. Of interest, the radiation exposure is 50 times lower than a traditional CT scan, being the ideal technique for a repeated use.

**Objectives:** Given the young age of the majority of the patients with LSF and the need for a combined bone and soft tissue evaluation, we investigated whether CBCT may represent a potential reliable tool for assessing the patients and for monitoring the course of LSF over time.

**Methods:** Ten consecutive patients with LSF, aged 3-21 years, and 5 age-matched healthy controls underwent CBCT assessment. The transverse sections of CBCT scan, in digital format, were analyzed according with three arbitrarily selected anatomic levels: mandibular condyle (MC), floor of the maxillary sinus (MS) and mandibular foramen (MF). Measurements of both affected and unaffected side of the face were made by a standardized methodology. From the intersection axes, an origin point was generated and from this one, 30° and 60° lines, crossing bony and soft structures, were drawn. For each given degree, the soft tissue thickness and the total thickness (bony and soft tissues) of the right and left side were calculated by using the software Onis 2.4 free edition. Twenty-four measures for each subject (two for each side, right and left, of the three transverse sections) were therefore evaluated.

Five raters, all physicians, after a one-hour training session, evaluated LSF patients’ and controls’ CBCTs twice and blindly one from the other. The intra-rater reliability was assessed by the repeatability coefficient and the inter-rater reliability by the Intraclass Correlation Coefficient (ICC) and interpreted as follows: ICC values range 0.75-1 excellent reliability, 0.4-0.74 good reliability, <0.4 poor reliability.

All statistical analyses were performed by using IBM SPSS (Vers. 18.0).

**Results:** CBCT was fast and well tolerated by the patients even the youngest.

The intra-rater concordance resulted optimal as the repeatability coefficient ranged between 0.77 and 0.99.

The inter-rater concordance for the total thickness, among the assessors, was also excellent with mean ICC value of 0.75 (SD 0.16) for patients and 0.89 (SD 0.09) for controls. The mean ICC for the soft tissue thickness was 0.49 (SD 0.24) for patients and 0.66 (SD 0.28) for controls, respectively. 58.3% of the measurements for patients and 91.2% of those for controls showed excellent ICC results (> 0.75). The best performances were obtained at the level of the MF and MC sections.

**Conclusion:** CBCT has shown to be a reliable method to assess and monitor skin and bone changes in patients with linear scleroderma of the face. It is a fast, reproducible and reasonably safe technique. Although it is not reliable for the assessment of lesions on the forehead, CBCT is indicated for the assessment of the remaining parts of the face. A prospective validation to confirm its relevance in evaluating the disease progression is ongoing.

**Disclosure of Interest**

None Declared.

## Juvenile dermatomyositis

### P284 Correlations between clinical and objective muscle scores in juvenile dermatomyositis assessed after long-term follow-up

#### Kristin S. Berntsen^1^, Truls Raastad^2^, Berit Flatoe^1,3^, Ivar Sjaastad^4,5^, Helga Sanner^1,6^

##### ^1^Department of Rheumatology, Oslo University Hospital, Rikshospitalet, Oslo, Norway; ^2^Norwegian School of Sports Sciences, Oslo, Norway; ^3^Faculty of Medicine, University of Oslo, Oslo, Norway; ^4^Institute of Clinical Medicine, Faculty of Medicine, University of Oslo, Oslo, Norway; ^5^Institute for Experimental Medical Research, Oslo University Hospital and University of Oslo, Oslo, Norway; ^6^Norwegian National Advisory Unit on Rheumatic Diseases in Children and Adolescents, Oslo University Hospital, Rikshospitalet, Oslo, Norway

###### **Correspondence:** Kristin S. Berntsen

**Introduction:** The unilateral manual muscle test (MMT-8) and the childhood myositis assessment scale (CMAS) are regarded as important measures of muscle strength and function/endurance in juvenile dermatomyositis (JDM). However, due to ceiling effects the tests are known to have low sensitivity and specificity in detecting mild muscle weakness that is often predominant in long term JDM. Isokinetic knee extension is a more objective evaluation of muscle power and endurance.

**Objectives:** To explore the association between the MMT-8, CMAS, and an isokinetic knee extension test in JDM patients with long term disease.

**Methods:** JDM patients and gender- and age-matched controls from the general population were examined. Patients were divided into active and inactive disease by the PRINTO criteria (2012) [1]. Height and weight were measured, and MMT-8 (0-80) was scored in both groups; CMAS (0-52) in patients only. An isokinetic knee-extension device with an attached force transducer (HBM U2AC2, Darmstadt, Germany) was used to measure maximal voluntary contraction (MVC) force as a measure of muscle strength. At 30% of the MVC force, the participants then performed repetitive one-leg knee extensions at 0.25Hz to exhaustion. Exercise volume (mass derived from the 30% MVC force x number of repetitions) was used as a measure of muscle endurance. Values represent averages of right and left leg.

**Results:** Forty-five patients (60% women) and 50 controls participated in the study. Mean disease duration from symptom onset was 20.6 (11.9) years, 28 (62%) patients had inactive disease and 11 (24%) were on medication. Muscle strength (MMT-8 and MVC force) and endurance (exercise volume) were reduced in patients compared to controls and in patients with active compared to inactive disease. CMAS was not statistically different between the patient groups (Table 31). In patients, MVC force showed moderate correlation with MMT-8 (Spearmans rho (r) 0.419, p = 0.001) and weak correlation with CMAS (r = 0.368, p = 0.013). Exercise volume showed moderate correlations with both CMAS and MMT (r = 0.506 and r = 0.435 respectively, p’s ≤ 0.003).

**Conclusion:** Isokinetic knee extension force and endurance were significantly lower in patients than controls and in active versus inactive JDM after long term follow-up. The tests showed weak to moderate correlations with MMT-8 and CMAS and might aid in the detection of mild muscle weakness after long term disease.

1. Lazarevic D, Pistorio A, Palmisani E, et al. The PRINTO criteria for clinically inactive disease in juvenile dermatomyositis. *Ann Rheum Dis* 2013;72:686-93.

**Disclosure of Interest**

None Declared.

**Table 31 (Abstract P284).** See text for description

	Patients (n = 45)	Controls (n = 50)	p	PRINTO active (n = 17)	PRINTO inactive (n = 28)	p
Age	28.6 (11.9)	29.8 (12.5)	0.640	30.0 (14.5)	27.8 (10.1)	0.594
Height	168 (12)	171 (10)	0.141	163 (14)	170 (9)	0.049
Weight	67.6 (17.6)	66.6 (14.3)	0.848	63.5 (20.1)	69.4 (15.9)	0.277
MMT-8	78 (75-79)	80 (79-80)	<0.001	76 (73-77)	78 (77-79)	0.003
CMAS	50 (48-52)	NA	NA	49 (46-52)	51 (49-52)	0.110
Force (N)	322 (104)	383 (126)	0.012	273 (98)	353 (96)	0.010
30% mass (kg)	9.8 (3.0)	11.5 (3.8)	0.017	8.3 (2.6)	10.6 (2.9)	0.010
Repetitions (#)	25 (9)	29 (7)	0.067	22 (8)	27 (9)	0.072
Exercise volume (kg)	256 (138)	340 (174)	0.012	194 (121)	292 (137)	0.018

### P285 Abdominal manifestations in juvenile dermatomyositis

#### Caroline M. Besnard^1^, Magali Guarella^2^, Nicole Fabien^3^, Pierre Quartier^1^, Lucile Musset^4^, Nathalie Bardin^5^, Marine Desjonquères^6^, Brigitte Chabrol^7^, Anne-Laure Jurquet^2^, Karine Retornaz^2^, Alexandre Belot^6^, Brigitte Bader-Meunier^1^

##### ^1^Unité d’Immunologie-Hématologie et Rhumatologie Pédiatrique, CHU Paris - Hôpital Necker Enfants Malades, APHP, Paris, France; ^2^Service de médecine infantile et de rhumatologie pédiatrique, CHU Hôpital Nord, APHM, Marseille, France; ^3^Laboratoire d’immunologie, Centre Hospitalier Lyon Sud, HPL, Lyon, France; ^4^Département d’immunochimie, GH Pitié-Salpêtrière, APHP, Paris, France; ^5^Laboratoire d’immunologie, CHU Conception, APHM, Marseille, France; ^6^Service de néphrologie, rhumatologie et dermatologie pédiatrique, Hôpital Femme mère enfant, Université Lyon, HPL, Lyon, France; ^7^Service de neurologie pédiatrique, CHU Timone Enfant, APHM, Marseille, France

###### **Correspondence:** Caroline M. Besnard

**Introduction:** Juvenile dermatomyositis (JDM) is an idiopathic inflammatory myopathy in children.

**Objectives:** To describe the frequency of abdominal manifestations at presentation of JDM and at follow-up.

**Methods:** Medical records of patients with JDM followed up in 3 French pediatric centres (Paris, Lyon and Marseille) were reviewed and abstracted for abdominal manifestations. Inclusion criteria were: 1) diagnosis of dermatomyositis according to Peter and Bohan before 18 years-old; 2) presence of an abdominal manifestation (hepatic, pancreatic and gastrointestinal) at presentation or at follow-up. Severe manifestation was defined by at least one intensive care unit stay for a life-threatening condition. Etiologies will be classified as specific related to JDM, treatment-related and infectious.

**Results:** We included 60 patients with JDM, from 3 French pediatric centres. Mean age at diagnosis were 7 years and 2 months. Forty-four children (75%) presented with abdominal manifestations: hepatic (39/44), pancreatic (5/44) and gastrointestinal (24/44). The etiologies included JDM-related hepatitis (33/34), treatment-related hepatitis (7/34), JDM-related pancreatitis (4/5), treatment-related pancreatitis (1/5), mesenteric lipomatosis (2/24), intestinal vasculitis (2/24), occlusive syndrome (1/24). Clinical presentation of children with abdominal manifestations in JDM were more edematous, inflammatory, extra-muscular (including articular, lung and swallowing involvement) and more treatment lines were required. Nine patients (20.5%) had severe abdominal manifestations. They had abdominal manifestations at diagnosis (7/9) and at last follow-up (3/9), with a mean of 3 different organs involved. They received specific treatments, including parenteral nutrition (6/9), plasmapheresis (3/9) and immunoadsorption (4/9). Mortality rate was 5% (3/60), all 3 children with severe abdominal manifestations. Myositis-specific auto-antibodies were negative for 64.7% of patients without abdominal manifestations, 47.7% of patients with abdominal manifestations and 44.4% of patients with severe abdominal manifestations. Abdominal imaging displayed a diffuse infiltration of fat or mesentery. Digestive (4) and liver (2) biopsies, performed in 5 patients, showed a macrophagic or lymphoplasmocytic inflammation and diffuse macrovacuolar steatosis.

**Conclusion:** Abdominal manifestations are frequent in juvenile dermatomyositis. Severe abdominal manifestations are associated with a poor prognosis.

**Disclosure of Interest**

None Declared.

### P286 Muscular weakness in children with juvenile dermatomyositis induces gait deviations with propulsion reduction in level walking

#### Matthias Hartmann^1^, Josephine Merker^1,2^, Katharina Koestner^1^, Boris Huegle^1^, Renate Haefner^1^, Ansgar Schwirtz^2^, Johannes-Peter Haas^1^

##### ^1^German Center for Pediatric and Adolescent Rheumatology, Garmisch-Partenkirchen, Germany; ^2^Department of Biomechanics in Sports, Technical University of Munich, Munich, Germany

###### **Correspondence:** Matthias Hartmann

**Introduction:** Juvenile dermatomyositis (JDM) is a rare disease with an incidence of 1:300.000/year. Active JDM results in severe functional impairment with muscular atrophy and -weakness of the trunk and the limbs. Patients may develop joint contractures, calcinosis and osteoporosis with great impact on their daily life. Validated instruments to measure the muscular limitations are the MMT8 and the CMAS but only little data exist concerning the functional damage of JDM in simple daily life actions like walking.

**Objectives:** The aim of this single-center study was to analyze relieving postures in the kinematic gait function of JDM patients by a clinical 3D gait analysis (3DGA).

**Methods:** Twelve JDM patients (♀ = 8; age = 10.4yr (± 3.6); weight = 35.3kg (± 13.8); height = 136cm (± 20.1)) were included and 12 healthy, age-matched peers served as control group (CG) (♀ = 7; age = 10.8yr (± 3.0); weight = 35.0kg (±10.7); height = 142cm (± 15.6)). To measure muscular limitations the CMAS was used and the creatine kinase (CK) was determined as sensitive test for skeletal muscle damage. Clinical 3DGA was performed with eight infrared cameras (Vicon-MX F-40 (200Hz)) and the Plug-In-Gait model. All participants walked with a free chosen velocity. At least six right and left gait-cycles were analyzed for the comparisons in spatiotemporal and kinematic gait parameters in the sagittal plane of the pelvis, hip, knee and ankle joints. For the analysis a reduction to one side were done due to the fact of a typically symmetric affection. With given normal distribution the unpaired t-test was used for statistics (p < 0.05).

**Results:** Mean values in the JDM group were a CMAS of 26.7/52 (± 16.4), CK of 459U/L (SD = ± 1228; min = 24; max = 4527) and a disease duration of 2.6 years (± 2.6) with four patients within the first 12 months. The patients walking-speed was clearly slower (1.07m/s (± 0.22)) than in the CG (1.27m/s (± 0.09); p < 0.01). No kinematic changes were found in the sagittal plane of the pelvis and hip joints during the gait cycle. Deviations occurred in the Range of Motion (RoM) of the knee flexion in the loading response phase. This force absorbing flexion motion of the knee joint was less distinct in JDM (JDM = 8.7° (± 5.9); CG = 16.2° (± 4.0); p < 0.001) and the following extension motion was slightly reduced (p = 0.105). The maximal extension position in the knee that occurs in the single support phase of walking showed significantly lower values in JDM (JDM = 4.9° (± 4.5); CG = 10.2° (± 3.3); p < 0.01). The foot position in JDM had a less pronounced sole angle in relation to the ground in the moment of initial contact (JDM = 9.9° (± 10.0); CG = 18.0° (± 2.2); (p < 0.05)) with an accompanied reduced ankle plantarflexion motion in the loading response phase. In the push off motion the ankle RoM differs strongly with smaller values in the JDM group (JDM = 26.3° (± 5.7); CG = 34. 8° (± 6.0); p < 0.01). Moreover, a distinct reduction of the ankle dorsiflexion in the swing phase was observed in 6/12 patients.

**Conclusion:** JDM patients showed gait deviations with predominant involvement of the knee and ankle joints. We observed common compensatory signs of muscular weakness with the effect of reduced propulsion especially in the ankle push off phase. This results in slower walking speed of JDM patients. Next step will be to include kinetic joint data and the results of manual measured muscle scores to get more detailed relations about muscle strength and compensatory movement.

**Acknowledgements**

“Ironman-Hilfe Kinderrheuma”, the association “Hilfe für das rheumakranke Kind” and the foundation “Deutsche Kinderrheuma-Stiftung”.

**Disclosure of Interest**

None Declared.

### P287 Development of consensus-based treat-to-target protocols for the management of juvenile dermatomyositis in Germany

#### Claas Hinze^1^, Prasad Oommen^2^, Frank Dressler^3^, Ulrike Schara^4^, Frank Weller-Heinemann^5^, Jürgen Brunner^6^, Dirk Föll^1^, Elke Lainka^4^, Ulrich Neudorf^4^, Tobias Schwarz^7^, Fabian Speth^8^, Andreas Urban^9^, Wolfgang Müller-Felber^10^, Johannes-Peter Haas^11^ and Working Group Juvenile Dermatomyositis of the German Society for Pediatric Rheumatology’s PROKIND Initiative

##### ^1^Pediatric Rheumatology and Immunology, University Hospital Münster, Münster, Germany; ^2^University Hospital Düsseldorf, Düsseldorf, Germany; ^3^Hannover Medical School, Hannover, Germany; ^4^University Hospital Essen, Essen, Germany; ^5^Klinikum Bremen Mitte, Bremen, Germany; ^6^Medical University Innsbruck, Innsbruck, Austria; ^7^St. Josef-Stift, Sendenhorst, Germany; ^8^Medical University Rostock, Rostock, Germany; ^9^Amberg Hospital, Amberg, Germany; ^10^University Hospital Munich, Munich, Germany; ^11^German Center for Pediatric and Adolescent Rheumatology, Garmisch-Partenkirchen, Germany

###### **Correspondence:** Claas Hinze

**Introduction:** Juvenile dermatomyositis (JDM) is the most common inflammatory myopathy in childhood and causes substantial morbidity. Treatment of JDM in Germany is very heterogeneous and should be improved. The German Society for Pediatric Rheumatology’s (GKJR) initiative PROKIND has the goal to develop consensus-based protocol regarding the diagnosis, therapy and monitoring of pediatric rheumatic diseases.

**Objectives:** To develop consensus-based statements and protocols regarding the diagnosis, therapy and monitoring of JDM in Germany.

**Methods:** The working group was established in 2015. Members included pediatric rheumatologists, pediatric neurologists and dermatologists with an expertise in the management of JDM (JDM experts). Specific goals of the project were identified by consensus. Via an online survey among pediatric rheumatologists and neurologists (67 participants) using different case scenarios, various approaches were identified. These approaches were harmonized. A final consensus meeting took place in January 2017 (13 participants). By means of nominal group technique individual statements were developed. Consensus was considered to be achieved if more than 80% of participants supported a statement.

**Results:** Overall, ten individual statements were developed, each with a consensus of 92-100%, regarding (1) diagnosis of JDM, (2) case definitions for the application of the protocols, (3) initial diagnostic work-up, (4) monitoring and documentation, (5) treatment targets for a treat-to-target strategy, (6) supportive measures, (7) explicit definition of a treat-to-target strategy, (8) different glucocorticoid treatment options, including intermittent intravenous methylprednisolone pulse therapy and high-dose oral glucocorticoid therapy and suggestions for tapering, (9) initial glucocorticoid-sparing treatment, and (10) treatment in case of refractory disease.

**Conclusion:** We developed individual statements regarding the management of JDM by means of a consensus process among JDM experts in Germany. These statements and resulting treatment protocols may be applied to patients with active moderately severe and severe JDM. Long-term goals of the project include the establishment of a documentation platform for JDM in Germany, the participation in international registries and further optimization of these protocols.

**Disclosure of Interest**

None Declared.

### P288 Anti-ku antibodies in two paediatric cases with juvenile dermatomyositis-scleroderma overlap and literature review: why is it important to know?

#### Parichat Khaosut^1,2^, Simone Castagno^3^, Abimbola Aluko^3^, Muthana Al-Obaidi^1^

##### ^1^Paediatric Rheumatology, Great Ormond Street Hospital, London, UK; ^2^Paediatrics, Faculty of Medicine, Chulalongkorn University, Bangkok, Thailand; ^3^Faculty of Medicine, Imperial College London, London, UK

###### **Correspondence:** Parichat Khaosut

**Introduction:** Juvenile dermatomyositis (JDM) is a heterogeneous autoimmune-mediated disease characterized by proximal muscle weakness, elevated levels of muscle enzymes, and rashes typically heliotrope rash and Gottron’s papules. Other clinical features that may contribute to major morbidity include calcinosis, skin ulceration, treatment-resistant rash, and internal organ involvements of the gut, lungs, and brain. Serologic investigation has been explored in JDM in order to define subgroups that can help us predict clinical course, treatment and prognosis. Autoantibodies directed against the Ku autoantigen are one of myositis associated antibodies found in adult patients with systemic sclerosis (SSc) and have been associated with myositis overlap and interstitial lung disease (ILD). However, there is a paucity of data on the clinical correlates of anti-Ku antibodies in the paediatric autoimmune conditions.

**Objectives:** The aim of this study was to review clinical phenotype, prognosis and treatment of anti-Ku antibodies in two paediatric cases with juvenile dermatomyositis-scleroderma overlap.

**Methods:** 380 of the patients enrolled in the Juvenile Dermatomyositis Cohort and Biomarker Study (JDCBS) were tested for autoantibodies (at the time of this study) and we found only two cases who have anti-Ku positivity as myositis-associated autoantibodies (MAA). The review of patient medical notes were performed with the comparison between disease characteristics of anti-Ku antibodies in our patients and other case studies.

**Results:** We report a 10 year old male with anti-Ku associated myositis/scleroderma overlap syndrome characterized by destructive myopathy, sclerodermatous rashes, joint restriction, oesophageal dysmotility, interstitial lung disease (ILD) and possible cardiac involvement. The patient initially presented at the age of 8 as a hyperpigmented lesion over the chest and abdomen which developed into a widespread tightening and thickening of the skin. He was started on high dose intravenous steroid, subcutaneous methotrexate injection and courses of Rituximab and cyclophosphamide infusion. His clinical progression has gradually improving after six doses of cyclophosphamide. Another case review is a 10 year old girl with a three year history of gradual tightening of her fingers, progressing to her elbows, shoulders, hips, knees and back. She also complained of Raynaud phenomenon with progressive muscle weakness. After seven years of treatment with steroid and methotrexate, her disease had still active and required more aggressive treatment. Similar to previous adult cohort studies, anti-Ku positive patients are associated with more severe muscle weakness, articular symptoms, Raynaud phenomenon and a tendency to develop ILD.

To the best of our knowledge, this report is one of the youngest anti-Ku positive patients with juvenile dermatomyositis-scleroderma overlap.

**Conclusion:** Our review suggests that anti-Ku antibodies might be used to identify individuals with juvenile dermatomyositis-scleroderma overlap whose disease will progress to severe muscle weakness and systemic internal organ involvements, particularly interstitial lung disease. Prompt aggressive treatment should be managed according to the severity of symptoms in order to improve clinical outcome and prevent serious complication. Our report underscores the screening for anti-Ku antibodies at diagnosis in JDM patients presenting with scleroderma features such as Raynaud phenomenon to guide further investigation and initiate appropriate treatment.

The written informed consent for the publication of these details was obtained from the participants.

**Disclosure of Interest**

None Declared.

### P289 Capillary microscopy in juvenile dermatomyositis is not strongly correlated to clinical and laboratory markers of disease activity

#### Katharina Köstner^1^, Boris Hügle^1^, Fabian Speth^2^, Johannes-Peter Haas^1^

##### ^1^German Center for Pediatric and Adolescent Rheumatology, Garmisch-Partenkirchen, Germany; ^2^Rheumatology, Universitätskinderklinik Rostock, Rostock, Germany

###### **Correspondence:** Katharina Köstner

**Introduction:** Juvenile dermatomyositis is a systemic inflammatory disease in childhood, with a predominantly vascular pattern of injury. Vascular changes are frequently documented using nailfold capillary microscopy. Other markers to monitor disease activity include the childhood myositis activity scale (CMAS) and laboratory markers including neopterin.

**Objectives:** The objective of this study was to correlate various capillary microscopy findings in patients with JDM with markers of clinical activity (CMAS) and laboratory markers of disease activity (neopterin).

**Methods:** Consecutive patients admitted to the German Center for Pediatric and Adolescent Rheumatology from September 2008 until December 2016 were included in this study. Inclusion criteria were diagnosis of JDM according to the criteria by Bohan and Peter. All patients received capillary microscopy following a standardized institutional protocol, at the time of diagnosis and on follow-up appointments. Capillary density (capillaries per mm) was determined by trained investigators, as well as presence of mega-capillaries, nailbed edema, sludge or hemorrhages and the morphologic shape of the capillaries. The following data were extracted by retrospective chart survey: age, age at diagnosis, medication, disease activity measured by CMAS and neopterin levels. Data were analyzed using descriptive statistics as well as non-parametric statistics (Mann-Whitney U or Kruskal-Wallis-Test) as appropriate.

**Results:** 47 JDM patients with 85 examinations were included (77% female). CMAS showed no significant correlation to capillary density (r = 0.200, p = 0.139), nor with the presence or absence of megacapillaries (p = 0.164), nailfold edema (p = 0.607), sludge (p = 0.280), hemorrhages (p = 0.817) or various levels of morphologic changes (0.444). Neopterin levels showed significant correlations with elevations in patients with megacapillaries (p = 0.019) and morphologic changes (p = 0.013). Neopterin levels did not correlate to capillary density (r = 0.200, p = 0.139), nailfold edema (p = 0.699), sludge (p = 0.508) or hemorrhages (p = 0.304).

**Conclusion:** Capillary microscopy as a marker of vasculitis is not correlated to clinical activity as measured by CMAS, but morphologic changes and development of microcapillaries are correlated with neopterin levels as a laboratory marker of myositis.

**Disclosure of Interest**

None Declared.

### P290 A profile of myositis autoantibodies in an irish cohort of juvenile dermatomyositis (JDM)/myositis patients

#### Jayne M. Macmahon^1^, Vincent Tormey^2^, Emma J. MacDermott^1^, Orla Killeen^1^

##### ^1^National Centre for Paediatric Rheumatology, OLCHC, Dublin, Ireland; ^2^Department of Immunology, NUIG, Galway, Ireland

###### **Correspondence:** Jayne M. Macmahon

**Introduction:** Juvenile dermatomyositis is an auto-immue inflammatory vasculopathy of uncertain aetiology that affects skin and striated muscle. It is historically diagnosed by proximal muscle weakness, characteristic skin lesions and evidence of muscle inflammation. It has an incidence in UK and Ireland of approximately 1.9 per million [1]. In recent years, myositis autoantibodies have been identified that may play a role in further distinguishing subtypes of inflammatory myositis. They can be broadly divided into myositis-specific autoantibodies (MSA) and myositis-associated autoantibodies (MAA) [2].

**Objectives:** To document the presence or absence of myositis autoantibodies in an Irish cohort of JDM/myosits patients and to describe the clinical phenotype in those with the presence of a positive autoantibody.

**Methods:** A retrospective review of myositis autoantibody sampling over a 3 year period in patients with JDM/inflammatory myositis was performed. Myositis autoantibody analysis was made using traditional laboratory methods including immuno-blotting, reverse immunoprecipitation and immunoprecipitation-immunodepletion.

**Results:** Mysositis autoantibodies were identifed in 25 patients. 24 had a diagnosis of JDM, of which 1 had amyotrophic JDM and the remaining patient has a diagnosis of idiopathic inflammatory myositis (IIM). 11 patients tested were found to be positive for MSA/MAA. 8 of these were MSA positive and included 3 MDA 5 positive, 1 Mi2 alpha weak positive, 1 Mi2 alpha and PL-7 positive, 1 TIF-1 gamma positive, 1 SRP and Pl12 positive and 1 Anti-MJ positive. The 3 MAA positive patients included 1 PM-Scl 175 weak positive, 1 PM-Scl175 and Ro52 positive and 1 Ro52 positive.

**Conclusion:** Myositis auto-antibodies were present in a significant number of our cohort, MSA accounting for the majority (73%). Anti MDA 5 antibody accounted for 38% of those with a positive result and 12% of the overall cohort. This is slightly higher than previously quoted studies in Caucasian/UK populations [3]. The presence of anti MDA 5 is known to be associated with a more aggressive clinical course with more pronounced interstitial lung disease, arthritis and cutaneous manifestations such as skin ulcerations. This was reflected in our population of MDA 5 positive patients.

[1] Symmons DP, Sills JA, Davis SM. The incidence of Juvenile Dermatomyositis: results from a nation-wide study. Br J Rheumatol. 1995; 34(8):732-6.

[2] Ghirardello A, Borella E, Beggi M, et al. Myositis autoantibodies and clinical phenotypes. Autoimmun Highlights. 2014; 5:69-75.

[3] Tansey SL, Betteridge ZE, Gunawardena H, et al. Anti-MDA5 autoantibodies in juvenile dermatomyositis identify a distinct clinical phenotype: a prospective cohort study. Arthritis Res Ther. 2014 Jul 2;16(4):R138.

**Disclosure of Interest**

None Declared.

### P291 Clinical characteristics of children and adolescents from the Brazilian registry of myositis: an inception cohort study

#### Claudia S. Magalhaes^1^, Adriana Sallum^2^, Teresa Robazzi^3^, Ana Julia Moraes^4^, Sheila Oliveira^5^, Flavio Sztajnbok^6^, Luciana Carvalho^7^, Luciana Marques^8^, Maria Teresa Terreri^9^, Silvana Sacchetti^10^, Simone Appenzeller^11^, Ana Paula Vecchi^12^, Andre Cavalcante^13^, Marcia Bandeira^14^, Claudio Len^9^, Clovis Silva^15^

##### ^1^Sao Paulo State University (UNESP), Botucatu, Brazil; ^2^University of Sao Paulo (I Cr-USP), Sao Paulo, Brazil; ^3^Bahia Federal University (UFB), Salvador, Brazil ^4^Para Federal University (UFP), Belem, Brazil ^5^Rio de Janeiro Federal University (IPPMG-UFRJ), Rio de Janeiro, Brazil ^6^Rio de Janeiro State University (UERJ), Rio de Janeiro, Brazil ^7^University of Sao Paulo (USP-Ribeirao), Ribeirao Preto, Brazil ^8^Albert Sabin Hospital, Fortaleza, Brazil ^9^Sao Paulo Federal University (UNIFESP), Sao Paulo, Brazil ^10^Santa Casa Medical School, Sao Paulo, Brazil ^11^Campinas State University (UNICAMP), Campinas, Brazil ^12^Children Hospital of Goiania, Goiania, Brazil ^13^Pernambuco Federal University (UFPE), Recife, Brazil ^14^Pequeno Principe Hospital (HPP), Curitiba, Brazil ^15^University of Sao Paulo (ICr-USP), Sao Paulo, Brazil

###### **Correspondence:** Claudia S. Magalhaes

**Introduction:** A clinical registry is an organized database about epidemiology, clinical features and outcome, which is useful for studying rare diseases.

**Objectives:** Investigate clinical aspects of idiopathic inflammatory myopathies, or myositis syndromes, through a Brazilian multicenter registry.

**Methods:** The clinical registry was set up in an electronic data-collection platform. Trained pediatric rheumatologists enrolled recently diagnosed patients according to a standard protocol. A previous retrospective registry protocol was published (Sato J et al. Clin Exp Rheumatol 2009,27:1031). Ethical approval for all centers was obtained and training offered about key outcome assessments: Muscle strength by MMT-80, CMAS, physician-parents disease activity (VAS), disability by CHAQ and the Disease Activity Scoring (DAS) tool. Main investigations included: muscle enzymes, full blood count, ESR, renal function, nail fold capilaroscopy, pulmonary function tests, muscle MRI, EMG, skin and muscle biopsy, calcinosis image, overlap features and auto-antibodies. Recommended treatment guidelines was distributed for all participants (Enders FB, Ann Rheum Dis 2016,0:1-12).

**Results:** Of the 27 participating centers, 13 enrolled 55 patients in 2-years. At the baseline visit the 2:1 female ratio, mean age 8.1 (SD 4) years, mean time before diagnosis 5.4 (SD 6.2) months, URI preceding diagnosis 4/54 (7%), recent immunizations 8/54 (15%) (3 HPV, 2 Influenza, 2 MMR). Bohan & Peter (B&P) criteria were fulfilled by 28/54 (52%) while modified B&P with MRI in 35/54 (65%), however abnormal MRI only in 9/11. Records of myalgia 47 (87%), fatigue 47 (87%), fever 25 (46%), facial edema 24 (44%), body edema 13 (24%), arthritis 17 (31%), dysphagia 19 (35%), dysphonia 12 (22%), calcinosis 8 (15%) and skin ulceration in 13 (24%), abnormal EMG in 25/45 and muscle biopsy 7/7. High serum muscle enzymes was recorded for all, being 67% with high CK, 87% LDH, 78% AST, 59% ALT and 92% aldolase. Positive ANA in 30/44 (68%) anti-DNA 1/30 (3%). Initial treatment included high dose prednisone 91%, combined IV methylprednisolone 44%, methotrexate 76%, hydroxychloroquine 52% in addition to sunscreen measures, IVIG 16%, cyclosporine 1.6%, azathioprine 3,6%, IV cyclophosphamide 3.6%. The baseline muscle strength, functional assessment and disease activity indices were performed in more than 80% of the cases, DAS in 100%. Median MMT80 was 43 (range 3-80); median CMAS 23 (range 2-51), CHAQ-DI 1.68 (range 0,1 – 3), median Parents-VAS 46, median disease activity-VAS 51, median DAS index 13 (range 2-20).

**Conclusion:** This preliminary analysis of a baseline visit indicates Myositis syndromes are indeed rare in this population, with a moderate to severe presentation indicated by onset edema, calcinosis and skin ulceration. Standardized outcome assessments were evaluated for the majority and mainstay prednisone and methotrexate starting treatment was conducted according to current guidelines. However, there was a limited use and availability of biomarkers, imaging and muscle biopsy.

**Disclosure of Interest**

None Declared.

### P292 The case of fatal lung injury in juvenile dermatomyositis

#### Ludmila I. Omelchenko^1^, Olena A. Oshlyanska^1^, Victoria G. Ivanova^2^

##### ^1^Department of Connective Tissue Disorders in Children, State Institute of Pediatrics, Obstetrics and Gynecology, Academy of Medical Sciences of Ukraine, Kyiv, Ukraine; ^2^Kardiorhevmatology, Regional Hospital, Kropivnitsky, Ukraine

###### **Correspondence:** Olena A. Oshlyanska

**Introduction:** According to the branch of children’s connective tissue diseases clinical register, JDM has the highest lethality among other rheumatic diseases, all cases being accompanied by lung lesions. The frequency of immunj-mediated interstitial lung lesions with JDM, according to published data, varies between 5-27% with lethality to 74%.

**Objectives:** Description of the catastrophic course of pulmonitis with the JDM.

**Methods:** Analysis of medical history

**Results:**

The girl, 9 years. The disease is found active: the child is unable to perform squats in the evaluation of Rufe test. Hospitalized: severe myopathic syndrome, tachycardia, pathognomonic cutaneous manifestations, arthritis, gingivitis, hypertransaminasemia, CK 8194 U/L LDH 3586 U/L, changes in EMG, ANA 1: 160, Jo1, Mi2 - negative. GC therapy 1 mg/kg for 3 weeks without effect, methotrexate 20 mg/week added and GC increased (1.5 mg/kg) for 2 months without effect, pulse GC - partial effect (tendency to decrease myolysis). Considering the increase of B-cells to 57.95% (3865 in μL) rituximab 400 mg No. 2 had been given off label. Initially, improvement was observed: an increase in the volume of active movements, CK 43 U/L, LDH 954 U/L. After 1 month: purulent panaritium, aphthous stomatitis, dorsalgia. Exclusion of compression fractures of the vertebrae, lungs and heart without special features. B-lymphocytes of peripheral blood are low (3.83% - 0.21 in μl), Ig total 8.65 g/l. The opening of panaritium, antibacterial therapy, IVIG, therapy with methotrexate interrupted. In 2 weeks the increase of transaminases (ALT 431, AST 73 U/l with normal CK and viral hepatitis excluded) had been noted, and therefore the restoration of DMARDs postponed.

After 1.5 months. – dorsalgias increase, starting reduction in the dose of GC, and azathioprine prescribed. Further LDH increased 955-1250-2835 U/L: isoenzyme 1 25.2%; 2 50.8%; 3 8.8%; 4 3.1%; 5 12,1% (no anemia, ECG sinus tachycardia, heart ultrasound EF 66%). After 10 days - a sudden aggravation, shortness of breath at physical exertion with minimal auscultatory symptoms, myalgia, peripheral cyanosis, erythema increase. Infiltrative changes in the lungs on the roentgenogram. Progressive aggravation. Pulse therapy GC (125 mg daily) and cyclophosphamide with AB-therapy (10 AB, including ciprofloxacin, targocid, vancomycin and 3 antifungals, including Vfend), IVIG, sildenafil - no effect: RR 40 in min., HR 132/min, transfered from mask oxygen to 70% ALV oxygenation with 80-51% saturation, against which clinical and radiological changes have grown: peripheral edema, liver enlargement, lyvedo, increased heliotrope erythema, myopathic syndrome, for the first time - a decrease in voltage and ectopic rhythms on the ECG. Procalcitonin has not grown, negative blood culture and antibodies to mycoplasmas, pneumocysts, chlamydia. 1 week on ALV saturation 88-95%, then: closed left-sided pneumothorax, drainage according to Bulau. After 3 days - arterial hypertension, supraventricular paroxysmal tachycardia, stopped, 3 days later - leukopenia, hyperthermia, colony-stimulating factors, 2 days later (4 weeks from the onset of pulmonary symptoms, 8 months from the debut) death.

**Conclusion:** the development of pulmonitis with JDM can not be predicted by the determination of topical enzymes and immunological parameters and prevented by aggressive immunotropic therapy.

**Disclosure of Interest**

None Declared.

### P293 Anti- melanoma differentiation associated gene 5(MDA5) autoantibody and interferons in juvenile dermatomyositis complicated with rapid progressive- interstitial lung disease

#### Kazuko Yamazaki

##### Pediatrics, Saitama Medical Center, Saitama Medical University, Kawagoe, Japan

**Introduction:** Clinically amyopathic dermatomyositis (C-ADM) is a subtype of dermatomyositis. Anti- Melanoma differentiation associated gene 5 (MDA5) autoantibody (Ab) was initially identified in adult Japanese patients with C-ADM and interstitial lung disease (ILD). Anti-MDA5 Ab was identified in a small population of patients with juvenile dermatomyositis (JDM) in Caucasians and was associated with skin ulceration and arthritis. Whereas, anti-MDA5 Ab was reported to be related to rapidly progressive (RP)-ILD and a poor prognosis in East-Asian patients with JDM. MDA5 is a member of the Rig-I-like receptors family that recognizes dsRNA in the cytosol, and MDA5 signals induce the antiviral responses.

**Objectives:** - To present a case of 4-year-old girl diagnosed as JDM complicated with RP-ILD.

- To measure over time changes in serum anti- MDA5 Ab, and cytokine and chemokine profiles of IFN-α, IFN-γ, Interleukin (IL)-18 and IFN-γ inducible protein (IP)-10/CXCL10.

**Methods:** The case was derived from a retrospective review of case notes and laboratory findings. The levels of IFN-α,IFN-γ, IL-18, IP-10 in frozen serums were measured by ELISA.

**Results:** A 4 year-old girl had fever, erythema on the dorsum of her hands, and stomatitis two month previously, and was referred to our hospital for the eruption and slight fever. The physical examination showed Gottron’s sign on the dorsum of her hands, periungual erythema, and erythematous rash of malar without heliotrope rash. She did not show clinical muscle weakness or muscle pain. She did not have respiratory symptoms such as cough or dyspnea. Blood tests showed raised aspartate aminotransferase (97 IU/L), lactate dehydrogenase (317 IU/L), Krebs von der Lungen-6 (KL-6) (887 U/mL), and ferritin (28.0 ng/mL). Although the level of Aldolase (11.3 U/L) was slightly increased, the level of creatine kinase (52 IU/L) was normal and no other abnormalities were observed. The anti-nuclear antibody and autoantibodies against Jo-1, SS-A/Ro, and SS-B/La, Scl-70 were not detected. We noticed the extremely high value of Anti- MDA-5 Ab: 615U/mL, where the cut-off value is 8 U/mL using ELISA. MRI of both arms and legs showed no myositis. Chest CT demonstrated ground-glass opacities and consolidations in the bilateral lower lung lobes. She was diagnosed as JDM complicated with ILD. We started the treatment with m-PSL pulse and cyclosporine. The erythema and slight fever disappeared in about 10 days, however the serum KL-6 level did not decrease and consolidations gradually extended in chest CT. We added intravenous pulse administration of cyclophosphamide. Because hypoxia worsened and mediastinal pneumothorax occurred, we started mechanical ventilation by high-frequency oscillation. The patient died from respiratory failure 4 months after hospitalization. The autopsy revealed the organizing diffuse alveolar damage.

We investigated the changes in the levels of anti MDA-5 Ab, IFN- α, IFN-γ, IP-10, and IL-18 in her serum. The level of anti MDA-5 Ab was extremely high at the first visit. Over the course of the treatment, anti MDA-5 Ab and serum IP-10 were persistently at increased levels, and serum IFN-γ and IFN-α were at undetectable levels.

**Conclusion:** Persistently increased levels of anti-MDA5 Ab and serum IP-10 may suggest a progressive and fetal clinical course in a patient with JDM complicated with ILD. Informed consent to publish has been obtained from the parent.

**Disclosure of Interest**

None Declared.

## Macrophage activation syndrome

### P294 Transfusion-related acute lung injury following platelet transfusion in a child with systemic juvenile idiopathic arthritis complicated by macrophage activation syndrome

#### Alenka Gagro^1,2^, Agneza Marija Pasini^1^, Ivancica Skaric^1^

##### ^1^Children’s Hospital Zagreb, School of Medicine, University of Zagreb, Zagreb, Croatia; ^2^Faculty of Medicine, Josip Juraj Strossmayer, University of Osijek, Osijek, Croatia

###### **Correspondence:** Alenka Gagro

**Introduction:** Macrophage activation syndrome (MAS) is a rare and potentially fatal disease, commonly associated with systemic juvenile idiopathic arthritis (sJIA) characterized also by cytopenias and coagulopathy that often require blood component transfusion. Transfusion-related acute lung injury (TRALI) is a life-threatening complication characterized by the development of progressive dyspnea, hypoxemia and bilateral pulmonary edema that occurs within 6 h of a whole blood or blood component transfusion. Mechanisms of TRALI have been postulated as both immune and non-immune mediated.

**Objectives:** The objectives of the case report are to describe a patient with sJIA complicated with MAS who developed a TRALI following transfusion of a whole blood derived platelets from a male donor.

**Methods:** Case report.

**Results:** A six-year-old girl with sJIA complicated by MAS received a whole blood derived filtered and irradiated platelets from a male donor due to profound thrombocytopenia. Approximately one hour post-infusion, she started coughing and rapidly became progressively dyspnoeic. Physical examination of the patient revealed tachycardia, tachypnoea and diffuse bilateral crepitations throughout her lungs. Hypoxemia was evident with a SpO2 persistently below 90% and increasing oxygen requirements. Administration of intravenous furosemide and hydrocortisone had no effect on her symptoms. Urgent chest radiograph showed bilateral nonhomogenous opacities but no cardiomegaly, and she was promptly intubated and placed on mechanical ventilation for two days. No evidence of acute heart failure secondary to an ischemic event or circulatory overload was found so a diagnosis of transfusion-associated circulatory overload (TACO) was unlikely. Other risk factors that might contribute to acute lung injury including sepsis were excluded. The patient’s pulmonary condition rapidly improved clinically and a radiograph taken 24 hours post-event showed marked resolution of the airspace shadowing. She was extubated and transferred out of the intensive care unit one day later, with no further pulmonary sequalae. Based on relevant criteria, a blood immunology studies were performed in order to detect HLA class I, class II, and/or granulocyte antibodies in donor’s and patient’s plasma. The presence of anti-human neutrophil antigen 3b (anti-HNA-3b) in patient’s plasma and no detectable HLA class I, class II, and granulocyte antibodies in donor’s plasma indicates that in our patient TRALI might resulted from the reverse mechanism in which the recipient has granulocyte antibodies that match residual donor white blood cells.

**Conclusion:** Although acute hypoxic pulmonary failure has been described rarely in children with sJIA complicated with MAS, it is important to consider TRALI in transfusion settings in these children.

**Disclosure of Interest**

None Declared.

### P295 Next generation sequencing analysis of familial haemophagocytic lymphohistiocytosis (HLH) related genes in macrophage activation syndrome and secondary HLH

**Abstract withdrawn**

### P296 IFNG and IFNG inducible genes are strongly up-regulated in the liver from a patients with active secondary hemophagocytic lymphohistiocytosis (HLH)

#### Giusi Prencipe, Ivan Caiello, Manuela Pardeo, Antonella Insalaco, Giulia Marucci, Fabrizio De Benedetti, Claudia Bracaglia

##### Division of Rheumatology, IRCCS Bambino Gesù Children’s Hospital, Rome, Italy

###### **Correspondence:** Giusi Prencipe

**Introduction:** An increasing body of evidence, in humans and in animals, support the hypothesis that IFNg plays a pivotal role not only in the pathogenesis of primary HLH, but also of secondary HLH (sHLH). Nevertheless, few data demonstrating the activation of the IFNg pathway in target tissues of patients during the active phase of the disease are available.

**Objectives:** To analyse the mRNA expression of IFNg and IFNg inducible genes in the liver and in the blood from a patient with sHLH and to measure the circulating levels of CXCL9, in order to evaluate whether the IFNg pathway is up-regulated.

**Methods:** A Caucasian boy presented with systemic inflammation and progressive worsening of general conditions at age of 17. At this time bone marrow (BM) was negatıve. Glucocorticoid treatment was started but a first relapse occurred during tapering. Ten months later, a third episode with progressıve multiple organ failure, and subsequent ICU admission, occurred. Laboratory parameters and BM were consistent with HLH. Analysis of primary HLH-related genes showed a heterozygous mutation (R928C) in UNC13d gene. Eight years later, he presented with progressive increasing in ferritin and transaminase (max value 2300 and 2450 of ferritin and ALT, respectively). No fever was present and general conditions were excellent. Blood count and acute phase reactant were normal. Bone marrow aspirate showed some activated macrophages. Liver biopsy showed acute hepatitis with massive infiltration from macrophages showing active haemophagocytosis.

**Results:** One liver biopsy specimen was snap-frozen for gene expression by Real-Time PCR and compared with tissue expression in a normal liver tissue. The expression of IFNg was strongly up-regulated (approximately 80 fold). Furthermore, we found that all IFNg inducible genes analysed (CXCL9, CXCL10, CXCL11 and IDO) were highly upregulated (from approximately 200 to 380 fold). In contrast, we did not observe marked up-regulation of the expression of pro- and anti-inflammatory cytokines (IL-1b, TNFa, IL-6, IL-18, IL-8, IL-10). Analysis of blood samples showed that the expression levels of CXCL9 and CXCL10 were higher (about 10 fold) while the expression levels of IFNg were lower, compared to those observed in blood from healthy donors (n = 2). Circulating plasma levels of CXCL9 were high during the active phase of the disease and paralleled the increase (max levels 6000 pg/ml) and the decrease in ferritin levels respectively during worsening and the subsequent progressive clinical improvement during treatment.

**Conclusion:** We show marked up-regulation of the IFNg pathway in the liver tissue from a patient with sHLH in which the disease was limited to the liver, without systemic involvement. No increased expression of other inflammatory cytokines was found. The selective activation of the IFNg pathway supports the pathogenic role of this pathway in HLH. The marked tissue up-regulation was leaked in the peripheral blood with increase in CXCL9 expression and CXCL9 protein levels. Our data support the hypothesis that circulating CXCL9 levels reflect tissue activation of the IFNg pathway and therefore represent a useful HLH biomarker.

**Disclosure of Interest**

None Declared.

## Scleroderma and related syndromes

### P297 Clinical case: congenital systemic scleroderma

#### Minira Bulegenova^1^, Gulmira Erdjanova^2^, Aliya Aytbaykyzy^3^

##### ^1^Scientific Center of Pediatrics and Children Surgery, Almaty, Kazakhstan; ^2^Cardiorheumatology Department, Scientific Center of Pediatrics and Children Surgery, Almaty, Kazakhstan; ^3^Cardiorheumatology, Scientific Center of Pediatrics and Children Surgery, Almaty, Kazakhstan

###### **Correspondence:** Minira Bulegenova

**Introduction:** The term scleroderma means “sclerosis”, stiffening of the “dermis” and the skin. Scleroderma has two main forms: systemic sclerosis (adults suffer more frequently) and localized scleroderma - more often revealed in children.Both types of scleroderma are rare in children, the annual incidence of systemic scleroderma (SSCL) makes up 1 cases per 1 million children, whereas the incidence of local scleroderma (lSCL) makes up 1-3 cases per 100.000 children. Aggressive form of the disease and early involvement of the internal organs may resul in severe disability and even fatal outcome in children. The percentage otf the lethal cases is lower when the progression of the disease is slow.

**Objectives:** In our work, we have presented a very rare case of congenital scleroderma. Patient F.D. was charged in Scientific center of pediatrics and children surgery at the age of 2.5 months old.

**Methods:** The child’s health status at the first admission was of moderate severity due to skin syndrome. Child gained weight in compliance with the age. The facial features are sharp, the mimicry is poor. On the lower extremities, in the region of the shin, the external surface of the thighs, on the lower part of the trunk the skin was tight and stiffened with a dense consistency, was not folded,the surface of the skin was glistering. Under the skin, as a result of the subcutaneous adipose tissue atrophy, there was tuberosity, painless. Cyanotic feet.Joints were the normal shape and size and there was limitation of knee and hip joints motion due to the fibrosclerotic changes of periarticular tissues.

Heart tones were clear, rhythmic. Enlargement of the liver: +1.0 + 1.5 + 1.5. These changes are common for following nosologies: SCL, phenylketonuria, sclera. To clarify the diagnosis, it was necessary to exclude feniketonuria, detect ANA, the consultation of a neurologist, a dermatologist.

Whole blood parameters were within references.Biochemistry - normal, coagulation parameters - normal.Ultrasound of the abdominal cavity organs showed reactive changes in the liver and pancreas, effusion in the abdominal cavity.Echocardiography: the left ventricle cavity was stretched, a moderate dilatation of the left ventricle.Consultation of a dermatologist: congenital systemic scleroderma.Treatment: prednisolone, ibuprofen, proserin, encephabol, plaquenyl.Health status at discharge: a positive dynamics was observed in the softening of the skin, increasing the volume of movements in the joints.

Re-entry 12.05.2016. SKL, induration stage, Raynaud’s syndrome, carditis.

The health condition is severe due to a systemic disease. Child poorly gained the weight. The facial features are sharp, the bird’s face, cyanosis of the nose tip and nasolabial triangle. Hair on the head is absent. Pronounced blood vessels on the head. New spots of the skin stiffening were revealed on the back and abdomen. Feet and brush are cold. Reynaud’s syndrome. The deformation of the thorax by the type of “shoemaker”.

Laboratory studies - persistent leukocytosis.Ultrasound of the abdominal cavity organs: strengthening of the liver blood vessels.ECG: sinus tachycardia, partial blockade of Guiss bundle right leg. Violation of the repolarization in the ventricles myocardium.

X-ray of the thoracic cavity organs: lungs are clean, the pulmonary artery is not dilated. Pulsation of the blood flow is revealed in the abdominal aorta. The heart is widened in diameter. Carditis

**Results:** Taking into consideration the progression of the disease such as widening of the skin lesions, Reynaud’s syndrome it was decided to change the therapy and use a pulse-therapy with prednizolone (28 mg/kg) and MabThera (rituximab) - 35 mg/body surface (which is 180 mg).

Status at discharge. Moderate improvement, lower limbs and hands are warm.

At this time the child is under the supervision of the pediatrician at the place of residence. There are no significant changes in the health status after treatment.

**Conclusion:** This clinical case is of special interest because the congenital form of systemic scleroderma was diagnosed in Kazakhstan for the first time and there are difficulties in predicting the disease outcome.

**Disclosure of Interest**

None Declared.

### P298 Juvenile scleroderma: epidemiology, clinical and immunology features in Mexican children

#### Luis Aparicio, Maria Teresa Braña, Sofia Osorio, Yuridiana Ramirez, Andrés Rodríguez, Enrique Faugier, Rocio Maldonado, Talia Diaz

##### Pediatric Rheumatology, Hospital Infantil de México Federico Gómez, Mexico City, Mexico

###### **Correspondence:** Talia Diaz

**Introduction:** Juvenile Scleroderma is a group of rare and complex diseases with varied clinical manifestations. Juvenile scleroderma syndromes, althought rare, represent the third most frequent chronic rheumatic disease in pediatric rheumatology practice after juvenile idiopathic arthritis and systemic lupus erythematosus.

The most obvious manifestation of the diseases is skin hardening and sclerosis. The disease has two modes of onset: Localized scleroderma (LS) and Systemic Sclerosis (SSc), they share a common underlying pathophysiology of excessive collagen deposition in an autoimmune setting. Early diagnosis, knowledge of clinical and immunology features is crucial to improve the long-term outcome.

**Objectives:** Primary objective: To describe and analyze the clinical and laboratory findings in a group of children diagnosed with scleroderma at a referral hospital. Secondary objective: To establish response to treatment as well as risk factors associated with relapse.

**Methods:** Retrospective cohort study of the Children’s Hospital of Mexico Federico Gomez. Description about demographic, clinical and immunologic pattern of juvenile scleroderma in Mexico

**Results:** Sixty patients were included in the group. All of them completed the classification criteria for juvenile sclerodema, both systemic and localized. The mean age at diagnosis was 7.8 (1–15) years. The mean time from disease onset to diagnosis, based on clinical manifestations, was 2.4 years.

Most frequent type of juvenile localized scleroderma forms: mixed morphea 34% (17/50), linear scleroderma 32% (16/50), circumscribed morphea 16% (8/50), generalized morphea 16% (8/50) and pansclerotic morphea 2% (1/50). Extracutaneous manifestations more frequent associated with LS were dysphagia (10%), slerodactyly (8%), arthralgia (8%), dyspnea (6%), arthritis (4%), interstitial pneumonitis (2%), fascicular cardiac hemiblock (2%). Antinuclear antibody were present in 46% of patients with LS, anti-Scl 70 were found to be positive in 20%. The treatment protocol was combination of oral and intravenous glucocorticoid and methotrexate (10-25mg/m2/week), with only one patient with clinical relapse.

In the case of Systemic scleroderma the most frequent clinical manifestations were gastro-esophageal reflux, delayed gastric emptying, inflammatory changes of the mucosa and velopalatine insufficiency were reported in 70%, sclerodactyly (50%), arthralgia (50%), interstitial pneumonitis (30%), telangiectasia (20%), digital gangrene (20%). Positive ANA was reported in 40%, anti-Scl 70 in 40%, anti-centromere 20%. The combined protocol treatment of glucocorticoid and methotrexate (15-20mg/m2/week), currently without relapse.

**Conclusion:** The most common onset of disease was juvenile localized scleroderma, from this group the principal skin lesion was mixed morphea, and the uncommon lesion panesclerotic escleroderma. Information on the long-term outcome of children with scleroderma is very few and based on small series of patients. The knowledge of clinical and immunology features at onset of disease improve the early treatment and long-term, with the purpose of decrease sequelae that significantly impact quality of life, including permanent functional and cosmetic impairment.

**Disclosure of Interest**

None Declared.

### P299 Is there a difference in the presentation of male and female patients with diffuse subtype of juvenile systemic sclerosis? Results from the juvenile scleroderma inception cohort www.juvenile-scleroderma.com

#### Ivan Foeldvari^1^, Jens Klotsche^2^, Ozgur Kasapcopur^3^, Amra Adrovic^3^, Valda Stanevicha^3^, Teresa Terreri^3^, Ekaterina Alexeeva^3^, Maria Katsikas^3^, Vanessa Smith^3^, Jordi Anton^3^, Rolando Cimaz^3^, Mikhail Kostik^3^, Thomas Lehman^3^, W.-Alberto Sifuentes-Giraldo^3^, Flavio Sztajnbok^3^, Tadey Avcin^3^, Mahesh Janarthanan^3^, Maria Jose Santos^3^, Monika Moll^3^, Dana Nemcova^3^, Christina Battagliotti^3^, Jürgen Brunner^3^, Despina Eleftheriou^3^, Liora Harel^3^, Tilmann Kallinich^3^, Kirsten Minden^2^, Susan Nielsen^3^, Kathryn Torok^3^, Yosef Uziel^3^, Ann Stevens^3^, Clarissa Pilkington^3^, Nicola Helmus^1^

##### ^1^Hamburg Center for Pediatric and Adolescent Rheumatology, Am Schöen Klinik Eilbek, Hamburg, Germany; ^2^German Rheumatism Research Center, Berlin, Germany; ^3^jSSc Collaborative Group, Hamburg, Germany

###### **Correspondence:** Ivan Foeldvari

**Introduction:** Juvenile systemic sclerosis (jSSc) is an orphan autoimmune disease. Several adult publications looked at the differences between the clinical presentation of male and female patients with Systemic Sclerosis. There is a rarity of data regarding this topic in pediatric jSSc. The juvenile scleroderma inception cohort (www.juvenile-scleroderma.com) is a prospective standardized register for patients with jSSc.

**Objectives:** comparison of clinical characteristics of patients with diffuse subtype at the time of inclusion in the registry who are male or female.

**Methods:** Patients with jSSc were included worldwide to the juvenile scleroderma inception cohort. We compared the demographics and clinical characteristics of male and female patients.

**Results:** Up till now 88 patients were enrolled, 62 (70%) with djSSc 11/62 (18%) of the patients were male (M) and 51/62 female (F) (82%). The mean disease duration at the time of inclusion was 3.5 in M and 3.6 in F patients. The mean age of the onset of Raynaud symptoms was 8.0 in M and 9.4 years in the F patients and the non-Raynaud symptoms was 8.2 in M and 10.0 in F patients.

At the time of the inclusion the mean modified Rodnan Skin Score was 24.3 in M and 17.3 in F patients. Anti-Scl 70 positivity was found in 4/11 (36.4%) in M and 14/49 (28.6%) in F patients. Anticentromere positivity occurred in 2/11 (18.2%) (p = 0.035) in M and 0/23 (0%) in F patients. Capillary changes were present in 8/11 (73%) of the M and 30/51 (59%) of F patients, but 36% of M and F had already history of ulcerations. 7/11 (64%) of the M and 21/51 (41%) of the F patients had cardiopulmonary involvement. Only 6 F patients had pulmonary hypertension. 7/11 (64%) of M and 11/51 (22%) of F patients had signs of interstitial lung disease (p = 0.005). Renal involvement was 2/11 (18%) in M and 3/51 (6%) in F patients. 37% in both sexes had gastrointestinal involvement. 9/11 (82%) of M and 26/50 (52%) in F patients had musculoskeletal involvement. Patient global disease damage was on a VAS (0-100) 56.9 in M and in 38.4 in F (p = 0.014) and patient global disease activity was 58.8 in M and 41.9 in M (p = 0.024). Physician global of disease activity on a VAS was 58.9 in M and 36.9 in F (p = 0.004) and physician global disease damage was 60.2 in M and 31.2 in F (p = 0.001).

**Conclusion:** We present the data of the first 62 diffuse subtype patients with jSSc included in our cohort. Male Patients presented a significantly more severe disease similar to adult male patients.

**Disclosure of Interest**

None Declared.

### P300 Is there a difference in the clinical presentation of juvenile systemic scleroderma patients according the age of onset: results from the juvenile scleroderma inception cohort. www.juvenile-scleroderma.com

#### Ivan Foeldvari^1^, Jens Klotsche^2^, Ozgur Kasapcopur^3^, Amra Adrovic^3^, Valda Stanevicha^3^, Teresa Terreri^3^, Ekaterina Alexeeva^3^, Maria Katsikas^3^, Vanessa Smith^3^, Jordi Anton^3^, Rolando Cimaz^3^, Mikhail Kostik^3^, Thomas Lehman^3^, W.-Alberto Sifuentes-Giraldo^3^, Flavio Sztajnbok^3^, Tadey Avcin^3^, Mahesh Janarthanan^3^, Monika Moll^3^, Dana Nemcova^3^, Maria Jose Santos^3^, Christina Battagliotti^3^, Jürgen Brunner^3^, Despina Eleftheriou^3^, Liora Harel^3^, Tilmann Kallinich^3^, Kirsten Minden^2^, Susan Nielsen^3^, Kathryn Torok^3^, Yosef Uziel^3^, Ann Stevens^3^, Clarissa Pilkington^3^, Nicola Helmus^1^

##### ^1^Hamburg Center for Pediatric and Adolescent Rheumatology, Am Schöen Klinik Eilbek, Hamburg, Germany; ^2^German Rheumatism Research Center, Berlin, Germany; ^3^jSSc Collaborative Group, Hamburg, Germany

###### **Correspondence:** Ivan Foeldvari

**Introduction:** Juvenile systemic sclerosis (jSSc) is an orphan autoimmune disease. It was rarely looked at the differences between the clinical presentations of patients at different paediatric age groups. The juvenile scleroderma inception cohort (www.juvenile-scleroderma.com) is a prospective standardized register for patients with jSSc.

**Objectives:** comparison of clinical characteristics of patients with different age range at the time of inclusion in the registry

**Methods:** Patients with jSSc were included worldwide to the juvenile scleroderma inception cohort. We compared the demographics and clinical characteristics of the patients at different age ranges. We created 3 cohorts with different age ranges at onset of disease. Patents aged less than 5 years (Group1), 5-10 years (Group2) and over 10 years (Group3) at the time of diagnosis of the first non-Raynaud involvement of jSSc.

**Results:** Up till now 88 patients were enrolled,14 patients (15%) in Group1, 22 (25%) in Group2 and 52 (59%) in Group3. Diffuse subtype occurred in 71% in Group1, in 82% in Group2 and in 65% in Group3. Most patients were Caucasian. Disease duration at time of inclusion into the cohort was 3.9 years in Group1, 4.9 years in Group2 and 2.2 years in Group3. ANA positivity was 57% in Group1, 77% in Group2 and 86% in Group3. Anti-scl 70 was around 30% in all groups. Anti-Centromere positivity was 7 to 10%. Mean modified skin score was 12.4 in Group1, 16.5 in Group2 and 15.9 in Group3. Raynaud Phenomenon occurred in 85 to 95% of the patients. History of active or inactive ulceration occurred in 57% in Group1, 62% in Group2 and 43% in Group3. Decreased FVC under 80% occurred in 43% in Group1, 32% in Group2 and 30% in Group3. Pulmonary hypertension occurred in 7% in Group1 and in 10% in Group3. No renal hypertension was observed. Urinary sedimentary changes occurred in 7% in Group1 and in 10% in Group3. Gastrointestinal involvement occurred in 21% in Group1, 45% in Group2 and 27% in Group3. Musculoskeletal involvement occurred in 58 to 64%. Patient global disease activity (VAS 0-100) was 42.8 to 47.9. Patient global disease damage (VAS 0-100) was 39.6- to 45.0. Physician global disease activity (VAS 0-100) was 35.4-40.0. Physician global disease damage (VAS 0-100) was 37.1 in Group1, 41.3 in Group2 and 27.7 in Group3.

**Conclusion:** It seems to be that patients, with onset of the disease in younger age have more severe disease as patients with disease onset after the age of 10 years. We need more patients in our cohort to gain more sufficient data to prove our preliminary observation.

**Disclosure of Interest**

None Declared.

### P301 Assessing the prevalence of juvenile systemic sclerosis in childhood using administrative claims data from the United States

#### Ivan Foeldvari^1^, Timothy Beukelman^2^, Fenglong Xie^2^

##### ^1^Hamburg Center for Pediatric and Adolescent Rheumatology, Am Schöen Klinik Eilbek, Hamburg, Germany; ^2^University of Alabama at Birmingham, Birmingham, AL, USA

###### **Correspondence:** Ivan Foeldvari

**Introduction:** Juvenile systemic Sclerosis (jSSc) is an orphan disease. There are some data regarding the incidence, but nearly no data exist regarding the prevalence of jSSc. To plan prospective trials, it is very important to gain data regarding the prevalence of jSSc.

**Objectives:** To estimate the prevalence of jSSc in the United States using administrative claims data

**Methods:** We used Truven MarketScan® commercial insurance claims data from the United States for the years 2010 through 2014, inclusive. In each individual calendar year, we identified all persons in the claims data who were less than 16 years old. Among these persons, we identified all persons with at least 1 physician diagnosis code for systemic sclerosis (ICD-9 710.1) and then removed any person with a diagnosis code for localized scleroderma (ICD-9 701.0). From this remaining group of patients, we identified patients with at least one filled prescription or infusion claim for immunosuppressant medications often used to treat systemic sclerosis, namely methotrexate, mycophenolate mofetil, or cyclophosphamide. We assumed these children had jSSc and calculated the estimated prevalence and 95% confidence interval using a Poisson distribution.

**Results:** The results for each calendar year are shown in the Table. Very few children received diagnosis codes for systemic sclerosis. Approximately 70% of these patients did not have concurrent diagnoses of localized scleroderma, but only approximately 14% of those had treatment with immunosuppressant medications often used to treat systemic sclerosis. The prevalence estimates were approximately 3 per 1 million children and were relatively stable from year to year.

**Conclusion:** Using diagnosis codes and medication usage from administrative claims, we estimated the prevalence of jSSc to be approximately 3 per 1 million children.

**Disclosure of Interest**

None Declared.

**Table 32 (Abstract P301).** See text for description

Year	N of Total Children	Diagnosis Code for Systemic Sclerosis	No Diagnosis Code for Localized Scleroderma	Use of Methotrexate, Mycophenolate Mofetil, or Cyclophosphamide	Estimated Prevalence per 1,000,000 Children [95% CI]
2010	5,888,868	254	186	23	3.9 [2.5-5.9]
2011	6,231,475	249	185	22	3.5 [2.2-5.3]
2012	6,278,116	217	170	26	4.1 [2.7-6.1]
2013	4,950,018	175	120	17	3.4 [2.0-5.5]
2014	4,933,522	138	91	14	2.8 [1.6-4.8]

### P302 Proposal for a juvenile systemic sclerosis response index(JSSCRI): result of the consensus meeting in hamburg. Germany 11th of December 2016

#### Ivan Foeldvari^1^, Dan Furst^2^, Jordi Anton^3^, Eileen Baildem^4^, Michael Blakley^5^, Tamas Constantin^6^, Patrícia Costa Reis^7^, Megan Curran^8^, Maurizio Cutolo^9^, Chris Denton^10^, Kim Fligelstone^11^, Bernd Hinrichs^12^, Francesca Ingegnoli^13^, Antonia Kienast^1^, Dana Němcova^14^, Claire Pain^4^, Clarissa Pilkington^15^, Vanessa Smith^16^, Dinesh Khanna^17^

##### ^1^Hamburg Center for Pediatric and Adolescent Rheumatology, Am Schöen Klinik Eilbek, Hamburg, Germany; ^2^UCLA, Los Angeles, CA, USA; ^3^Sant Joan de Déu Hospital, Barcelona, Spain; ^4^Alder Hey Children’s Foundation NHS Trust, Liverpool, UK; ^5^Indiana University, Indianapolis, IN, USA; ^6^Semmelweis University, Budapest, Hungary; ^7^Hospital de Santa Maria, Lisbon, Portugal; ^8^Northwestern University Feinberg School of Medicine, Chicago, IL, USA; ^9^University of Genova, Genova, Italy; ^10^Royal Free Hospital, London, UK; ^11^FESCA, London, UK; ^12^Practice Buchholz, Hamburg, Germany; ^13^University of Milan, Milan, Italy; ^14^Department of Pediatrics and Adolescent Medicine, Prague, Czech Republic; ^15^GOSH, London, UK; ^16^GU(H), Genth, Belgium; ^17^Univ Michigan, Ann Arbor, MI, USA

###### **Correspondence:** Ivan Foeldvari

**Introduction:** Juvenile Systemic Sclerosis (jSSc) is an orphan disease. There is increaseing interest to test novel therapies in management of fibrotic diseases. Therefore, is very important to develop a Response Index for jSSc (JSScRI) to separate effective therapies from placebo. In 2014 at the First Consensus Meeting in Hamburg, following two rounds of Delphi, a proposal for domains and items for a JSScRI were made. In 2016, the 2nd JSScRI Consensus Meeting was held in Hamburg, Germany.

**Objectives:** To conduct a Nominal Group Technique (NGT) and select potential core set items (outcome measures) that will be incorporated in the development of a Response Index for jSSc.

**Methods:** Before the 2nD JSScRI Consensus Meeting, the items from the first JSScRI Consensus Meeting (2014) were scored in a Delphi by the participants to the current meeting. Participants included 14 experts in adult and juvenile SSc and a patient partner. During the subsequent face to face NGT meeting, moderated by DEF, the items were scored anonymously by the participants after a nominal group discussion moderated by DEF. The domains and items were scored regarding their importance for 1 year clinical trial. The items were scored 1 (not relevant at all) to 9 (most relevant). A Priori, it was agreed by the participants that the goal oft he NGT was to exclude items that are 1. Not feasible and 2. Don’t represent an outcome measure but may represent the impact of disease on quality of life and vocational and avocational activity. Items that scored a median score of <4 or where greater than 1/3 of participants scored 1-3 [despite the item have a median score > 3] were excluded.

**Results:** 71 items in 13 domains were scored. 6 Items were not scored as they were felt not to represent an outcome measure or non-feasible and and 6 items received a median score less then 4.

Table 33 provides an example of musculoskeletal involvement with Median score for different outcome measures. ll).

**Conclusion:** In a rigorous, NGT consensus meeting, some item reduction for the JSScRI was achieved. These will be tested in a prospective, inception cohort of juvenile SSc patients. (www.juvenile-scleroderma.com)

**Disclosure of Interest**

None Declared.

**Table 33 (Abstract P302).** Assessment of the Activity of the Musculoskeletal involvement

	Whole Group	1 – 3	4 – 6	7 - 9
11) Swollen Joints	7	0	1	12
12) Limited Range	7.5	1	1	10
13a) MMT	8	0	1	11
13b) CMAS	7	4	1	7
14) Presence of tendon friction rub	7	1	2	10
15a) CK	8	1	1	11
15b) Aldolase	7	3	3	7

### P303 Clinical evaluation of 34 children with systemic sclerosis:a multicenter study

#### Reza Sinaei^1^, Yahyah Aghighi^2^, Reza Shiari^1^, Seyyed Reza Raeeskarami^2^, Fatemeh Fereshteh Mehrgan^1^, Vahid Ziaee^2^, Khosro Rahmani^1^, vadood Javadi Parvaneh^1^, Mehrnoush Hassas Yegane^1^, Arman Ahmadzadeh^3^, Mohammad Reza Fathi^1^, Sara Pezashki^4^

##### ^1^Pediatric Rheumatology, Shahid Beheshti Univesity of Medical Sciences, Tehran, Iran, Islamic Republic of; ^2^Pediatric Rheumatology, Tehran University, Tehran, Iran, Islamic Republic of; ^3^Rheumatology, Shahid Beheshti Univesity of Medical Sciences, Tehran, Iran, Islamic Republic of; ^4^Internal Medicine, Kerman Medical University, Kerman, Iran, Islamic Republic of

###### **Correspondence:** Reza Sinaei

**Introduction:** Systemic scleroderma is a connective tissue disease charecterized by fibrosis and multisystem involvment. Classification into diffuse or limited forms depends on the extent of skin thicknening and calcinosis; wheather the limited form is a mild form of diffuse scleroderma (dss) or a separate entiety is uncertain. Nontheless, both of them are rare conditions specially in childhood that they account for less than 3% of all age cases. As compared with adults, children at diagnosis show less organ involvments and may have their diagnose during adolescence.

**Objectives:** The purpose of present study is demanstrating of demographic and analysis of 34 childrern with Scleroderma and clinical charectristics of them.

**Methods:** We used all patients files who were reffered to three tertiary hospital in Tehran since 2008-2016. All demographic data were analyzed by SPSS.

**Results:** Thirty four patients who met preliminary classification for Juvenile systemic scleroderma were included while two of them were limited systemic scleroderma. Disease were more frequent in females (21/34 = 61.7%). the mean age at early presentation and diagnosis were 8 years (13m-15y) and 9.29 years (18m-15y) respectively. The first presentation symptoms attributed to systemic scleroderma were Raynouds phenomen in 25 patients (73.5%), artheralgia in 14 patients (41%) and constitutional symptoms in 6 patients (17.6%). Proximal sclerosis was the essential point and then sclerodactyly in 29 cases (85.2%), Raynoud phenomen in 25 patients (73.5%), artheralgia in 21 patients (61.76%), periungual capillary involvment in 17 cases (50%). Gastero esophageal reflux in 17 patients (50%). Twelve patients (35.2%) had restrictive pattern in Pulmonary Function Test. Six (17.64%) of them had dyspnea. Nine patients (26.4%) had artheritis. Six patients (17.64%) had calcinosis and only 1 patient (2.9%) had HTN. ANA and Anti topoisomerase-1 were found in 21 (61.7%) and 12 (35.29%) of patients respectively.

**Conclusion:** Raynouds phenomen heralds the begining of disease. Sclerodactyly, mouscloskeletal symptoms, periungual capillaropathy and GI and respiratory symptoms are the most common but may not presnt at diagnosis time; Therefore, we recomend that in children we should make a diagnosis with a less rigority in completing of available classification.

**Trial registration identifying number:** No Declaration

**Disclosure of Interest**

None Declared.

### P304 A boy with the aging hands and dystrophic fingernails

**Abstract withdrawn**

## Spondyloarthritis (SpA) and enthesitis related arthritis (ERA)

### P305 Immune reconstitution inflammatory syndrome associated with juvenile enthesis-related arthritis under treatment of adalimumab and tuberculosis

**Abstract withdrawn**

## Systemic JIA

### P306 Clinical and laboratory assessment of a systemic juvenile idiopathic arthritis cohort: a retrospective study

#### Alina Lucica Boteanu, Maria Llop Vilaltella, Maria Angeles Blazquez Cañamero, Sandra Garrote Corral, Cynthia Bouroncle Alaluna, Mariluz Gamir Gamir 

##### Rheumatology, University Hospital Ramon Y Cajal, Madrid, Spain

###### **Correspondence:** Alina Lucica Boteanu

**Introduction:** The sJIA, currently included according to the ILAR classification in the JIA category as one more category despite having clinical features and pathophysiology different from the other categories, represents approximately 5-15% of JIA. It has a low incidence, according to some studies 0.3-0.8 cases/100,000 children under 16 years. Given the clinical and analytical peculiarities and implication of the alteration of innate immunity in the pathogenesis, it has been suggested that JIA forms part of polygenic autoinflammatory diseases.

**Objectives:** To describe the general characteristics of a cohort of patients with systemic JIA and the course of the desease

**Methods:** We performed a retrospective observational study, which included 19 patients with follow-up in the Pediatric and Transitional Rheumatology Unit of our hospital.

**Results:** From the 19 patients included in our study 10 was boys and 9 girls. The mean age at diagnosis was 6,89 ± 5.3 years, with a mean in the diagnostic delay of 24.62 ± 35.1 months. The manifestations along the evolution have been: 100% of the patients presented fever, 94,4% articular manifestations, 83,3% cutaneous manifestations, 50% adenopathies, 27,8% hepatomegaly, 16,7% splenomegaly. None of our patients have presented uveitis, oral aphtha or neurological manifestations. 11.1% of the patients presented episodes of SAM. Regarding the analytical values performed during the periods of activity, our patients presented mild anemia (mean Hb 10.4 ± 1.55), leukocytosis, thrombocytosis, and elevation of RFA with mean of VSG 77 and PCR of 81.77. Elevated levels of ferritin have also been observed with a mean of 1439.

100% of the patients have received CT treatment throughout the course. As for FAME, 94.7% have been given methotrexate and 15.8% of Ciclosporin. Only 10.5% did not require biological treatment, while 36.8% of the patients received more than one biological drug. The biological drugs administered were Tocilizumab 26.3%, Canakinumab 10.5%, Anakinra 5.3%, Etanercept 5.3% and Adalimumab 5.3%. Currently, more than 50% of patients needs active treatment more than 50% of patients: 21.1% receive CT, 47.5% of Tocilizumab and 10.5% of Canakinumab.

**Conclusion:** In our cohort the distribution by sex has been similar to the description in the literature, with an equal distribution of males/females. Fever, joint and cutaneous manifestations have been the most frequent and not all patients have had arthritis. Unlike the other forms of juvenile idiopathic arthritis, no patient has had uveitis or other form of ocular involvement. Macrophage activation syndrome, one of the most serious complications of JIA has been observed in 11.1% of our patients. During the outbreaks of activity a marked increase of RFA, ferritin and platelets has been observed. As for the treatments administered, only a small number of patients did not require biological treatment, while more than 30% received more than one, the anti-IL-1 and anti-IL-6 drugs were the most frequently administered. Currently, more than 50% of patients receive biological treatment.

**Disclosure of Interest**

None Declared.

### P307 The disease burden of sJIA for patients and caregivers: an international health-related quality of life survey and retrospective chart review

**Abstract withdrawn**

### P308 Identification of best cut-off points and clinical signs for discrimination of acute lyphoblastic leukemia and systemic onset of juvenile idiopathic arthritis depending on presence of macrophage activation syndrome

#### Mikhail M. Kostik^1^, Eugenia Isupova^1^, Irina Chikova^1^, Tatyana Panova^2^, Olga Kopchak^1, 3^, Margarita Dubko^1^, Vera Masalova^1^, Ludmila Snegireva^1^, Tatyana Kornishina^1^, Olga Kalashnikova^1^, Vyacheslav Chasnyk^1^

##### ^1^Saint-Petersburg State Pediatric Medical University, Saint-Petersburg, Russian Federation; ^2^Leningrad regional Children’s clinical hospital, Saint-Petersburg, Russian Federation; ^3^Kirov’s regional children’s hospital, Kirov, Russian Federation

###### **Correspondence:** Mikhail M. Kostik

**Introduction:** Systemic onset of juvenile idiopathic arthritis (SoJIA) is a diagnosis of exclusion and required a broad spectrum of differential diagnosis. Patients with acute lymphoblastic leukemia (ALL) can have the similar to SoJIA symptoms as fever, lymphadenopathy, hepatosplenomegaly, joints pain and arthritis, increased ESR, CRP, anemia and should be discriminated from SoJIA. Presence of macrophage activation syndrome (MAS) lead to cytopenia in SoJIA and make similar ALL and SoJIA.

**Objectives:** The aim of our study was to find the best cut-off points and clinical signs allowed to discriminate patients who with ALL from patients with SoJIA depending the presence of MAS.

**Methods:** In the retrospective study were included 119 patients with SoJIA and 21 children with ALL who were admitted in the rheumatology department due to rheumatic masks with initial provisional diagnosis - SoJIA. We evaluated the presence of main clinical signs and detected the best cut-off points of qualitative variables with ROC-analysis and analysis of sensitivity and specificity. We calculated the diagnostic odds ratios (DOR) for identification the best cut-off parameters. For comparison of two independent groups was used Mann-Whitney test, chi-square test and Fisher’s exact test.

**Results:** There were differences in some parameters between three groups (Table 34). The main diagnostic signs, allowed to discriminate ALL from SoJIA despite the MAS were: number of active joints ≤3 (DOR = 4.4 (95%CI:1.5-13.2), p = 0.005), CRP < 15 mg/l (DOR = 5.6 (95%CI:1.7-18.4), p = 0.002), PLT ≤ 307x10^9^/l (DOR = 22.9 (95%CI:4.9-107.0), p = 0.0000001), WBC ≤ 8.9x10^9^/l (DOR = 50.2 (95%CI:6.3-401.3), p = 0.0000001), no rash (DOR = 39.8 (95%CI:8.4-188.5), p = 0.0000001), night bone pain (DOR = 2364.3 (95%CI:92.9-60169.9), p = 0.0000001), bone pain (DOR = 227.6 (95%CI: 12.5-4158), p = 0.0000001), pathologic fractures (DOR = 32.7 (95%CI:1.6-661.0), p = 0.0004). Predictors allowed to discriminate ALL from SoJIA without MAS were similar.

The additional predictors allowed to discriminate ALL from SoJIA with MAS were: Hb > 90 g\l (DOR = 4.3 (95%CI:1.2-14.80, p = 0.02), ferritin ≤ 755 ng/ml (DOR = 59.0 (95%CI:2.9-1184.1, p = 0.00004), ALT ≤ 205.1 U/l (DOR = 10.6 (95%CI:0.6-192.5, p = 0.03), AST ≤ 53 U/l (DOR = 58.8 (95%CI:3.3-1054.9, p = 0.00001), no splenomegaly (DOR = 3.1 (95%CI:1.0-9.3, p = 0.04), no hepatomegaly (DOR = 4.4 (95%CI:1.4-14.0, p = 0.01), no lung involvement (DOR = 15.5 (95%CI:1.9-127.6, p = 0.002), no hip involvement (DOR = 4.4 (95%CI:1.1-17.3, p = 0.02).

**Conclusion:** The found diagnostic criteria can help physicians to discriminate ALL patients with “rheumatic masks” into the group of patients with suspected SoJIA with and without MAS.

**Disclosure of Interest**

None Declared.

**Table 34 (Abstract P308).** See text for description

Parameters	ALL (n = 21)	SoJIA with MAS	SoJIA w/o MAS (n = 86)	p
WBC, x10^9^/l	6,2 (3,5-8,2)	10,6 (5,7-17,5)	17,1 (11,4-21,2)	0,00001
PLT, x10^9^/l	150,0 (105,0-198,0)	206,5 (89,0-395,0)	500,0 (386,0-632,5)	0,0000001
CRP, mg/l	13,6 (7,5-54,7)	105,5 (39,8-154,0)	54,0 (25,0-105,9)	0,023
Active joints, n	1 (1-3)	2 (1-4)	4 (2-14)	0,003
Ferritin, ng/ml	380,5 (220-537)	2893 (942-10600)	259,5 (117,5-803)	0,0000001
Rash, n (%)	1/21 (4,8%)	38/42 (90,5%)	52/74 (70,3%)	0,0000001
Night pains, n (%)	11/19 (57,9)	0/43 (0,0%)	1/75 (1,3%)	0,0000001
Bone pains, n (%)	19/21 (90,5%)	0/43 (0,0%)	0/76 (0,0%)	0,0000001
Pathological fractures, n (%)	2/21 (9,5%)	0/43 (0,0%)	0/76 (0,0%)	0,003

### P309 Recurrent macrophage activation syndrome associated with heterozygous unc 13D gene mutation in a child with systemic juvenile idiopathic arthritis

#### Balahan Makay^1^, Salih Gözmen^2^, Yılmaz Ay^2^, Tuğba Hilkay Karapınar^2^, Yeşim Oymak^2^, Nesrin Gülez^2^, Canan Vergin^2^

##### ^1^ılıca mah alkan sk 25/1 narlıdere, Izmir, Turkey; ^2^Behçet Uz Children s Hospital, Izmir, Turkey

###### **Correspondence:** Balahan Makay

**Introduction:** Recurrent macrophage activation syndrome (MAS) is rarely reported.

**Objectives:** To describe recurrent MAS in a 14-year-old boy with systemic juvenile idiopathic arthritis and heterozygous UNC 13D mutation, which may have a role in the patient’s recurrence despite the use of the HLH-2004 treatment protocol.

**Methods:** To describe recurrent MAS in a 14-year-old boy with systemic juvenile idiopathic arthritis and heterozygous UNC 13D mutation, which may have a role in the patient’s recurrence despite the use of the HLH-2004 treatment protocol.

**Results:** The patient experienced his first MAS episode when he was 9-years old, and the second one at 13-years-old. In both episodes, he was treated with HLH-2004 protocol. Genetic anaysis revealed that he had UNC 13 mutations (pK867E, a disease-causing mutation and pR928C, an insignificant variant). Perforin and STX 11 mutations were negative. He had his last MAS episode when he was 14-years-old. He was treated with pulse steroid and anakinra. However; because of severe angioedema after anakinra, anti-interleukine 1 treatment was switched to canakinumab. He is currently on metotrexate and canakinumab and achieved complete remission.

**Conclusion:** The patient’s recurrent course of MAS may have been because of the coexistence of juvenile idiopathic arthritis and heterozygous UNC 13D mutation. We suggest to search for mutations in HLH genes in recurrent MAS cases.

**Disclosure of Interest**: None Declared.

### P310 Withdrawal of canakinumab in patients with sJIA, maybe we “can”! A real life single center experience

#### Despoina Maritsi, Stavroula Papailiou, Milena Papatesta, Maria Tsolia, Olga Vougiouka

##### Second Department of Paediatrics, Medical Faculty, University of Athens, Athens, Greece

###### **Correspondence:** Despoina Maritsi

**Introduction:** The optimal duration of treatment in patients with JIA, especially systemic JIA (SJIA), is a major concern for parents and physicians alike. Innovative medicines have managed to fully control this otherwise potentially lethal condition; however questions regarding duration and cessation of treatment remain unanswered.

**Objectives:** The aim of this study was to describe the long-term outcome of children with sJIA treated with anti-IL1β monoclonal antibody (canakinumab-CAN-), in which treatment was discontinued, following sustained clinical remission; and to identify potential relapse associated risk factors.

**Methods:** Single center retrospective case study including patients with SJIA (based on ILAR criteria) who had received CAN from January 2008 to January 2017. All subjects had clinically inactive SJIA (clinical remission on medication-CRM-Wallace criteria) for two years under CAN. Patients were monitored for up to three years following attempt of CAN withdrawal. Demographics, clinical and laboratory parameters were recorded. We also collected data on medication exposure as well as clinical outcome measures. Data were analyzed using SPSS 19.0. Differences were tested with the Mann–Whitney U test using level of significance p < 0.05.

**Results:** In a total of 12 (7 girls) patients with inactive SJIA, (mean age of 8.5 years), CAN treatment was discontinued. Mean disease duration at the time of CAN initiation was 3.8 years (range from 1 to 7 years). All patients were on steroids when biologics were started (<1mg/kg prednisolone), which were discontinued within three months. Ten (80%) patients were on methotrexate (12.5mg/m^2^), one on leflunomide (20mg). They remained on the same dose of DMARDS throughout the study period. 75% of patients had received at least one other biologic before starting CAN (2 anti-TNFα, 1 abatacept, 2 tocilizumab, 3 anakinra). Two (12.5%) patients had received at least two biologics prior to CAN. One patient had received three biologics. All patients received 4mg/kg CAN at monthly intervals for at least two years. Mean duration of treatment was 28 months (range 26-40). Discontinuation was gradual (spacing) in 75% of patients. In 25% of patients CAN treatment was successfully stopped. Out of these four patients, two managed to stop methotrexate and remain in drug free clinical remission. Eight (67%) patients flared during the follow-up period. Mean duration of remission following CAN withdrawal was 2.5 months (range 1.5 to 12 months). Six of the eight patients (75%) were restarted on CAN while a short course of steroids resolved the flare in the remaining two. Subgroup analysis showed that biologic naïve patients (3/4 vs 1/8 p = 0.03) and patients with shorter disease duration (1.8 vs 3.4 years p = 0.04) had a higher chance of successful withdrawal. Relapse rate decreased proportionally to time (7/8 relapse < 6m vs 1/8 > 6m p = 0.02). The relapse percentages were identical in the abrupt versus gradual discontinuation groups (67%). Age, gender and ethnic origin did not affect clinical outcome.

**Conclusion:** This is a novel study underlining the importance of early aggressive management in SJIA, supporting the “window of opportunity” theory, which posits that early therapeutic intervention with anti-IL-1 is associated with better outcomes. Our findings are supported by data published by other groups finding that biologic naïve patients treated early show a more favorable disease course. Patients with shorter disease duration and no previous exposure to biologics showed a higher chance of attaining long-term remission. We suggest that patients remain in CRM for two years prior to discontinuation of treatment.

In conclusion, CAN withdrawal is feasible in a significant proportion of SJIA patients. Further studies are required to shed light into optimal treatment duration and factors associated with a favorable outcome.

**Disclosure of Interest**

None Declared.

### P311 Generalized lymphadenopathy in a child with systemic onset juvenile idiopathic arthritis treated with anakinra

**Abstract withdrawn**

### P312 Nasal septal perforation in a child with recurrent macrophage activation syndrome secondary to systemic juvenile idiopathic arthritis

**Abstract withdrawn**

## Systemic lupus erythematosus and antiphospholipid syndrome

### P313 Cluster analysis and clinical associations of auto antibodies in pediatric SLE - a single center cohort study

#### Sathish Kumar^1^, Siva Prasad^1^, Visalakshi Jeyaseelan^2^

##### ^1^Pediatric Rheumatology, Department of Pediatrics, Christian Medical College, Vellore, India; ^2^Department of Biostatistics, Christian Medical College, Vellore, India

###### **Correspondence:** Sathish Kumar

**Introduction:** The serological hallmark of systemic lupus erythematosus (SLE) is the presence of autoantibodies directed against multiple nuclear and cytoplasmic antigens and phospholipid components of cell membranes. Some of these autoantibodies are useful for classification purposes and are part of the American College of Rheumatology classification criteria for SLE and associated with specific clinical features. Studies in SLE have reported a tendency of these autoantibodies to occur in pairs or even in clusters, which has led to description of new clinical associations in adult onset SLE. To date, there have been few studies of autoantibody associations with clinical disease in pediatric-onset SLE

**Objectives:** 1) To evaluate the spectrum of serum autoantibodies in pediatric-onset systemic lupus erythematosus using cluster analysis and (2) to identify patients with similar autoantibody patterns and to determine their clinical associations using cluster analysis

**Methods:** A single center retrospective cohort study of all newly diagnosed pSLE seen over a 6 year period was performed. Clinical manifestations and autoantibody tests which were done at time of presentation were collected. Cluster analysis identified groups of patients with similar autoantibody profiles. Associations of these groups with clinical and laboratory features of pSLE were examined

**Results:** Cluster analysis was done for all 212 patients and we divided them into five major clusters. But in Akaike’s information criteria (AIC), we have identified that only 3cluster group analysis can be done to get better results. Cluster 1 consisted of ANA and anti-dsDNA antibodies. Cluster 2 consisted of anti-dsDNA, ANA, anti-RNP, anti-Sm autoantibody. Cluster 3 consisted of anti-dsDNA, ANA, anti-SSA, and anti-cardiolipin autoantibody. Cluster 1was characterized to have major organ involvement with increased frequency of renal, neurological and haematological manifestations. Cluster 2 was characterised by arthritis and increased frequency of raynauds phenomenon.Cluster 3 tended to have milder symptoms with more frequently having hairloss,photosensitivity and serositis.

**Conclusion:** Autoantibodies in pSLE tended to cluster together and these clusters were associated with different clinical courses. From the clinical perspective, this finding suggests that determining the complete autoantibody profile may help predict the clinical course of pSLE and identify patients at risk of developing major organ involvement**.**

**Disclosure of Interest**

None Declared.

### P314 Clinical features of systemic lupus erythematosus in boys according to 5-years experience in single center

#### Alia Latypova^1^, Maria Kaleda^1^, Svatlana Rodionovskaya^2^, Irina Nikishina^1^

##### ^1^Pediatrics, V.A. Nasonova Research Institute of Rheumatology, Moscow, Russian Federation; ^2^Pediatrics, Central Children Hospital FMBA, Moscow, Russian Federation

###### **Correspondence:** Alia Latypova

**Introduction:** Systemic lupus erythematosus (SLE) is a rare disease in pediatric patients especially in boys who along with clinical presentations diversity is an obstacle to timeous diagnosis.

**Objectives:** To analyze the clinical features of juvenile cases of SLE in boys including the age at disease onset, spectrum of manifestations, time of diagnosis verification, and therapy approach.

**Methods:** Retrospective study includes clinical data regarding all male patients (n = 17) among of 111 in-patients with definite diagnosis of SLE who were treated in single Russian center from 2011 to 2016. The age, and time of symptoms development, verification time from the beginning of clinical presentation, the therapy measures and timing, immunology evolution.

**Results:** In according to the data, the share of boys with SLE was 15,3% of cases (17 out of 111). The disease onset average age was 11,4 years (range 3,9;17,0), 29,41% were under 10 y.o. at the moment of the first clinical presentations. The average duration of the diagnosing from the first clinical manifestation was 2,6 years. The correct diagnosis of SLE within first 6 months was established only in 6 cases (35,2%). The most often wrong diagnosis included of various infectious diseases (in 52,9% of cases). Among the initial symptoms the most frequently were: hematological disturbances - 76,4% (thrombocytopenia - 35,3%, leukopenia-41,2%, anaemia - 47%), constitutional disturbances (76,47% patients), fever (58,8%), articular syndrome - 70,9% (arthralgia-47,05%, polyarthritis – 23,5%), typical cutaneous manifestations (malar rush) - 52,94%, nephritis (53%), neurological disorders (23,5%), less than 10% had Raynaud’s phenomenon, stomatitis, stomach-ache. Two male patients (11,7%) had macrophage activation syndrome (MAS) in the first 6 months of the disease. Another two male patients over 10 years old had severe presentations of SLE – the combination of nephritis and central nervous system affliction. In one case of early onset in boy SLE started with MAS and further severe skin and mucose lesions with typical for Ro-associated vasculitis had delayed (in 1,08 year) nephritis. All patients had immunological markers, including ANA (100% of cases), dsDNA (47%). The therapy depends on disease severity: 100% patients were treated by corticosteroids, 94% used cytostatic treatment (47,05% azathioprine, cyclophosphamide – 35,2%, methotrexate, cyclosporine, mycophenolate mofetil had 5,8% equely). 76,5% recieved Plaquenil, 47,1% - IVIG. In 17,6% cases required Rituximab. Despite serious course of disease remission was achieved on the treatment and ANA inversion to negative was found in 9,1% cases.

**Conclusion:** SLE is rarely diagnosed among boys and, in spite of the severity of clinical manifestations the delay of diagnosis is more significant. The most common symptoms in male patients are hematological and articular signs. Frequent nonspecific constitutional disturbances among the first symptoms without adequate laboratory examination result in delay of timeous diagnosis. More than the half cases suffered by nephritis and quarter by central nervous system involvement. The severity of clinical presentations requires aggressive treatment strategy that leads to good results in most cases.

**Disclosure of Interest**

None Declared.

### P315 Clinical features associated with histological pattern in childhood lupus nephritis

#### Valentina Litta-Modignani, Francesco Baldo, Carlo Virginio Agostoni, Marisa Giani, Giovanni Montini, Giovanni Filocamo

##### Fondazione IRCCS Ca’ Granda Ospedale Maggiore Policlinico, Milan, Italy

###### **Correspondence:** Valentina Litta-Modignani

**Introduction:** Systemic lupus erythematosus (SLE) is a clinically diverse, autoimmune disease characterized by the development of autoantibodies, multi-organ involvement, and a chronic disease course.

Renal involvement is one of the most common manifestations of SLE, and lupus nephritis (LN) has a higher prevalence in childhood-onset SLE (cSLE), occurring in up to 60% of patients during the disease course.

The outcome in cSLE with nephritis is worse than in those without it, and the initial presentation at onset has been reported as predictive for renal involvement.

**Objectives:** To analyze the clinical presentation at onset of cSLE and to identify clinical features associated with different histological findings at kidney biopsy.

**Methods:** We retrospectively analyzed the records of patients with LN followed from 1994 to 2017 at Ospedale Maggiore Policlinico of Milan, who underwent a renal biopsy in the first 2 years since the disease onset.

The Systemic Lupus Erythematosus Disease Activity Index (SLEDAI) was used for measuring disease activity at onset of cSLE.

The WHO classification of LN was used to grade histological findings. We considered renal involvement mild if the histological class was II or III (16 patients), and severe if it was IV or V (14 patients).

Continuous variables were compared between the two groups with the Student’s t-test, whereas categorical variables were compared with the chi-squared test. In all analyses, P < 0.05 was taken to indicate statistical significance.

**Results:** Among 45 children with LN who underwent a renal biopsy in the first 2 years since the disease onset, 15 were excluded because of incomplete data, thus a total of 30 patients was finally enrolled.

Among the 30 patients selected, the distribution of histological class was: Class II: 5 patients (16.7%), Class III: 11 patients (36.7%), Class IV: 12 patients (40%) and Class V: 2 patients (6.7%).

The mean disease duration since disease onset until renal biopsy was 10 and 19 months for mild and severe involvement respectively. The mean SLEDAI score at onset was 31 for the mild subgroup and 25 for the severe subgroup. Neurological manifestations were more frequent in children with higher histological classes at renal biopsy.

Among the activity indices at onset, hematuria was more frequent in the patients in the mild group than in those in severe group (P = 0.02), as well as pyuria (P = 0.01).

Patients with the development of severe renal involvement presented more frequently with hypertension, compared to those who developed a mild involvement (35.7% vs 18.7%, respectively, P = 0.294).

Other demographic and clinical characteristics at disease onset are shown in Table 35.

F/M: female to male ratio.

Neurological manifestations: Seizure, Cerebrovascular accidents, and Organic brain syndrome.

Hypertension (PAs > 140, PAd > 90)

**Conclusion:** As expected, the disease duration is higher in the group with more severe histological findings, even if it is not statistically significant in our population, probably due to the small sample size and high dispersion of the results.

Pyuria and hematuria were most frequently observed among the group with mild involvement at kidney biopsy, whereas hypertension and neurological manifestations are more represented in the severe group.

The SLEDAI score at onset was higher in children with mild kidney involvement.

Further data collection is still ongoing on prospective cases.

**Disclosure of Interest**

None Declared.

**Table 35 (Abstract P315).** Demographic and clinical characteristics at disease onset

	*Severe (n = 14)*	*Mild (n = 16)*	P value
Age at onset (years)	10,3	12,4	0,4
Disease duration (months)	19,9	10	0,402
mean SLEDAI	25,6	31,1	0,281
Neurological manifestation	6	1	**0.018**
Lupus headache	3	5	0,474
mean UPr/UCr	1,33	1,07	0,631
Hematuria	9	14	**0,023**
Pyuria	8	15	**0,018**
Hypertension	5	3	0,294

### P316 Evaluation of HMGB1 and its various post-translational forms in JSLE

#### Angela Midgley^1^, Daniel Antoin^2^, Michael Beresford^1^

##### ^1^Department of Women’s and Children’s Health, University of Liverpool, Liverpool, UK; ^2^Department of Molecular and Clinical Pharmacology, University of Liverpool, Liverpool, UK

###### **Correspondence:** Angela Midgley

**Introduction:** High-mobility group box 1 (HMGB1) is a highly conserved ubiquitous nuclear protein with key functions including regulation of DNA binding, transcription, repair and recombination. Extracellular HMGB1 elicits chemotaxis cytokine induction and has pro-inflammatory effects. The active release and the extracellular functions of HMGB1 are currently believed to be regulated by specific post-translational modifications (PTM) of HMGB1. There are very limited data investigating the different HMGB1 isotypes in chronic inflammatory disease.

**Objectives:** To measure HMGB1 PTM in serum from patients with Juvenile-onset Systemic Lupus Erythematosus (JSLE), Juvenile Idiopathic Arthritis (JIA) and paediatric non inflammatory healthy controls.

**Methods:** HMGB1 from JSLE (n = 39), JIA (n = 6) and paediatric healthy control (n = 23) serums were isolated by immuno-precipitation. Different isoforms of HMGB1 were analysed by liquid chromatography using electrospray tandem mass spectrometry. Samples were also split depending on their BILAG score for Renal disease to see if HMGB1 levels observed correlated with disease activity.

**Results:** Significantly higher levels of total HMGB1 (ng/ml; mean, SEM) was observed in JSLE (7.91ng/ml, ± 0.89 patients compared to healthy control (0.917 ± 0.09. p < 0.0001) but not JIA (6.63 ± 1.04 p = 0.8), whilst acetyl HMGB1 levels were higher in JSLE (5.76ng/ml ±0.85 patients compared to both JIA (0.94 ± 0.37 p < 0.0001) and healthy controls (0.04 ± 0.0067, p < 0.0001). Monomethylation of lysine 43 (K43), a proposed neutrophil-specific PTM, was also observed in all JSLE patients serum (6.17ng/ml ± 0.99, p < 0.0001) but not the healthy control group. When samples were split depending on their Renal domain BILAG score, higher total acetyl and mono methyl HMGB1 (ng/ml: mean, SEM) were measured in active disease (BILAG score A&B: Total, 10.94 ± 0.95; acetyl, 8.8 ± 0.88; and mono methyl 9.43 ± 1.06, compared to inactive disease (BILAG score D&E: Total, 2.5 ± 0.22, p < 0.05; acetyl, 0.33 ± 0.09, p < 0.05; and mono methyl 0.019 ± 0.002, p < 0.05).

**Conclusion:** Hyperacetylation of HMGB1 was observed in all JSLE patients, indicating active release of HMGB1. Monomethylation of lysine 43 (K43) was also observed in all JSLE patients serum but not controls, implying that neutrophils are a source of the released HMGB1 in JSLE patients. Fully reduced HMGB1 was observed in all patient groups whereas disulphide HMGB1 was only observed in the JIA and JSLE patients suggesting that in chronic inflammatory disease HMGB1 can act as both a chemotactic and a driver of cytokine production. Measuring PTM of HMGB1 could potentially provide a useful biomarker for JSLE and/or therapeutic target.

**Disclosure of Interest**

None Declared.

### P317 Aquaporin-4 antibodies in patients with juvenile systemic lupus erythematosus

#### Elena Moraitis^1,2^, Muthana Al-Obaidi^1^, Kshitij Mankad^3,4^, Yael Hacohen^4,5^, Cheryl Hemingway^4,5^, Despina Eleftheriou^1,2^

##### ^1^Rheumatology, Great Ormond Street Hospital NHS Foundation Trust, London, UK; ^2^Infection, Inflammation and Rheumatology, UCL GOS Institute of Child Health, London, UK; ^3^Radiology, Great Ormond Street Hospital NHS Foundation Trust, London, UK; ^4^UCL GOS Institute of Child Health, London, UK; ^5^Neurology, Great Ormond Street Hospital NHS Foundation Trust, London, UK

###### **Correspondence:** Eleftheriou

**Introduction:** Neuromyelitis optica spectrum disorders (NMOSD) encompasse a group of inflammatory disorders of the central nervous system which often present with a relapsing course involving typically the optic nerve and the spinal cord, but with possible involvement of the brain. They are typically associated with a disease-specific serum NMO-IgG antibody that selectively binds aquaporin-4 (AQP4), and have been shown to be highly pathogenic.

The presence of anti-AQP4 antibodies has been described in the context of systemic autoimmune diseases such as adult onset systemic lupus erythematosus; however this is the first study to focus on the characteristics of this subgroup of patients in the paediatric population.

**Objectives:** The aim of the study is to describe the clinical characteristics, imaging, diagnostic features, treatment and outcome in patients with juvenile SLE (jSLE) and anti-AQP4 antibody positivity.

**Methods:** We conducted a retrospective case review of children presenting to the Rheumatology and Neurology services at Great Ormond Street Hospital for Children NHS Foundation Trust between 2002 and 2016 with jSLE as per 1997 American College of Rheumatology revised criteria for SLE classification or newer SLICC classification criteria and anti-AQP4 antibody positivity.

**Results:** A total of 4 patients median age 10.5 (range 9-12 years), 3 females and 1 male were identified. The median length of follow up was 5.5 years (range 2-9 years). All patients were positive for anti-AQP4 antibodies during the disease course and remained positive on repeat measurements during follow up. Two (50%) of the patients in our cohort had a diagnosis of SLE and consistently positive NMO-IgG serology without overt clinical and radiological NMO at a follow up of 2-3 years. The other two patients fulfilled the 2015 NMOSD diagnostic criteria, with AQP4 antibodies positivity and optic neuritis and/or transverse myelitis. A total of 3/4 patients had systemic involvement whilst 1/4 had primarily a neurological presentation. Interestingly, this latter patient presented at the age of 10 years with unilateral optic neuritis and no other features, while broader investigations revealed raised antinuclear and anti-dsDNA antibodies. Over the next five years from the original presentation the symptoms fluctuated with a relapsing-remitting course including bilateral ocular disease and transverse myelitis. At eight years post onset she was relapse free on medication for three years but with residual visual impairment.

Three patients had abnormal brain imaging: one patient presented changes consistent with brain abnormalities previously described in the context of NMOSD, and two patients had non-specific brain MRI findings previously described in patients with SLE.

One patient had concomitant onset of SLE and NMOSD, with monoparesis and inflammatory lesion of cervical cord C3 to T1. During a 9 years follow up she presented longitudinal extensive transverse myelitis, multiple relapses, optic atrophy; she had no abnormalities on brain imaging.

**Conclusion:** We describe the first paediatric cohort of jSLE with anti-AQP4 antibodies positivity. Although limited by the small sample size and retrospective nature, our study possibly suggests that there is an association between jSLE and NMOSD. Therefore patients with jSLE should be screened for presence of AQP4 antibodies to help identifying children at risk prior to the development of clinical symptoms.

**Disclosure of Interest**

None Declared.

### P318 comparison of the ACR and SLICC classification criteria in borderline (aged 16-18 years) juvenile-onset systemic lupus erythematosus patients

#### Cristina Pamfil^1,2^, Laura Damian^3,4^, Maria Deac^3^, Ioana Felea^2,3^, Siao-Pin Simon^1,3^, Laura Muntean^1,5^, Iulia Szabo^5^, Elisabeta Candrea^6^, Gabriel Gusetu^7^, Simona Rednic^1,5,8^

##### ^1^Rheumatology, Iuliu Hatieganu University of Medicine and Pharmacy, Cluj-Napoca, Romania; ^2^Rheumatology, Centre for Rare Musculoskeletal Autoimmune and Autoinflammatory Diseases, Cluj-Napoca, Romania; ^3^Rheumatology, Emergency Clinical County Hospital, Cluj-Napoca, Romania; ^4^Rheumatology, Centre for Rare Musculoskeletal Autoimmune and Autoinflammatory Diseases, Cluj-Napoca, Romania; ^5^Emergency Clinical County Hospital, Cluj-Napoca, Romania; ^6^Dermatology, Iuliu Hatieganu University of Medicine and Pharmacy, Cluj-Napoca, Romania ^7^Cardiology, Iuliu Hatieganu University of Medicine and Pharmacy, Cluj-Napoca, Romania; ^8^Centre for Rare Musculoskeletal Autoimmune and Autoinflammatory Diseases, Cluj-Napoca, Romania

###### **Correspondence:** Cristina Pamfil

**Introduction:** The Systemic Lupus International Collaborating Clinics (SLICC) group issued revised classification criteria for systemic lupus erythematosus (SLICC-2012 criteria).

**Objectives:** We aimed to compare these criteria with the 1997 American College of Rheumatology (ACR-1997 criteria) classification criteria in a cohort of borderline juvenile-onset systemic lupus erythematosus (JSLE) patients, aged 16-18, referred to a tertiary adult rheumatology center.

**Methods:** We assessed both classification criteria within 1 year from diagnosis in 12 JSLE patients.

**Results:** All patients were Caucasian and 84% were female; mean (standard deviation [SD]) age was 16 (*+*1.1). The majority of patients had lupus nephritis (66%) and/or neuropsychiatric involvement (25%). The SLICC-2012 was more sensitive than ACR-1997 at diagnosis (91.6% versus 75%); patients met a higher mean number of SLICC-2012 than ACR-1997 criteria (6.4 [+2.8] *vs*. 5.8 [+2,4]). Most patients meeting the SLICC-2012 criteria, and not the ACR-1997 fulfilled more than one additional criterion of the SLICC-2012. A proportion of patients (16.6%) exhibited organ manifestations (orhiepididimitis, lupus hepatitis) not included in the classification criteria, however all of cases had other significant organ involvement and met at least 4 other criteria in both evaluation systems.

**Conclusion:** The SLICC-2012 was better able to classify patients with JSLE than the ACR-1997 within 1 year from diagnosis in JSLE patients aged 16-18. SLICC-2012 could be considered a reliable tool for the classification of borderline JSLE patients in observational studies and clinical trials.

**Funding:** PN-II-RU-TE-2014-4-2708

**Disclosure of Interest**

None Declared.

### P319 Capturing the initial profile of the pediatric SLE in greek patients

#### Polyxeni Pratsidou-Gertsi^1^, Maria Trachana^1^, Olga Vougiouka^2^, Erato Atsali^3^, Florence Kanakoudi-Tsakalidou^1^

##### ^1^Pediatric Immunology & Rheumatology Referral Center, First Department of Pediatrics, Aristotle University, Hippokration General Hospital, Thessaloniki, Greece; ^2^Second Department of Pediatrics, P. & A. Kyriakou Children’s Hospital, University of Athens, Athens, Greece; ^3^Third Department of Pediatrics, Attikon University Hospital, Athens, Greece

###### **Correspondence:** Polyxeni Pratsidou-Gertsi

**Introduction:** Several metrics regarding the disease status assessment have been used in adult SLE as primary endpoints to evaluate the disease activity and course. Such a quantitative policy is still accumulating for Caucasian homogenous pediatric SLE (pSLE) populations.

**Objectives:** To depict the onset characteristics and the first year disease course in a Caucasian Southern European population with pSLE.

**Methods:** pSLE pts fulfilling the ACR or SLICC classification, with a disease onset < 18yrs and a 12mo follow-up in 3 Outpatient Pediatric Rheumatology Clinics were enrolled. The disease activity and course was assessed according to SLEDAI-2K, SLEDAI Flare Index and the Lupus Low Disease Activity Score (LLDAS).

**Results:** 52 pts (M:F 12:40), aged 12.09 ± 2.64 yrs were studied; 11/52 were < 10yrs at diagnosis. 41 were from Thessaloniki’s Referral Centre, and the rest from Athens (Hospitals (P&A Kyriakou: 7 and Attikon: 4, respectively). The lag-time from onset to diagnosis was 5.28 ± 10.18 mo (median 2.28). The predominant system involved was the skin (45/52), presenting either as a malar rash (n = 45), or photosensitivity (n = 31), followed by mucous ulcers (n = 13), hair loss (n = 10) and skin ulcers (n = 1). A musculoskeletal involvement was detected in 34/52, as sole arthritis (n = 34) or with myositis (n = 4/34). Systemic manifestations were evident in 32/52, as fever (n = 32), malaise (n = 31), lymphadenopathy (n = 19), hepatomegaly (n = 17), serositis (n = 16), splenomegaly (n = 12) or weight loss (n = 12). Hematologic findings were the 3^rd^ prevailing system (n = 31/52), mainly as autoimmune hemolytic anemia (n = 27). The initial renal involvement was in 20/52; 17/20 underwent a diagnostic biopsy and 15/20 had a grade III to V nephritis, but none with chronicity indices. Vascular thromboses had 6/52 and CNS manifestations 4/52. Neuropsychiatric SLE presented either as lupus headache (n = 2, escorted by psychosis + amnesia in 1/2), or chorioathetosis in other 2 (in 1/2 accompanied by aphasia and cognitive disorders). All pts were ANA positive, while anti-DNA were detected in 49/52, antiSm, anticardiolipin autoantibodies in 13 and 16, respectively, and low complement (C3 or C4) in 46/52 pts. The initial SLEDAI-2K was 28 ± 10.8 (0-105).The induction regimen was steroids (49/52, in 10 as pulses) plus hydroxychloquine (35/52), while immunosuppressants received 26, mainly cyclophosphamide (n = 11), followed by azathioprine (n = 8) and mycophenolate (n = 5). 2 pts with arthritis received methotrexate and sulfasalazine. 21 received other drugs (anticoagulants, IVIG, antihypertensives) and 2 received rituximab (hemolytic anemia, catastrophic antiphospholipid syndrome). At 12 mo, absence of flares had 29/52 and Lupus Low Disease Activity, 6/52.

**Conclusion:** These findings indicate that early diagnosis and quantitative assessment at baseline as well as at 12-mo post treatment, can capture the severity of pSLE and evaluate the therapeutic choices that lead to early disease taming.

**Disclosure of Interest**

None Declared.

### P320 Epidemiology of lupus in Estonian children in 2012-2016

#### Chris Pruunsild^1^, Sirje Tarraste^2^, Jaanika Ilisson^3^

##### ^1^Children’s Clinic, Institute of Clinical Medicine, Faculty of Medicine, Tartu University, Tartu, Estonia; ^2^Tallinn Children’s Hospital, Tallinn, Estonia; ^3^Children’s Clinic, Tartu University Hospital, Tartu, Estonia

###### **Correspondence:** Chris Pruunsild

**Introduction:** Nearly one fifth of lupus’ cases has the start in childhood. Juvenile systemic lupus erythematosus (jSLE) is a serious disease with multi-organ damage and variable clinical presentation among which nephritis develops in over 95% of cases. Although the era of biologics has given new promising options in the handling of jSLE, the control of the disease process remains still often a challenge. Neonatal form is a distinct entity under the umbrella of pediatric lupus, demanding a special follow-up by cardiologists.

**Objectives:** To find the incidence rate (IR) of lupus (neonatal, cutaneous and systemic) in children in Estonia during 2012-2016 and the point prevalence (PP) on Dec 31th, 2016. To describe the clinical presentation, presence of autoantibodies and treatment choices in the study group.

**Methods:** Retrospective analysis of the electronic records of the two tertiary pediatric centres in Estonia. For calculation of IR and PP, the data of the Statistics Estonia (www.stat.ee) were used.

**Results:** During years 2012-2016 twelve new cases of lupus (among them five with cutaneous form) were diagnosed; in addition five neonatal lupuses (NL) were described. In the systemic + cutaneous group (12), there was only one boy; the neonatal group consisted of two girls and three boys. The mean annual IR for systemic and cutaneous lupus was 3.0 and 1.5 per 100 000 children aged 0-19 years, respectively. Per 10000 live births the mean IR of NL was 3.6. The PP on Dec 31th, 2016 was 4.8 per 100 000 children aged 0-19 years for jSLE, and 1.8 for CL. Per 10 000 children aged 0-1 year, the PP of NL was 5.0. All the jSLE and CL cases had their start after 7th birthday. According to the ACR criteria, the systemic cases presented with at least four criteria during the disease course, of which antinuclear antibody (ANA) positivity, arthritis, hematological findings, ds-DNA positivity, and photosensitivity being the most often described (92%, 67%, 67%, 58%, and 58%, respectively). Kidney biopsia had been performed in 4 cases – II-III class histological changes mostly described, “full-house” fenomen in one case. All the cutaneous forms had been histologically proven. From the seven NL cases three presented with rash and three with congenital atrio-ventricular blockade; in two of the last the pacemaker has been placed. All the NL cases were anti-SSA/Ro positive. Hydroxycholoroquine was in the treatment scheme in all the 13 jSLE patients in the prevalence group. Six patients were on methotrexate and five on azathioprine. 69% (9/13) of jSLE patients were on regular peroral glucocorticosteroid treatment, four on mycophenolate mophetil, one rituximab and one had received cyclophosphamide pulse therapy.

**Conclusion:** The mean annual incidence rate for the years 2012-2016 and the point prevalence rate on Dec 31th, 2016 for systemic, cutaneous and neonatal lupus were calculated for the overview of the epidemiology of pediatric lupus in Estonian children. Systemic JLE with it’s very heterogeneous presentation and the criteria fulfilled over several years in some cases, still remains a diagnostic and therapeutic challenge. Neonatal lupus patients with bradycardia need careful monitoring by pediatric cardiologists.

**Disclosure of Interest**

None Declared.

### P321 Screening fabry disease in juvenile systemic lupus erythematosus

#### Ertugrul Kiykim^1^, Sezgin Sahin^2^, Tanyel Zubarioglu^1^, Kenan Barut^2^, Amra Adrovic^2^, Mehmet Serif Cansever^1^, Cigdem Aktuglu-Zeybek^1^, Ozgur Kasapcopur^2^

##### ^1^Pediatric Nutrition and Metabolism, Istanbul University, Cerrahpasa Medical School, Istanbul, Turkey; ^2^Pediatric Rheumatology, Istanbul University, Cerrahpasa Medical School, Istanbul, Turkey

###### **Correspondence:** Sezgin Sahin

**Introduction:** Fabry disease is an X-linked lysosomal storage disorder that share similar organ involvement pattern with systemic lupus erythematosus (SLE). Mutations in GLA gene decrease the activity of lysosomal alpha galactosidase enzyme and responsible from the clinical manifestations. Decreased enzyme activity results with accumulation of a ganglioside; globotriaosylceramide inside of the endothelial cells and this accumulation impairs the normal functioning of these cells. Although previously reported as an X-linked recessive disease, currently Fabry disease is classified under the group of X-linked diseases owing to presence of clinical manifestations also in females. A more quiescent and nonspecific disease course are encountered particularly in females and children. Hence, delay in diagnosis and treatment is frequently observed. Presence of autoantibodies in Fabry disease and co-occurence of Fabry disease and SLE are reported previously in various case reports.

**Objectives:** In this study, we aimed to screen Fabry disease in all ANA positive juvenile SLE patients that were diagnosed according to ACR 1997 criteria. All subjects were also interrogated in terms of clinical manifestations of Fabry disease.

**Methods:** From January 2016 to June 2016, 76 ANA positive juvenile SLE patients were enrolled consecutively in the study. All patients had met the 1997 revised criteria of American College of Rheumatology for classification of SLE. All of the patients or parents gave written consent for participation. Since the majority of the patients were female, initially GLA gene was searched for mutations causing Fabry disease. Mutation positive subjects were further evaluated for storage of globotriaosylceramide in lysosomes (lyso*Gb3).

**Results:** Of the 76 subjects, 12 were male and 64 of them were female. While the mean onset of the disease was 11.6 ± 3.6 years, the mean duration of disease was 4.4 ± 2.9 years. Parental consanguinity was present in 25% (n = 19) of the patients. Fourteen subjects have been reporting burning sensation in hands and feet; two patients hypohidrosis and 11 patients gastrointestinal manifestations. There were no cornea verticillata and angiokeratomas in any of the patients. A heterozygous p.D313Y has been found in 2 female subjects. These subjects were further investigated for lyso*Gb3 levels in dry blood samples. Lyso*Gb3 levels were found to be also normal in these two subjects and we have excluded Fabry disease.

**Conclusion:** Fabry disease may occasionally manifests with nonspecific features particularly in females. Hence, the diagnosis is delayed and the patients receive treatments due to various misdiagnoses. Although coexistence of SLE and Fabry disease previously has been reported in case reports, a systematic screening of juvenile SLE for Fabry disease has not been performed up to date. However, we have not detected any Fabry disease in our juvenile SLE cohort. In conclusion, we do not suggest screening Fabry disease in patients with SLE.

**Disclosure of Interest**

None Declared.

### P322 Are prematurity and environmental factors determinants for developing childhood-onset systemic lupus erythematousus?

#### Paola G. Conde, Luis C. Farhat, Alfésio L. Braga, Adriana E. Sallum, Sylvia C. Farhat, Clovis A. Silva

##### Pediatric Rheumatology Division, Children’s Institute, Hospital Das Clinicas HCFMUSP, Faculdade De Medicina, Universidade De Sao Paulo, São Paulo, Brazil

###### **Correspondence:** Adriana E. Sallum

**Introduction:** It has been observed during follow-up of childhood-onset systemic lupus erythematosus (cSLE) patients that exposure to inhalable pollutants may increase disease activity. However, to our knowledge there is no study assessing environmental determinants during pregnancy and after birth until diagnosis as risk factors for developing cSLE.

**Objectives:** To evaluate the influence of exposure to air pollutants and inhalable environmental elements during pregnancy and after birth until cSLE diagnosis.

**Methods:** This case-control study comprised 30 cSLE patients and 86 healthy controls living in the Sao Paulo metropolitan area. A structured and reliable questionnaire (kappa index for test-retest was 0.78) assessed demographic data, gestational and perinatal related-factors, and exposure to inhalable elements during pregnancy and after birth (occupational exposure to inhalable particles and/or volatile vapor, and/or tobacco, as well as, the presence of industrial activities or gas stations near the home/work/daycare/school). Tropospheric pollutants included: particulate matter (PM_10_), sulphur dioxide (SO_2_), nitrogen dioxide (NO_2_), ozone (O_3_) and carbon monoxide (CO).

**Results:** The median current age was similar between cSLE patients and healthy controls [16.0 (5-21) vs. 15.0 (4-21) years, p = 0.32], likewise the frequency of female gender (87% vs. 78%, p = 0.43). The frequencies of prematurity (30% vs. 6%, p = 0.001), maternal occupational exposure during pregnancy (59% vs. 12%, p < 0.001), exposure to volatile vapor (48% vs. 8%, p < 0.001) and fetal smoking (maternal and/or secondhand) (37 vs. 19%, p = 0.008) were significantly higher in cSLE patients compared to controls. In a multivariate analysis regarding the gestation period, maternal occupational exposure (OR 13.5, 95%CI 2.5-72.4, p = 0.002), fetal smoking (OR 8.6, 95%CI 1.6-47, p = 0.013) and prematurity (OR 15.8, 95%CI 1.9-135.3, p = 0.012) remained risk factors for cSLE development. Furthermore, exposure to secondhand smoking during pregnancy and after birth (OR 5, 95%CI 1.2-20.1, p = 0.002) was also a risk factor for cSLE development.

**Conclusion:** Prematurity and environmental factors were risk factors for developing cSLE. Further longitudinal multicenter studies in large urban areas are required to a better understanding of these associations.

**Disclosure of Interest**

P. Conde: None Declared, L. Farhat: None Declared, A. Braga: None Declared, A. Sallum: None Declared, S. Farhat: None Declared, C. Silva Grant/Research Support from: Conselho Nacional de Desenvolvimento Científico e Tecnológico (CNPq 472155/2012-1).

### P323 Capillaroscopy findings in childhood-onset systemic lupus erythematosus, a Dutch experience of 22 children and adolescents

#### Dieneke Schonenberg-Meinema^1^, Merlijn vd Berg^2^, Amara Nassar-Sheikh-Rashid^2^, Godelieve de Bree^3^, Liesbeth Hak ^4^, Marieke van Onna^4^, Karin Melsens^5^, Maurizio Cutolo^6^, Taco Kuijpers^2^, Vanessa Smith^5,7^

##### ^1^Department of Pediatric Hematology, Immunology, Rheumatology and Infectious Diseases, Emma Children’s Hospital, Academical Medical Center (AMC), Amsterdam, Netherlands; ^2^Department of Pediatric Hematology, Immunology, Rheumatology and Infectious Diseases, Emma Children’s Hospital, Academical Medical Center, Amsterdam, Netherlands; ^3^Department of Infectious Diseases, Academical medical center, Amsterdam, Netherlands; ^4^Department of Clinical Immunology and Rheumatology, Academical medical center, Amsterdam, Netherlands; ^5^Department of Rheumatology, Ghent University Hospital, Ghent, Belgium; ^6^Research Laboratory and Academic Unit of Clinical Rheumatology, University of Genova, Genova, Italy; ^7^Faculty of Internal Medicine, Ghent University, Ghent, Belgium

###### **Correspondence:** Dieneke Schonenberg-Meinema

**Introduction:** Capillaroscopy findings can be qualitatively described as: normal, microangiopathy (non-specific abnormalities) or scleroderma pattern. Capillary abnormalities, described in varying prevalence in patients with systemic lupus erythematosus (SLE), are mainly described as microangiopathy

**Objectives:** To describe capillary characteristics in a cross-sectional tertiairy cohort of patients with childhood-onset SLE (cSLE) by quantitative and qualitative assessment

**Methods:** Nailfold videocapillaroscopy (NVC) was performed in cSLE-patients (onset < 18 years) with a x200 magnification lens (Optilia). All fingers except the thumb were examined with 4 images per finger. The following capillaroscopic characteristics were evaluated per millimeter: density (compared to mean density known for age, sex and ethnicity), number of abnormal shapes (as defined by the EULAR study group on microcirculation in Rheumatic Diseases), giant capillaries (defined as apical diameter >50 mcm), maximum apical diameter (dilatations defined as apical diameter 20-50mcm) and microbleedings (categorized by large hemorrhages and small multiple point-shaped hemorrhages surrounding the capillary loop)

**Results:** 4607 capillaries from 22 patients with cSLE, were analyzed. 18.2% (n = 4) showed a scleroderma-pattern, all other patients (81.8%, n = 18) showed signs of microangiopathy. Details from quantitative assessment are shown in Table 36. A mean density below 2.5 percentile for age was seen in 45.5% (n = 10), multiple (>8 per patient) abnormal shapes in 59.1% (n = 13), apical dilatations in 90.9% (n = 20). Total count of large/small point shaped hemorrhages was 114/948, with a mean of 0.2/1.6 per analyzed image/per patient. These were detected in resp. 82.8/90.9% of patients (n = 18/20)

**Conclusion:** In this pilot (n = 22) of cSLE patients, all showed capillary abnormalities. The majority (n = 18, 81.8%) was qualitatively classified as microangiopathy. Quantitatively, the most striking abnormality was a specific subtype of hemorrhage (small point-shaped bleedings surrounding the capillary), detected in 90.9% (n = 20) of patients. These have never been described in adults nor in children with SLE. Prospective longitudinal cohort studies in children through the EULAR study group on microcirculation in Rheumatic diseases will elucidate whether specific findings can be found in (childhood-onset) SLE

**Disclosure of Interest**

None Declared.

**Table 36 (Abstract P323).** Quantitative findings of NVC in cSLE, n = 22. IQR = interquartile range

	Total	Median (IQR 25-75)
Total capillaries	4607	215 (184.5-242.8)
Density in capillary/mm		7.1 (6-7.7)
Total abnormal shapes	369	14.5 (7-24.5)
Mean (of 8 fingers) of max diameter in mcm/capillary		27.6 (24.5-31)
Maximum diameter (per patient) in mcm/capillary		39.5 (35-47)
Total giant capillaries	14 (mean 0.6, SD 2.4)	
Total micro-hemorrhages:	1062	
-Large micro-hemorrhages	114	2 (1-6.3)
-Small point-shaped hemorrhages surrounding the capillary	948	35 (10.3-62.5)

### P324 Ficolin h in pediatric SLE cohort from North India

#### Anil K. BN, Madhubala Sharma, Amit Rawat, Anju Gupta, Surjit Singh

##### Allergy immunology unit, APC, PGIMER, Chandigarh, India

###### **Correspondence:** Madhubala Sharma

**Introduction:** Studies have been performed in adult SLE patients, but there is a dearth of data on the functional assessment of Ficolin pathway of complement in Paediatric SLE patients. The present study is being entailed with a view to delineating the role of Ficolin pathway of SLE and estimate Ficolin H levels in children with Paediatric-onset SLE. The characterization of this expression profile also has pathogenetic and potential therapeutic implications.

**Objectives:** To estimate Ficolin H in pediatric SLE cohort from India

**Methods:** Thirty 33 consecutive children diagnosed with SLE and fulfilling the requisite inclusion criteria were enrolled for the study. Fourteen of these 33 children enrolled in the study group were in an active state of disease. Ficolin-H (Ficolin 3) was estimated in the serum of the test and control subjects using a commercial enzyme-linked immunosorbent assay kit.

**Results:** The ficolin H levels were elevated in study subjects compared to controls. Mean ficolin H levels in study group was 248μg/ml compared to 184 μg/ml in controls. No correlation was observed between disease activity as measured by SLEDAI score and Ficolin H levels. However, there was a negative correlation between ds DNA levels and ficolin H levels both in active and inactive disease.

**Conclusion:** No correlation was found between ficolin H levels and disease activity as determined using SLEDAI scores in pSLE. A negative correlation was observed between anti-dsDNA levels and Ficolin H levels. However, this correlation was not found to be statistically significant. Further large studies would be needed to confirm any association between anti-dsDNA and Ficolin H.

**Disclosure of Interest**

None Declared.

### P325 Paediatric lupus nephritis - a single-centre experience of over 10 years

#### Claudia Sirbe^1^, Iulia E. Szabo^2^, Bogdan Bulata^1^, Mihaela Spirchez^1,3^, Dan Delean^1^, Simona Rednic^2,3^

##### ^1^Department of Paediatrics, Emergency Clinical County Hospital for Children, Cluj Napoca, Romania; ^2^Department of Rheumatology, Emergency Clinical County Hospital, Cluj Napoca, Romania; ^3^Department of Rheumatology, University of Medicine and Pharmacy “Iuliu Hatieganu” Cluj-Napoca, Romania, Cluj Napoca, Romania

###### **Correspondence:** Claudia Sirbe

**Introduction:** Kidney involvement is a common manifestation in systemic lupus erythematosus (SLE), the incidence being 50% to 75% of childhood-onset SLE (cSLE) patients. Lupus nephritis (LN) encompasses a clinical spectrum from microscopic glomerular haematuria, cellular casts and minimal proteinuria to a myriad of life-threatening disorders.

**Objectives:** The aim of this study was to assess the clinicopathological characteristics and treatment outcomes in children with lupus nephritis in a paediatric nephrology department for 13 years.

**Methods:** This study was based on the lupus nephritis cohort, which included 19 hospitalised patients from the Department of Nephrology, Emergency Clinical County Hospital for Children Cluj Napoca, Romania, from 2003 till 2016. In this cohort were included detailed demographic data, ethnicity, age of LN onset, time between onset and diagnosis, clinical laboratory results, renal biopsy result and medication information.

Eligible patients were diagnosed with childhood-onset lupus nephritis prior to 17 years of age, using revised American College of Rheumatology (ACR) SLE classification criteria and SLICC criteria. The activity of disease was assessed according to SLE Disease Activity Index scale (SLEDAI). Renal biopsy was performed before the initial induction therapy in 13 patients and the results were classified using ISN/RPA. Partial and complete renal response were categorised based on EULAR/ERA-EDTA recommendations of LN.

**Results:** A total of 19 patients ages 5 to 17 years were evaluated in this analysis. The mean age of LN onset was 12.6 years and 3 boys (16%) were included, yielding a female-to-male ratio of 5 :1.

Patients were referred to our nephrology centre for further investigation of acute glomerulonephritis (47%) and treatment in cSLE with kidney involvement (32%). Timely diagnosis of paediatric SLE perceived an average delay from onset of 8.5 months. One-third of children (32%) had overt kidney disease at the time SLE was diagnosed.

All cohort participants had proteinuria, with nephrotic syndrome attributed to 6 patients (32%), 16 children (84%) had haematuria and hypertension was reported in 7 cases. Moreover, 8 patients developed acute kidney injury (42%), of which 3 cases (16%) progressed to ESRD and required renal replacement therapy with subsequent kidney transplantation (KTX).

Renal biopsy was performed in 13 patients and most commonly revealed LN class IV (46%) and class V (23%). LN could not be confirmed by kidney biopsy in 6 patients due to severe anaemia or increased risk of bleeding.

Extrarenal manifestations included: hepatic, cardiac, articular, cutaneous, haematological and nervous system involvement. Antiphospholipid syndrome (APS) was described in 6 children.

The induction therapy aimed to control disease activity, contained in most of the cases (79%) intravenous cyclophosphamide (CY), administered according to NIH regimen in addition to methylprednisolone (MP) pulses. Four patients were treated with Mycophenolate (MMF) and one child with Cyclosporine, additional to MP pulses.

Overall, 16 (84%) patients had a response to induction therapy, while 3 cases (16%) were treatment resistant. Among responders, complete response (47%) was encountered more often than partial response (37%).

Furthermore, subsequent immunosuppression treatment included most commonly (74%) MMF and low-dose glucocorticoids (GC). Meanwhile, calcineurin inhibitors were considered in 3 cases and azathioprine in 2 children. Of the 16 patients who responded to aforementioned therapy, 9 (47%) experienced one or two relapses within 5 years of diagnosis.

**Conclusion:** Although significant improvement has been accomplished in overall prognosis, 10-30% of patients with proliferative glomerulonephritis develop end stage renal disease (ESRD). Despite worldwide awareness, LN is associated with an important morbidity and mortality, with a 10-year survival rate still lower than that in healthy populations.

**Disclosure of Interest**

None Declared.

### P326 Long-term quantitative assessment of the pediatric systemic lupus erythematosus gravity.factors that affect the disease outcome

#### Maria Trachana, Polyxeni Pratsidou-Gertsi, Florence Kanakoudi-Tsakalidou, Fotis Papachristou

##### First Department of Pediatrics, Aristotle University Thessaloniki, Pediatric Immunology and Rheumatology Referral Center, Ippokration Hospital, Thessaloniki, Greece

###### **Correspondence:** Maria Trachana

**Introduction:** The contemporary assessment of pSLE severity in several time points is based not only on the classic clinical/laboratory criteria, but also on the application of up-to-date “assessment tools” that measure the disease activity and cumulative damage. This objective assessment determines the appropriate therapeutic approach for the optimal disease outcome.

**Objectives:** The longitudinal quantitative evaluation of the “disease state” in Caucasian patients with pSLE and the identification of factors that could affect the long –term outcome.

**Methods:** 31 patients (M:F 9:22, with an age at diagnosis of 11.23 ± 2.69 years, were followed up for 5 (31/31) and 10 years (13/31), respectively. They were retrospectively assessed regarding the lag time from onset to diagnosis, the various organ/system involvement and annually quantitatively thereafter, by applying the contemporary tools for measuring the disease activity ((MDVAS score 0-3, SLEDAI-2K score 0-105, SELENA-SLEDAI) and the damage development (SLICC-DI). Moreover, the induction therapy, the subsequent regimen during the follow-up, the evaluation of the Quality of Care and the Health - Related Quality of Life were recorded.

**Results:** The mean lag time from the diseaseonset to diagnosis was 3.96 months. At baseline, a renal involvement was recorded in 13/31 patients, a CNS in 2/31 and cytopenias in 24/31 (mainly autoimmune hemolytic anemia, 21). Minimal mean disease activity (MDVAS) had 1/31, mild to moderate 20/31 (mean SLEDAI-2K score16.35) and moderate to severe 10/31 (SLEDAI-2K28.3).The induction therapy was steroids (31/31), hydroxychloroquine (19/31) and immumosuppressants (25/31). During the 5-year follow-up, seven additional patients developed renal involvement, 4 of the CNS, 1 the blood and during the 10-year follow-up, 1, 1 and 1, respectively. All patients received immunosuppressives and 7 targeted therapy (Retuximab). About ¾ of the patients complied with the Quality of Care indices. At the end of their follow-up, development of damage was recorded in 7/31 patientsevaluated as moderate to severe according to the SLICC –DI (score 1 και 2). No patient had psychiatric disorders.

**Conclusion:** These findings indicate that the early diagnosis and quantitative assessment of the pSLE severity overtime, are key determinants of the optimal treatment, targeting to a rapid regression of the disease activity and restriction of the cumulative damage development to a minority of patients.

**Disclosure of Interest**

None Declared.

### P327 Chylous ascites and podocytopathy as presenting features of systemic lupus erythematosus in a young child

**Abstract withdrawn**

## Treatment

### P328 Persistence with first biological agent and reasons for discontinuation in patients with juvenile idiopathic arthritis: data from the portuguese register, reuma.PT

#### Ana F. Mourão^1,2^, Mónica Eusébio^3^, José Melo-Gomes^4^, Filipa Oliveira-Ramos^5,6^, Fernando Martins^7^, Paula Estanqueiro^8^, Manuel Salgado^8^, Margarida Guedes^9^, Marta Conde^10^, Sónia Carvalho^11^, José Costa^12^, Iva Brito^13,14^, Cátia Duarte^15^, Carolina Furtado^16^, Ana Rodrigues^17^, Graça Sequeira^18^, Raquel Campanilho-Marques^4,6^, Jaime Branco^1,2^, Maria José Santos^19,20^, João Eurico Fonseca^5,20^, Helena Canhão^21^

##### ^1^Rheumatology, HOspital Egas Moniz, CHLO, EPE, Lisbon, Portugal; ^2^CEDOC, Nova Medical School, Lisbon, Portugal; ^3^Portuguese Society of Rheumatology, Lisbon, Portugal; ^4^Rheumatology, Portuguese Institute of Rheumatology, Lisbon, Portugal; ^5^Rheumatology, Lisbon Medical Academic Center, CAML, Lisbon, Portugal; ^6^Pediatric Rheumatology Unit, Hospital de Santa Maria, CHLN, Lisbon, Portugal; ^7^Rheumatology, Portuguese Society of Rheumatology, Lisbon, Portugal; ^8^Pediatrics, Centro Hospitalar Universitário de Coimbra, Coimbra, Portugal; ^9^Pediatrics, Centro Hospitalar do Porto, Hospital de Santo António, Porto, Portugal; ^10^Pediatrics, Hospital Dona Estefânia, Lisbon, Portugal; ^11^Pediatrics, Centro Hospitalar Médio-Ave, Famalicão, Vila Nova Famalicão, Portugal; ^12^Rheumatology, ULSAM - Hospital Conde de Bertiandos, Ponte de Lima, Portugal; ^13^Rheumatology, Centro Hospitalar São João, Porto, Portugal; ^14^Rheumatology, Faculdade de Medicina do Porto, Porto, Portugal; ^15^Rheumatology, Centro Hospitalar Universitário de Coimbra, Coimbra, Portugal; ^16^Rheumatology, Hospital do Divino Espírito Santo, Ponta Delgada, São Miguel, Portugal; ^17^Rheumatology, Hospital de Santo Espírito, Angra do Heroísmo, Terceira, Portugal; ^18^Rheumatology, Centro Hospitalar de Faro, Faro, Portugal; ^19^Rheumatology, Hospital Garcia de Orta, Almada, Portugal; ^20^Rheumatology Research Unit, IMM, Faculdade de Medicina de Lisboa, Lisbon, Portugal ^21^EpiDoc Unit, CEDOC, Nova Medical School, Lisbon, Portugal

###### **Correspondence:** Ana F. Mourão

**Introduction:** Persistence on medication mainly reflects both effectiveness and safety of a drug. Understanding the reasons to stop bDMARD in routine clinical practice can help to better define the efficacy and safety of biologic medications in children with juvenile idiopathic arthritis (JIA).

**Objectives:** To investigate persistence on treatment and the reasons for discontinuation of the first biological in patients with JIA.

**Methods:** Portuguese patients with JIA registered in Reuma.pt who started a bDMARD were analyzed. Persistence was defined as the length of time between treatment initiation and discontinuation of the first bDMARD. The mean time until discontinuation was calculated using Cox regression survival estimates and the reasons for discontinuation of the first bDMARD were registered.

**Results:** Of the 1724 JIA patients registered in Reuma.pt, 319 received biological therapy, 62% (198) female. The mean age at disease onset was 7.7 ± 4.8 years the mean time between the beginning of JIA and the first bDMARD was 8.2 ± 9.4 years. The mean disease duration was 13.7 ± 10.7 years and the mean age at the beginning of biological therapy was 15.8 ± 9.4 years. The distribution of JIA categories was: 19.1% polyarticular RF-negative, 17.2% enthesitis-related arthritis, 16.6% polyarticular RF positive (Poly RF +), 16% extended oligoarticular, 13.5% persistent oligoarticular (OligoP), 12% systemic JIA and 0.9% had undifferentiated arthritis. Considering the whole group, 53.2% have had extra − articular manifestations since the beginning of the disease. The mean time till treatment discontinuation of the first bDMARD (due to any cause) adjusted for gender, biological therapy, JIA subtype, age at the beginning of biological therapy, and disease duration until initiating first bDMARD was 44.8 ± 38.3 months (median: 34.7 months). Considering the categories of JIA, patients with Poli RF + had a longer stay in first biologic treatment (mean: 64.6 ± 48.6; median 50.4 months) while OligoP patients had a shorter duration of biological treatment (mean: 30.9 ± 29.8; median: 18.7 months). The biologic agent with longer persistence was etanercept (mean: 49.9 ± 40.9; median: 41.4 months) The major reasons for drug discontinuation were inefficacy (49.6%), remission (14.2%), adverse events (10.6%), patient decision (1.6%) and pregnancy planning (1.4%). In 22.7% the reason was not specified.

**Conclusion:** Our study shows that persistence with the first biological treatment in the overall population of JIA was almost four years, being longer in Poly RF+ patients. Almost half of the patients stopped their first biological agent due to lack of response, reinforcing the need for the existence of several treatment options fully studied in JIA.

**Disclosure of Interest**

None Declared.

### P329 The influence of biological therapy on the physical development of children with JIA

#### Olena A. Oshlyanska^1^, Ludmila I. Omelchenko^1^, Julia V. Biliavska^2^, Volodimir M. Kovalenko^2^

##### ^1^Connective Tissue Disorders in Children, State Institute of Pediatrics, Obstetrics and Gynecology, Academy of Medical Sciences of Ukraine, Kyiv, Ukraine ^2^Department of Myocardial Diseases and Clinical Rheumatology, NSC “M.D.Strazhesko Institute of Cardiology” MAS of Ukraine, Kyiv, Ukraine

###### **Correspondence:** Olena A. Oshlyanska

**Introduction:** Children with juvenile idiopathic arthritis (JIA) have impaired physical development: delay in linear growth (mostly in systemic (sJIA) and polyarticular (pJIA) subtypes) and body weight disturbances (mostly in pJIA).

**Objectives:** To determine the effect of biological therapy on the main parameters of physical development in children with JIA: weight (W), height (H) and body weight index (BWI).

**Methods:** The data from the Ukrainian register of JIA patients receiving biological therapy were analyzed. 339 patients with different JIA subtypes were enrolled since 2014. 67,3% patients received as a therapy adalimumab (ADA); 27.9% - tocilizumab (TOZ) and 4.8% - etanercept (ETA).The mean age of patients with pJIA is10,59 ± 1,9 ys, sJIA - 10,72 ± 2,3 ys. Mean disease duration of pJIA is 9,03 ± 3,1 ys, sJIA - 6,89 ± 3,3 ys respectively. Patients with pJIA who received ETA were younger (8,83 ± 0,9 ys; p = 0,06), hand had shorter disease duration (4,0 ± 1,6 ys; p = 0,04).

**Results:** 23% of patients with pJIA had W below 10th percentile at the time of onset of biological therapy, and 15% had H retardation. The BWI in pJIA group did not depend on the disease duration, but correlated with JADAS (r = -0.42). In sJIA H retardation was noted in 69% patients there wasn’t any W deficit possibly due to exogenous hypercorticism presence. The average H increase in pJIA patients treated with ADA after 6 months of therapy was +4,59 ± 1,1 cm, W - +3,6 ± 0,8kg, JADAS value 9,71 ± 1,7. In children with pJIA who received ETA and TOZ, the H increase after 6 months of therapy was significantly less (+4,0 ± 1,2cm and +3,57 ± 0,7cm, respectively), W gain (+2,7 ± 0,6kg and +2,41 ± 0,5kg) (p = 0,11) with comparable disease activity (JADAS 9,94 ± 2,7). After 12 months of therapy, in pJIA patients who received ADA the H gain was +7,17 ± 1,2cm, W +4,75 ± 0,7kg, JADAS 6,8 ± 0,8. The H gain in ETA group was equal (+7,0 ± 1,3cm), the W was less (+3,81 ± 0,8kg, p ≤ 0.05) respectively; in TOZ group: H +6,31 ± 1,1cm, W +3,75 ± 0,6kg and JADAS 8,58 ± 1,9 respectively. The specific feature of pJIA patients in TOZ group was higher disease activity at the baseline. In sJIA patients in ADA group disease activity changes results a greater H increase after 6 months of therapy compare to pJIA (+6,2 ± 0,9cm) (p = 0,1), and with less W gain (+2,8 ± 0,6 reduction possibly due to exogenous hypercorticism). Disease activity value was similar (JADAS 9,71). In sJIA patients in TOZ group after 6 months H changes were +3,75 ± 0,7cm (p = 0,03), W +2,78 ± 0,8kg (p = 0,38), which could be explained by the higher disease activity (JADAS 13,25 ± 2,1). sJIA patients in ADA group after 12 moths of the therapy demonstrate JADAS 7,0 ± 0,9, H and W gains were greater compare to pJIA (+9.34 ± 1,1cm,+6,34 ± 0,81kg), and more than in patients with TOZ (7,19 ± 0,9cm,+5,19 ± 0,6kg), that could be explained by higher activity (JADAS 11,71 ± 1,1). After 18 months of the biological therapy in patients with pJIA in ADA group, have H changes +10,15 ± 1,0cm and W changes 6,76 ± 0,7 kg. Fewer children (11%) had a decrease in BWI (p ≤ 0,05). pJIA patients in TOZ group, have significantly higher H changes greater: +11,71 ± 1,2 cm (p ≤ 0,05) and less in W gain: +5,22 ± 0,5 kg (p = .0,34). After 18 months sJIA patients in ADA group demonstrate significantly less gain in H (+11,0 ± 1,1cm), significantly more in W (12,33 ± 0,9kg), with JADAS value 9,50 ± 1,3. sJIA patients in TOZ group, H increase after 18 months was 10,78 ± 0,7cm, W gain +7,69 ± 0,9kg, JADAS value 15,9 ± 4,1. Growth changes in sJIA patients correlated with JADAS (r = -0,52).

**Conclusion:** A less W increase in ETA group can be explained by the lack of binding it to adiposities receptors, that causes the preferred prescription of ADA to children with hypotrophy. The effect of biological therapy on H processes in JIA is mostly due to disease activity suppression.

**Disclosure of Interest**

None Declared.

### P330 A five year U.K. tertiary paediatric rheumatology centre experience for the use of rituximab

#### Nadia K. Rafiq, Mark Wood, Valentina Leone

##### Paediatric Rheumatology, Leeds Teaching Hospitals NHS Trust, Leeds, UK

###### **Correspondence:** Nadia K. Rafiq

**Introduction:** The anti-CD20 chimeric monoclonal antibody Rituximab (RTX) depletes pre-plasma B cells. It may increase the risk of hypogammaglobulinaemia and predispose patients to infections. There have also been published reports of neutropenia subsequent to RTX. RTX is licenced for use in Rheumatoid Arthritis and ANCA associated vasculitis in adults in the U.K. Increasingly it is used off label in paediatric rheumatology patients.

**Objectives:** We aimed to describe the cohort patients treated with RTX in a tertiary Paediatric Rheumatology Centre over the last five years. We collated epidemiological and clinical features pre-RTX. Post-RTX we analysed the presence of B cell depletion, hypogammaglobulinaemia and neutropenia.

**Methods:** All patients and clinical details were identified using hospital specific electronic clinical databases.

**Results:** We identified 16 patients that had received RTX between 01/2012 and 04/2017. 2 patients had to be excluded due to unavailable data. The male: female ratio was 1:13.

6/14 patients had SLE, 3/14 polyarthitis, 3/14 autoimmune dysregulation and 2/14 granulomatosis with polyangiitis (GPA). The average age of diagnosis of all patients was 11.4 years. The average patient age at first dose of RTX was 14.8 years and the average disease duration pre RTX was 3.4 years (Table 37).

All patients were on concomitant disease modifying anti-rheumatic drugs at the time of RTX infusions.10/14 steroids, 5/14 Hydroxychloroquine, 4/14 Mycophenolate mofetil, 2/14 Intravenous immunoglobulin (IVIG), 1/14 Azathioprine, 1/10 Methotrexate and 1/14 Sulfasalazine.

4/14 (28.6%) had hypogammaglobulinaemia (IgG < 5.4g/L) after RTX. 3/14 (21.4%) within 3 months of receiving 2 doses of 750mg/m^2^ (≥1g). 1/14 (7.1%) had 4 weekly doses of 375mg/m^2^ for thrombocytopenia and developed hypogammaglobulinaemia 15 months’ post treatment and hence unlikely to be due to RTX. This particular patient had been given IVIG for thrombocytopenia pre-RTX.

Another patient was on 6 weekly concomitant IVIG for autoimmune myositis and they never developed hypogammaglobulinaemia.

Complete B cell depletion (<0.0001 x10*9/L) using very sensitive flow cytometry was achieved in 9/14 (64%) of patients, however a significant proportion 4/15 (36%) remained B cell detectable.

Neutropenia (<1.5x10*9/L) was identified in one SLE patient (1/14, 7.1%) post-RTX. They had normal levels pre-RTX, which dropped to 0.34x10*9/L, 5 months post-RTX.

**Conclusion:** The findings provide a useful insight into the use of RTX in paediatric rheumatology patients. The incidence of hypogammaglobulinaemia attributable to RTX is significant (21.4%). Neutropenia was identified in a single patient (7.1%). It isn’t possible to definitively ascertain whether this was due to disease activity or RTX. No patient with hypogammaglobulinaemia or neutropenia suffered with a serious infection, requiring hospitalisation. We did not feel IVIG replacement was indicated. This study does not support the use of prophylactic/routine IVIG replacement therapy during/after RTX.

We would recommend checking blood cell counts and immunoglobulin levels following each dose of RTX and at least monthly for 3 months following the last dose of RTX. Certainly, checking and considering IVIG replacement would be sensible in any patient presenting with serious infection following 3 months of RTX.

The limitations of this study include the retrospective design, small sample size and single centre experience.

Biologic registries to gather long-term safety data are essential. Current guidelines also recommend women of childbearing potential should use effective contraception while on RTX and for 12 months following treatment.

**Disclosure of Interest**

None Declared.

**Table 37 (Abstract P330).** See text for description

Disease	Number of Patients	Mean Age of Diagnosis (years)	Mean age of 1st RTX (years)	Mean Disease Duration pre RTX (years)
SLE	6	11.7	13.5	1.8
Polyarthritis	3	10	16.3	6.3
Autoimmune Dysregulation	3	12.3	15.3	3
GPA	2	11.5	14	2.5
Total/Average	14	11.4	14.8	3.4

### P331 Evaluation of long term safety and indications of biologic agents in juvenile rheumatic diseases: a single center experience in Turkey

#### Sezgin Sahin, Sule Bektas, Ezgi Belhan, Duhan Hopurcuoglu, Amra Adrovic, Kenan Barut, Ozgur Kasapcopur

##### Pediatric Rheumatology, Istanbul University, Cerrahpasa Medical School, Istanbul, Turkey

###### **Correspondence:** Sezgin Sahin

**Introduction:** The spectrum of diseases that biologic agents are used in treatment of juvenile rheumatic diseases has been increasing gradually since the first approval of etanercept for JIA in 1999. Inadequate response to synthetic disease-modifying antirheumatic drugs (DMARDs) is the most frequent reason of biologic agent introduction. These agents significantly improved the outcome of not only JIA patients but also other rheumatic diseases. There is still lack of data regarding the safety of these therapies in juvenile rheumatic diseases.

**Objectives:** We aimed to report our single center experience regarding the usage indications and long term safety of various biologic agents in Turkish children.

**Methods:** We have included all the patients treated with biologic agents at least 6 months due to various rheumatic diseases from January 2003 to October 2016. Demographic characteristics, diagnosis and all adverse events (AEs) were obtained from medical records of the patients.

**Results:** Overall, 502 patients (281 female) with rheumatic diseases have been received biologic therapy since 2003. The most frequent diagnosis was juvenile idiopathic arthritis (n = 393), followed by autoinflammatory diseases (n = 55), uveitis (n = 20), connective tissue diseases (n = 18), vasculitis (n = 12), inflammatory bowel disease (n = 3), idiopathic recurrent pericarditis (n = 1). While the median age at disease onset was 5.4 years (range 1 month-17.6 years), median age at onset of biologic therapy was 10 years (range 1 – 20 years). Among the patients treated with biologic agents, 355 subjects (70.8%) showed complete response to the first agent and so, used only one biologic drug: 244 etanercept, 35 adalimumab, 21 anakinra, 20 infliximab, 13 rituximab, 12 canakinumab, 9 tocilizumab, 1 belimumab. However, 29.2% of the subjects (n = 147) switched to another biologic agent due to side effect or inefficacy. Of the 147 subjects who switched, 58 (39.4%) finally remained on adalimumab therapy, 37 (25.2%) patients on canakinumab, 24 on tocilizumab (16.3%), 21 on etanercept (14.3%), 5 (3.4%) on infliximab and 2 (1.4%) on anakinra after various biologic drugs. At least one AE occured in 177 subjects (%35.2) and 53 patients (%10.5) had at least one serious AE. While 21 patients received prophylaxis for tuberculosis, only six patients developed tuberculosis disease on biologic therapy.

**Conclusion:** We have observed, the usage of biologic therapies with a wide range of indications in Turkey. The frequency of AEs and SAEs are similar with the previous studies. Other than tuberculosis disease, these agents seem fairly safe in juvenile rheumatic diseases. We recommend close screening of patients on biologic therapy particularly in countries where tuberculosis disease is still widespread as in Turkey.

**References**

1. Barut K, Adrovic A, Şahin S, Kasapçopur Ö. Juvenile Idiopathic Arthritis. Balkan Med J 2017; 34(2): 90-101.

2. Al-Mayouf SM, Alenazi A, Al Jasser H. Biologic agents therapy for Saudi children with rheumatic diseases: indications and safety. Int J Rheum Dis 2016; 19: 601–6.

3. Sen ES, Ramanan AV. Biologic drugs in pediatric rheumatology. Int J Rheum Dis 2016; 19: 533-5.

**Disclosure of Interest**

None Declared.

### P332 The experience of oral and parenteral methotrexate therapy in the juvenile idiopathic arthritis patients

#### Betul Sozeri^1^, Amra Adrovic^2^, Nuray Aktay Ayaz^3^, Serife Gul Ercan^3^, Hacer Arıkan^1^, Aslı Kaplan^2^, Kenan Barut^2^, Mustafa Cakan^3^, Sezgin Sahin^2^, Ozgur Kasapcopur^2^

##### ^1^Pediatric Rheumatology, University of Health Sciences, Istanbul, Umraniye Training and Research Hospital, Istanbul, Turkey; ^2^Pediatric Rheumatology, Medical Faculty of Cerrahpasa, Istanbul University, Istanbul, Turkey; ^3^Pediatric Rheumatology, University of Health Sciences, Kanuni Sultan Suleyman Training and Research Hospital, Istanbul, Turkey

###### **Correspondence:** Betul Sozeri

**Introduction:** Methotrexate (MTX) is the cornerstone disease-modifying anti-rheumatic drug (DMARD) in juvenile idiopathic arthritis (JIA). It’s efficacy for the treatment of JIA was established in the early 1990s. MTX is beneficial in around 70% of the patients. However, most of JIA patients who take MTX are reported to experience difficulties including gastrointestinal complaints and behavioral changes.

**Objectives:** The aim of this study was to determine the prevalence of MTX intolerance in a multicenter cohort of children with JIA and to assess differences in intolerance profiles.

**Methods:** A cross-sectional study of JIA patients on MTX was performed. Primary outcome was MTX intolerance, which was determined using the validated Methotrexate Intolerance Severity Score (MISS) questionnaire. The prevalence of MTX intolerance was compared between patients on MTX SC and MTX administered orally (PO).

**Results:** We included 189 patients with JIA, 124 (65.6%) were female. Their mean age at onset was 7.3 ± 4.8 years, age at study entry 10.2 ± 4.4 years. The median disease duration (first to third quartiles) 12 (10-42) months. The majority JIA category was oligoarticular (54%).

MTX intolerance (score ≥6 and at least one anticipatory, associative or behavioural symptom) was present in 67 (35.5%) patients. The majority intolerant patients was female (60%), it is statistically significant (p = 0.038). The mean age was 10.7 years higher than the tolerant patients (9.4 years) (p = 0.046).

The median MISS score of the SC group was higher than the median score of the PO group (p < 0.05). behavioural symptoms (restlessness, crying, irritability and MTX refusal combined) were more frequent among children taking MTX SC.

MTX intolerance was high in those who were taken medicine empty stomach than post prandial (P = 0.01). Also, intolerant patient’s mother’s education level is low than tolerant group.

The prevalence of MTX intolerance was high in the implementation of the health institution (p = 0.02) but the effect of the application time was not found (p = 0.46).

**Conclusion:** The frequency of MTX intolerance was high in patients who applied on an empty stomach, applied SC, and patients in the older age group in our cohort.

However, given the cross-sectional design of the study, results need to be interpreted with caution. Controlled randomized prospective studies are required in children and juvenile patients for definite recommendations for the route of application of MTX treatment.

**Disclosure of Interest**

None Declared.

### P333 Methotrexate in JIA: sustainability of early remission achieved with subcutaneous administration

#### Sarka Fingerhutova^1^, Katerina Kobrova^1^, Dita Cebecauerova^1^, Radoslav Srp^1^, Jana Franova^2^, Dana Nemcova^1^Michal Uher^3^, Pavla Dolezalova^1^

##### ^1^Department of Paediatrics and Adolescent Medicine, General University Hospital in Prague, Czech Republic, Prague, Czech Republic; ^2^University Hospital Brno, Brno, Czech Republic; ^3^Institute of Biostatistics and Analyses at the Faculty of Medicine and the Faculty of Science of the Masaryk University, Brno, Czech Republic

###### **Correspondence:** Radoslav Srp

**Introduction:** There is a lack of published evidence on the importance of methotrexate (MTX) dose and route of administration on both its efficacy and adverse events in children with Juvenile Idiopathic Arthritis (JIA).

**Objectives:** We aimed to document a long-term outcome of a cohort of patients in whom disease inactivity was induced within 24 months of MTX monotherapy.

**Methods:** Study inclusion criteria were indication of new MTX therapy for active arthritis in confirmed JIA patients younger than 18 years. Eligible patients were evaluated prospectively every 3 months for 1 year and then 6-monthly using standardized instruments for treatment response (Juvenile Arthritis Disease Activity Score (JADAS) 71) and adverse events (laboratory monitoring, Methotrexate Intolerance Severity Score (MISS)). MTX intolerance was defined by MISS ≥ 6.

**Results:** From the original cohort of 55 patients (1) 3 were transferred to adult care. The 52 patients (35 girls, median age 5.3 years, IQR 2.8;10.6) were prospectively followed for the median of 32 (IQR 28;40) months. From those, 34 (65%) achieved inactive disease (JADAS <1) within the median of 6 (IQR 4;11) months of treatment with the median MTX weekly dose of 14.4 mg/m^2^ given s.c. in 27 (79%). At the last follow-up 20/34 patients (59%) retained disease remission while 9 of them were still receiving MTX and 11 were off-treatment. From the remaining 14/34 patients (41%) who initially achieved inactivity with MTX but were active at the last follow-up only one patient relapsed on the same MTX dose while remaining 13 children relapsed after MTX withdrawal or dose reduction/change from s.c. to p.o. administration. Their therapy was then escalated individually (return to s.c./higher MTX dose and/or addition of biologic). MTX intolerance as expressed by the MISS ≥6 at 24 and 36 months of follow-up in the whole cohort was present in 9 out of 44 (20%) and none of 13 patients still treated with MTX at the last follow-up, respectively. After the first year of therapy MTX intolerance led to its withdrawal in 3/52 patients only.

**Conclusion:** In this extension of our previously reported study we have documented a longer-term outcome of a subgroup of MTX monotherapy responders who achieved early disease inactivity while receiving mostly parenteral MTX. In this JIA cohort disease remission was maintained by nearly two thirds of patients after more than 2.5 years of follow-up. Although noticed in about 20% of cases MTX intolerance did not have any major impact on MTX therapy outcome. These results support our current approach to MTX therapy that consists of using preferably parenteral MTX from the beginning of treatment at the dose around 15 mg/m^2^.

(1) Fráňová J et al. Methotrexate efficacy, but not its intolerance, is associated with the dose and route of administration. Ped Rheumatol 2016, 14:36

Supported by the grant NT149-3/2013

**Disclosure of Interest**

None Declared.

### P334 A novel transdermal hydrogel-forming microneedles patch a promising system to deliver methotrexate for JIA patients

#### Ismaiel A. Tekko^1,2^, Ryan F. Donnelly^1^, Helen McCarthy^1^, James McElnay^1^, Madeleine Rooney^2^

##### ^1^School of pharmacy, Queen’s University Belfast, Belfast, UK; ^2^Centre for Experimental Medicine, Queen’s University Belfast, Belfast, UK

###### **Correspondence:** Ismaiel A. Tekko

**Introduction:** Methotrexate (MTX) is a cornerstone of therapy worldwide for juvenile idiopathic arthritis [1], yet its current drug delivery systems (oral and subcutaneous) are still challenging and associated with serious side effects [2]. Transderaml route is an attractive alternative drug delivery route, yet the outer layer of the skin presents a strong barrier for transdermal permeation of such drug (MTX is hydrophilic and its log P -1.85). In a previous in-vitro study [3], a novel PVA-based hydrogel-forming microneedles (HF-MNs) array was developed and showed to be a promising efficient transdermal delivery system for MTX. However, its application in an in-vivo setting (rat model), suggested further optimisation is required to form more rigid and strong MNs and special patch design is needed to make its application feasible.

**Objectives:** To optimise PVA-based HF-MNs array properties to improve its rigidity and develop a special HF-MNs patch design loaded with the required MTX dose and evaluate its efficiency to deliver MTX in an in-vivo setting.

**Methods:** HF-MN arrays composed of 11 x 11 MNs of conical shape with MNs measuring (729.5 ± 11.2 um) in height, 300 um in-width at the baseplate and interspacing 200-250 um were fabricated as per [3] and optimised to form a stronger HF-MNs array. The fabricated HF-MNs arrays were characterised in terms of their mechanical strength, swelling capacity and permeability to MTX in an in-vitro setting employing Franz-type diffusion cells.The optimised HF-MNs arrays were then used to build up a novel HF-MNs patch loaded with MTX dose of 0.5 mg/kg. The ability of the fabricated HF-MNs patch to deliver MTX in an in-vivo setting was evaluated using Sprague Dawley rats (n = 6). One patch was applied onto the back of each rat with 20 μl of deionised water added into the MTX wafer reservoir and then removed after 24 hours. 200 μL blood samples were collected over 48 hours from application at predetermined time intervals and then analysed for MTX and its polyglutamates (PGs) content.

**Results:** The optimised HF-MNs arrays showed superior properties in terms of their mechanical strength and swelling capacity in comparison with those reported in previous study [3]. Upon applying the HF-MNs patch onto skin at the back of the rat, MNs were inserted into skin without breaking or bending. Furthermore, upon removing the patch. Also, it was noticed that HF-MNs completely swelled and the MTX wafer was dissolved indicating that the HF-MNs after insertion into skin imbibed skin interstitial fluid forming continuous conduits between the dermal microcirculation and an attached MTX wafer reservoir within the patch. This allowed the drug to be delivered in a controlled sustained manner. Interestingly, the HF-MNs arrays were removed intact from skin and no marks of irritation or severe inflammation were observed at the application site.

**Conclusion:** The optimised PVA-based HF-MNs with the novel patch design has shown to be a promising, minimally invasive transdermal drug delivery system which could be used to deliver MTX efficiently to JIA patients. However, further investigation is required to prove its efficiency and safety and advantage in comparison with other administration routes (oral, subcutaneous) after single and multiple application using larger number if rats

References

[1] Becker, M. L. (2013). Role of methotrexate in juvenile idiopathic arthritis: where we have been and where we are going. International Journal of Clinical Rheumatology, 8(1), 123-135.

[2] Klein, A., et al. Efficacy and safety of oral and parenteral methotrexate therapy in children with juvenile idiopathic arthritis: an observational study with patients from the German Methotrexate Registry." Arthritis care & research 64.9 (2012): 1349-1356.

[3] Tekko I et al. Delivering methotrexate transdermally employing novel PVA-based hydrogel-forming microneedles for treatment of Juvenile Idiopathic Arthritis: In-vitro studies. 2016. Poster session presented at BSPAR Annual Conference 2016, Manchester, UK.

**Disclosure of Interest**

None Declared.

## Uveitis

### P335 Prevalence and characteristics of uveitis in a cohort of Portuguese juvenile idiopathic arthritis patients

#### Francisca Aguiar^1,2^, Raquel Ferreira^1,2^, Mariana Rodrigues^2,3^, Luís Figueira^2,4^, Luís Torrão^2,4^, Iva Brito^2,3^

##### ^1^Rheumatology, Centro Hospitalar São João, Oporto, Portugal; ^2^Faculty of Medicine of Porto University, Oporto, Portugal; ^3^Pediatric Rheumatology Unit, Centro Hospitalar São João, Oporto, Portugal; ^4^Ophtalmology, Centro Hospitalar São João, Oporto, Portugal

###### **Correspondence:** Raquel Ferreira

**Introduction:** The most common extraarticular manifestation seen in juvenile idiopathic arthritis (JIA) is uveitis. Most cases involve the anterior chamber, are asymptomathic, bilateral and chronic, and can be associated to ocular complications and permanent vision loss.

**Objectives:** To study the prevalence, characteristics and outcome of JIA-associated uveitis in a population of portuguese patients.

**Methods:** Retrospective observational study including patients with JIA diagnosis followed in our Paediatric Rheumatology Unit. Clinical, demographic and laboratory characteristics were retrospectively collected by consulting medical records. JIA classification was based on ILAR criteria/1997-2001. ANA positivity was considered when at least 2 titers were ≥ 1/100. Remission status at follow-up was defined using the Wallace preliminary criteria. Statistical analysis was performed using Student t-test, Mann-Whitney test, Chi-square or Fisher test (SPSS 23.0). Significance level was set as <0.05.

**Results:** 94 patients were included, 67% (63) were female, with a mean age at diagnosis of JIA of 7.2 ± 4.4 years and mean follow-up of 8.9 ± 6.6 years.

The most common subgroup was oligoarticular persistent JIA (38.3%), followed by polyarticular (25.5%), systemic (13.8%), psoriatic (10.6%), enthesitis-related arthritis (7.4%) and oligoarticular extended JIA (4.3%).

Uveitis was observed in 14 patients (14.9%); they were diagnosed with arthritis at a median of 4.0 years of age (range 1-11) and uveitis at 5.0 years (range 1-21). Children with uveitis were primarily female (71.4%), most of them had positive ANA (71.4%), 11 had oligoarticular persistent form, 1 oligoarticular extended, 1 poliarticular-rheumatoid factor negative and 1 had enthesitis-related arthritis. All had anterior uveitis, most of them chronic (78.6%), bilateral involvement (71.4%) and several ocular complications (cataracts (57.1%) synechiae (28.6%), band keratopathy (35.7%) and glaucoma (21.4%) and macular oedema (7.1%). In what concerns treatment with immunomodulating agents, all patients were treated with oral or subcutaneous methotrexate and 6 patients (42.9%) had to initiate biologics because of ocular involvement: 1 with infliximab that was switched for adalimumab because of drug-induced sarcoidosis, and all the rest with adalimumab. At last follow-up 7 patients had active disease, 6 patients were in remission on treatment and 1 patient was in remission off treatment.

Regarding the prevalence of uveitis, statistically significant differences were found associated with the form of JIA, being more prevalent among patients with oligoarticular JIA (p = 0.019), ANA positivity (71.4% vs 36.3%, p = 0.015) and earlier onset of disease (5.2 ± 3.7 years vs 7.6 ± 4.5, p = 0.045). Other factors such as gender, disease duration and disease activity at last evaluation were not associated with the presence of uveitis.

**Conclusion:** The incidence of uveitis in our study was similar to that reported in the literature. Our results also confirm that the frequency of uveitis is higher in those patients with oligoarticular JIA, positive ANA and younger age at onset.

**Disclosure of Interest**

None Declared.

### P336 Cyclophosphamide, a sight for sore eyes: a case of refractory uveitis successfully treated with cyclophosphamide

**Abstract withdrawn**

### P337 Efficacy of infliximab in treatment of resistant uveitis in pediatric rheumatologic diseases

#### Shima Salehi^1^, Reza Shiari^1^, Masoud Soheilian^2^

##### ^1^Pediatric Rheumatology, Shahid Beheshti Univesity of Medical Sciences, Tehran, Iran, Islamic Republic of; ^2^Ophthalmology, Shahid Beheshti Univesity of Medical Sciences, Tehran, Iran, Islamic Republic of

###### **Correspondence:** Shima Salehi

**Introduction:** Uveitis is a broad-spectrum term unfolding a group of inflammatory diseases that causes swelling and destroys eye tissues. The term “uveitis” is coined because the diseases often target a part of the eye called the uvea. With respect to anatomical involvement of the eye, uveitis is classified into anterior, intermediate, posterior and pan uveitis; however, anterior subtype tends to be most prevalent subtype according to various literatures. Classification of uveitis however is based on various factors such as duration, course and etiology. Management of uveitis can be done via medical therapy or even surgical intervention. As earlier mentioned, various medications have been shown promising results with respect to treatment of uveitis. Anti-TNF agents like etanercept, adalimumab, and Infliximab (INF) have been successfully used in the management of treatment refractory uveitis and several retrospective case series describe the use of INF in pediatric uveitis.

**Objectives:** Current study tends to assess efficacy of Infliximab in treatment of resistant uveitis as manifestations of eye involvement in the context of rheumatic diseases.

**Methods:** 15 patients with refractory Uveitis in the context of Rheumatologic disease were included in our study via convenient sampling method. Firstly, PPD test, CXR, complete blood counts, liver and kidney function tests, urine analysis, ESR, ANA, ACE, anti-CCP, HLA-B27, HLA-B51, HLA-B5, Anti ds-DNA and RF,were done for all patients to rule out the infections and find the possible etiology of Uveitis. Participants were administered premedication 30 minutes before each injection. Subsequently in one till two hours, all participants received Infliximab (5mg/kg) intravenous infusion with close monitoring. Then at 2^nd^ and 6^th^ weeks of treatment, and tfinally every 6-8 weeks their injections continued. Intervals during maintenance treatment were decided with respect to response to treatment for each patient.Ophthalmological examination was done with respect to severity of the activity atbaseline: between 1-3 months intervals.

**Results:** According to results of current study, 13 (86.7%) patients positively responded to Infliximab without experiencing any side effect (Table 38).

**Conclusion:** It appears plausible that Infliximab maintains high rate of improvement among refractory uveitis patients.

**Trial registration identifying number:** Shahid Behehsti University of Medical Sciences

**Disclosure of Interest**

None Declared.

**Table 38 (Abstract P337).** Response of Infliximab based on type of Uveitis

Type of Uveitis	Not Improved	Improved
Oligoarticular JIA	1 (11%)	8 (89%)
Psoriasis JIA	0 (0%)	3 (100%)
Behcet’s Disease	0 (0%)	1 (100%)
Primary Uveitis	1 (33%)	2 (67%)

### P338 A long-term greek study assessing the safety and efficacy of adalimumab in pediatric patients with refractory non-infectious uveitis

#### Maria Trachana^1^, Polyxeni Pratsidou-Gertsi^1^, Evaggelia Koulali^2^, Nikolaos Kozeis^3^, Paris Tranos^3^, Aggeliki Doudou^4^, Sofia Androudi^5^

##### ^1^First Department of Pediatrics, Aristotle University Thessaloniki, Pediatric Immunology and Rheumatology Referral Center, Ippokration Hospital, Thessaloniki, Greece; ^2^Department of Ophthalmology, Ippokration Hospital, Thessaloniki, Greece; ^3^Ophthalmica, Aristotle University, Thessaloniki, Greece; ^4^First Department of Ophthalmology, Aristotle University, Thessaloniki, Greece; ^5^Department of Ohthalmology, University of Thessaly, Larisa, Greece

###### **Correspondence:** Maria Trachana

**Introduction:** Non-infectious uveitis (NIU) can either be the single manifestation of deranged immunity or can escort another autoimmune disease, usually Juvenile Idiopathic Arthritis (JIA). Treatment with DMARDs may hinder or alleviate complications and further damage development that lead to impaired vision. However, 15-40% of NIU are refractory to these medications and respond to targeted management with biologics, namely anti-TNFs. Recently, Adalimumab received an indication for posterior adults’ uveitis but is still an off- label drug for pediatric NIU with limited publications.

**Objectives:** To assess the safety and efficacy of Adalimumab (ADA) in Greek pediatric patients with NIU, resistant to conventional regimen.

**Methods:** Pts’ inclusion criteria: Non-responders NIU pts with or without any concomitant autoimmune entity and with at least 3 mo-activity despite conventional DMARDS. The following data were retrospectively collected: demographics, co-existence of autoimmune diseases, number of eyes involved, previous and concurrent medication, existing complications, lag-time between ADA commencement and disease quiescence, drug survival (pt-years), adverse events. Time point 0 was determined as the 1^st^ dose of ADA and the preselected post-treatment assessment points were 3,6, 12 mo and annually thereafter.

**Results:** 25 pts (F:M 14:11, median age 10.16 yrs) with refractory NIU (JIA associated n = 14, pure NIU, n = 11) were enrolled (Table 39). The median age was significantly lower in JIA-NIU (5.1 *vs* 8.75, t = 0.03), but did not differ between the 2 Groups (t = 0.23). The previous regimen post diagnosis were steroids (n = 16), methotrexate (n = 14), methotrexate plus cyclosporine (n = 16) and azathioprine (n = 6). 6/25 pts received 2 therapeutic cycles of ADA, thus resulting to 31 cumulative study cycles). The ADA exposure was 84.26 yrs (06.0-9.5, mean 3.37 yrs/pt). Three mo post-treatment, disease quiescence was achieved by all 31 cycles, at 6-mo by 29/31, by the 12^th^ mo by 19/21 and by the 24^th^ mo by 15/16..A waning response was noted in 4 pts, 1.5-6.5 yrs post -treatment. Impressively, 4 pts were able to discontinue ADA due to a 3yr clinical remission, but ¾ flared 0.34-1.5yr later. 8 patients underwent surgical intervention during the study for cataract (n = 7) and cataract + glaucoma (n = 1). ADA additionally controlled pre-existing NIU complications (n = 18 pts) in 11/18 pts according to the last assessment. No serious AE (SAE) or events of Specific Interest (ESI) were recorded (severe infections, anaphylaxis).

**Conclusion:** Finding of the study indicate that ADA administration in Greek pediatric pts with NIU proved to be efficacious, as it led to the rapid regression of the ocular inflammation, within 3 mo and safe, in respect to SAE and ESI. Thus, ADA although still off label, can be an alternative for refractory NIU cases.

**Disclosure of Interest**

None Declared.

**Table 39 (Abstract P338).** Disease characteristics in the 2 NIU Groups

	JIA-associated NIU (n = 14)	Pure NIU (n = 11)	Total
Bilateral (pts)	12/14	11/11	23/25
Total eyes with NIU	24	22	46
Location:			
Anterior/Posterior/Panuveitis	12/6/2	2/4/2	16/7/2

## Vasculitides

### P339 High risk of cardiac coronary aneurysm in young children with incomplete Kawasaki disease

#### Maria Mossberg^1^, Aladdin J. Mohammad^2, 3^, Mårten Segelmark^4^, Robin Kahn^1^

##### ^1^Department of Pediatrics, Clinical Siences Lund, Sweden; ^2^Department of Rheumatology, Clinical Siences Lund, Lund University, Sweden, Lund, Sweden; ^3^Department of Renal medicine, Vasculitis and Lupus Clinic, Addenbrooke’s Hospital, Cambridge, UK; ^4^Department of Medical and Health Sciences, Linköping University, Linköping, Sweden

###### **Correspondence:** Robin Kahn

**Introduction:** Kawasaki disease is the second most common primary vasculitis in children. The incidence of Kawasaki disease varies depending on ethnicity, with the highest incidence in the Japanese population (265/100 000 children under 5 years of age). The incidence in Caucasians is reported to be 5-15/100 000 children under 5 years of age. Children with Kawasaki disease has a high risk of coronary artery aneurysms if untreated. Since the introduction of intravenous immunoglobulin as treatment for Kawasaki disease this risk has substantially decreased.

**Objectives:** To study the incidence of Kawasaki disease in a population-based study of a defined geographical area in southern Sweden and to elucidate which children are at most risk to develop coronary artery aneurysms.

**Methods:** All children in Skåne, the southernmost part of Sweden (population of 1.29 million; 21,4% aged <18 years), with a clinical suspicion of Kawasaki disease were identified in a comprehensive regional healthcare register. All case records for the children (0-17 years) assigned an ICD-10 code of M30.3 (Kawasaki Disease) were reviewed to ascertain diagnosis. Only patients diagnosed between 2004 and 2014 were included. Data on age, gender, date of diagnosis, date of first symptom, presenting clinical features, relevant clinical chemistry, serology and radiology results at time of diagnosis and all histopathology reports, treatments and coronary ultrasound was collected. The study was approved by the Regional Ethical Review Board for southern Sweden (2010-517, 2015-153 and 301-2007).

**Results:** In total, we identified 79 patients clinically treated as possible Kawasaki disease. Out of these patients, 47 had symptoms compatible with complete Kawasaki disease and 32 with incomplete Kawasaki disease, defined according to the American Heart Association diagnostic criteria. Nine out of the 32 patients with incomplete symptomatology had affection of the coronary arteries. Thus, 56 patients meet the diagnosis criteria for Kawasaki disease. The estimated incidence rate of Kawasaki disease was 5.5 (95% CI 3.9 - 7.1) per 100 000 children under 5 years of age. The median age at diagnosis was 1.5 years (IQR 0 - 2.75, range 0 - 16). Nine cases (16.1%) developed coronary artery aneurysm and all of these patients were 5 years or younger. Of these 9 patients, 6 patients had symptomatology consistent with incomplete Kawasaki disease and three with complete Kawasaki disease. Thus, in the patients with a clinical suspicion of Kawasaki disease but with incomplete symptomatology, 18.8% develop coronary aneurysms. Whereas in the children with complete Kawasaki disease, only 6.4% developed coronary aneurysms.

**Conclusion:** The risk to develop coronary aneurysm is disturbingly high in small children with Kawasaki disease in Sweden, especially in the children with incomplete symptomatology. Both early and intense treatment is important to try to reduce this risk. In addition, it is important to repeatedly conduct coronary ultrasound on all children with a clinical suspicion of Kawasaki. In light of the data presented, we suggest that the spectrum of Kawasaki disease in Sweden significantly differs from that seen in East Asia.

**Disclosure of Interest**

None Declared.

### P340 Juvenile polyarteritis nodosa presenting as refractory Kawasaki disease: a diagnostic challenge in young infants

#### Parichat Khaosut^1^, Pantipa Chatchatee^1^, Hiroshi Chantaphakul^2^

##### ^1^Paediatric Allergy and Immunology, Chulalongkorn University, Bangkok, Thailand; ^2^Allergy and Immunology, Faculty of Medicine, Chulalongkorn University, Bangkok, Thailand

###### **Correspondence:** Parichat Khaosut

**Introduction:** Juvenile polyarteritis nodosa (PAN) is a rare, necrotizing vasculitis primarily affecting small to medium-sized muscular arteries. Kawasaki disease (KD) is a relatively frequent vasculitis in children, particularly in thoseyounger than 5 years of age. Both juvenile PAN and KD share overlapping clinical features. However, PAN is considered as a chronic disease and frequently affects multiple organs, which require more aggressive treatment.

**Objectives:** We present an unusual case of polyarteritis nodosa first diagnosed with typical Kawasaki disease. This report demonstartes some clinical manifestations, which help differentiate these two conditions and facilitate appropriate treatment.

**Methods:** Case review of the medical notes.

**Results:** A 2-year-old boy presented with typical characteristic features of Kawasaki disease, which fulfils 4 out of 5 diagnostic criteria. His first echocardiogram done on the 15^th^ day of illness revealed significant pericardial effusion and coronary aneurysms. He was treated with three doses of IVIG, pulse methylprednisolone and infliximab. His persistent fever, abdominal pain with mucous-bloody diarrhea and renal involvement suggested an alternative diagnosis. Based on computer angiographic abnormalities and other laboratory investigation, finally this patient met criteria for juvenile PAN. He received and steroid, later continued maintenance therapy with azathioprine. At present, he remains on anticoagulation for persistence of the aneurysms without evidence of active vasculitis.

**Conclusion:** KD and PAN can have overlap of symptomatology. Nevertheless, there are some clinical manifestations that could help differentiate one from the other. Prompt treatment should be managed according to the severity of symptoms in order to prevent serious complication. Our report underscores the need to consider the diagnosis of PAN in KD patients who are refractory to the standard treatment.

The written informed consent for the publication of these details was obtained from the participants.

**Trial registration identifying number:**

**Disclosure of Interest**

None Declared.

### P341 Hypomorphic RAG1 defect in a child presented with pulmonary hemorrhage and digital necrosis

**Abstract withdrawn**

### P342 To predict the risk of recurrence of Henoch-Shönlein purpura in children

#### Gulden Yildirim Usta^1^, Emre Usta^2^, Ismail Islek^3^, Betul Sozeri^3^

##### ^1^Pediatrics, University of Health Sciences, Istanbul, Umraniye Training and Research Hospital, Istanbul, Turkey; ^2^Pediatrics, University of Health Sciences, Istanbul, Zeynep Kamil Training and Research Hospital, Istanbul, Turkey; ^3^Pediatric Rheumatology, University of Health Sciences, Istanbul, Umraniye Training and Research Hospital, Istanbul, Turkey

###### **Correspondence:** Betul Sozeri

**Introduction:** Henoch-Schönlein purpura (HSP) is a disease with multi-system involvement and clinical course.

**Objectives:** This study was performed with the aim to develop an index (disease recurrence index) serving to determine the common clinical and laboratory features of HSP and to predict the risk of recurrence on the base of signs observed at the onset of clinical course.

**Methods:** The subjects who were followed-up for at least six months with HSP between the dates of 2010 and 2015 were included in the Ümraniye Training and Research Hospital Pediatric Health and Diseases Clinic. The file records of the cases were evaluated retrospectively. The diagnosis was confirmed on the diagnostic criteria of the European Rheumatism Association on HSP. Cases’ age, sex, clinical and laboratory features at the onset and in the long term progress of course and treatments details and follow-up of patients were recorded.

**Results:** : A total of 146 cases (82 males, 64 females) with a mean age of 1-17 years were evaluated. Purpuric rash was the unique common feature in all cases. Joint involvement occurred in the second degree (65.7%, n: 96) in cases and sequel improvement of joint involvement was observed. The incidence of gastrointestinal involvement was 54.8% (n: 80) and only two patients had invagination healed without surgery. Renal involvement and nephritis were in 28.7% (n: 42) and 16.6% of cases, respectively. End stage renal failure and hypertension did not develop in any cases. The recurrence rate was 14.4% (n: 21) and we observed higher scores associated with recurrence at the onset of clinical course.

**Conclusion:** Gastrointestinal tract and renalinvolvement rarely cause significant complications in the Henoch-Schönlein purpura. The initial clinical and laboratory features are indicative of risk of recurrence.

**Disclosure of Interest**

None Declared.

### P343 Comparison of patients with iga vasculitis in Mediterranean and continental part of Croatia

#### Sasa Srsen^1^, Marijan Frkovic^2^, Vitomir Metlicic^1^, Luka Stricevic^1^, Marija Pecnjak^3^, Martina Held^2^, Marija Jelusic^2^

##### ^1^Department of Pediatrics, University Hospital Centre Split, Split, Croatia; ^2^Department of Pediatrics, University Hospital Centre Zagreb, Zagreb, Croatia; ^3^Department of Pediatrics, General Hospital “Dr. Josip Bencevic”, Slavonski Brod, Croatia

###### **Correspondence:** Sasa Srsen

**Introduction:** IgA vasculitis is the most often vasculitis in the paediatric population, presenting predominantly with palpable purpura, arthritis, abdominal pain and renal involvement.

**Objectives:** To compare characteristics of paediatric patients with IgA vasculitis treated in two tertiary centres in different parts of Croatia: Split (Mediterranean part of Croatia) and Zagreb (continental part of Croatia) during ten years period.

**Methods:** Historical chart review of patients treated in University Hospitals in Split and Zagreb from 2006 until 2015.

**Results:** During observed period of time 160 children were treated due to IgA vasculitis in University Hospital Zagreb (81 boy and 79 girls), and 135 in University Hospital Split (66 boys and 69 girls). Most of the patients had a monophasic disease (129 in Zagreb and 120 in Split), 23 patients in Zagreb and 11 in Split had one relapse of disease, 6 patients in Zagreb and 4 in Split had two relapses, while 2 patients in Zagreb had three relapses of the disease. The largest number of patients was between 3 and 8 years old with a peak number of patients at the age of 4. Mean age of patients (average +/- standard deviation) was 6.68+/-3.01 in Zagreb and 7.11+/-3.86 in Split. A number of patients treated per year were between 11 and 24 in Zagreb and 10 and 20 in Split. The peak incidence of IgA vasculitis was observed in February and October in Zagreb (26 and 25 patients), but with a stable number of patients from August until June. In Split peak incidence was in November (23 patients) with a predominance of disease in the period from October until February and lower number of patients in rest of the year. All of the patients in both centres presented with a characteristic rash. In Zagreb 62.7% of patients had arthritis, 33.1% gastrointestinal involvement, 17.5% renal involvement and 4.7% of boys had affected scrotum, while in Split those numbers were 68.9%, 32.4%, 8.6% and 8.1% in the same order. 15 patients with renal involvement in Zagreb underwent renal biopsy and 9 of them had IgA nephritis (5 subclass II according to Haas and 4 subclass III). 8 patients in Split underwent renal biopsy. 1 of them had IgA nephritis subclass I, II and V according to Haas, 3 had subclass III and 1 had subclass IV. Rash was most often present on legs (98.8% in Zagreb, 100% in Split), buttocks (52.7% in Zagreb, 59.7% in Split) and arms (31.7% in Zagreb, 31.5% in Split), while it was more seldom on trunk (10.2% in Zagreb, 10.7% in Split) and face (6.5% in Zagreb, 5.4% in Split). IgA vasculitis was preceded by infection in 59.4% of patients in Zagreb and 58.3% in Split, most often respiratory tract infection (47.5% of patients in Zagreb, 49% in Split), while other (gastrointestinal tract, urinary tract and other) were seldom. Most often isolated cause of infection was beta-hemolytic streptococcus in Zagreb and Streptococcus pneumoniae in Split. Antistreptolysin O titer was positive in 29.1% of patients in Zagreb and 52.6% of patients in Split. The largest number of patients in Zagreb was treated with non-steroid anti-inflammatory drugs (NSAID) (50.6%), 45% were treated with corticosteroids, and 1.9% of patients with immunosuppressives, while in Split 32% of patients were treated with corticosteroids, 28.8% with NSAID and 2.9% with immunosuppressives.

**Conclusion:** Patients with IgA vasculitis treated in two tertiary centres in Croatia, one in Mediterranean and one in continental region, had similar characteristics. We noticed more often the appearance of the disease in autumn and winter in the Mediterranean part of Croatia, while in continental although there were more patients in autumn and winter, a number of patients was stable during the whole year. There were fewer patients with renal involvement than expected in Mediterranean area.

**Disclosure of Interest**

None Declared.

### P344 CMV infection as a complication of gpa immunosuppressive therapy in the teenage patient: diagnostic and therapeutic challenges

#### Maria B. Tomaszek, Aleksandra Rybkowska, Aleksandra Sobiesiak, Violetta Opoka-Winiarska

##### Department of Paediatric Pulmonology and Rheumatology, Medical University of Lublin, Lublin, Poland

###### **Correspondence:** Maria B. Tomaszek

**Introduction:** The cytomegalovirus (CMV, human herpesvirus 5, HHV-5) is a common pathogen that is widespread all over the world. The seropositivity of CMV reaches a level of around 60% in highly developed countries, but stands at more than 95% in the developing world.

The primary infection in immunocompetent patients is self limited or asymptomatic. CMV, like all herpes viruses, can establish long lasting latent infections. The possibility of the reactivation of the infection in patients receiving immunosuppressive therapy is a diagnostic and therapeutic challenge. There are few studies of CMV infection in patients with GPA. Latest reports indicate that those studies that do exist have reported a high risk of the heavy course of the disease and a poor prognosis, especially if the diagnosis of CMV is delayed.

**Objectives:** This study presents a patient suffering from granulomatosis with polyangiitis (GPA) which was complicated with CMV pneumonia.

**Methods:** Retrospective analysis of patient’s history.

**Results:** The diagnosis was made using the GPA criteria: pulmonary involvement (nodules, cavities, fixed pulmonary infiltrates), histopathology showing granulomatous inflammation within the wall of the artery, an ANCA positive result and renal involvement (proteinuria).

The initial therapy involved a combination of glucocorticosteroids (GC), Cyclophosphamide and IVIG with initial remission of symptoms. During maintenance therapy with Azathioprine and low-dose GC, exacerbation was observed. High-resolution computed tomography (HRCT) of the lungs revealed widespread pulmonary infiltrates. A bronchoscopy with an endobronchial ultrasound (EBUS) revealed alveolar bleeding in the both lungs.

CMV DNA was detected by the PCR method of analysing bronchoalveolar lavage and a lung tissue sample. The specific IgM and IgG antibodies in the sera were also detected. Furthermore, DNA of the human cytomegalovirus in the urine was found. Plasma CMV- DNA viral was not detected. Differential diagnoses were conducted but all tests were negative.

Following the diagnosis of CMV pneumonia, the patient was started off on a course of Ganciclovir and the administration of GC was continued without Azathioprine.

After two weeks of receiving Ganciclovir, the patient was examined and the DNA of the human CMV was not found in the urine or plasma. The specific IgM and IgG antibodies in the sera were still detected. After one month of antiviral therapy, immunosuppressive treatment (Cyclophosphamidum) was restored. At this time the patient’s condition was good and their effort tolerance was normal.

**Conclusion:** In presented case study, the correlation between GPA, immunosuppressive therapy and CMV infection was a threat to health and life of our patient. Currently, no guidelines exist concerning the monitoring and treatment of CMV infections in paediatric patients with rheumatic diseases. In light of current knowledge, symptoms of the primary viral infection or virus reactivation have to be sought out particularly during immunosuppressive therapy in order to improve chances of timely diagnosis. It is worth remembering that the symptoms of infection might be atypical. This strategy would improve the patient’s prognosis, significantly

**Disclosure of Interest**

None Declared.

### P345 Pediatric-onset Behҫet’s disease: an experience from a tertiary-care centre in North India

#### Pandiarajan Vignesh^1^, Rakesh Kumar Pilania^1^, Avinash Sharma^1^, Deepti Suri^1^, Anju Gupta^1^, Biman Saikia^2^, Ranjana Walker Minz^2^, Amit Rawat^1^, Surjit Singh^1^

##### ^1^Allergy Immunology Unit, Pediatrics, Postgraduate Institute of Medical Education and Research, Chandigarh, India; ^2^Immunopathology, Postgraduate Institute Of Medical Education and Research, Chandigarh, India

###### **Correspondence:** Pandiarajan Vignesh

**Introduction:** Behҫet’s disease (BD) is an inflammatory systemic disorder characterised predominantly by oromucosal and skin manifestations. Pediatric-onset BD (PED-BD) constitutes around 3.3-36% of cases, and the clinical symptoms may overlap with autoinflammatory disorders. Clinical manifestations may vary among different population and ethnicities.

**Objectives:** To describe the clinical features, laboratory parameters, treatment measures, and outcome of PED-BD cohort from a tertiary care centre in North India.

**Methods:** Case records of children with PED-BD (1998-2017) were retrieved from the clinic files of the Pediatric Rheumatology Clinic, Advanced Pediatrics Centre, Postgraduate Institute of Medical Education and Research (PGIMER), Chandigarh, India. PGIMER serves as a federally funded tertiary-care referral centre for North India. The retrospective analysis of the case files was done and the parameters including- age of onset, sex, clinical manifestations, inflammatory parameters, and treatment outcomes were analysed. All the patients underwent ophthalmology evaluation for detection of uveitis. The presence of HLA-B51 was assessed by polymerase chain reaction (PCR) in all patients. The diagnosis of BD was based on the Diagnostic criteria from the Behçet syndrome research committee of Japan (1987 Revision).

**Results:** Thirteen (13) children had suspected PED-BD based on clinical consensus, out of which 10 were classified as BD. Three (3) children had only ocular manifestations (posterior uveitis-2, panuveitis-1 and retinal vasculitis-3) and positive HLA-B51 by PCR. Seven (7) were classified as possible BD, 3 had incomplete BD. The mean age of onset of clinical manifestations and diagnosis of BD was 5.1 years (range: 0.25-10.5 years), and 7.7 years (range: 3-15 years), respectively. Mean follow-up is 3.2 years (range: 0.2-15 years). Male female ratio was 4:1. Recurrent oral ulcers were the commonest manifestation (9/10) and it was the commonest symptom at the disease onset (9/10). Uveitis was the initial and presenting manifestation in one of the patients (10%). Genital ulcers were seen in 4 children and skin manifestations were noted in 4 children. Positive pathergy test was observed in 4 children (40%). Skin manifestations noted were non-infective pustulosis (3), folliculitis (3), acneiform nodules (2), pyoderma gangrenosum (2), and panniculitis (2). Histopathological evidence of small vessel leukocytoclastic vasculitis was noted in skin biopsies of all 4 patients with cutaneous manifestations. Inflammatory colitis was seen in 3 children. Terminal ileal ulcer was noted in one patient. Uveitis was noted in 3 children and all were boys. Ocular manifestations noted were retinal vasculitis (3), posterior uveitis (2), and panuveitis (1). Arthritis was noted in 3 children (ankle-3, knee-1, hip-1, elbow-1, wrist-1). Bilateral symmetrical subclavian artery stenosis was noted in a female child. HLA-B51 was positive in 4 children. Colchicine, thalidomide and dapsone were used in the management of oral ulcers in 7, 1, and 1 patients, respectively. Response to colchicine was observed in 5 patients. Azathioprine was used as a steroid sparing therapy in 7 children (3- uveitis, 2- gut inflammation, 1- large vessel vasculitis, 1- pyoderma gangrenosum). Gut inflammation and pyoderma gangrenosum lesions showed a good response, whereas, a significant response to eye lesions with azathioprine was observed in only one child. Cyclosporine was used in the management of uveitis in 2 children with moderate success. One child died due to pulmonary haemorrhage, however, the angiography did not demonstrate a pulmonary artery aneurysm. Thrombotic manifestations were not seen in our cohort.

**Conclusion:** We describe the first case series of PED-BD from India. A significantly higher proportion of male children (80%) was noted in our cohort. Arterial stenotic lesions mimicking Takayasu arteritis, though well reported in adults, is described for the first time in a cohort of PED-BD.

**Disclosure of Interest**

None Declared.

### P346 Polyarteritis nodosa in children, clinical characteristics and course, in a third level hospital in Mexico City

#### Ana V. Villarreal, Talia Diaz Prieto, Yuridiana Ramirez Loyola, Enrique Faugier Fuentes, Maria Del Rocio Maldonado Velazquez

##### Pediatrics Rheumatology, Hospital Infantil De Mexico Federico Gómez, Ciudad de Mexico, Mexico

###### **Correspondence:** Ana V. Villarreal

**Introduction:** Classical Polyarteritis Nodosa (PAN) was defined as necrotizing inflammation of médium or small sized arteries without vasculitis in arterioles, capillaries, or venules. There is currently a lack of data describing the epidemiology of childhood PAN; however, systemic PAN is generally considered the third most common systemic vasculitis encountered in children.

**Objectives:** To describe clinical characteristics and course of the disease in a cohort of Mexican children diagnosed with childhood Polyarteritis Nodosa.

**Methods:** A transversal descriptive study in a cohort of patients with childhood Polyarteritis Nodosa attendend during a 7 years period (2010-2017) in Hospital Infantil de México Federico Gómez, México City

**Results:** We found 6 patients diagnosed with PAN during this time period. The minimum age at diagnosis was 48 months, halft of them were female (50%), all patients from Hispanic ethnicity, the mean of days until unset of the symtoms until the diagnoses were 1200 days. 100% (n = 6) reported fever, and 83% (n = 5) presented skin disease at the time of diagnosis, 66%(n = 4) presented arthritis on physical examination, no hepatitis B or streptococcal infection was found in any patient. 2 patients with an exclusive cutaneous condition, 50% has a skin biopsy, and 83% has anormal panangiography, one with aggregated uveitis, and n = 4 with a renal condition undergoes a renal biopsy that reports the presence of diffuse necrotizing proliferative glomerulonephritis, one received treatment with cyclophosphamide boluses (total of 5), the second patient received treatment with cyclophosphamide and prednisone, two patients recived infliximab. One patient with systimic PAN developed malignant Hipertension and gastrointestinal involment and very agresive course. The 2 patient diagnoses exclusive cutaneous PAN started treatment with prednisone and subsequently added azathioprine and methotrexate, all patients have shown an adequate evolution.

**Conclusion:** There is a lack of data describing the clinical characterisitics and course of childhood PAN.Patients with PAN in Mexico present a clinical course similar to those described in the literature. Poor response to treatment related to delayed diagnosis and gastrointestinal involment was observed in this patients.

**Disclosure of Interest**

None Declared.

### P347 Simultaneous occurrence of Kawasaki disease and salmonella infection with a positive dengue serology

#### Vijay Viswanathan^1^, Paramanand Andankar^2^, Neha Gupta^2^, Ishita Singh^3^, Mahesh Rajguru^2^, Srinivas Laxmivenkateshaiah^4^

##### ^1^Pediatrics (Pediatric Rheumatology), Jupiter Hospital, Thane, India; ^2^Jupiter Hospital, Thane, India; ^3^Pediatrics, Jupiter Hospital, Thane, India; ^4^Pediatric cardiology, Jupiter Hospital, Thane, India

###### **Correspondence:** Vijay Viswanathan

**Introduction:** Kawasaki disease (KD) is a predominantly medium vessel vasculitis with a predilection for the coronary arteries. Proposed etiology include a host of infectious causes including bacterial, viral and fungal agents (superantigen theory) coupled with genetic predisposition and autoimmunity. Co- occurrences of associated infections are described but relatively infrequent.

**Objectives:** We report a case of Kawasaki disease with concurrent salmonella infection and a dengue positive serology

**Methods:** A two-year-old male child presented with a history of fever for 7 days, irritability and abdominal distension since 2 days. Physical examination revealed evidence of non-purulent bulbar conjunctivitis, erythematous cracked lips with mucositis, dorsal edema of feet and hepato splenomegaly.

Investigations revealed anemia, leucopenia, thrombocytopenia with elevated acute phase markers with hypoalbuminemia and transaminitis. His ultrasonography of abdomen showed organomegaly, gall bladder hydrops with evidence of ileoileal intussusception. Blood culture grew salmonella typhi.Also a dengue serology (IgM) came positive. Antibiotics along with supportive care was commenced in view of the positive blood culture. Suspecting an incomplete Kawasaki disease, a 2 D echocardiogram was ordered which revealed significant dilatation of coronaries (LAD2.8 mm Z score 3.7, LMCA 4.8mm Z score 6.5, RCA 2.1 mm Z score 0.8). With no improvement and persistent fever spikes after 72hours, he was treated with intravenous immunoglobulin (2 gms/kg) with a significant clinical improvement within 24 hours of the commencement infusion.Repeat echocardiogram at 8 weeks showed complete resolution of coronary dilatation.

**Results:**

**Conclusion:** Few case reports of KD with dengue fever has been reported. However, none with blood culture proven salmonella has been reported so far. Dengue positive serology in addition was extremely striking. Such a rarity coupled with features of both small vessel vasculitis (intussusception) and medium vessel vasculitis (KD) makes this case extremely unique.

It is rare to have two diseases present at the same time with an additional inflammatory manifestation. Whether salmonella typhi with a positive dengue serology was a coexisting infection or the potential organism triggering the development of KD is debatable. However, this case concurs with the theory regarding the possible role of microorganisms in the etiology of KD.

**Disclosure of Interest**

None Declared.

**Table 40 (Abstract P 347).** See text for description

Hb	9.1	8	8.9	8.3	9.4	9.3
WBC	7420	3120	10540	8860	9600	12300
Neutrophils	34	32	47	54	45	47
Lymphocytes	56	58	44	40	46	47
Platelets	98000	56000	104000	170000	255000	513000
CRP	107			19		6.7
ESR	50	55		45		
SGPT	99		223	177	145	88
Albumin	2.3					

### P348 Unusual infections and Kawasaki disease: cause, correlation, or coincidence?

#### Vijay Viswanathan^1^, Ishita Singh^2^, Aniket Deshmukh^2^, Sudhir Sane^2^, Lakshmivenkateshaiah Srinivas^3^

##### ^1^Pediatrics (Pediatric Rheumatology), Jupiter Hospital, Thane, India; ^2^Pediatrics, Jupiter Hospital, Thane, India; ^3^Pediatric Cardiology, Jupiter Hospital, Thane, India

###### **Correspondence:** Vijay Viswanathan

**Introduction:** Kawasaki disease (KD) is a predominantly medium vessel vasculitis with a predilection for the coronary arteries resulting from an interaction of a host of infections (superantigen theory), genetic predisposition and autoimmunity. Co-occurrences/associated unusual infections with KD has not been reported so far.

**Objectives:** We describe two cases of KD with amoebic liver abscess and one case with salmonella typhi managed at a tertiary care centre in Western India

**Methods:** Case 1

A 4-year-old male child was referred with persistent fever for eighteen days and abdominal pain. Ultrasonography abdomen had revealed an abscess (4cm x 4.8cm x 3.8cm) involving the right hepatic lobe. He had received multiple antibiotics and amoebicidal drugs. Clinical examination revealed mucositis, pedal edema, and bulbar conjunctivitis. Laboratory findings revealed anaemia, neutrophilic leucocytosis, thrombocytosis, elevated acute phase reactants and hypoalbuminemia. A transthoracic 2 D echocardiogram showed significantly dilated coronary arteries with right coronary artery aneurysm (RCA- Z score 4.31), left main coronary (LMCA- Z Score 3.22), left anterior descending artery (LAD-Z score 2.55). Immuno-hemagglutination (IHA) test for IgG Amoeba antibody was positive. With no response to antibiotics and the positive cardiac findings, IVIG was administered at 2 gm/kg with complete resolution of symptoms.

Case 2

A 5-year-old female child presented with fever of 7 days, mucositis with a strawberry tongue, left anterior cervical lymph node enlargement (>1.5 cm), and pedal edema. A history of a macular rash and non-purulent conjunctival congestion was elicited. Laboratory findings were suggestive of KD; 2D ECHO showed (LMCA- Z score 0.06), (LAD -Z score of 1.91) and RCA -Z score of 5.97). IVIG was administered with a diagnosis of KD. Post IVIG, with persistent fever spikes and tender hepatomegaly, USG showed a 7.4 X 6.5 X 6.4 cm liquefied abscess. IHA for IgG amoeba antibody was positive. Percutaneous drainage of 200ml fluid with an ‘anchovy sauce’ like appearance with parenteral amoebicidal led to resolution of symptoms.

Case 3

A 14-month-old female child presented with fever for 9 days, mucositis, edema, and non-purulent conjunctivitis. Investigations were suggestive of inflammation and transaminitis. Blood culture grew salmonella typhi. Antibiotics were commenced and a suspicion of incomplete KD was considered. 2D ECHO showed coronary dilatation (LMCA- Z score 1.7), (LAD -Z score of 2.6) and RCA -Z score of 2.7). with loss of tapering. With no response to the 1^st^ dose of IVIG, a 2^nd^ dose was administered with complete resolution of symptoms.

**Results:**

**Conclusion:** Co-occurrence of amoebic hepatitis or salmonellosis with KD has not been described in medical literature so far. Whether amoebiasis or isolated salmonella was a coexisting infection or the potential organism triggering the development of KD is debatable. However, these cases strengthen the theory regarding the possible role of microorganisms in the etiology of KD. Also, associated infections could be a possible predictor towards incomplete/atypical presentation, unusual course and possibly IVIG resistance.

**Disclosure of Interest**

None Declared.

**Table 41 (Abstract P348).** See text for description

	Case 1	Case 2	Case 3
Hb (gm/dl)	7.6	7.6	7.5
WBC (/UL)	16.86	21.41	21
Neutrophils (%)	44	83	44
Lymphocytes (%)	36	11	50
Platelets (/UL)	1071	580	309
CRP (mg/L)	49.5	380	30
ESR (mm/hr)	112	90	53
SGPT (U/L)	14	22	58
Albumin (gm/dl)	2.5	1.5	1.9

